# Experience and perceptions of mental ill-health in people with epilepsy in rural Ethiopia: A qualitative study

**DOI:** 10.1371/journal.pone.0310542

**Published:** 2024-12-13

**Authors:** Ruth Tsigebrhan, Charles R. Newton, Medhin Selamu, Charlotte Hanlon

**Affiliations:** 1 College of Health Sciences, Department of Psychiatry and WHO Collaborating Centre in Mental Health Research and Capacity-Building, Addis Ababa University, Addis Ababa, Ethiopia; 2 Centre for Innovative Drug Development and Therapeutic Trials for Africa (CDT-Africa), College of Health Sciences, Addis Ababa University, Addis Ababa, Ethiopia; 3 Department of Psychiatry, University of Oxford, Warneford Hospital, Oxford, United Kingdom; 4 Centre for Global Mental Health, Health Services and Population Research Department, Institute of Psychiatry, Psychology and Neuroscience, King’s College London, London, United Kingdom; University of the Witwatersrand Johannesburg, SOUTH AFRICA

## Abstract

**Introduction:**

Understanding the lived experience of mental health and illness in people with epilepsy has been little investigated in Africa and yet is essential to inform person-centered care. The aim of this study was to explore the experiences mental ill-health in the contexts of the lives of people with epilepsy in rural Ethiopia.

**Methods:**

A phenomenological approach was employed using in-depth individual interviews with PWE. Participants were selected purposely. The setting was Gurage Zone in south-central Ethiopia, where efforts had been made to expand access to mental health and epilepsy care through integration in primary health care. Thematic analysis was used.

**Result:**

Twenty-two participant were interviewed (8 women, 14 men). The following themes were identified: expression of ill-health; the essence of emotions; the emotional burden of epilepsy and aspirations and mitigating impacts. Participants reported multiple bodily (e.g., fatigue) and emotional (e.g., irritability, sadness) experiences that were tied up with their experience of epilepsy and not separable into physical vs. mental health. Occupation and social life difficulties were interconnected with emotional and bodily sickness. Emotions were considered inherently concerning, with emotional imbalance spoken of as a cause or trigger for seizures. These emotional burdens resulted in difficulties fulfilling occupational and social life obligations, in turn exacerbating the epilepsy-related stigma experienced by others. Participants sought to mitigate these interconnected psychosocial impacts through finding spiritual meaning in, or acceptance of, their experiences, drawing on family care and, for some, emotional support from health professionals.

**Conclusions:**

People living with epilepsy in this rural Ethiopian setting experience various emotional, financial, occupational and interpersonal problems that are crucially interwoven with one another and with the experience of epilepsy. A people-centered approach to support the recovery of people with epilepsy requires consideration of mental health alongside physical health, as well as interventions outside the health system to address poverty and stigma.

## Introduction

People-centered care is at the heart of recommendations for effective primary health care services but cannot be achieved unless broader psychosocial impacts of chronic physical health conditions such as epilepsy are recognized and addressed [1]. Epilepsy is a chronic neurological condition characterized by recurrent seizures that is found worldwide but is particularly burdensome in low- and middle-income countries (LMICs) [2–4]. Recent epidemiological data estimate the lifetime prevalence of epilepsy to be 7.60 per 1,000 population (95% confidence interval (CI) 6.17 to 9.38) globally [2], which is consistently higher in LMICs [2, 4], although it is estimated to be 5.2 per 1000 population in Ethiopia [5]. Epilepsy is associated with high comorbidity of mental health conditions, a relationship that has been the focus of enquiry for decades [6]. Depression and anxiety disorders are the most common mental health conditions co-occurring with epilepsy [7]. The pooled prevalence of mood and anxiety disorders is estimated to be 35% and 26%, respectively [8]. A systematic review of hospital-based studies carried out in sub-Saharan Africa (SSA) estimated the prevalence of depression in people with epilepsy to range from 7% to 49% [9].

The epidemiological interrelationships between epilepsy and mental health are complex, with bidirectional relationships between epilepsy and mental health conditions [10–12], as well as common risk factors for both contributing to comorbidity. Studies from high-income countries have identified several psychosocial risk factors for mental health conditions in people with epilepsy that operate alongside potential biological mechanisms [13–15]. Epilepsy-specific risk factors for depression include epilepsy-related stigma, longer duration of epilepsy, poor adherence and polytherapy with anti-seizure medications, and poor adjustment to the condition [14, 15]. Furthermore, a history of depression is related to poor control and the development of intractable epilepsy compared to those without depression [12]. PWE may also have increased exposure to more generic risk factors for depression, including low education, unemployment, stressful life events, and financial constraints [13–15]. On the other hand, comorbid mental health conditions are associated with a range of impacts that can result in poorer seizure control (e.g., poorer medication adherence, reduced health seeking behavior), poorer quality of life and increased disability [16–18]. The quality of life of PWE is more strongly affected by the presence of a comorbid mental health condition than seizure control [19].

Despite this large body of quantitative evidence enumerating the various psychosocial problems related to epilepsy and mental ill-health, there has been minimal qualitative research in LMICs to explore how their interconnections and impacts are experienced by individuals within the contexts of their lives [20]. Rather, qualitative studies have focused on the subjective experience of epilepsy or mental ill-health alone. The expression and manifestations of mental ill-health in PWE are inevitably affected by culture and social context and by the nature of epilepsy itself and the available treatments and care systems [21, 22]. In a systematic review of qualitative studies of the experiences of people with epilepsy in LMICs, thirteen studies (four from SSA) described the perspectives of people with epilepsy on the cause of epilepsy, the treatment and their social relationships [23]. The review found that external supernatural factors (e.g., demonic possession, curse from God) were considered to be important causes of epilepsy but that most people with epilepsy pragmatically drew on both traditional and biomedical models of treatment and that their social life had been affected in both positive (e.g., increased depth and extent of social support) and negative ways (e.g., stigma) [23]. Notably, in some of the reviewed studies, depression, stress and anger were mentioned as triggers for seizures [23]. The emotional toll (sadness and suicidality) of epilepsy was also described in a qualitative study conducted in a rural part of Ethiopia [24].

The experiences and conceptualizations of depression and anxiety have similarly been investigated in some countries of sub-Saharan Africa, including Ethiopia, but not in the context of people with epilepsy [25, 26]. A recent systematic narrative synthesis of 25 qualitative studies explored the explanatory models of depression in people living in diverse sub-Saharan African countries [25]. The review concluded that depression was rooted in social adversities, especially relationship and financial problems [25]. The review also noted that manifestations of depression in people with physical health conditions (e.g., HIV, pregnancy) were uniquely described as “thinking too much” [25].

Understanding how mental ill-health is experienced and responded to within the context of living with epilepsy is essential for a more people-centered approach to care. Such an understanding will have important implications for the way that interventions are designed to improve recognition of, and responses to, comorbidity and to achieve outcomes that are valued by individuals with epilepsy. This approach to care is also the way forward recommended by the Intersectoral Global Action Plan on Epilepsy and other Neurological Disorders (IGAP) [27]. The mental health of people living with epilepsy has been neglected and the complex inter-relationship with epilepsy have not been explored adequately from the perspective of people with lived experience. This evidence gap is especially prominent in low income country settings like Ethiopia. Therefore, the aim of this study was to explore the lived experiences of people with epilepsy on mental ill-health and its interrelationships with epilepsy in Sodo district, Ethiopia. Sodo district, located in the Southern parts of Ethiopia, is populated with several government primary health care centers, non-governmental and community based traditional and faith based organizations [28]. Traditional and faith based healing is usually the first ports of call when people seek help for those illnesses which are thought to be caused by supernatural powers like mental illness. The main research question was how are mental health illnesses experienced and perceived by people with epilepsy in Sodo district, Ethiopia?

## Materials and methods

### Study design

A phenomenological approach was used, including in-depth interviews with PWE. We followed a social constructionist approach, as the subjective meaning and the experiences of epilepsy and mental ill-health are shaped and influenced by a person’s social life and cultural background [29]. This study design was adopted for its suitability to explore the lived experience of PWE.

### Setting

This study was conducted in Sodo district, one of the 15 districts of the Gurage zone in the Southern Nations, Nationalities and Peoples’ region (SNNPR) of Ethiopia. This zone is predominantly rural and is characterized by fertile semimountainous terrain. The majority ethnic group is Sodo Gurage, followed by Oromo. Gurage languages are spoken as a first language by 80.5% of the population but the official language of Ethiopia (Amharic) is also widely spoken. The majority of inhabitants are Muslims (51.0%), followed by Orthodox Christians (41.9%) and Protestants (5.8%).

Sodo district is 105 km away from the capital city of Ethiopia (Addis Ababa) and has an estimated population of 194,253 persons (96,120 men; 98,133 women) according to the 2022 projections. At the time of data collection (June and July 2018), there were a total of 58 *kebeles* (the lowest administrative unit) in the district. Most inhabitants are farmers and small-scale traders. Wheat, sorghum, maize, beans, teff and false banana (*enset*) are commonly harvested crops in the area [28]. Enset has additional cultural importance for all Gurage people. Food insecurity is chronically present in the district due to population pressure and degraded land [30].

Sodo district has one primary hospital, eight health centers and fifty-eight health posts. The health centers are staffed by health officers and nurses, whereas the health posts are run by female health extension workers (HEWs). Health centers each serve a population of 20,000–25,000 people. In collaboration with the Programme for Improving Mental health carE (PRIME) project [31], an integrated district-level mental health care plan was developed, implemented, scaled up and evaluated. This programme focused on care for people with epilepsy, psychosis, depression and alcohol use disorders and expanded mental health care through integration within primary health care services [31]. Facility-based mental health care was based on the World Health Organization’s mental health Gap Action Programme (mhGAP) [32], whereby health officers and nurses were trained to assess and treat people with the selected priority conditions.

In addition to biomedical services, there are also multiple informal, traditional and religious healing centers located in the district [28]. Religious centers, in particular ‘holy water places’ linked to the Orthodox Church, are commonly visited for health problems (including mental illness) that are thought to have a supernatural cause.

### Study population and sampling

Participants for the qualitative study were selected purposively from a cohort study of people with epilepsy who had been identified in the community and had engaged with integrated epilepsy care within the primary health care (PHC) facility. Details of recruitment into the larger cohort study from March, 2017 to June, 2018 have been published previously [18] and will now be described briefly. Community key informants and health extension workers (HEWs) were trained to recognize people who may have active convulsive epilepsy, augmented by house-to-house screening by HEWs. Screen-positive individuals were referred to the nearby PHC facility where the diagnosis of epilepsy was confirmed by trained PHC workers. After confirmation of the diagnosis, the person was invited into the cohort study, and clinical care was provided regardless of whether the person was included in this study.

Recruitment into the study took place at the point when the person had attended the health centre for health care upon the recommendation of the health extension worker or community key informant. The person was free not to attend the health centre and thus their attendance was assumed to be of their own volition and motivated by their interest to receive treatment. At the point when the person was confirmed to have a diagnosis of active convulsive epilepsy by the primary care worker, the primary care worker introduced the person to the project psychiatric nurse. The psychiatric nurse then screened for eligibility, assessed for capacity to consent to participate in the study and obtained informed consent before the person was recruited into the study.

For this qualitative study, purposive sampling was conducted based on gender, age, area of residence, and level of mental distress, as indicated by scores on the culturally validated Self-Reporting Questionnaire, 20-item version [33–36]. Data was collected till theoretical saturation was reached and approximately 10–20 participants were estimated to be included. Participants were recruited and interviewed from December 6^th^, 2019 and February 15^th^, 2020.

### Data collection

Individual in-depth interviews were conducted to develop a rich understanding of the day-to-day, lived experience of mental ill-health in relation to epilepsy in this context. The topic guide (S1 File) explored the person’s experiences of being diagnosed with epilepsy, having seizures and epilepsy treatment, and its relation to emotional distress (including how this manifested) and mental ill-health; their social life and experiences of social exclusion, and any psychosocial problems they faced; how their self-care, occupation, social life had changed due to epilepsy, how they viewed/thought about themselves and their health and wellbeing, how they sought to manage emotional disturbances, and their perspectives on how care could be improved to achieve better quality of life.

Interviews were conducted in Amharic, the official language of Ethiopia and the language most widely spoken in the Gurage zone, and audio-recorded if permission was given. The principal investigator (RT) and a research assistant with extensive experience in qualitative research carried out the interviews. The location for interviews was a private room in a nearby health facility. Informed written consent was obtained, and privacy and confidentiality were maintained at all times. The interviewers had experience addressing sensitive topics, responding appropriately to any distress displayed by the respondent. Field notes on the participants’ emotional expressions, nonverbal communication and the feelings of the interviewer during the interview were also recorded.

### Data management and analysis

Audio-recordings were transcribed into Amharic. Transcripts were then reviewed alongside the audio recordings as a quality check. The transcripts were then translated into English.

Thematic analysis with an inductive coding approach was used. OpenCode software was used for data management. The first author read and reread the transcripts to become thoroughly familiar with the dataset. After the initial ideas were written down, codes were generated by the first author and an independent coder, initially using two selected transcripts. Discussion between coders led to refinement of the codebook, as well as discussion with CH and MS. The first author then coded the remaining interviews, further modifying the codebook and recoding where necessary. See S2 File for the final code book. After all data were coded, themes and subthemes were generated by grouping codes that tapped into similar concepts. Following further discussion with CH and MS, other emerging themes and subthemes were generated by grouping and organizing the codes. Participant quotes were selected to illustrate the themes and subthemes. After forming a thematic diagram of all the themes and subthemes, they were tested against the whole dataset. We further agreed on the naming and defining of the final themes to be reported. The themes were also then interpreted by looking deep into the patterns, meanings and implications. All quotes were anonymised (participant identification number only), and efforts were made to ensure that the individual was not identifiable from any quotes.

### Researcher’s reflexivity and positionality

Confidence in the final findings of the study is reinforced by stating an honest and informative account of the lead researcher involved in the field work (RT). Being female, being a mental health professional and coming from the city could have intimidated some participants. The information disclosed within interviews may have been influenced by participants’ expectations and the sociocultural and power differences between the interviewer and participants. The setting of the interviews (in a hospital) could have signaled to the participants that the focus was on biomedical ways of knowing. The interviewer began data collection with background knowledge of the epidemiological evidence for the role of various psychosocial factors in triggering or maintaining mental health conditions, which could have influenced the emphasis of the interview and analysis.

### Ethics approval and consent to participate

All the methods were performed in accordance with **the Declaration of Helsinki**.

Ethical approval was obtained from the Institutional Review Board of the College of Health Sciences, Addis Ababa University and the Research Ethics Committee of King’s College London (HR-15/16-2434). Informed consent and witnessed verbal consent (for non-literate participants) were sought after adequate information was given about the study. The information sheet contained all the details of the study and potential benefits and risks associated with being part of the study. Adequate amount of time was given to process the information, ask questions and no coercion to participate was implied.

In papers drawing on the data, participant quotes were referred to by the identification number and any personal identifying data were removed to ensure that the individual is not identifiable from the quote. Confidentiality was further maintained by storing the data in locked cabinets and password protected computers. The identification number was linked to the personal details of the participant in a separate document which were kept separately (hard copy) and password protected (electronic copy).

Participants who expressed suicidal ideation or mental distress were referred for evaluation and management in the locally available mental health service.

## Result

### Socio-demographic characteristics

Twenty-two participants were interviewed. A summary of their sociodemographic characteristics is presented in Table 1. The mean age was 32.5 years. One third of the participants were women (8/22), half had a primary education, and the majority were farmers (18 out of 22 participants).

**Table 1:** Sociodemographic characteristics of participants.

Characteristics		Frequency % (n)
Age	In years	18–55
Gender	Males	63.6 (14)
	Females	36.4 (8)
Place of residence	Urban village	54.5 (12)
	Rural	45.5 (10)
Education	Non-literate	18.2 (4)
	Primary	54.5 (12)
	Secondary	27.3 (6)
Occupation	Farmer	81.5 (18)
	Daily laborer	22.7 (5)
	Housewife	22.7 (5)
	Student	18.2 (4)
Marital status	Single	40.9 (9)
	Married	54.5 (12)
	Other	4.5 (1)

The following themes and subthemes emerged from the data: expression of ill-health; the essence of emotions; the emotional burden of epilepsy; and aspirations and mitigating impacts.

### 1. Expression of ill-health

*1*.*1*. *Distress and dysfunction*. Participants experienced multiple emotional and bodily difficulties, as well as problems with their power of thinking, particularly when seizures were recurrent. Many of these difficulties were experienced as emotional in nature, for example, including feelings of anxiety (15 participants/22), anger or irritability (17 participants/22), sadness (17 participants/22), disappointment (8 participants/22), hopelessness or feeling helpless (15 participants/22) or suicidal (2 participants/22).

*“It is just an anxiety on my heart….an anxiety on my heart. Even now I sometimes get sick even though I am taking the drug. I get sick. I feel anxious in my heart*…… *even if I am taking the drugs, the anxiety is on my head day and night*.” ID-020, F

*“I will be simply depressed*. *When I will be depressed*, *it will go to my head*, *it (the epilepsy) doesn’t like depression*. *I was told the disease didn’t like depression and then I will try to make my mind free but I can’t*, *at that time I will seize and I will be hurt a lot*.*” ID-02*, *F*

For some participants, there were also unusual and frightening experiences of atypical images and scenes. They were well aware of these unusual experiences and have described them signaling the inevitable occurrence of their seizure.

*“… it is just I see something in front of me and then I fell. I get very sick… I feel like someone is speaking to my face, I feel like something is coming on me. I see a lot of stuff…” ID-022, M*

Struggling to think clearly, solve problems, concentrate or remember important things was concerning to participants when it interfered with their ability to accomplish their daily tasks. Similarly, for bodily difficulties, including fatigue, loss of balance, headache, poor appetite and pain in the stomach.

*“I feel tired. I take one tablet in the morning and one in the evening. I just feel tired. I sleep again and again. I don’t feel like working. I wake up to go to the toilet then I sleep again….. I wonder ‘how am I going to raise my kids?’ and I try to work a little then I sleep” ID-025, M*

*“…my body is no longer present*, *my body has become weak*, *I feel like an 80- or 100-year-old man*. *Like a very old man*, *my body is all dead and weak*. *Now I just cannot run*, *I cannot even keep my hand straight*, *it becomes floppy*, *has become very weak*. *I don’t know whether it is from the way I took the drugs or whether it is from the illness… for example I had a fight with my cousins and they have hit me on the head*, *my head was hurt very badly*. *I don’t know whether it is from the wound or the illness*, *my ear drum was damaged*, *I was very sick for 4 or 5 years*. *I had a treatment at Butajira… Now I am waddling like a drunken guy*. *This has also become a problem*. *The main thing is my body has become weak*. *I cannot describe this illness*. *It has made me not to work*. *I just cannot stay still*, *I feel dizzy*.*” ID-019*, *M*

Some respondents were not at all bothered by any emotional or bodily distress, describing their life as any person living in their society without epilepsy. In these cases, they did not face any difficulties in their day-to-day activities or fulfilling their obligations.

*“Thanks to God, I had epilepsy before and then started taking the medication. Now, I take the medication every month and use one pill per day. I am fine now…… I completed my education while doing my work…. I don’t also have any problem with my social life.” ID- 012, M*

*1*.*2*. *Unified experience*. The signs of having epilepsy or mental illness were not considered as a separate illness by most of the participants. Rather, they had a unified experience of the body and the mind. They experienced both physical and mental health problems at the same time, without distinction, or the experiences followed each other closely.

*“When I have pain, I lost my consciousness. I don’t know where it touched me whether it was my leg or my hand. I feel very irritable…..what shall I do with my life? I lost my consciousness. It made me change …what shall I do? shall I hang myself?” ID- 0017, F*

*“When I am sick*, *it means I am gone seize*. *It shows me a sign when it is going to seize me*. *When I am going to seize it showing me signs*, *I will be depressed and then lose interest in everything*. *After that*, *I will know myself and sit down or I will be suddenly sick*.*” ID-02*, *F*

Mental distress was also not considered a separate entity from their social or economic struggles. All the physical, psychological and financial manifestations were intertwined.

*“R: … After I fall (experience a seizure), I start having a headache… And the headache makes me anxious, then I feel like I can’t live with this disease.*

I: What about difficulty sleeping?

*R*: *I have that too*. *I sleep better when I have money in my pocket; if I don’t have money*, *I think about what I should do to get the money*. *That is my biggest worry*.*” ID- 023*, *M*

Much of the concern that participants linked to these difficulties was related to their ability to live adequately or according to their expectations. Emotional distress was linked by some of the participants to the social losses that they experienced because of the effects of epilepsy.

*“I used to be much better than this. My friends bought cars, and they live a much better life. At one point I was much better than them, so I said, ‘why did I end up like this?’ …” ID- 016, M*

For others, feelings of anger were justified by their dire social and economic situation, again linking back to the root cause of epilepsy.

*“….. when I cannot fulfil everything, I have to feel angry. It is not that I like to be angry. It is a must. As I said before, as long as you don’t fulfil your needs, there is going to be a worry…”* ID- 019, M

### 2. The essence of emotions

*2*.*1*. *Epilepsy as an emotional journey*. Several participants related a detailed social account of how the first seizure happened and emphasized the extent to which this had been a significant turning point in their life, associated with strong emotions. These strong emotions were mirrored in the emotions of those around them at the time of the first seizure. Some of the participants were also overwhelmed by the sudden and unpredictable occurrence of their illness.

*“….when it began, my cousin had a graduation ceremony and I was carrying a pot to the kitchen with my cousin. They were shocked when I fell. In addition, they just worried as what it is. All of them said “take her to hospital…”* ID- 02, F

“At that time, my father died, and we went to collect wood in the forest. When we went to the forest, I fell there, which I have never experienced before. Then, after a while it is known as it is this disease, I didn’t know anything about it. It was like a dream” ID- 06, M

“*At the time when the illness started*, *I used to work at another person house*. *I was servant and I seized while grinding coffee*, *I have never felt that way before*. *…*.. *I wasn’t sick before that and then I became sick” ID-09*, *M*

*2*.*2*. *The power of emotions*. Several participants framed the onset of seizures in terms of emotional shock that resulted from a life stressor. The stressful events reported by the participants included loss of property, grief or severe financial constraints. However, it was their effect on the person’s emotional equilibrium that was linked to the onset of epilepsy.

*“I bought a land and was building a house on it then I was betrayed, that is how the anger started and with the anger came the epilepsy…. I haven’t stopped doing my job, I’m still doing my job. I think the epilepsy is related to the way I feel and think….. when I feel anxious, the epilepsy starts” ID-016, M*

*“…I think it started approximately 1986 EC*. *My older sister was sick*, *and I cried a lot*. *I do not know whether I have the disease before that*, *but after that*, *I have it…That is how it started*. *Then*, *my sister died*, *I loved her so much*, *the way she died was also very sad*, *she died suddenly*, *I cried a lot*. *At the time*, *I cried until I lost my consciousness*. *Then*, *I started to get sick that night” ID-019*, *M*

The presence of emotional turmoil was not only considered in the onset of epilepsy but was also reported to trigger seizures for most participants. Feeling depressed or anxious or feeling anger was considered to be the most potent emotion. Disruptive relationships with a close family member were reported to be the most common reason for the flaring up of these negative emotions.

*“If I have a fight with someone and if I am angry, I get sick, I feel it…. When I have a fight with my father, if I am with him and if he does something to me or insult me when we fight, I feel upset and get sick”* ID-022, M

*“When I got depressed*, *I just know it*. *Most of the time*, *I spend the day at home fearing that it might make me faint/seize*. *I hate working…It’s just that he [her husband] has no sympathy*. *He doesn’t think that I get sick if I get angry*. *If he doesn’t speak to me in good spirit*, *I get angry*. *If he speaks to me arrogantly*, *I get unconscious*. *There are times that I get unconscious while I am sitting*. *He speaks nonsense to me and then I get unconscious and for a while I couldn’t identify a person*. *I realize that I got so sick after I return to myself”* ID-011, F

### 3. The emotional burden of epilepsy

*3*.*1*. *Living apart from others*. Almost all participants reported the experience of stigma from their community or their school friends or their close family members, rooted in the fear that the illness would be transmitted to them. Only a few participants, specifically those who had never had a seizure in a public place, led their social life as any person in their society. The participants even had the apprehension that the illness would be transmitted to their family members and therefore tried to isolate themselves. In this sense, the isolating actions of society were understandable to participants.

“*………. There are other people in society who try to discriminate against me because of my illness. I mean in our area…… they will say like ‘if someone has epilepsy, don’t have a bright mind, or he is crazy’. Both you and me, there is nothing we can do. You cannot be in other persons shoe and judge them. This is what I say regarding the illness…….. One of my children, my second child, he urinates while he is sleeping. As I told you before, I am worried that his mind is not working properly. Even I am worried than him, because I have a suspicion that this illness is inherited by them (my children) and they are gone suffer*” ID-019, M

Some participants reported that they were not able to accomplish their social duties, such as going to funerals or weddings, because they were not able to contribute to the social activity in terms of both money and labor. For some, they avoided going to crowded places with the anticipation of having a seizure. For some participants, even their close acquaintances advised them not to be part of any stressful gatherings to protect them from being sick. Participants reported being irritable due to feelings of being unwanted, which in turn made it difficult to get along with their family and neighbors and reinforced their separation.

“*Because I am sick even if I was at home I don’t go to funeral places, I don’t go to weddings, I don’t go places where people talk and discuss. It (the epilepsy) does not allow* it” (ID-023, M).

*“… I was sick previously at the funeral*. *They (neighbors) said they won’t be upset if I don’t come they said that ‘please don’t come to funeral what if something happens to you’*. *All the women in the neighborhood know my situation…” ID- 020*, *F*

*3*.*2*. *Not living as expected*. The participants also reported that they were not able to work as they used to before they developed epilepsy. Some experienced the reappearance of seizures when they were involved in hard labor work, so this was purposely avoided. For some, experiences of fatigue, dizziness and excessive sleeping decreased their full potential to work. Some had a fear that they might get hurt if a seizure happened while they were at work, for example, while at the fireplace or while fetching water.

The deterioration of their capacity to work and income was devastating, especially for the male participants when they compared it to their previous preseizure life. The female participants said that when they were sick, they were not even able to do simple house chores. Their children or the neighbors were the ones who helped them. This made them feel a burden on their families.

*“Sometimes people say he is not like he used to be, he is sick, he can’t work and they discharge me. The people I work with tell people who I’m not like I used to be because I can’t climb up the roof. It has affected my work. It’s not like it used to be.” ID 016, M*

*“I can’t get close to fire*, *I can’t go to the river*, *I can’t walk a long distance*, *I can’t go anywhere*. *I have to stay around the house*. *If I fall into a fire or if I fall into a river there is no way back no one can help me*, *I will die*.*” ID- 0023*, *M*

Some participants described the consequence of having epilepsy on their education, as they were forced to discontinue school or were absent for most days. They reported that routine school activities stressed them, which later exacerbated or triggered their seizures.

*“…. I hate education when I am sick. I don’t know why I say I don’t want to go to school. I will be stressed when I enter the class. I didn’t hear when they talked and taught. I used to have good results, but I am losing now since I will be absent many times”* ID- 02, F

*“I discontinued it (my education) when I got sick*. *I feel anxious when I learn*. *Whenever there was an exam*, *I started to have a seizure*. *That is the reason I discontinued*.*” ID-022*, *M*

### 4. Aspirations and mitigating the impacts

*4*.*1*. *Anticipation of cure and a better life*. Curing seizures and ceasing mental distress were desired the most. Participants sought advice from traditional healers and spiritual treatments when their epilepsy was not managed as they wanted. A sense of helplessness was reported by some participants, feeling that they had no control over what was happening to them or what would happen next. At the same time, control of the illness and cure by an external deity remained a hope while also investing in medication.

*“Even if I am taking the drugs, the anxiety is on my head day and night. If I don’t stop the drugs, it (the seizure) is not going to relapse. They say to get to a holy water rather than the hospital, so I went to Hawasa, to Goro, to Addis Ababa Shunkuru holy water…. what can I do? I just kept saying that may God reveal everything…. What can I do? My God reveals it to me…. I just need to take these drugs, and one day it might be all cured.” ID-020, F*

*“However*, *if I want to be cured*, *for example*, *I am praying to God asking him ‘how you are going to save me*?*’*. *I am praying for God to save me*, *but I don’t know when it is going to happen at this time*. *Your pill is helping me; it makes me feel healthy” ID-05*, *F*.

They also aspired to better job opportunities and identified the importance of financial assistance for those people who had the same kind of problem as them.

*“I wish I could work with my friend. I wish there is some work created for me and do that. Society should find me a job when my friends are doing some jobs. I would be happy if I work, give me a job. Even if I was not able to work as they, it will be good if I work other jobs.”* ID-022, M

*4*.*2*. *Community responses and hopes*. Respondents spoke of the need for community respect for their right to be loved and be treated equally, like other people.

*“…they have to think and understand that we used to be equal like them. We were equal…. They should wonder what happened to this guy. It was because of the illness that he has come below us. They have to think about this. This must be improved”* ID- 025, M

Participants suggested that education of the community about epilepsy and mental illness would decrease stigma and improve the lives of people with epilepsy.

*“I would prefer to be able to teach people about this when they are in such difficult situations, along with the health workers…. The community should just tell for others in the area…. The community expected what should be done when someone fell in front of them. You should tell what they have to do, and then the community will help patients as much as possible if they know what do.”*ID-012, M

Although some participants reported community exclusion related to epilepsy, others also mentioned ways in which community members helped them manage their illness. For those participants who received emotional and financial support from their family or community members, this support played an important role in relieving their problems. Family members supported them, reminded them to take their medicine, to attend regular follow-up at the health care facility and helped with household chores.

*“I don’t do any work. He (her husband) is the only one who knows my anxiety. He is the only one. He knows about my anxiety and he is. Everything for the house”* ID-020, F

“The community is supportive and they aid. There is no community that is more supportive than this community. The people of this town are very nice. They care too much for people. I can’t tell you, they are very nice for other people. ID-03,F

*“People in society love me*. *They tell me to follow up on my treatments and take care of myself*. *I have a good relationship with people in society*.*”* ID-016, M

*4*.*3*. *Formal care for emotional support*. There were only a few participants who had received evaluations of their mental health problems or any emotional support from a primary health care professional alongside the usual education on anti-seizure medication. For those who did receive this service, psychological support and health education were very helpful.

*“The doctor could not be able to follow-up me well, but he advised me several times to not be stressed before. However, I don’t know, I try to be happy when I am with my friends but I can’t; I didn’t do that intentionally. Then, he tells my family that she is just worrying about simple things.” ID-02, F*

*“He (the health professional) asks me about my personal life*. *He says*, *“Don’t worry about the past*, *don’t get angry; you shouldn’t stress that is what gets you sick”*. *He is the only one who advises me I haven’t talked to anybody else*. *He gets very upset when I don’t take the medicine properly because he loves me*.*” ID-016*, *M*

*4*.*4*. *Acceptance of the illness*. Accepting their illness also helped some of the participants cope with the various social pressures. Justifying the occurrence of their illness as being due to an external authority provided moral relief from the feeling of helplessness.

*“Some of your family will discriminate you. There are some who care about what you eat, but there are those who wish your death. However, I don’t care; I already got it so you cannot do anything. What can I do, it is an illness that God gave me.”* ID- 05, F

*“This is a problem…*.*it is my condition that brought it on me so I don’t feel bad or angry*. *I just say thank God*, *I’ve worked when I wasn’t sick*. *I don’t feel upset when people say that because I’ve worked for many years*.*”* ID 016, M

## Discussion

In this qualitative study, we explored the lived experience of people with epilepsy regarding mental ill-health and its interrelationships with epilepsy in a rural setting in Ethiopia. The participants related many experiences of mental ill-health, ranging from negative emotions to the extent of considering ending their life, alongside multiple physical health problems. Occupation and social life difficulties interconnected with their emotional and bodily sickness. Distressing emotions were reported as the initiator or precipitator of seizures and negatively impacted their social life and functioning. Cure was anticipated by most participants, together with the hope of better job opportunities and financial assistance. Family, community and health professional support helped many, but not all, to cope with the psychosocial adversities associated with stigma and chronic illness. For some, acceptance brought relief.

Strong emotions were feared by the participants, not only considered to be a significant cause of epilepsy but also a potent trigger for recurrent seizures after biomedical treatment had commenced. This conceptualization of various emotional traits, such as fear and grief, as triggers for the onset of epilepsy was also shared by other individuals with epilepsy from LMICs [23]. The way in which bodily sickness was interpreted and responded to comprised a holistic conception of ‘epilepsy’, which included emotional, spiritual and social aspects, as well as seizures [37]. In this way, the ‘disease’ of ‘epilepsy’ was socially constructed and given meaning, which was in line with the biopsychosocial and spiritual model of illness [37, 38]. This holistic understanding is essential to inform the care needed by people with epilepsy, going beyond what can be provided by the health system in isolation.

Disappointment, irritability and anger were also prominent in some interviews, reflecting the consequences of epilepsy on their socioeconomic life. Such persistent negative feelings have been associated with a worse response to medication and increased experience of adverse effects of anti-seizure medication in studies from high-income country settings [12, 17]. In addition to the functional impacts of poorly managed seizures, bodily symptoms also had a tremendous emotional toll due to their impact on functioning. The report was of a vicious cycle of emotional turmoil triggering recurrent seizures that resulted in social and functional impairment, which in turn led to a life marred by sadness and hopelessness.

The significance of relationship problems, social disparities and poverty in the development of emotional illness, including depression, accorded with the findings from the meta-synthesis of qualitative studies from sub-Saharan Africa [25]. In the case of epilepsy, additional social and financial threats to mental wellbeing were reported in our study, arising from physical disability, the stigma-fuelled fear of having a seizure in a public place and anxiety linked to the dangers that could result from a seizure while working or alone. Reduced capacity to work and provide for their family exacerbated the perceived stigma and feelings of being a burden on their family. The misconception of epilepsy caused by supernatural entities and being contagious was seen across cultures and can further exacerbate stigma [4, 39, 40]. The anticipated stigma and social rejection by the community was the most extensively reported psychosocial burden experienced by people with epilepsy living in LAMICs [23]. In Iran, stigma and discrimination toward people with epilepsy from the community or from their family members was reported to lead them to question their identity and lead to self-debasement [40]. In our study, the consequence of stigma was displayed in social isolation and limitations on involvement in important community social gatherings. The fear of having a seizure in stressful but mandatory social commitments alongside self-stigmatization brought a sense of inadequacy and low self-esteem. The unpredictable nature of the seizure and a life with fear have been experienced by other people with epilepsy living in various socioeconomic cultures, also resulting in severe impairment of their social life [40, 41]. People with bipolar illness living in the same setting in Ethiopia reported similar kinds of stigma, contributing to social alienation and decreased seeking of biomedical care [42].

The social cost of epilepsy also extended to economic adversity. In keeping with a previous study, for those who were able to live a seizure-free life or were not publicly identified as having seizures and could function well, a normal social and economic life was possible [43]. However, the situation was different for those who were visibly affected by epilepsy. Fatigue and weakness inhibited participation in the hard labor demanded by farming. Non-optimal functioning culminated in poor financial status, to the extent of poverty for the whole household, which led to worries about how they would raise their children or earn their daily bread. It was similarly observed that depression, poor sleep quality [44] and side effects of older anti-seizure medications [45] are the major contributors to the experience of fatigue in patients with epilepsy.

Existing support systems at the family and community levels were reported to be vital for people with epilepsy in terms of improving their quality of life. However, emotional support in formal healthcare settings, where it could be accessed, was also valued. The healing nature of social and emotional support was similarly reported to be beneficial for those people who had depression from sub-Saharan African countries in the presence of having a person to share the psychosocial and economic burden [25].

Acceptance of the illness, which was apparent due to the attribution of the illness to an external authority (God), served as an adaptive coping strategy for these participants. Despite the association of the external locus of control of illness with depression from a review of the psychosocial adaptation of epilepsy [46], self-blame and isolation might be mitigated with this external locus of control of illness. Unlike acceptance of illness and engagement in self-management and problem-solving techniques, wishful thinking to reverse a condition may exacerbate helplessness and distress [46].

Our findings emphasize that psychosocial and economic concerns cannot be neglected in the comprehensive care of people with epilepsy. Care needs to address physical health, mental health, poverty and stigma in an integrated fashion. At the primary health care level routine detection, evaluation and management of the mental health needs of an individual presenting with the physical problems is required. In a previous study from Ethiopia formal care of people with epilepsy at the health center comprised of biomedical treatment with limited provision of health education [24]. Furthermore, other studies from Ethiopia have shown very low detection of depression in primary care [47] and specifically in people with epilepsy [36]. The existing health service can be strengthened by improving the capacity of PHC clinicians to detect and manage common mental disorders. Additionally, clinicians in the clinical care of individuals with epilepsy should include evaluation and management of anti-seizure medication side effects with the goal of seizure free life. Addressing associated mental health conditions through effective psychosocial interventions [48, 49] can also help to achieve optimal occupational and educational functioning. The social and economic impact of comorbid mental health concerns should also be solved to achieve optimum quality of life for the people with epilepsy living in this setting. In addition, efficient poverty alleviation interventions may be needed to bring relief from economic difficulties [50].

It is necessary for policy makers to implement people-centered care for epilepsy and other chronic health conditions within primary health care [1]. People centered and integrated health services which are comprehensive and tailored to the individual’s needs, co-ordinated across different health care providers, continuous and sustainable, and addressing mental, physical and socioeconomic problems will be important for improving the quality of life of people with epilepsy [1]. Empowering individuals, families and the population at large through promotion of the mental health is one of the Ethiopian national mental health strategies consistent with people centered care [51]. Access to effective anti-seizure medication in this rural community is also important for better health outcomes. Community education about the cause of epilepsy, and treatment options was suggested by the participants to tackle stigma against epilepsy and facilitate social integration. Public awareness interventions carried out in Ethiopia [52, 53] and the different parts of the world have been shown to increase the community knowledge and decrease stigma towards epilepsy [54]. Advocacy including public awareness campaign for better understanding of brain health and reducing stigma against people with epilepsy and neurological disorders is one of the global targets in the WHO IGAP [27]. Further research investigating the level of stigma and its impact on mental health care utilization is needed. Future research on the development and evaluation of interventions that address the various physical, emotional, social, and financial adversities is recommended.

This study has uniquely tried to describe the experience of people with epilepsy in relation to their mental health in a rural setting in Ethiopia. The credibility of the reports obtained was enhanced by giving the participants plenty of time to express their thoughts and feelings. We collected data until theoretical saturation was reached. The interviewers had much experience in conducting qualitative data. We also clearly stated our position as a clinician and researcher and how this might influence participant responses and our analyses. We sought to engage in continuous reflection on our presumptive beliefs about the relationship between mental health and epilepsy throughout the research process. The data gathered and presented were enriched, as the participants were from different sociodemographic backgrounds and at different levels of seizure control or emotional difficulties. There were also more than two researchers involved in the coding and analysis of the data. However, we were not able to triangulate data from other resources or from other methods of data collection. People with lived experience were not included as researchers. The data gathered from this study reflect only those people with epilepsy living in the rural areas of Ethiopia who were receiving care from primary health care only. Although most parts of Ethiopia are predominantly rural, this study has a limitation in that it does not represent the experiences of people with epilepsy living in major cities where there is better access to care. Similarly, the study findings may not be applicable to more remote areas where no efforts have yet been made to integrate mental health and epilepsy care within primary care.

## Conclusions

This study has shown that adult people with epilepsy living in a rural area of Ethiopia have experienced multifaceted bio-psychosocial and financial problems that are interlinked. These problems have significant consequences for their occupational and social functioning. Seizure control, mental wellbeing, social support, and maximal functioning with poverty reduction were highly regarded by people with epilepsy in rural Ethiopia.

## Supporting information

S1 File Topic guide. (DOCX)

S2 File Code book. (DOCX)

S3 File Data set. (ZIP)
