# Supplementary material for: Experience and perceptions of mental ill-health in people with epilepsy in rural Ethiopia: A qualitative study
Source: PLoS One. 2024 Dec 13;19(12):e0310542. doi: 10.1371/journal.pone.0310542 (PMC11643256; doi:10.1371/journal.pone.0310542)
Supplement: S1 File — (DOCX) [file pone.0310542.s001.docx]

**Topic Guide**

**Epilepsy and comorbid mental illness**

**Brief ID. Details**

**Date [ ] [ ] / [ ] [ ]/ [ ]**

**Participant’s ID [ ] [ ] [ ] [ ]**

**Interviewer’s ID [ ] [ ] [ ] [ ]**

**Introductions (after the consent is taken)**

I am ___________________from Epilepsy and comorbid psychiatric disorders project

The general purpose of this interview is to look at the experiences and perception of people with epilepsy on the association of mental illness and epilepsy. It will take us about 1 – 1.30 hours

Thanks for your participation.

**Socio-demographics characteristics**

Can you please tell me about yourself?

Sex

Age

Occupation

Educational status

Residence (rural /urban)

Marital status

Number of children

**Now I am going to ask you some questions about your experiences regarding your illness**

| **Domain** | **Topics and probes** |
| --- | --- |
| **Experience of being diagnosed with epilepsy and/ or comorbid mental illness** | What brought you to the health centre for the first time?  Probes:  Is there any health problems? What are the symptoms?   - What are the symptoms of your illnesses? - What are the symptoms of epilepsy? |
| **Mental illness symptoms** | - What additional symptoms do you have besides the epilepsy? (any other mental health or substance related problems or diseases?) what do you or other people call it ? - Please tell me what you know about the additional symptoms. - How did you know about it (the comorbid symptoms)? - From all the problems or symptoms you have told me which ones are the most important and why? |
| **Impact on Social/ interpersonal/ occupational functioning** | OK you have told me about …… ( both the epilepsy and the additional symptoms or mental health or substance use problems). What is the effect epilepsy (symptoms of seizure) on your life ( work, education or social life )? ( what problems did the above mentioned symptoms create in your life ? give examples of social life or work  Probes:   - What is the effect of having these symptoms on your work?   You have told me your occupation is ….. is there any difficulty to do the work as you wanted ? What were the problems?   - What is the effect of having these health problems on your education? (if the participant is attending school)?   What kind of difficulties do you face at school in relation to the symptoms that you mentioned before?   - What about the effect on social life?   (Eder, wedding, funerals)  How is your relation with your family and friends? |
| **Association of mental illness and epilepsy** | - What do you think is the relationship of these symptoms? - What is **your thought about the link between the symptoms or the problem**s? How is it related?   Probing questions   - What is the contribution of major life events or tense relationship or social life or working environment in the association of mental illness and epilepsy? - Was there any stigma or discrimination from the community? Please explain |
| **Help seeking/ coping mechanisms ( protective factors** | I understand the impact that the symptoms and I have understand that the most important one are ….. What have you done to manage the symptoms ? what about the …… (other additional symptoms)?  Probes:   - What kind of treatment facilities have you attended to manage the epilepsy? what about the other symptoms? - Why did you choose this treatment modality (ask for both the seizure and the other complaint? - What other additional activities have you done to manage the epilepsy and what about the other ……. Symptoms - What have you done to improve …. ( the psychosocial problem mentioned above). - Which one helped the most ? - Please tell me any other treatment options you know for the management of epilepsy |
| **Perception and experience at the health facility** | If the participant did not mention the biomedical treatment (going to the health facility) as option of treatment. Ask - what has been your experience to the health center?  Probes:   - Who is helping you to treat the epilepsy or the other symptoms you mentioned above? - What was the questions asked when you come for the first time? What about on follow up? - How was the health professional approach towards you? please explain - How many times have you attended the health centre? - What do you know about your prescribed medication (what kind of tablets? How many times a day? - Did you ever forget to take the drugs regularly? Why? - Have you ever missed an appointment? why - What is the purpose of regular intake of drugs? - What are the effects of the treatments? - How did you find the treatment given at the health center? - **What do you feel if the health professional asked about your personal life? what about your emotions?** - What barriers have you faced in your visit to the health centre? What about facilitating factors? - What do your family and close friends think about your treatment at the Health center? |
| **Improving comprehensive care** | Therefore   - **What should be done to improve ….(the** psychosocial problem mentioned above?)   Probes   - What would have helped more? - what can the society do to improve? - What do you think the health professionals or the health institution can do? |
| **Closing remark**  Is there anything you would like to add? Summarize  Thank the participant | |
