# Supplementary material for: Experience and perceptions of mental ill-health in people with epilepsy in rural Ethiopia: A qualitative study
Source: PLoS One. 2024 Dec 13;19(12):e0310542. doi: 10.1371/journal.pone.0310542 (PMC11643256; doi:10.1371/journal.pone.0310542)
Supplement: S2 File — (DOCX) [file pone.0310542.s002.docx]

Codebook

| Themes | Parent code | Child code | Inclusion | Exclusion | Description |
| --- | --- | --- | --- | --- | --- |
| Expression of ill-health | Psychological  distress | Anxiety  Disappointment  Feeling helpless  Not supported  Hopelessness  Isolated justifiable mood  Negative mood  Poor memory  Sleep problem  Substance use | All complaints | If it is not problems experienced by the participants | Participants have spontaneously complained of their mental health related ( psychological), social life and about their body symptoms problems they faced.  How all the problems are related |
|  | Social dysfunction | Isolated  Social life |  |  |  |
|  | Physical dysfunction | Memory and concentration problem  Fatigue  Physical illness symptoms |  |  |  |
| The essence of emotions | Onset of illness | How the mental ill health is related with epilepsy onset | Perception on emotional or mental ill health | If there is no belief or thoughts or view on the association of symptoms | Participants view / perception the relationship of mental / psychosocial problems and epilepsy |
|  | Triggering | Association with relationship  Precipitating factors\precipitating social factors |  |  |  |
| The emotional burden of epilepsy | Occupation / economic | Expense of living  Imbalance in need and reality  Life before the illness | Effect on work, social life or on the body | Not the consequence | Participants experienced the epilepsy, emotional and social consequence |
|  | Social life | Burdened  Living in the society  Stigma/ isolation |  |  |  |
| Aspirations and mitigating impacts | Hopes for self/ cure | Financial stability  Helping self and others  Hoping for cure  Improving QOL  Instilling hope  Self-expectation  Acceptance | Participants expectations and hopes from many aspects of life | If it is not helpful or not related with coping style  Not related with expectation or response | Participants experience of how they cope , the factors that has kept them going  Participants expectation on improving their overall life or their needs |
|  | Response from the society | Positive family attitude  Financial stability  Helping self and others |  |  |  |
|  | Response and expectation from the health professional(HP) | Feeling about HP interview  Good relationship with HP  HP psycho education  Interview of the HP |  |  |  |
|  | Expectation from the health facility | Knowledge on the mood  Outcome of treatment  Unrealistic treatment options |  |  |  |
| The lived Experience of having epilepsy |  |  | Not included | It is not the primary objective of this study | Participants experience when they have seizure for the first time |
