# Supplementary material for: Experience and perceptions of mental ill-health in people with epilepsy in rural Ethiopia: A qualitative study
Source: PLoS One. 2024 Dec 13;19(12):e0310542. doi: 10.1371/journal.pone.0310542 (PMC11643256; doi:10.1371/journal.pone.0310542)
Supplement: S3 File — (ZIP) [file pone.0310542.s003.zip › data set/Translation 0016.docx]

I: Hi my name is Doctor R

R: My name is MA

I: I appreciate your willingness for this interview

R: Okay

I: I will ask you some questions for the next forty to forty five minutes

R: Okay

I: What was the first issue that brought you to the health center?

R: I had epilepsy

I: aha when? What were the symptoms?

R: The symptom is anger. I was betrayed and that made me very angry. I think that is how it started.

I: aha what kind of anger? Tell me properly.

R: I bought a land and was building a house on it then I was betrayed, that is how the anger started and with the anger came the epilepsy

I: aha what were the symptoms?

R: The symptoms, at first I feel dizzy then I lose my consciousness for five minutes

I: Okay what else

R: Even after I regain my consciousness I get confused about where I am for like ten minutes then I decided to come to this health center

I: aha Okay

R: They gave me a medicine and I started getting better

I: You started getting better?

R: Yes. It has been about three years since I started taking the medicine but in between stopped taking the medicine because I thought I was better. Then it started again, the doctor was annoyed with me he said why did you stop? And told me not to do that. Now I’ve started following up properly. I come every month to take the medicine. I’m getting better now but once a day around nine (local time) in the afternoon I lose my consciousness for ten minutes.

I: Every day?

R: Yesterday I lost my consciousness once and my work is also very tiring

I: What is your work?

R: I work in construction (builder) and carpentry

I: aha how old are you?

R: I am fifty years old

I: Okay, education?

R: I have learned meserete temhirt (literacy program) in the derg era. I know how to read and write properly. I don’t have any other education

I: Very good, are you married?

R: Yes

I: Do you have kids?

R: I have kids

I: How many kids do you have?

R: I have seven kids

I: aha do you live with your wife?

R: Yes

I: In the city or in the rural?

R: We live in the rural. We have light but no water yet. It is close to the road we live like the people in the city it is good.

I: Very good, so now you’ve told me that you lose your consciousness

R: Yes

I: What else

R: I’m better now because I’m taking the medicine. I get sick when I do too much work otherwise I’m okay. The medicine is very good it helps.

I: What other symptoms does it have? Is anger a symptom or

R: When I feel exhausted I lose my consciousness for like ten minutes. After I regain my consciousness I feel weak and have pain in my arms, my legs all over my body. It makes me forget where I was, what I was doing. I normally do my job very well but when this thing comes I don’t know anything.

I: What other symptoms does it have?

R: My heart, my heart jumps. It beats very fast

I: Okay, what else?

R: I have something like a head ache but it is not a head ache. Something shouts in my head.

I: Do you have other health problems?

R: I used to drink alcohol you know people from the rural are like that. And because of that I had gastric problem, I didn’t have appetite for food. But now I don’t drink alcohol because I take medicine so I don’t have gastric problem anymore, my appetite is good.

I: aha tell me about the alcohol

R: I used to drink tela, areke (traditional alcoholic drinks). When I started taking medicine for the epilepsy I stopped drinking because I doesn’t go with the medicine. I had gastric problem because of the alcohol, I didn’t have appetite for food. After I started taking the medicine I stopped drinking so it got better. But the epilepsy is not completely gone.

I: What triggers it?

R: I haven’t stopped doing my job, I’m still doing my job. I think the epilepsy is related to the way I feel and think.

I: aha so you think it is related to anxiety?

R: Yes. When it started it used to make me fall. Now I don’t fall I just lose my consciousness as I am for a few minutes then I don’t know what happened but I don’t fall. It has been a long time since that happened.

I: Now you’ve told me you have headache, heart problem

R: Yes, also feeling of exhaustion

I: Do you think this symptoms are related?

R: I think it is related to my thought

I: How? What happens?

R: It makes me forget even though I’m taking the medicine

I: What makes you forget? How did it start?

R: I think it is the anxiety

I: anxiety tell me how

R: I don’t have a misunderstanding with my wife at home. I live a relatively good life from others in the rural. My kids are learning, I have a small land, me and my kids farm on. I don’t have a problem. I just get upset when I think about how my life was. I used to be much better than this. My friends have bought cars and live a much better life and at one point I was better than them so I say why did I end up like this.

I: Because of this you feel angry, betrayed and that makes you anxious

R: Yes

I: And the anxiety causes the epilepsy

R: Yes when I feel anxious the epilepsy starts

I: Now how have these symptoms of the epilepsy and heart problem affected your work and your social life the relationship you have with people?

R: It hasn’t affected the relationship I have with people. Sometimes I’m impatient and get angry very easily and people tell me that it is not good for my health and I shouldn’t be like that. My brothers, my sisters everybody tells me not to stress. I don’t have any problem with the society.

I: So it hasn’t affected your life?

R: It hasn’t but sometimes I fight with people

I: Do you fight a lot with people?

R: No I don’t

I: Do you participate on weddings, funerals, work in the society?

R: Yes

I: Your condition doesn’t hold you back?

R: No it doesn’t, I go to the programs but I stay for a short time

I: Why do you stay only for a short time?

R: Because I don’t drink alcohol like the others, I leave when the main program finish’s

I: aha so you don’t stay for long like other people

R: I don’t, I leave as soon as the program finish’s

I: Do people stigmatize you because of your condition? Do they tell you can’t participate?

R: No they don’t. They advise me.

I: They don’t stigmatize you instead they advise you?

R: Yes. When it comes to work we go around the rural and build houses. Sometimes people say he is not like he used to be he is sick, he can’t work and they let me go. The people I work with tell people that I’m not like I used to be because I can’t climb up the roof. It has affected my work. It’s not like it used to be.

I: What do you feel when things like this happen?

R: This is a problem, it is my condition that brought it on me so I don’t feel bad or angry. I just say thank God, I’ve worked when I wasn’t sick. I don’t feel upset when people say that because I’ve worked for many years.

I: Now from the symptoms you told me which one has affected you the most?

R: Losing consciousness. I lose my consciousness for a few minutes it could be in the house or while I’m working. I’ve also told Dawit about this. The medicine I was taking when I used to fall helped me a lot then I stopped taking it I thought I was cured then it started again. He said don’t stop taking the medicine that makes it worse. From that day on I take my medicine regularly I never forget. I take the medicine in the morning and in the evening.

I: When this problem started, what was the first thing you did?

R: My brothers took me to the hospital and there they told me I need to see a psychiatrist so I came here. They told me I will be okay as long as I follow up on the medicine.

I: aha so you didn’t go anywhere else? Tebel (water believed to have spiritual remedy) or traditional medicine?

R: No I didn’t. I have a sister in Addis Ababa she always tells me to go to tebel but why should I go to tebel I tell her this is not something spiritual it is related to mental health and the medicine is helping me I don’t fall anymore why would I go to tebel. My brothers also tell me to go to tebel but I refused.

I: Okay, what have you done about the other symptoms like headache and forgetting?

R: I just take this medicine and pray to God that he heals me with it. The other day Dawit said that they will write a referral for me to a different hospital if I don’t take the medicine properly. He said they could send me to Tekur Anbesa hospital in Addis Ababa.

I: aha okay so they’re saying the medicine has made a difference keep taking it?

R: Yes

I: aha Good, When you first came here who did you see?

R: Dawit

I: aha what questions did he ask you?

R: I knew him even before I got sick he used to rent my brother’s house. When I got sick he started treating me. He even came to my house to treat me. We used to be in the same compound in my brother’s house behind the health center. He is the one who told me to start following up I’m very thankful to him. He advises me to take the medicine properly.

I: Okay so you like the treatment here?

R: Yes

I: What did they ask you when you first came?

R: They ask me about the symptoms, how I feel and for how long I lose my consciousness. I don’t know how long I stay unconscious it could for ten to fifteen minutes or for an hour. He told me that I will get better if I keep taking the medicine. Now I don’t fall anymore I just lose my consciousness once a day around 9(local time).

I: How long do you stay unconscious?

R: He also asks me this question. He tells me to register the time. Yesterday when I was at work I lost my consciousness around 9 (local time) and stayed for five or ten minutes.

I: Do other health workers ask you about your personal life?

R: In other health centers?

I: No, health workers here like Dawit does he ask you about your personal life?

R: He asks me about my personal life. He says “don’t worry about the past don’t get angry you shouldn’t stress that is what gets you sick”. He is the only one who advises me I haven’t talked to anybody else. He gets very upset when I don’t take the medicine properly because he loves me.

I: aha do you forget to take your medicine?

R: Sometimes I get better when that happens I stop taking the medicine then it starts again. He tells me I shouldn’t do that. He gets very upset when I stop taking the medicine.

I: What do you think should be done in order to minimize the impact it has on your work and social life?

R: I should avoid stress and decrease the amount of work I do. That is what my brothers tell me. They say you have kids you should relax.

I: What should the society do?

R: people in the society love me. They tell me to follow up on my treatments and take care of myself. I have a good relationship with people in the society.

I: Is there any problem you faced to come to the health center?

R: It is very convenient for transport, it is very close. I come every month on my way to work to take the medicine. There is no problem.

I: Is there anything hinders you from using the medicine properly?

R: Now I’m taking the medicine regularly and I’m okay. But as I told you the epilepsy comes once a day or once in two days around 9 in the afternoon (local time), and in the evening when I sleep I feel something moving in my head and I get a head ache, my heart beat increases. I thought it was anemia at first because I’ve heard people say anemia has symptoms like this but the doctors told me it is not anemia. So I think it is the effect of the medicine.

I: aha the medicine?

R: Yes

I: Side effects?

R: Yes side effects

I: Have you talked to the health professional

R: Yes he told me to take fluids with the medicine but I can’t get fluids.

I: What kind of fluids

R: Fluids any fluid

I: You said you used to drink alcohols but you don’t any more. Why did you stop?

R: Alcohol doesn’t go with the medicine

I: Is that why you stopped?

R: Yes

I: Do you think it is related to the illness?

R: Yes if I take the medicine and drink alcohol it is related

I: How?

R: It makes me sick

I: In general what do you think should be done for people who have epilepsy so they live a good life?

R: Take the medicine properly, follow up with the doctor, obey the instruction of the doctor, avoid drinking alcohol, and eat food properly the epilepsy doesn’t make you lose your appetite. I have better appetite now.

I: From before?

R: Yes

I: How?

R: I have better appetite after I stopped drinking. I had gastric problem but now I’m better because I don’t drink alcohol anymore. Now the problem I have is with my head and heart.

I: What happened to your heart?

R: It starts with my heart and goes up to my head that is how I lose my consciousness

I: Okay is there something I didn’t ask that you want to tell me?

R: No Nothing else

I: Okay thank you very much
