# Supplementary material for: Experience and perceptions of mental ill-health in people with epilepsy in rural Ethiopia: A qualitative study
Source: PLoS One. 2024 Dec 13;19(12):e0310542. doi: 10.1371/journal.pone.0310542 (PMC11643256; doi:10.1371/journal.pone.0310542)
Supplement: S3 File — (ZIP) [file pone.0310542.s003.zip › data set/translation 0017D.docx]

**ID ( audio record) 0017**

**Translation 002**

Identification – WD, 32 years old female from the rural, daily labourer and house wife

I: I am Dr R and came from…….

I: ok you live in the rural, what is your job?

R: I have a small kind of bar… I sell ’Tella’…. People may not have a respect for women work (homemade cooking). The people from the rural don’t give you money easily. They gave me money and told me to invest it and help myself.

I: aha…homemade cooking (Yebaletena sera)

R: Yes, homemade cooking…. If there is any funeral or holiday in the neighbourhood, I will make small….Small money. When there is problem or funeral, we help

I: do you save money for future problems??

R: yes, small amount

I: small amount?

R: then I brew ‘Tella’ and sell it. I get 100 -150 birr small amount. Otherwise I don’t have any business.

I: aha, that is good and you also work at home ?

R: I work at home too. I clean the house, I do my job. Whether I the morning or at night I am the one who does the work.

I: that is good. How many children do you have?

R: three

I: three yes

R: one daughter and two boys

I: ok, how old is the youngest one?

R: the youngest….the youngest one is 8 years old. I did not give birth after that

I: you didn’t give birth after that?

R: Yes

I: where do they live now? Is it with you?

R: Currently, they live in A.A.

I: all the three kids?

R: all of them

I: why are they living in AA?

R: they said they don’t want to live in the rural?

I: they said that?

R: yes

I: with whom are they living with in AA?

R: Addis Ababa

I: are they with a relative?

R: they live in a rented house

I: aha

R; yes

I: do they work by their own?

R: yes they do

I: they work?

R: they don’t have a house but they rented a house.

I; they work in a rented house?

R: they sell something like soap….

I: so they take care of themselves?

R; yes in a rented house. They don’t have their own house

I; No house

R: yes only rented house

I: Ok are you with your husband?

R: yes

I: ok, very good, what then brings you to the health centre for the first time?

R: what?

I: what kind of problem brought you to the health centre?

R: the problem brought me there

I: what was the problem?

R: I got sick

I: Are you sick?

R: yes

I: how did you feel? What were the symptoms?

R:….. gurgenga words. The symptoms were at the time ….

I: tell me in Amaharic

R: I lost my consciousness, got dizzy

I: got dizzy?

R; my head was spinning

I: spinning?

R: I was dizzy, lost my head and fall down. I was baking Injera, I fall down on the injera after I baked one or two injera. I fall down it was gone kill me so he brought me to the hospital

I: so that brought you here

R: to the hospital

I: was there any other symptoms besides the falling down and dizziness?

R: I got dizzy for the second time

I: did it occur again?

R: after 15 days it occurs again on the fire for two days

I: did you fall on a fire?

R: Yes, I fall on the baking stove (Metad) and it got broke. It was worse and I came to Bue

I: did they say why you came here?

R: why I came here, it is the epilepsy

I: epilepsy?

R: I told him that it is epilepsy. He asked me what I feel, I told him I got irritable when I sleep with my husband

I: irritable?

R: yes when we have sexual intercourse

I; You get irritable during sexual intercourse?

R: yes, I have pain ….. It pierced me … oh intercourse (patient showed her abdomen with painful affect)

I: would you please get a little louder? Oh, do you scream when you have sexual intercourse? Do you feel pain in your abdomen?

R: yes I had pain in my abdomen, cramping, I lost my consciousness

I: you had pain?

R: Yes, when I have pain I lost my consciousness. I don’t know where it touched me whether it is my leg or my hand. I feel very irritable

I: so you have irritability?

R: yes, what shall I do with my life? I lost my consciousness. It made me change …what shall I do? shall I hang myself?

I: did you hate your life?

R: yes

I: do you feel very angry?

R: Yes

I: did you lose consciousness?

R: yes

I: what did you say about ending your life?

R: it is just to commit suicide

I: why?

R: it just that I feel very anxious …….(there is some guragegna)

I: do you argue a lot with him (her husband) or you just feel anxious?

R: yes, he nags me

I; do you feel anxious?

R; I just feel so anxious

I: ok what else do you feel?

R: I did not have my period (menstruation) for three or four months. Then I felt something on my abdomen then I came to Butajera hospital. Then I lost the baby

I: did you abort in Butajera hospital?

R: Yes I had an abortion…… Guragegna

I: what does that mean?

R: it is just on the fire

I: did you fell on the fire and your cloth got burned?

R: yes

I: did you feel angrier when you got burned?

R:, yes

I: you feel irritable you have abdomen pain?

R: yes

I: what else?

R: yes I have abdominal cramp and there is a fluid coming out of my genitalia

I: from your genitalia

R: Yes

I; is it blood?

R; yes and something which looks like “Aser”

I;”aser” means whitish colour?

R; yes

I: what else?

R; then when I had a headache…

I: do you loss consciousness when you have a headache?

R: I lose my consciousness, I don’t know anyone, I lost my consciousness for one and half hour and I have drooling of saliva

I; do you have drooling of saliva?

R: Yes drooling of saliva then blood

I: the blood from your tongue?

R: Yes I fell anxious first then…….. guragegna

I: does this mean that you don’t go out when you got sick?

R: Yes

I: you don’t work?

R: It doesn’t let me work

I: It doesn’t let you work?

R: It doesn’t let me talk, it doesn’t let me work

I: Okay what else were you feeling? Crying

R: I cried and cried then I just fall

I: Do you feel dizzy, sleepy?

R: When I get sick I feel like sleeping a lot, I fall down especially in the hours of coffee drinking

I: Do you feel tired?

R: Yes I feel very tired and off-balance like a drunk person

I: Aha you feel like a drunk person?

R: Yes like a drunk person, when I spend the day at the market and come back I feel like screaming

I: what is screaming?

R: I feel like screaming…screaming inside me

I: Do you feel like screaming?

R: Yes, Yes very much

I: Aha Do you feel like running away?

R: Yes, before I even finish my shopping and then I leave

I: To your house?

R: Yes, just to my house……. When there is much to do if I get good health I prayed for God will…….I used to be upset

I: Okay, Did you feel lonely?

R: Yes

I: this must be hard for you?

R: Yes

I: Do you still have all those feelings and problems?

R: Yes I do, if I sit down for a while I feel like going out

I: Do you feel this when you’re with people?

R: Yes……

I: Do you still have epilepsy?

R: Yes, it got better after I took the medicine

I: Is it better?

R: Yes

I: That is very good

R: Yes

I: Is there anything that hasn’t improved or that you still have?

R: The pain in my abdomen hasn’t improved, I still have pain

I: Do you have abdominal pain?

R: Yes

I: Still?

R: Yes

I: aha what else?

R: There is nothing else

I: You’ve told me lots of things now; you get angry, you are irritable, you have abdominal pain, you lose consciousness, is that right?

R: Yes

I: Are all these pains related?

I: I mean do all the pains have relations, is one connected to the other?

R: Yes

I: Okay, how?

R: After the pain in my abdomen gets better I have my menstruation

I: You have your menstruation after you get better?

R: Yes

I: Okay so is it the epilepsy that causes your menstruation?

R: I don’t know

I: How do you relate it?

R: I don’t know

I: But is it related?

R: I don’t know it may be related

I: Do you think it is related?

R: Yes

I: You know the reason for the relationship because they follow each other?

R: Yes

I: Is it?

R: Yes

I: Okay so now when you feel pain, you feel lonely, you can’t go to the market and shop whatever you want, right?

R: Yes

I: what are the other effects?

R: Nothing else

I: Now for instance you social life like going to weddings, funerals

R: Funeral

I: Yes

R: ……….it will pass me

I: You don’t go to burial ceremonies?

R: ……… I go out but it will pass me

I: Do they pass by you?

R: Yes

I: Other wise you don’t go to burials

R: I do go but I don’t sit down

I: You don’t sit down?

R: Yes

I: Okay so you do not do it as you wanted or like other people

R: Yes

I: What about work? Do you do work in the society?

R: Work when this problem started………

I: Okay, what does it mean?

R: I sit down and……

I: Do they tell you to see people off?

R: Yes

I: Otherwise you don’t work

R: Yes

I: Why don’t they let you work?

R: They don’t let me work because of my disease

I: Okay people are scared?

R: Yes

I: Aha so they tell you can’t work?

R: Yes

I: Is it the same for weddings?

R: Yes

I: You don’t work for weddings?

R: For weddings I make kitfo (traditional food)

I: You chop kitfo meat?

R: Yes

I: Okay

R: I do not work around fire

I: You don’t do work around fire?

R: Yes I don’t……..They tell me to sit down

I: Okay they tell you to sit down

R: Yes

I: Do you feel anxious around many people?

R: Yes when I feel anxious I go out stand outside for a while and go back

I: So you don’t have social interactions as much as you want

R: Yes

I: What about house work?

R: I don’t do a lot of house work. I do a few things. I bake injera sometimes.

I: a few things?

R: yes

I: Why couldn’t you do a lot?

R: My condition doesn’t let me

I: It doesn’t

R: Yes

I: When there is something to do in the community do people say don’t call her or do they stigmatize you?

R: No they don’t do that to me

I: They don’t?

R: Yes

I: Do they involve you in everything?

R: Yes

I: Some people say mental illness is contagious?

R: No they don’t say that

I: They don’t

R: Yes

I: Is everyone supportive

R: Yes

I: Your husband, your kids

R:everybody

I: All your neighbours

R: Even my neighbours

I: That’s good

R: Yes

I: How is it?

R: yes, when I get dizzy,…. immediately after that they put a scarf (Netela) in my head

I: Do they look after you?

R: Yes

I: They don’t abandon you?

R: yes, when I feel better… he puts a scarf on my head and he lets me sleep….when I lost my consciousness…

I: What did you say now?

R: They don’t pick me up immediately

I: Why?

R: I don’t know they fear that it is contagious

I: Do you think that’s why

R: yes, when I regain my consciousness….i feel very depressed….keeps quiet

I: When you regain your consciousness?

R: Yes

I: You see that they’re looking after you?

R: Yes, when I am aware……

I: Does it make you feel angry, anxious?

R: Yes

I: Has it (the seizure) happened to you recently?

R: Yes

I: When

R: This month

I: This month?

R: Twice

I: Twice this month?

R: Yes

I: Okay do you take the medicine properly?

R: Yes

I: You don’t forget?

R: No

I: Do you take it on time?

R: Yes

I: Then why do you have a seizure?

R: I don’t know

I: Why do you think it happens?

R: I don’t know. I have no idea

I: Okay so what do you do when you feel pain? Where did you go when you first got sick?

R: Hospital

I: Is it hospital?

R: This is where I came

R: Is it?

R: Yes

I: holy water?

R: I have been to holy water tebel

I: Is it before or after?

R: After

I: After?

R: First they gave me tablets and then they took me to Addis abeba

I: You went to Addis Ababa?

R: Addis Abeba ulos hospital

I: Paulos? (St. Paul hospital)

R: Yes Paulos and I got tablets for a month

I: They gave you medicine?

R: Yes they examined my abdomen

I: Did they examine your abdomen there?

R: Yes they told me I have a lump

I: They told you it’s a lump?

R: They told me it’s a lump

I: Then what did they do for you?

R: They didn’t do anything

I: Did they give you medicine?

R: No they told me to get surgery

I: They told you to get surgery?

R: Yes

I: And did you get surgery?

R: I refused

I: Why?

R: No

I: were you scared okay what else did you do?

R: I have been to three holy water places; Arsema holy water, Gabriel holy water and Abo holy water

I: Okay what additional treatment did you get?

R: When I went to Arsema tebel I stayed for 3 days and then I got my menstruation

I: Three days in Arsema

R: Yes and then I got my menstruation so I left

I: You left?

R: Yes then another holy water (Kidase’s tebel)

I: Did you drink it?

R: Yes

I: Is there something else you did?

R: Nothing else

I: You haven’t tried traditional medicine?

R: I went to the Wacho health centre close to me

I: you went to there?

R: I have

I: From all which one helped you the most?

R: From all?

I: Yes

R: there is nothing which helped me, only this one

I: what is this one?

R: It is only the tablet that helped me

I: The tablet?

R: Yes, when I feel tired, gets anxious or when I feel heavy …crazy on my heart…….

I: What happens when you take it? What do you feel in your heart?

R: It burns my heart

I: Okay it burns you?

R: Yes then it gets a little

R: A little what?

R: I feel a little better

I: Okay then do you feel better?

R: Yes I feel better

I: Okay when you first came to the health center how was the treatment?

R: The treatment?

I: Yes

R: The tablet wasn’t making a difference so I got injection

I: Did you get Injection?

R: Yes

I: Because they said the tablet wasn’t making a difference

R: Yes

I: Then what did you say?

R: Then they gave me tablet for the headache

I: They gave you

R: They gave me droplet for my eye

I: They gave you

R: Yes

I: Okay what else

R: Nothing else

I: What about your epilepsy? You came to this health centre for your epilepsy?

R: Yes I come here, I also use another health centre if they don’t have my tablets there they tell me

I: So they tell you to take your tablets from here?

R: Yes it was also closer for transportation

I: What is closer for transport?

R: Transport to come here

I: To come here

R: I have to walk to the other one

I: You have to walk to the other one?

R: Yes

I: Which one is better?

R: This one is better

I: This one is better

R: Yes

I: Okay why is it better? is the medicine available?

R: Yes here they give me in bulk, there it is little

I: What questions do they ask you when you come?

R: They ask me how I am feeling

I: Do you tell them?

R: Yes I tell them

I: Do you tell them what you told me now about your anxiety?

R: Yes

I: Properly?

R: I tell them properly

I: But do they first ask you about your private life?

R: They ask me what my illness is

I: They ask you what your illness is

R: I tell them I have anxiety, I lose consciousness. They ask me

I: What else do you tell them? that you feel like crying?

R: I just called

I: Okay you tell them

R: Yes

I: Do you tell them when they ask you or they don’t ask you

R: Even if they don’t ask I tell them everything

I: You tell them everything?

R: Yes

I: Okay what else? Do they ask you about the medicine?

R: They ask me what type of tablet it is

I: Do you tell them the type?

R: Yes

I: Do they ask you what type you take?

R: Yes it is a packed

I: Do you take the medicine correctly?

R: Yes

I: You don’t forget

R: Yes even if I forget my husband won’t

I: Okay that is good so what do you know about the medicine that is prescribed for you?

R: I don’t know anything

I: What do you know about the prescribed medicine?

R: What is it?

I: Do you know what type it is, the side effects it has? Do they tell you?

R: They don’t tell me anything

I: They don’t tell you? You don’t ask?

R: Yes

I: Okay what else, do they ask you about your personal life for example about your past relationships and so on?

R: Nothing

I: They don’t ask about your personal life?

R: They don’t ask

I: What would you feel if they asked?

R: I wouldn’t feel anything

I: You would tell them

R: Yes

I: To the health professionals?

R: Yes

I: Would you be offended if they ask for instance how your relationship with your friends or husband is?

R: No I wouldn’t be offended

I: You wouldn’t be offended?

R: I tell them even if they don’t ask me

I: Have they ever asked you?

R: Yes those who come home

I: Those who come home?

R: I tell those who come home

I: You tell them?

R: Yes they ask me about what makes me angry and what do I feel when I have intercourse with my husband

I: They say that?

R: Yes

I: Have these doctors asked you?

R: They’ve asked me

I: What did you feel when they asked you?

R: What is it

I: Did it annoy you? Do you tell them?

R: Yes

I: So how do you like the treatment here?

R: The treatment here?

I: Yes

R: After I got this treatment they visit me at home

I: They visit you?

R: Yes they come to my neighbourhood with a car…………..

I: What does it mean?

R: Now I go only when I feel sick

R: Is that what you tell them?

R: Yes

I: You don’t go even when you have an appointment?

R: I don’t have appointments

I: They don’t give you appointments?

R: Yes they don’t give me

I: What about the health centre close to you?

R: they don’t give me the medicine from that too. Only when it is finished

I: Do you go and take? Do they treat you?

R: Yes

I: But you don’t have appointments?

R: No I don’t come now I came here to you with an appointment

I: Here it is with appointments

R: There is nothing else

I: You only go when you finish your medicine?

R: Yes I go and bring tablets when I am left with two or four tablets

I: Do you follow up?

R: Yes

I: Do your neighbours tell you to follow up your treatment?

R: Yes

I: What do they say?

R: They say that

I: Do they support you?

R: Yes

I: Or are they against it?

R: They’re not against it they tell me to use it

I: They tell you to go use it?

R: Yes

I: But what about the illness that you told me which was harmful?

R: Yes

I: You have had seizure twice this month?

R: Yes

I: Right?

R: Yes

I: Okay so what do you think should be done for you to have a healthy and better life?

R: Die፣ dying

I: What does it mean?

R: I had enough of the world

I: Enough of the world?

R: Yes

I: You want to die?

R: Yes that is good

I: Why?

R: ………….I’m always sick and sick what can I do?

I: But what do you think should be done for you to live like other people

R:E… I can’t get what I want. Where can I get what I wanted.

I: What do you want?

R: I want to live my own independent life. It is not there and not in here. I don’t want anything else I just want to live my own life

I: My own life

R: Yes

I: How can you live your own life?

R: I want to support myself

I: By myself, how?

R: Me by my self……..

I: Okay you’re not independent now?

R: No not that much

I: Not that much?

R: Yes

I: Okay so my questions is what is missing for you to be independent?

R: What is missing is I live in a rural area

I: You live in a rural area?

R: Yes, I clean up the cowshed, I have to clean the house, fetch water………

I: Life is not easy?

R: Yes

I: Is it hard? Do you have a lot of work?

R: Yes too much work

I: Okay so even if life is expensive in the city. Is the money enough?

R: Yes

I: Is it enough?

R: Yes

I: Does it make you happy?

R: Yes it is okay. It is okay.

I: So why do you feel like it is better for you to die?

R: (Speaks in gurage)

I: What does that mean?

R: If it doesn’t get better in the rural

I: If it doesn’t get better in the rural?

R: Yes

I: Okay do you know why the illness is not getting better?

R: I don’t know

I: You don’t know?

R: I don’t know

I: What else do you think the society should do for your life to get better, for your condition to improve?

R: I don’t know

I: Nothing?

R: There is nothing

I: I mean for your life to get better

R: I don’t have a father, I don’t have a mother, I don’t have anybody next to me

I: Okay so it would have been good if there was someone who supports you

R: It would have been good

I: What else do you think the health centre should do?

R: what……….

I: So you get better

R: I don’t know

I: eeeee tell me what you know it is okay don’t worry

R: I think the city is better for me

I: It is better?

R: Life in the city makes me happy

I: aha better than life in the rural?

R: If I spend two days in the city everything is good

I: What is it about life in the city that makes you happy?

R: It is good

I: Is it good?

R: Yes it is good

I: Why don’t you like life in the rural?

R: I just don’t want it

I: You don’t want it?

R: I’m not healthy, My spirit is not in the rural. Nothing changes, the life doesn’t get better. But the life in here is good and better…..

I: everyone caring?

R: Nothing gets better, it is always the same, the same

I: Aha the life?

R: Yes

I: So does that mean you will have less burden? I don’t get it. What do you get in the city?

R: I don’t get anything

I: Then why do you want to live here?

R: Neatness

I: Neatness?

R: Yes

I: Okay what else

R: Its clean, there is water; water for drinking, water for washing that is it

I: You like that

R: Yes

I: Have you talked to your husband about this?

R: Yes

I: What did he say?

R: He doesn’t say anything. What can a person from the rural say…..

I: Is it? He just wants to live?

R: Yes

I: Okay thank you so much
