# Supplementary material for: Experience and perceptions of mental ill-health in people with epilepsy in rural Ethiopia: A qualitative study
Source: PLoS One. 2024 Dec 13;19(12):e0310542. doi: 10.1371/journal.pone.0310542 (PMC11643256; doi:10.1371/journal.pone.0310542)
Supplement: S3 File — (ZIP) [file pone.0310542.s003.zip › data set/Translation 0021 (1).docx]

I: My name is Dr.R. I’m going to interview you about epilepsy and other related problems what is your name?

R:

I: Tell me about yourself? How old are you?

R: Twenty

I: Okay twenty years old?

R: Yes

I: Are you a student?

R: Yes I’m a student

I: What grade are you in?

R: Nine

I: Do you work?

R: Yes

I: You only do house chores?

R: Yes

I: Where do you live?

R: Gule

I: aha where is Gule?

R: Gule kebele

I: Is it in the city or in the country side?

R: It is in the country side

I: Okay who do you live with?

R: With my mother and father

I: You live with your mother and father?

R: yes

I: Are you married?

R: No I’m not

I: Okay very good. What was the first issue that brought you to the health center?

R: Illness

I: Okay what kind of illness? What happened to you? Where do you follow up now?

R: Here

I: Where does here mean?

R: Health center

I: Health center?

R: Yes

I: In Kela?

R: Yes Kela

I: Okay, when did you start following up?

R: ………0155

I: more than a year ago?

R: Yes

I: Why do follow up?

R: Illness

I: Illness?

R: Yes

I: What kind of illness? What is it called?

R: I feel dizzy

I: Epilepsy?

R: Yes

I: Okay how did you feel?

R: I used to feel dizzy

I: You used to feel dizzy?

R: Yes

I: Okay what else?

R: Nothing else

I: You don’t feel anything else?

R: Yes

I: You only feel dizzy? How?

R: I feel dizzy

I: Okay is there anything else?

R: No

I: Do you lose your consciousness?

R: No I don’t

I: Are you sure? Do you understand what I’m saying?

R: Yes

I: You get what I’m saying?

R: Yes

I: Okay now you’ve told me that you have epilepsy, right?

R: Yes

I: So is there something you feel? Symptoms of the epilepsy?

R: When I work I feel dizzy

I: You feel dizzy when you work?

R: Yes

I: Okay what else?

R: I feel dizzy, I hear students making noise

I: When students shout?

R: Students in the school

I: aha How?

R: When I hear students make noise I get disturbed and sick

I: Aha when there is too much noise?

R: Yes

I: Okay what else do you feel?

R: I feel sick

I: What does your family say?

R: They don’t say anything

I: They don’t say anything?

R: Yes

I: Okay what about headache do you have head ache?

R: No I don’t

I: You don’t?

R: I don’t

I: How is your sleep?

R: It is good

I: Do you sleep well?

R: Yes

I: Okay what about anxiety or feeling of fear?

R: I don’t have either

I: You don’t okay so the only symptom of the epilepsy is feeling dizzy?

R: Yes

I: There is nothing else you feel? What do you feel when students make noise?

R: I feel pain in my head

I: you feel pain in your head?

R: Yes

I: How? What kind of pain?

R: I went to school one day then got sick so I asked for permission and went back home

I: You asked for permission?

R: Yes

I: To your house?

R: Then I called home and my mother came to take me, she waited for me

I: Your mother?

R: Yes, she came with my uncle

I: Why did she come?

R: The boy told her to take me

I: Who is the boy?

R: The teacher

I: The teacher?

R: Yes, he called her and

I: Why did he tell her to take you?

R: I was falling down repeatedly

I: You were falling?

R: They got close to me and told me to go so I left with her

I: aha you left with her?

R: Yes

I: When did this happen?

R: Around Tir(January)

I: Around Tir?

R: Yes

I: Okay then when you got home what did you feel? What pain did you feel?

R: I felt heavy

I: You felt heavy?

R: Yes

I: Headache?

R: Yes

I: Okay, what else?

R: Nothing else

I: Nothing?

R: Yes

I: Do you vomit?

R: No I don’t

I: You don’t vomit Do you have stomach ache?

R: No I don’t

I: You don’t?

R: Yes

I: Do you have Fear, Anxiety?

R: No

I: Do you lose your consciousness when the epilepsy starts?

R: eeee

I: When you have the epilepsy do you lose your consciousness? Do you understand what losing consciousness means?

R: I don’t understand

I: You don’t understand?

R: Yes

I: To lose your consciousness means not knowing where you are. Do you understand? Have you ever felt that way?

R: Yes

I: Does that happen to you?

R: Yes

I: Okay, when you lose your consciousness do you bite your tongue?

R: No I don’t

I: You don’t?

R: Yes

I: Do you pee your pants?

R: No I don’t

I: You don’t aha what do you feel when you re-gain your consciousness?

R: I don’t feel anything

I: What do you feel?

R: I forgot

I: You forgot?

R: Yes

I: Is there any other pain you feel?

R: No

I: You don’t feel any other pain?

R: No I don’t

I: aha so what do you think is the cause of all the problems you told me about?

R: I don’t know

I: You don’t know?

R: I don’t

I: aha so what did you do after you had the epilepsy?

R: eeee

I: What did you do? After you got dizzy you’re uncle came to school and took you home then what did you do?

R: Then they told me to go

I: Where? Health center?

R: Yes, They didn’t say anything, they just gave me a medicine and told me to leave

I: They just gave you a medicine and told you to leave?

R: Yes

I: Okay then what happened

R: I wanted to get checked

I: You wanted to get checked?

R: Yes but they just call me, give me medicine and tell me to leave

I: aha they told you it will get better and gave you a medicine, they didn’t ask you anything?

R: They didn’t

I: What did the doctor ask you? How did he talk to you?

R: I wanted to get checked

I: Why?

R: I wanted to get checked

I: You wanted to get checked?

R: Yes

I: What did you want checked?

R:…………

I: aha eeee

R: I didn’t get checked, they told me to give urine sample and leave

I: What did they ask you?

R: I told them I was sick

I: When they asked you why you went, you told them you were sick

R: Yes

I: What else did they ask you?

R: They didn’t ask me anything

I: Didn’t they ask you how you felt, where you had pain?

R: No they didn’t

I: Do you come every month?

R: I come every month to take my medicine

I: Every month?

R: yes

I: What do they ask you when you come every month?

R: They tell me to take the medicine every month

I: Okay

R: They don’t ask me anything else

I: They don’t ask you how you’re doing, how you’re feeling

R: They don’t

I: They don’t ask you if you’re feeling better

R: They don’t

I: Do they ask you about class?

R: No

I: About your friends?

R: I don’t want friends

I: What about girl friends

R: I’ve never had friends

I: You don’t have girl friends?

R: No I don’t

I: Why?

R: I don’t have

I: With whom do you go to school?

R: With students

I: With students?

R: Yes

I: Those students are not your friends?

R: They are my class mates, I had other friends but

I: aha they went to a different school?

R: They went to the university

I: aha you didn’t make any new friends?

R: They don’t do anything for me only for them selves

I: aha why didn’t you go to the university with your friends?

R: I Failed

I: What grade did you fail?

R: Fourth grade

I: How many times did you fail?

R: Three times

I: Now you were supposed to be in grade 11 or 12?

R: Yes but I stopped

I: You stopped

R: Yes and this year I started form grade 9

I: Okay now how do you feel because your friends are not with you?

R: The students shout

I: These students?

R: The students shout

I: They shout?

R: They don’t behave

I: They don’t behave?

R: They call everybody

I: You don’t like them?

R: No I don’t, they call me for no reason

I: They make noises?

R: Yes they call me for no reason

I: aha what does that mean?

R: They call everybody they see, they don’t keep quiet, and they hit me

I: Why?

R: And they tell them not to hit me or call me

I: aha the teachers?

R: No the students, the teachers don’t say anything

I: The teachers don’t say anything?

R: Yes

I: They don’t teach?

R: After class ends around 10:30 local time teachers call me from behind

I: And then what? Aha they call you for no reason?

R: Yes

I: aha you don’t like that?

R: Yes, I don’t like that

I: Okay, Do you talk to your friends who went to the university?

R: No I don’t

I: You don’t?

R: Yes

I: Okay, when you come here don’t they ask you how you’re feeling about class and your friends?

R: Who?

I: The doctors

R: No they don’t

I: Don’t they ask you about your personal life, school, family?

R: No they don’t

I: How would you feel if they ask?

R: I would feel happy

I: You would feel happy?

R: Yes

I: If they asked, would you tell them properly?

R: Yes

I: How did you come here?

R: When I was passing by

I: I mean how did you decide to come to the health center? Didn’t you go to tsebel (water believed to have spiritual remedy) or somewhere else?

R: Tsebel

I: Yes

R: I tried tsebel but it got worse

I: How

R: After I go to tsebel I start having bad thoughts

I: Why?

R: Because of my sisters

I: Your sisters?

R: Yes

I: aha if you go to tsebel

R: Yes if I go I tell them that I’m doing it for them, then I stopped

I: You used to go?

R: Yes

I: Why did you stop?

R: I stopped, I tell them to go themselves

I: Your sisters?

R: Yes

I: You tell them to go? You don’t want to go?

R: I don’t want to

I: Why?

R: I used to go but then I stopped

I: Why did you stop?

R: I stopped because of them

I: Because of who?

R: My sisters

I: Your sisters?

R: Yes

I: what did they do?

R: Sometimes they tell me not to stay home

I: Don’t stay home?

R: Other times they tell me not to go out

I: They say don’t go out?

R: They don’t want me to go every day, they say why do you go every day

I: aha They tell you not to go everyday

R: Once a week on Sundays

I: They tell you to go?

R:

I: aha now you don’t want to go?

R: Even if I want to

I: What do they say now? Do they tell you to go to the health center or tsebel?

R: They tell me to stay home

I: They tell you to stay home? They don’t want you to go both?

R: Yes

I: Why?

R: They tell me not to go

I: They tell you not to go? What about to the health center?

R: ……………..I was sick and I came

I: Who? Your mom?

R: Yes, they know her

I: They said they know her?

R: Yes

I: They said they don’t know you?

R: Then I told them to give the medicine

I: aha is it your mom who brings you to the health center?

R: She takes the medicine

I: So she is the one who brings you to the health center and takes the medicine

R: No she comes alone

I: You don’t come with her?

R: I came twice

I: Only?

R: Yes to take the medicine

I: Why don’t you come?

R: I will bother her

I: Is it?

R: Yes

I: If you come with her will you ask her to bring it for you?

R: Yes

I: Okay but isn’t it better if you come yourself for check ups

R: I’ll be late

I: You’ll be late?

R: Yes, The only free time I have is lunch time

I: Aha so did the medicine make a difference?

R: Yes

I: What kind of difference?

R: …………it helped me a lot, I was taking the whole pack………2134……..then I got sick

I: what did you say? She took you to the hospital?

R: ……………..

I: aha it wasn’t good?

R: Yes, If I couldn’t make it today….

I: When did that happen? When did you get typhoid and typhus?

R: I had an appointment here I came and went to school, I got sick when I ate my breakfast then when they saw me they asked me if I was sick and told me to get up and wash and go to the hospital

I: What else? Is the epilepsy better?

R: Yes

I: That is good

R: Yes

I: What changed?

R: After the medicine I feel much better

I: Do you feel sick?

R: Sometimes

I: Sometimes?

R: Yes

I: How often is that? Once a month?

R: Yes

I: You get sick once a month?

R: Yes

I: But it is better than before?

R: Yes

I: Aha so is the health care good?

R: Yes eee yes

I: You find it good?

R: Yes; the tablet is very sour

I: Is the tablet sour?

R: Very much, I don’t like it

I: It is big and sour

R: Yes it is sour

I: Okay what other problem does the health care have?

R: Nothing else, the medicine is not the same

I: It is not the same

R: Yes

I: Which one is better?

R: The one I was taking at first is better

I: aha the one they gave you now is not good?

R: Yes it is not right

I: Why? What side effects does this one have?

R: It hurts me

I: How does it hurt you?

R: It hurts me when I swallow and is very sour. It is very difficult

I: aha it is sour, is that the only problem?

R: ………….2354

I: aha what does it do?

R: It disturbs me

I: It disturbs you?

R: Yes

I: What could that mean?

R: …………2409

I: aha you said it in guragegna (gurage language) what does it mean?

R: ……………..

I: Your mom always tells you to swallow the medicine

R: Yes

I: Don’t you always take the medicine?

R: I do

I: Do you want to stop taking the medicine? Why do you ask your mother to take you to tsebel( water believed to have spiritual remedy)

R: It is going to choke me….2504

I: You thought you would get better

R: Yes

I: Then did you go?

R: They told me it doesn’t affect the medicine

I: They did

R: I go to tsebel in the morning……………..2529

I: aha because you weren’t getting better

R: Yes

I: Who arranged this for?

R: The doctors

I: aha the ones that are in buue

R: Yes and they wrote me a paper

I: aha have you ever went to buue to get checked?

R: Yes

I: Did you get checked?

R: Yes

I: Did the doctors see you

R: Yes

I: What about the health service there? How is it?

R: It is good

I: It is good

R: Yes

I: Which one is better here or there?

R: This one is better

I: This one is better?

R: Yes

I: aha Why?

R: They told me this one is closer

I: It is far aha is it only the distance? What about the health service?

R: The health service is good

I: It is good?

R: Yes

I: do you like the health service there?

R: Where?

I: There, did they check you properly like you want?

R: Yes, I went to the tsebel and……2728

I: To who? You?

R: The doctors

I: The doctors told her to take you?

R: ……….2736

I: Then did she take you?

R: Yes

I: To buue?

R: Yes

I: Then what happened

R: Then they arranged 2751…………

I: What did you want? Did you want to stop the medicine and go to tsebel?

R: I wanted to go tsebel

I: Then the doctors told you it doesn’t affect the medicine

R: Yes

I: So they told you to take your medicine and drink the tsebel

R: …….2827

I: aha so why did you choose the tsebel

R: To get better

I: aha you wanted to get better

R: Yes

I: Has the epilepsy caused a problem in your life? Why did you come to the health center? Is it because you were sick?

R: I couldn’t live

I: Is it?

R: Yes

I: What else, Are there any other problems the epilepsy or the symptoms have brought to your life?

R: The students make noise

I: Does it make you anxious when they make noises?

R: When I take the teacher 2939……….

I: aha the students yell at you

R: ………..3008

I: aha we don’t know who told them

R: ………..3020

I: They ask you?

R: Yes, but I don’t know them

I: You don’t know them?

R: I don’t know them

I: Is everything okay with your family, your sisters

R: Everything is good

I: How is your relationship?

R: everything is good

I: Do you do house chores when you get back from school?

R: ……….3059

I: Your sisters?

R: Yes

I: Turn by turn

R: Yes they are the ones who work. They told me I can bake injera and clean the house then left

I: Who said that?

R: The doctors

I: The doctors said you can bake injera?

R: …………..3140

I: You do chores?

R: Yes

I: What kind of chores?

R: Injera

I: You bake injera

R: Yes

I: What else

R: I don’t do anything else

I: What about wete (Traditional stew)

R: I don’t make wete

I: aha do you have cows

R: Yes, there are cows

I: Do you look after the cows

R: There are kids for that

I: Why don’t you do that?

R: What?

I: Look after the cows

R: There are boys

I: There are boys?

R: Yes

I: Your brothers?

R: Yes

I: Is it a job for boys?

R: His job is looking after cows…………..3228

I: aha does he eat

R: Yes

I: Okay, Is there a job your sisters do but you don’t?

R: No

I: okay there isn’t? You do all chores? Are there other things that your sisters do and you don’t, like playing?

R: No

I: You don’t go out, why?

R: ………..3251

I: After you finish working don’t you go out with your friends?

R: I don’t

I: Why?

R: I don’t go out

I: Why? What about your sisters do they go out?

R: Yes they go out sometimes

I: Why don’t you go out?

R: ………..3316

I: What about you?

R: I don’t go

I: Don’t you talk to your friends over the phone?

R: I don’t

I: Why?

R: I don’t call them

I: Don’t you want friends?

R: No

I: Don’t you feel lonely?

R: I don’t

I: You don’t?

R: No I don’t

I: Okay so what do you do after school and house chores? How do you enjoy? What do you like to do?

R: I don’t like anything

I: You don’t like anything?

R: Yes

I: I meant what do you like to do?

R: I don’t understand

I: You don’t understand?

R: No I don’t

I: In your free time what do you do to have fun?

R: I work

I: Work, now if there is a wedding in your neighborhood would you go?

R: Yes

I: You go to weddings, what about funerals?

R: I go to funerals too

I: You do?

R: Yes, both family and friend

I: You go to both family and friends?

R: Yes

I: aha what if they’re distant

R: I don’t go

I: You only go if they’re close

R: Yes

I: So now is it getting better? Is there a pain you still have?

R: I’m getting better

I: There isn’t any pain you still have?

R: Yes

I: Is there something you want to improve?

R: There isn’t

I: There isn’t

R: Yes

I: Everything is good?

R: Yes

I: How are you doing in school?

R: Fine

I: Fine?

R: It is good

I: Is your result good?

R: It is low

I: It is low?

R: Yes

I: aha do you want to improve

R: Yes

I: do you study?

R: I can’t study

I: You can’t study?

R: Yes

I: why?

R: When I open my exercise book my eyes don’t see

I: When you open your exercise book?

R: Yes

I: Then how do you write 3550……….how do you write

R: I can write but I don’t study

I: You don’t study?

R: I don’t

I: What will happen if you study?

R: It will cause a problem, I feel dizzy

I: You feel dizzy?

R: Yes, if I start studying I feel dizzy

I: aha

R: It doesn’t like it when I study

I: aha is it?

R: Yes

I: How do you pass if you don’t study?

R: 3625………

I: If it is an easy question

R: I answer the easy questions and leave the hard ones

I: Is it?

R: Yes

I: The questions on the exam

R: Yes

I: So you listen when the teacher teaches and you pass

R: Yes

I: eeee Do you do your homework?

R: Yes I do

I: Where do you do it in school or at home?

R: At home

I: You do it at home?

R: Yes

I: aha it is reading that you don’t like?

R: Yes

I: For you to study isn’t it good if you have a friend what do you think?

R: I don’t want

I: You don’t want friends?

R: Yes

I: Not a boy a girl friend

R: 3223………….

I: Someone you can talk to when you’re stressed

R: I don’t want

I: If you’re stressed or in a problem who do you talk to?

R: My mother

I: Your mother, do you tell your mother everything?

R: Yes

I: aha so your mother is like your friend

R: She is my mother

I: she is your mother

R: She gave birth to me so

I: Yes, eee okay what do you think should be done for you to improve your study

R: I don’t know

I: You don’t know?

R: Yes

I: You want to read right you want to study?

R: No I don’t

I: Why?

R: I don’t want to study

I: Are you sure?

R: Yes

I: Didn’t you tell me you wanted to improve your education, get better

R: 3857……..

I: You didn’t say that….. When I asked you why you went to tsebel what did you say?

R: To stop the medicine

I: aha you don’t want to take the medicine

R: 3923……… my mother tells me to take the medicine

I: Your mother

R: Yes

I: What do you want?

R: To go tsebel

I: To get better?

R: Yes

I: Completely

R: Yes

I: aha that is what you want. What do you think the doctors, your neighbors, the society should do for you to get better and improve your education?

R: It is their business

I: Why?

R: I don’t care about people

I: about people

R: Yes

I: Do your neighbors, the people around you talk to you properly?

R: Yes

I: Do they take care of you?

R: They don’t

I: How? Why?

R: But they talk to me

I: They talk to you?

R: Yes, I talk to them when they talk to me

I: Okay what else? Do you say hello to them?

R: Yes

I: Do they say bad things to you because of the epilepsy?

R: No they don’t

I: They don’t?

R: Yes

I: Okay, Do they stigmatize you?

R: I don’t know

I: You don’t know?

R: Yes

I: I meant if there is something you want to be a part of do they refuse to let you participate because of the epilepsy?

R: eeee Yes

I: They do?

R: Yes

I: Okay for example what tell me?

R: 4153……

I: What for example?

R: They tell me to do something else

I: Who says that?

R: neighbors

I: What do your neighbors say?

R: They tell me whatever they want me to do

I: What do you feel when they say that?

R: I don’t feel anything

I: You don’t feel anything?

R: Yes

I: Don’t you feel sad?

R: No I don’t

I: You don’t feel sad at all?

R: I don’t

I: Don’t you feel disappointed or upset?

R: No I don’t

I: So you think they’re right

R: Yes

I: When there is something to give do they say “don’t give it to her give it to the others”

R: They don’t

I: They don’t? When they get benefits do they share with you?

R: No they don’t share

I: Why?

R: I don’t know

I: Why don’t you ask? Don’t you say this is not fair? why don’t you ask them to share with you?

R: I don’t say that

I: You don’t?

R: Yes

I: What I’m saying is, what do you think they should do so that you get equal benefits

R: Nothing

I: Nothing?

R: Yes

I: Okay what about the doctors, what do you think they should do to improve the health service so that you get better?

R: …………..4304

I: eeeee can you say that in Amharic

R: …………4309

I: eee the medicine? What does it mean?

R: Because I take all the medicines through my mouth…….4315

I: eeeee you want them to be everywhere? What does it mean?

R: …….4331

I: Is there anything else I haven’t asked you that you want to tell me? Things that should be improved?

R: No

I: Nothing else?

R: Yes

I: Everything is good?

R: Yes

I: Thank you so much for coming here and being a part of the interview
