# Supplementary material for: Experience and perceptions of mental ill-health in people with epilepsy in rural Ethiopia: A qualitative study
Source: PLoS One. 2024 Dec 13;19(12):e0310542. doi: 10.1371/journal.pone.0310542 (PMC11643256; doi:10.1371/journal.pone.0310542)
Supplement: S3 File — (ZIP) [file pone.0310542.s003.zip › data set/Translation 004.docx]

**Translation 004**

I; we will ask question and please try to answer the questions freely

R; ok

I: let me ask about yourself what is your age?

R: me

I; yes

R; thirty

I: thirty ok. What is your occupation?

R; I don’t have permanent job. I just work what I get.

I: ok what is education level?

R: grade eight

I: up to grade eight?

R: yes

I: where do you live?

R: Sefere Selam

I: is it nearby in the city?

R; yes

I: your marital status?

R: I don’t have one

I: you are not married? Any kids?

R: no

I: ok let me start my question. Where was your medical follow up at the beginning?

R; here

I; when you come for the first time to the hospital what was the problem or what were your symptoms?

R: it is just something. I came here immediately after the first illness. I didn’t give it a lot of time. it was just one time. people told me that that it is good. It is my brother ….who first got the something….

I:you brother had also the same kind of illness?

R: yes he used to be sick then he started follow up here. there was someone called Bele who used to work here and he told me to start the follow up in here. Then they brought me the hospital so that I don’t …something. It was in the beginning of 2010 that I started the follow up and since then I have never discontinued the treatment thanks to God. Even if I plan to have a journey I took extra medicine like two tablets with me in case I spend the night.

I; what were the symptoms at the beginning?

R: it was just I fall down in a circular motion, I don’t see anything only something red. I thought it was initially the intoxication of Khat. I had poor sleep, we used to go some places, we used to dance the whole night. That did not work for me. It was after that it started.

I: is there any mental illness that is associated with the epilepsy symptoms that you told me like any feeling of anxiety or depression?

R: I worry that how can I live my life, since I don’t have anything that I own, how can I earn something. That is all my worry. Otherwise thanks to God I won’t discontinue the drug, that is my only worry.

I: it could be associated with the illness or it can also happen before the illness, was there any problems associated with substance use for example chewing Khat or drinking alcohol or smoking different kinds of stuff? Was there any kind of this problems?

R: e… I used to chew khat and drink alcohol. I don’t smoke. I only chew Khat and finish up and go. Then I stopped the drinking but khat is to speed up or to get over boredom.

I: do you still chew khat?

R: yes, in small amount.

I; in small amount?

R: then I went home early, after I took my medicine at night and I sleep

I: you take your medicine?

R: yes, after I ate my dinner

I: do you chew khat?

R: yes in small amount so that I don’t get bored otherwise it will shut your mouth

I: do you ever have a talk with the health professional about this issues?

R: yes

I; about the khat?

R: yes

I: ok what kind of advices did you get?

R; e.. you mean about the khat, even the alcohol I have stopped using it. Because of my health till that time

I: ok , any illness associated with the epilepsy

R: I don’t have any headaches. When I am on the sun for long time, when I work day time labour. Because of this I don’t work any heavy duty. I only work for someone close to me, when I deliver something they give me ten birr or something.

I: are they family?

R: yes, it is my family. I only have a mother. She sometimes sells coffee or something on the market. Otherwise she does not have any income. As I told you we don’t have any income. They pay for the medicine from that income or some other people pay for it every month. It is a must, every month I have to buy it even by taking the money from other people. You have to buy it with the money.

I:you buy it?

R: yes

I; from all the symptoms which one is the most bothersome? Which ones are the one that you want to improve?

R: the symptom?

I; illness sign or symptom?

R; now days?

I: yes , something that needs to improved so that you will be in a better health

R: what I want to improve now is something…. Since I don’t have income .. something…I don’t have a health insurance so that I can take my medicine … can you help to get me a health insurance?

I; ok

R: regarding my income I don’t have any income

I: what about regarding the illness symptoms?

R: illness symptoms?

I; you don’t have one?

R; yes

I: e.. regarding the community what is the perspective of the society towards people with epilepsy? the neighbours , family

R:most of the time I had this experience. The amazing thing I should tell to people what I am taking. There is one guy they bring the medicine from Addis Ababa, he is still not cures, sometimes he has something. He is called (A). I might know him if you have come here before

I:I did

R: his kid .. they bring him the medicine from Addis Ababa. If we ever come across him, I would have brought him and show him

I: show the medicine

R; yes I will show

I: is there any stigma or discrimination in the environment, in the family, neighbours. Have you ever encountered any isolation when there is a seizure ?

R; never. By the way when someone has a seizure on the road people run away, they are afraid, they say it is contagious. By the way even if I see something like that…

I\; was there any thing that happened to you or was there any discrimination from the social life or from school or from getting employed or not getting hired because of your illness

R: as I told you since being daily labourer is harmful for me I don’t do it. I have to hustle in the town to earn my daily income. I don’t have any shop, or office work. I hustle and work. I have stopped the daily labourer work because I thought it is harmful for me

I : you don’t work?

R: I don’t work

I; so this illness has created some pain on your work, you would not work, you would not get hurt

R: so that I don’t get hurt

I: what else

R: so that I can improve

I: what about school?

R; there is nothing on the school

I: what about relations with other people, during festivity or some programs

R: I attend all that

I: ok, when you compare it to other healthy person, is there anything that you were not able to do? Do you feel that you can do anything like other people or is there anything that you think you should not do?

R: no there is nothing

I: when you compare yourself you think that you can do anything?

R: yes I can . it is just that I thought it is harmful for me otherwise I can do anything. There is nothing hold me back. It is just the health professional told me that I might get hurt in my head if i become daily labourer. Otherwise I can work

I: how do you choose this health facility?

R:I am comfortable here

I: how did you come here ? some people go to Addis Ababa?

R: it was one day that I fall down it was by accident. I took me with a Bajaj. Then I got treated, I was hurt here… then I was told to start the medicine. Then after my wound got healed I started to use the drug

I: how did you find the change of medical treatment

R: the something…is good

I: how was the treatment? What has helped you?

R: thus one?

I: yes

R: I have seen a lot of change. A lot a lot

I: do you have any associated mental illness like anxiety with the epilepsy? have you ever had someone talking to you or something that you see which other people don’t see? In addition associated with the illness have you ever had feeling bored or depressed, not doing something that you wanted to do was there any of these symptoms?

R: like that

I: is there?

R: no there isn’t.

I:for the epilepsy or the headache what have you done to improve your life other than the medical treatment?

R: E… There is nothing I did because I don’t have anything

I : was there any changes in your life from the things that you used to do?

R: I have decreased to use substances. I even discontinued to drink Alcohol. I don’t even smoke cigarette. As I told you before I use Khat in small amounts to get over my boredom

I: do you still chew khat?

R; yes

I; ok let me ask you about the hospital or the health center how did you find it?

R; the hospital is good

I; what do you discus with the health professionals? Do they talk discus with you well?

R: yes they do well

I: what kind of questions did they ask you when you come for the first time?

R; they asked me whether I am taking the medicine properly? “Do your health get improved? How is your substance use?”

I: they say that?

R; yes

I; do they ask you when you come for follow up or when you come for the first time?

R: around the beginning

I: yes do you remember what they asked you when you come for the first time with Bajaj?

R: I did not start that day. It was after that

I; ok what did they ask when you come for that day?

R: when I come that day

I: yes before they start you with medicine?

R; don’t do that, don’t do this. Take your medicine properly, have a proper monthly follow up

I: do they ask you about your daily life, the problems of life when you come here?

R: daily life , yes they do.

I: what do they ask/

R; for example, what kind of work do I do? I used to deliver Khat by cycle and it was very sunny and he told me to stop that job. And I stopped

I: did you ever forget or discontinued to take your medicine?

R; never

I; what do you think is the reason that you have to take your medicine without discontinuation.

R; for my health

I; please explain

R: to get out of my health problem

I: ok , what else?

R: it means that so that I can get better, so that I can reach to what other people achieve

I: what do you understand about the medicine? Do you take it without questioning or do they give you an explanation?

R; yes they tell me to take the medicine properly.

I: do they tell you about the side effect of the drugs… etc?

R: yes they ask me to eat well.

I: do they say that?

R: yes

I; ok, some people don’t like to be asked about their personal life for example about their way of life or their income or something that is worrying them. So some people might like to be asked or they might not talk these kind of stuffs if they were not asked or some people talk even if they are not asked. People have different kind of behaviours. Therefore , the health professional from this health centere did they ask you about your personal life in depth

R: yes they asked me.

I: how did they ask you please tell me

R; something like how is your work? Did you look for work? Something like that

I: what about your relationship with your family

R: the relationship with my family they ask me that too. I am living with my mother since I don’t have any income

I: what do you feel that you are asked this?

R: it is very good. It is only when you talk with other people that you get solution. If you hold it to yourself…

I:did you have obstacles of getting to the hospital?

R: what?

I:for example getting stressed or very busy with work etc… may make you not to come to the hospital. Something like the distance from here….

R: no there is nothing much important than my health. Whatever work it is I will discontinue it and will come

I: what do your family or the people around you comment about your medical treatment?

R: yes my mother does not want me to discontinue the medicine. She asks me whether my follow up day has reached. On my follow up date even if I don’t \have money I will take it from my mother for the medicine.

I: e.. do they tell you about your improvement? Do they say that you have improved

R: yes very much

I: like..

R: yes my mother will be happy. She gives thanks to God. She becomes happy that her child has become like this. She becomes very happy

I: ok what about people other than your mother

R: since there is nothing seen out of the ordinary…other people like neighbours etc don’t know about it

I; ok, you know about the disease. So what do you suggest should be done for people living with this condition to improve their quality of life, not only their health but also their general health quality of life, their work and their relation with other people?

R; I will tell him what I saw and what I got and I think he will get better after he has used it.

I: what should the health professional do? What is expected from the health professionals?

R: follow up , follow up

I: what kind of follow up is that?

R: it is medical follow up. Follow up for his health, asking him about how, are there any improvements or is it getting worse. if it is getting worse , changing the medicine.

I: what is expected from the hospital? what should be facilitated?

R: for example for those who have poor income to facilitate the drug intake… as I told you before.

I: what is expected from the society for those people who have epilepsy?

R: yes

I: for a better quality of life?

R: to create easy jobs for those people who can work in association with the people and taking them to the hospital

I; what about the government?

R: if the government knows, it is good to get a follow up

I: what kind of follow up?

R; it is to take and use the medicine properly. To have a better level of care

I: to improve the care

R: yes

I: ok is there anything that you want to tell me, things that I did not ask but something that you want to tell me other than this treatment

R: what I am telling you is since my level work is as I told you and if you can assist me to take the medicine without ….

I: to get it for free?

R: yes, to show them a paper. And to get something and pay for it …. Is with in two or one year time.

I: you mean insurance?

R: yes since I don’t have any to pay for that

I: ok what else?

R: there is nothing

I:ok, thank you. Thank you for your time. I have finished my questions.
