# Supplementary material for: Experience and perceptions of mental ill-health in people with epilepsy in rural Ethiopia: A qualitative study
Source: PLoS One. 2024 Dec 13;19(12):e0310542. doi: 10.1371/journal.pone.0310542 (PMC11643256; doi:10.1371/journal.pone.0310542)
Supplement: S3 File — (ZIP) [file pone.0310542.s003.zip › data set/translation 01.docx]

**Code 130615-001**

**I:** okay, I am very thankful for your willingness to come and do the interview. As I told you I came from Addis Ababa University and I am here to ask you about your illness and what you went through because of it.

**Responder:** I’ll tell you about my illness, right?

**I:** I am here to ask

**Responder:** yes

**I:** okay, there is a disease that you are treating here, right?

**Responder:** yes

**I:** tell me about it. How do you come to the health center?

**Responder:** I always come to the health center. I come here monthly. I come here whenever I finish it. It was a long time ago when I got this disease. It’s been about 20 years since I’ve got this disease. I take care of myself. I’ll never stop this medicine. I don’t drink that much of alcohol. I drink a little. The disease will decrease that way. But strength, strength; I don’t have strength. it can make me sick if I run out of pills. But if I take other pills I’ll be free from the disease. I don’t have any. i took the pills on a daily basis. I have nothing. I just take the pills and if I finish; I’ll come and take from here. I came here by myself. No one would come here to bring it to me. So it’s because of the pill that I’ve been on so far. If it wasn’t for it, I wouldn’t be here. I’ll fall and faint and end up broken. I wouldn’t have come this far.

**I:** nothing

**Responder:** my body would be broken, my body would be broken and I would stay home. So if I take the pill day to day the disease will improve. It wouldn’t make me faint. And most of it has strength and it’ll boost your strength but I don’t feel like it. I’ll go anywhere on my own. I took my pills with me whenever I go to the city. I have finished the pills. And every time I finish my pills I come here to take. I took my pills where ever I go whether I went to Addis Ababa or anywhere else. I bring my own medicine with me. I won’t buy there. I took it from here. I am very fine till now. Thanks to God.

**I:** okay, you said that you first came here about 20 years ago, right?

**Responder:** yes

**I:** you got it 20 years ago, about 20 years ago

**Responder:** yes

**I:** do you remember how it was the first time?

**Responder:** yes. I used to faint a lot. I don’t even know it until I wake up. What if I fell into the fire? Well, I don’t even know it. If I fall into the water, I might die in the water. But after I started taking the pills I haven’t faint. Well, thanks to God, I am here because of the pill.

**I:** yes

**Responder:** I would be dead by now; I am here because of the pill.

**I:** let’s talk about the time that it first started, when it first started

**Responder:** The first time

**I:** what kind of symptoms did you have?

**Responder:** nothing, I just faint

**I:** it makes you faint

**Responder:** I bite my tongue, I am just sick

**I:** what else?

**Responder:** my lips darken and my body is weak.

**I:** you used to faint

**Responder:** yes, I don’t know it

**I:** you didn’t know

**Responder:** yes

**I:** for a long time, for a long time

**Responder:** no, it doesn’t last long

**I:** it doesn’t last for long?

**Responder:** it doesn’t. I went to Addis Ababa but they didn’t accept me either.

**I:** no, do you stay unconscious for a long time?

**Responder:** no I just took the pill at that time.

**I:** yes, before you took it

**Responder:** yes, I took the pill

**I:** when you get the seizure

**Responder:** yes

**I:** does it make you faint?

**Responder:** yes, I don’t know it

**I:** you don’t know it?

**Responder:** I don’t it makes me faint, but I don’t know anything when I faint. I don’t know anyone, I don’t know where I fell; I just know when I wake up. It’ll stay for long, like an hour or more than an hour. It makes me faint.

**I:** it makes you faint

**Responder:** yes, I am okay now. I haven’t stayed for long till I started taking the pill. I don’t know, I have waited for about four months. I even went to Addis Ababa but I didn’t find it there, I didn’t find it here also. Then I went to butajira and I found it in butajita.

**I:** Did you find it?

**Responder**: I found it butajira. They told me to bring someone who can tell them that I had a seizure and how it makes me. And I took him and he told them

**I:** what did he say?

**Responder:** neighbor, neighbor

**I:** what did he say to them?

**Responder:** “he has a seizure, it makes him faint. It makes him urinate. He’s been through such problems”

**I:** what does it mean when you say it makes you urinate?

**Responder:** it makes me urinate

**I:** it makes you urinate

**Responder**: it makes you urinate; it has a lot of problems

**I:** what about foam

**Responder:** yes, it has foam. It makes me urinate. Everything is miserable. Well, it’s a very serious disease. But after I took the pill, I am living peacefully. I also farm. Yes, I manage my own home.

**I:** that’s good

**Responder:** so if I hadn’t taken this pill on a regular basis, I wouldn’t even have been able to stay here for a couple of years.

**I:** what is it called? What is your illness called in your environment?

**Responder:** what is the disease called?

**I:** it’s known, right?

**Responder:** yes, what was it called? Stress, I don’t know.

**I:** Don’t you remember it?

**Responder:** I forgot

**I:** It’s okay

**Responder:** I forgot it

**I:** okay, what kind of health problem do you have besides the seizure?

**Responder:** nothing

**I:** you don’t have any

**Responder:** yes

**I:** addiction, it can be something like alcohol addiction

**Responder:** no, I don’t have to worry about it.

**I:** you don’t have alcohol addiction

**Responder:** yes

**I:** do you drink alcohol

**Responder:** I drink a little. I don’t drink much. I drink a little. I drink as much as I can and go.

**I:** do you drink on holidays or do you drink daily

**Responder:** I go and drink with my friend

**I:** what if you don’t drink? Aren’t you going to get depressed if you don’t drink? Like if you don’t drink today

**Responder:** it’s boring

**I:** aren’t going to be disappointed?

**Responder:** it’s our habit so we’ll get bored, because of our habit

**I:** some

**Responder:** a little bit, a little bit. Yes, we come to the city here. We are in the countryside, the city is nearby. We are close so we come here to drink.

**I:** where is it? I forgot to ask you where your kebele is.

**Responder:** around Bui

**I:** it’s around Bui

**Responder:** it’s around Bui. Well, kebele but it’s going to take it. It’s going to take our land. It’s about to take it all in. it’s said to be around Bui.

**I:** around Bui

**Responder:** yes, around our kebele

**I:** yes, and the problem with your drinking, is it because you get depressed if you don’t drink?

**Responder:** it would be better if I don’t drink

**I:** do you drink for fun or?

**Responder:** it’s just for fun, to spend the time. it’s for fun but it would be better if I don’t drink. I’d rather drink water and live.

**I:** how much do you drink once? How much do you drink?

**Responder:** I drink around three glasses of tela and other thing too.

**I:** what about vodka

**Responder:** vodka is hard for me

**I:** you can’t drink vodka

**Responder:** no, I can’t

**I:** do you drink tela?

**Responder:** we drink tela, if we get draft beer, we drink draft. And most of the time we drink draft.

**I:** do you drink daily?

**Responder:** no, we don’t get it every week

**I:** weekly?

**Responder:** we don’t get it daily. We don’t even get it within a week. If relatives come or if a friend comes or if it is sold for free. But we don’t buy it from our pocket. How can we manage our house if we finish it all by drinking? We do farming just to buy us food. It doesn’t have enough profit.

**I:** some people becomes addicted and there’s something like that. I just wanted to ask if there’s such kind of thing

**Responder:** yes, dome, some of them can’t spend a day without drinking. There are some who take it home. They take it home to drink it at night. Yes, but we will immediately drink and cross over.

**I:** it doesn’t matter if you don’t drink.

**Responder:** it doesn’t matter if we don’t drink, we don’t care. We drink on market days. There is a market twice a week. Well, we drink much on that day like yesterday was Thursday, right?

**I:** yes it was Thursday

**Responder:** Tuesday, we came here on these days okay

**I:** it’s like that

**Responder:** yes

**I:** what about stress, depression. Do you have a feeling of depression or something like that?

**Responder:** I don’t get depressed

**I:** you don’t

**Responder:** I’ll never be depressed. I’ll have fun. I’ll have fun with everyone. Everyone looks for me to have fun with them. But I don’t get stressed.

**I:** you don’t get depressed

**Responder:** I don’t get stressed

**I:** stress?

**Responder:** I don’t get depressed, I don’t

**I:** hating noise, wanting to be alone, difficulties in falling asleep; have you ever such kind of things. Do you hate noises? Something like hating noise when your children are talking

**Responder:** no, nothing. Our children are not with us. They are in Addis Ababa.

**I:** they are in Addis Ababa

**Responder:** they live in Addis Ababa. They are in Addis Ababa but there are one or two here. There are two and they’re very little. But the rest are in Addis Ababa. They’re in Addis Ababa.

**I:** what about insomnia? Do you have a problem with having difficulty in falling asleep?

**Responder:** on day time? In God’s name!

**I:** not during the day but at night.

**Responder:** I sleep at night.

**I:** do you sleep?

**Responder:** yes

**I:** so you don’t have stress, mental stress.

**Responder:** absolutely not.

**I:** you don’t have it

**Responder:** after I took this pill

**I:**no, is it the same before you took the pill

**Responder:** I don’t have that much stress before this.

**I:** the medicine that you are taking, is it just for the seizure

**Responder:** just for the seizure

**I:** is it just that?

**Responder:** I took only that.

**I:** don’t you have any other stress

**Responder:** no, I don’t know any.

**I:** okay, okay you told me your symptoms, you told me your symptoms. And where did you first got the treatment

**Responder:**butajira clinic.

**I:** first butajita

**Responder:** yes

**I:** 20 years ago, when you got sick.

**Responder:** yes, I went to Addis Ababa. Addis Ababa, what was it called?

**I:**Amanuel

**Responder**: I went to Amanuel. They told me that I am fine and that I don’t have anything. My wife went with me but they didn’t ask her. They said they wouldn’t accept it

**I:** did they say that?

**Responder:** yes

**I:** they wanted you to go to butajira

**Responder:** they said they keep only those who are mentally ill. But they don’t accept people who can speak well. Then I went to butajira. After that I started coming here. I started coming here soon.

**I:** yes, it’s not that far.

**Responder:** Yes, I got it most. But before that we used to go to Butajira and brought it.

**I:** yes, so it means you first went to butajira

**Responder:**yes

**I:** before that, when you get the seizure, who took you to butajira? Who told you there’s a treatment there?

**Responder:**my wife

**I:** your wife?

**Responder:**yes, I mean I knew it. I knew the hospital. I used to go to that hospital.

**I:** yes, I mean at first

**Responder:**we go, we always go there. Sometimes we went to the market to find it.

**I:** no, I didn’t mean who took you to butajira, I mean you’ll go to the hospital if it’s considered as a disease, right?

**Responder:**yes

**I:** who considered it as a disease and decided for you to go to the hospital

**Responder:**my wife

**I:** your wife?

**Responder:**my wife.

**I:** did you go as soon as you get the seizure or did you go somewhere else

**Responder:**we didn’t think it’s serious so we stayed for a while

**I:** for a while?

**Responder:**it comes; it comes back again after sometime. It comes after a month. Yes, it comes about a month later. It stayed for about 3 or 4 months before coming back again.

**I:** after you went there, haven’t you tried to go to tsebel or traditional healer or something like that?

**Responder:**I didn’t go

**I:** you haven’t tried

**Responder:**I didn’t go

**I:** did you go to the hospital only

**Responder:**I come only to the hospital. I never went to tsebel; not even once.

**I:** why did you choose to go there? Do you remember the reason?

**Responder:**I didn’t think tsebel will heal me but it would’ve been great if we went there. It might heal me if I try it.

**I:** did you really think that you would be healed there? In butajira?

**Responder:**butajira, butajira is a hospital

**I:** is that why you went there?

**Responder:**yes

**I:** did you know someone who was being treated? Did you wife know someone who was being treated? Did she know someone who went to butajira to get the treatment?

**Responder:**no one was getting treated. I was the only one who went there

**I:** it’s just you

**Responder:**yes

**I:** neighbor. From neighbors

**Responder:**we find the pills from butajira, mostly.

**I:** yes, I mean if there is someone from your neighbors who was getting the treatment

**Responder:**around our community

**I:** around your community

**Responder:**there’s no one

**I:** no one?

**Responder:**yes

**I:** so you just said that you should go to butajira, casually?

**Responder:**yes

**I:** okay. Okay great. You told me earlier that you get some benefits from going there, that the seizure has reduced and that you are doing your job.

**Responder:**yes

**I:**do you get the seizure these days?

**Responder:**no, I don’t.

**I:** don’t you?

**Responder:**no

**I:** how long has it been since you had the seizure

**Responder:**it’s a long time ago. The pain has gone away at some point.

**I:** have you stopped taking the medication?

**Responder:**yes for about five or six years. I was okay. I was okay for about a year.

**I:** why did you stop?

**Responder:**the pain has gone.

**I:** it’s enough, I am okay?

**Responder:**after a long time if I get tired. I’ll get the seizure if I get tired. We built a terrace. You build a terrace in the countryside, right? Yes and when I was working on the terrace, I was working on it for about a year. I don’t know if it’s a year but I didn’t take it for a long time. And after working I fainted in my house.

**I:** were you taking the medications at that time?

**Responder:**I take it actually. I couldn’t stop it so I was taking it. But I don’t take the pill. They told me to take the little one. I was just taking that one.

**I:** I don’t understand, what do you mean by the little one?

**Responder:**they don’t bring the primary one. But the gram okay

**I:**oh you mean the gram, okay.

**Responder:**they told me to take it that way but I didn’t take it. I just wanted to take this

**I:** that means the big one which is 100 grams

**Responder:**100 grams, okay. I was taking it and I left it for a while. It’s been five or six or seven years but I fainted.

**I:** when you said “I left it”, it means you stopped taking it, right?

**Responder:**yes, I went to the terrace and I fainted then the doctors told me that I shouldn’t get tired.

**I:** you started again

**Responder:**yes until now

**I:** you are taking it now

**Responder:**I am taking it

**I:** are you taking the 100gram

**Responder:**no

**I:** hundred grams, the white one

**Responder:**yes, I took the hundred grams; I took it for one month.

**I:** for a month. You come here every month and take it.

**Responder:**yes for a month. They give us for two months also. But if they doesn’t have supply they’ll give us for a month or for 15 days sometimes until it’s brought to them. Yes, that’s because there are a lot of people that come here.

**I:** okay, when you come here, what did they ask? How is their treatment? You told me you got treated in butajira, right?

**Responder:**yes

**I:** after it was started bui, you told me you had been treated in bui too.

**Responder:**yes

**I:** when you go there, how do they greet you? What do they do?

**Responder:**here, yes hospital.

**I:** yes, in hospital

**Responder:**in hospital, they are great.

**I:** are they great

**Responder:**they are good, they are really good

**I:** do they ask you?

**Responder:**they ask me about what my disease is, if something happened.

**I:** always, every month you come.

**Responder:**they ask me if something happened, every month.

**I:** they ask me

**Responder:**yes

**I:** did you get a seizure or something like that?

**Responder:**yes, they ask me, “What happened to you?” “Nothing happened” but if something happened I’ll tell them. I have nothing to worry about. I am not afraid to tell them. But they give me the pill.

**I:** they give you after they ask you

**Responder:**yes

**I:** okay great. What did they ask you the first time you come here? And now when you come here for the follow up, do they ask you if there is a difference and if you are taking the medicine?

**Responder:**yes

**I:** what about the first time you come here?

**Responder:**I went to butajira. The record was in butajira. well, the record was not sent here.

**I:** it was not sent, okay.

**Responder:**they didn’t send it. Finally, the gram of the pill, I mean what’s it called the thing that lets you take the medicine.

**I:** the prescription.

**Responder:**I brought the prescription and they see it. They see the record.

**I:** without the referral paper

**Responder:**yes, something like that. They told me to go back there. It was far away plus it was expensive.

**I:** yes, it’s far.

**Responder:**yes this happened. They said they don’t know it and they told me to bring them the type that I was taking there and to bring them the paper. Then I said okay and I brought it to them. I brought them the paper without taking the medicine from there. Then they have a record here and they record it. But when I didn’t find it here I went to the other record.

**I:**butajira?

**Responder:**yes, I go there. There is a card so I take it out with the card. Then we got a lot here and butajira was closed.

**I:** when you first come here did they ask you well about your illness and how it started?

**Responder:**yes, they asked me after I come to butajira.

**I:** after you come to butajira

**Responder:**yes

**I:** you were asked in butajira, right?

**Responder:**I went to butajira. Well, after I got back from butajira they asked me. In fact, they called my wife and asked her. They didn’t ask me. They asked her how I was doing.

**I:** they asked her

**Responder:**yes

**I:** why didn’t they ask you?

**Responder:**because I don’t know how I fainted and how I was

**I:** you don’t know

**Responder:**they said” you know when you wake up but you don’t know how you were and how you’ll be when you are asleep” so they asked me to bring her and I called her and told her to come.

**I:** did they start after she came and explained?

**Responder:**she come and explained

**I:** did you start your treatment after that?

**Responder:**yes

**I:** okay about the medication, you told me earlier that you take the hundred gram, right?

**Responder:**yes, yes

**I:** what do you know about the medicine?

**Responder:**other thing?

**I:** what were you told about the medicine?

**Responder:**about the medicine, just this. It’s just this.

**I:** what did they say about the medicine, if the doctor told you about it?

**Responder:**they said it might go away so they told me to take the smallest gram then I told them that I was not going to take it. They said “what if it goes away”, but I don’t want to. I wanted to take that one so I am still taking the first one.

**I:** yes, just to reduce the gram

**Responder:**yes

**I:** but they didn’t tell you to stop it, right?

**Responder:**yes, it’s 70 gram and there is 30 gram. He said it should be 60 gram.

**I:** did he say that?

**Responder:**yes, they told me to take this one but I said no. I told them that I was not going to take it.

**I:** okay, have they ever told you about the side effects of the medicine?

**Responder:**I didn’t have any problem.

**I:** what happened to you because of the medicine?

**Responder:**nothing happened

**I:** the medicine didn’t cause any harm

**Responder:**no, it didn’t

**I:** what about them, did they say something like” if this, this things happen it might be the medications side effect so you don’t have to worry.” Have they ever said that to you?

**Responder:**I wasn’t told.

**I:** you weren’t told, okay. What about when you should take it?

**Responder:**I take it at night.

**I:** did they tell you that here?

**Responder:**yes, it shouldn’t be quitted.

**I:** that it shouldn’t be stopped.

**Responder:**yes

**I:** do they tell you that always or do you take it because you believe so.

**Responder:**yes, I, myself.

**I:** but they didn’t tell you that.When you come to the health center, when you come to the hospital what advice do they give you, not to stop taking the drug.

**Responder:**they say,” if you stop taking the medicine you’ll get hurt. It can’t go away so you should take the medicine regularly.” They even wrote the type of the medicine in my card in case I go somewhere so that if finish it; I could buy it somewhere else. There wrote it in my card.

**I:**matema

**Responder:**we brought it out then it was written on our card. Well, then that one was lost.

**I:** okay, I mean if there’s anything that you were told about the medicine. They advise you not to stop the medicine because you might get hurt. What else did they advise you?

**Responder:**they didn’t advise me anything else

**I:** if there is any advice that they give you.

**Responder:**we just take it. They ask us if something happened but they didn’t say anything else, we just take it.

**I:** they didn’t tell you anything

**Responder:**they don’t.

**I:** nothing, for example: did they say something like “don’t drink alcohol”

**Responder:**they told me not to drink

**I:** did they say that?

**Responder:**yes, they told me that alcohol will make it worse.

**I:** it will make it worse, don’t drink.

**Responder:**yes

**I:** what did they tell you about sleep?

**Responder:**they didn’t tell me about sleep; I sleep at night and nothing happened

**I:** what about fatigue?

**Responder:**fatigue, I got tired when I work

**I:** yes, is there anything that they say about fatigue?

**Responder:**nothing

**I:** don’t get tired by doing a lot of work.

**Responder:**yea but we work in order to get food. What should we do? If we don’t work where can we get food from? Our job is farming. We work in agriculture. We don’t have a business but it would be great if we have a business. Well, we don’t have other job so we farm and it’s tiring. It’s very tiring. Farming is very tiring.so who can leave, who can spend a day without eating food.

**I:** yes, have you ever forgotten to take your medicine? You are taking. It’s been a long time since you started taking the medicine, right?

**Responder:**I am taking

**I:** you are taking

**Responder:**yes

**I:** nothing. It’s just that you might forget sometimes.

**Responder:**oh please, I might forget to take it at night. Well, I put it in my coat, the one that I’ll wear so I’ll take it on my way.

**I:**but have you ever forgotten to take it at night?

**Responder:**sometimes I forget

**I:** what about home? Doesn’t your wife or your children remind you?

**Responder:**no, they don’t even remember it.

**I:** they don’t remember

**Responder:**yes

**I:** who can help you with your medication? Who helps you at home with your medication? For example: it might be by going and buying it for you or it might be by reminding you to take it, something like that.

**Responder:**I am the one who goes and bring it.

**I:** it’s you

**Responder:**me, not even once, not even once.

**I:** family does nothing here

**Responder:**no one brings it to me. I am the one who goes and brings it. We go while talking plus it’s close. I come here to take

**I:** so your family doesn’t help you with this. What does your family think about your treatment? Your wife, your children, your family; what do they think?

**Responder:**they want me to get the treatment

**I:** get the treatment

**Responder:**get the treatment and don’t forget

**I:** are they happy

**Responder:**it might bring you some problem if it’s forgotten. They told me to bring the medicine before it’s finished. The hospital told me too. They are telling me to come and take four or five days before I finish it. Both the doctors and my family told me not to forget to take the medicine before it’s finished.

**I:** have they ever said something like” you have been taking this medicine for a long time and it’s not worth it”?

**Responder:**It’s not worth it

**I:** “what does the treatment do for you? Stop it, don’t take this medicine.”

**Responder:**no one said that

**I:** they don’t say that at home

**Responder:**no they don’t

**I:** what about your neighbors?

**Responder:**my neighbors don’t even know that I am taking it.

**I:** they don’t know that you are taking

**Responder:**they don’t know. No one knows.

**I:** haven’t you ever had a seizure on the road.

**Responder:** no, never.

**I:** okay. What else? You told me that this disease is a very serious illness. You told me earlier that it causes a lot of pain and that it’s serious.

**Responder:**yes

**I:** tell me what kind of problems that it brings

**Responder:**when I had the seizure

**I:** yes the illness

**Responder:**I can’t eat food; I bite my tongue and my lips. It brings a lot of problems. It doesn’t let me eat food.

**I:** food

**Responder:**I can eat without my tongue. Well I bite my tongue with my own teeth and I spend a lot of days without eating at that time.

**I:** you spend a lot of days

**Responder:**it didn’t happen after that

**I:** some people fell in the fire

**Responder:**yes, they fell

**I:** has it ever happened to you? Have you ever fell in a bad place?

**Responder:**no, I never fall like that

**I:** you haven’t experience such kind of things?

**Responder:**nothing

**I:** okay, this disease has some affects. For example: in social life. Does it affect your social life?

**Responder:**no

**I:** it didn’t, when I say social life I mean going to the wedding or funeral

**Responder:**yes

**I:** you go to idir, right?

**Responder:**yes

**I:** yes, meeting with your friends and families?

**Responder:**I didn’t encounter anything there

**I:** you go there like any other person

**Responder:**yes, I do. I go to some funeral. I won’t miss it.

**I:** you participate everywhere.

**Responder:**yes, I take my pills. I go everywhere. I go to Addis Ababa. Not only places that are near but also in Addis Ababa. I haven’t had much trouble.

**I:** yes, you didn’t get any problem

**Responder:**absolutely not

**I:** some people do not go anywhere and do not participate in anything because they are afraid of the thought of getting a seizure on the road.

**Responder:**there are some people who don’t go.

**I:** there are some who go, how about you?

**Responder:**I’ll go

**I:** you’ll go

**Responder:**yes

**I:** your illness doesn’t stop you from that

**Responder:**yes

**I:** okay what about in work?

**Responder:**nothing, I took my pills and do my job.

**I:** you work. You do your farming well. Do you work as you used to do before the pain started?

**Responder:**yes, I work but my eyes and I’m just getting old.

**I:** isn’t there any fatigue related to the illness?

**Responder:**I don’t get tired that much. It’s just that I’m getting old.

**I:** It’s just aging but you can work just the same as people like your peers.

**Responder:**yes

**I:** there is no any fatigue

**Responder:**yes

**I:** okay, okay the other problem of this disease is discrimination and stigma. And some people get discriminated due to this illness.

**Responder:**yes

**I:** have you ever experienced that

**Responder:**no, I’ve never experienced that

**I:** but do people know?

**Responder:**people, my neighbors know. My friends and neighbors

**I:** Do your friends and neighbors know?

**Responder:**yes

**I:** yes and they didn’t abandon their relationship

**Responder:**they spend the day with me.

**I:** they spend the day with you?

**Responder:**yes

**I:** what about your neighbors?

**Responder:**well, there are groomsmen who are with us these days. There are groomsmen in our country, right?

**I:** groomsmen, yes

**Responder:**he’s in Addis Ababa right now. They are here. They even come and beg me and I spend the day with them. In fact, the man is an elder man. He was the commander. His son is getting married. The groomsmen were there and I was with them the whole day without any worry.

**I:** okay, you never experience such kind of problem

**Responder:**yes

**I:** and you didn’t isolate yourself

**Responder:**no, I didn’t

**I:** okay, for example: there are things that are being done to people and did they ever said something like” no you can’t because you have this disease, because you don’t have the strength so this should be done by someone else” have you ever lost a job position due to this or something like this?

**Responder:**my neighbors doesn’t know about my disease

**I:** don’t your neighbors know?

**Responder:**no one knows. It’s just me and my family. My wife and my children but no one else knows except them.

**I:** no one knows

**Responder:**no they don’t know

**I:** what if they know?

**Responder:**outside, I have never fainted outside.

**I:** you have never fainted outside

**Responder:**I told you that I have fainted in my house after I worked.

**I:** yes you told me that.

**Responder:**yes, I said that I have fainted at home after I made a terrace.

**I:** yes, did you faint only once

**Responder:**yes that was the first time.

**I:** a long time ago

**Responder:**yes I used to faint every month for about four times

**I:** yes and you’re neighbor didn’t know then.

**Responder:**it was at home

**I:** it was at home

**Responder:**I fainted at home but I have never fainted on the road

**I:** you have never fainted

**Responder:**it never happened to me, but I faint in the morning or at night after I got back home from work, I have never fainted while I am walking with people.

**I:** you’ve never fainted

**Responder:**yes

**I:** so your neighbors don’t know but do you think they don’t mind if they know.

**Responder:**my son also has this disease.

**I:** does your son have this illness?

**Responder:**yes he’s sick but he goes anywhere. It gives him power. I think its different from person to person. It’s just power.

**I:** what do you mean by power? It’s just power means?

**Responder:**just force

**I:** he’ll be forceful

**Responder:**he beats people

**I:** he beats people

**Responder:** he doesn’t obey at home, he doesn’t work but he faints outside when he’s with people and no one discriminates him.

**I:** people don’t discriminate him

**Responder:**they don’t

**I:** for example: in marriage, people don’t want to marry because they think it’s transmittable.

**Responder:**yes

**I:** and some people say that

**Responder:**yes

**I:** I mean has it ever happened to you?

**Responder:**no it doesn’t. He’s just a child

**I:** what did your wife felt when you had the seizure then.

**Responder:**it happens, she was very sad but what will happen to her. She was just sad.

**I:** no I mean what did she think? Okay, you told me that you didn’t go anywhere, right? You said you didn’t go anywhere else. You went only to butajira for the treatment, right?

**Responder:**I told you that I went to butajira and Addis Ababa. But they didn’t accept me in Addis Ababa

**I:** they didn’t treat you in Addis Ababa

**Responder:**they didn’t

**I:** okay you told me that you don’t have any problem; you can go anywhere as you like. You were your clothes by yourself, right?

**Responder:**yes

**I:** I mean right now

**Responder:**yes

**I:** what about then? When you were sick?

**Responder:**when I was sick, I changed my clothes. I was very sick. It bothers me so I changed my clothes.

**I:** yes at that time, your family was taking care of you because you couldn’t treat yourself

**Responder:**yes

**I:** people took you to the hospital

**Responder:**yes

**I:** but now that you are fine

**Responder:**I come by myself

**I:** you come by yourself. Okay now let me ask you a general question. You told me your son has the same illness. There are families around your neighbors. You told me that this disease bring many damage. You said that it’s a very bad disease and that it brings many harm and

**Responder:**yes

**I:** what do you think should be done to improve the life of people who has this disease?

**Responder:**we don’t have strength

**I:** no, in your opinion, what do you think that the government or the health center or the neighbors should do in order to improve their life?

**Responder:**I say that the government should help us with this disease but who else will help us?

**I:** what can the government do for example?

**Responder:**it should help us by providing the medicine and if there is anything else, it should help us with anything.

**I:** is there any problem with the supply of the pills?

**Responder:**there is no problem with the supply now, it’s very great.

**I:** have you ever had a problem with the supply of pills?

**Responder:**it was a long time ago. I told that I used to take the pills from butajira. Well, it’s coming right now it’s great. But before that we used to go to Addis Ababa and also butajira and take it from there. They wrote to us from here and we go to butajira and take it from there. We have a record there and we used that record to take the medicine, but now it’s getting better here. It has become a very exciting hospital right now.

**I:** you can get the medicine often

**Responder:**yes, we get it often, it’s great.

**I:** okay what should the hospital do?

**Responder:**anything that he can

**I:** no

**Responder:**how can we say help us with this?

**I:** no if you have any suggestion we will pass it on. And if you have any idea, it’s useful. Don’t take it for granted. Your opinion is very useful because you have been living with it and you have seen what it causes and what damage it can do. You are free from it but there are a lot of people who are suffering because of it. So what do you think should be done?

**Responder:**yes there are those who are suffering a lot

**I:** yes, if there is anything that you can say about what should be done for them?

**Responder:**if they can help us with what they can. There are those who cannot afford to pay so the government should help us with this. And we will be grateful and our illness will decrease.

**I:** no it’s about the disease

**Responder:**about the disease, they are giving us the pills. The pill is not free. We take it by our book so we pay for a year. So if it has the ability to help us, he should.

**I:** what about the health center, the hospital?

**Responder:**it’s giving us the pills and what else can it help us with?

**I:** okay what about the society

**Responder:**the society?

**I:** the community

**Responder:**the community, they themselves couldn’t.

**I:** they couldn’t

**Responder:**father, should helping be only with money? Isn’t there any kind of help?

**I:** in countryside, no. there’s nothing.

**Responder:**for example, I told you that there are those who have work and health related problems, right?

**I:** should we help farming?

**I:** yes, isn’t there such kind of help?

**Responder:**the neighbors say “give us.” If they say do this for us; they’ll go out of the country. We don’t tell them what to do.

**I:** don’t they help? Don’t they cooperate?

**Responder:**no

**I:** people shouldn’t get sick.

**Responder:**oh, God of law, I don’t tell them what they should do. Whatever they do, they should do it on their own. If the government can he should help us with something.

**I:** okay. Okay, my last question is, how does this disease affect your life, was there something that you wanted to do but you couldn’t because of the disease?

**Responder:**I, myself?

**I:** yes, you, yourself

**Responder:**my wife takes care of me; she helps me a lot when I get stressed. Sometimes it might be with farming or anything else; we buy someone and we work together.

**I:** does your wife help you?

**Responder:**yes she helps me with everything

**I:** I mean if there’s something that prevents you from doing because of your illness?

**Responder:**there are people who hire some people who can do their work for them. But I do my own job on my own. If I get a person who works for me, I would get a little rest. I am getting old. If I cultivate the field with this disease plus I am old so I am going to the ground. I have to go up now. If the children cooperate and help me but they are not here. No one stays at home nowadays, they all are working.

**I:** they are working

**Responder:**they are. It’s getting expensive. If one person is for 10000 birr, if they do that it would be great. But I should work. Well, I should work.

**I:** yes, work. Okay if there is anything that you want to add, if there is anything that I didn’t ask you?

**Responder:**there is nothing

**I:** nothing?

**Responder:**I have finished

**I:** okay, I have finished thank you.
