# Supplementary material for: Experience and perceptions of mental ill-health in people with epilepsy in rural Ethiopia: A qualitative study
Source: PLoS One. 2024 Dec 13;19(12):e0310542. doi: 10.1371/journal.pone.0310542 (PMC11643256; doi:10.1371/journal.pone.0310542)
Supplement: S3 File — (ZIP) [file pone.0310542.s003.zip › data set/translation 010.docx]

**P-010**

**Interviewer**: Okay, thank you very much for being volunteer and coming here for this interview. First, tell me about your illness. You come and follow-up at health center, right?

**Interviewee**: Yes

**Interviewer**: Okay, what type of illness do you have?

**Interviewee**: It is *Azurit*.

**Interviewer**: Is it called *Azurit* in your community

**Interviewee**: Yes

**Interviewer**: How do you feel?

**Interviewee**: It seizes me.

**Interviewer**: It seizes. What else? Doesn’t it have other symptoms?

**Interviewee**: Symptoms; swelling around my mouth

**Interviewer**: Is it when you seize?

**Interviewee**: When it is about to seize me.

**Interviewer**: Okay, what else?

**Interviewee**: It swells and fall me.

**Interviewer**: Do you twitch?

**Interviewee**: Yes

**Interviewer**: Do you have headache?

**Interviewee**: I lose consciousness and just fall.

**Interviewer**: Do you lose your consciousness?

**Interviewee**: Yes

**Interviewer**: Do you lose your consciousness for long time?

**Interviewee**: I will sleep for a while and then I will wake up.

**Interviewer**: How long it has been since it started you?

**Interviewee**: Ten years.

**Interviewer**: Will it be ten years?

**Interviewee**: Yes

**Interviewer**: When did you fall when it first begin you?

**Interviewee**: While I was grade six.

**Interviewer**: When you are grade six?

**Interviewee**: Yes

**Interviewer**: Did you fall at school?

**Interviewee**: At football field.

**Interviewer**: When you are playing?

**Interviewee**: Watching

**Interviewer**: What was done at that time?

**Interviewee**: They took me to hospital.

**Interviewer**: Did your family take you?

**Interviewee**: Yes, then the doctor examined me and I started medication at *Butajira*.

**Interviewer**: Is it at *Butajira* that they say you have epilepsy and take medication?

**Interviewee**: Yes, they said it is epilepsy and I started medication.

**Interviewer**: Did you fall at school football field?

**Interviewee**: No, it is club

**Interviewer**: Did you play at club?

**Interviewee**: No, I went to watch.

**Interviewer**: Did you go to watch?

**Interviewee**: Yes

**Interviewer**: How was the people reaction?

**Interviewee**: I didn’t fall but I went to home when I had confusion since I was worried about what it was. I fall immediately when I went to home.

**Interviewer**: When you had blurred vision and worried?

**Interviewee**: I came home when I was worried. As soon as I got home, I screamed and called others and fall there.

**Interviewer**: Did your family find you then?

**Interviewee**: My family

**Interviewer**: Did you fall after that?

**Interviewee**: Then, I fall very month.

**Interviewer**: While talking the medication

**Interviewee**: Yes, it seizes me while taking the medication.

**Interviewer**: Did it seize you every month?

**Interviewee**: Sometime I didn’t expose for sunlight

**Interviewer**: Is it when you are exposed to sun?

**Interviewee**: I will have dizziness when I am exposed to sun.

**Interviewer**: Have you ever been injured?

**Interviewee**: When I fall?

**Interviewer**: Yes, such as losing teeth, falling to fire; things like that.

**Interviewee**: Once, at 2010, I was with my friend and got together to celebrate his birthday. As soon as I got home, I sat down next to her and I fall on the chair and then drop to the floor then my teeth bleeds; that is all, I have never been hurt.

**Interviewer**: You never had, okay. Did you have any other illness besides the epilepsy?

**Interviewee**: Gastritis

**Interviewer**: Not gastritis; did you have any other mental illness?

**Interviewee**: I don’t have other than epilepsy.

**Interviewer**: Don’t you have any other?

**Interviewee**: I don’t have.

**Interviewer**: There is something like mental illness among those people who have epilepsy, so do you have feeling of depression?

**Interviewee**: No

**Interviewer**: What about drinking alcohol and chewing *Khat*?

**Interviewee**: No

**Interviewer**: Alcohol and other substance use

**Interviewee**: No, I don’t drink alcohol except coffee.

**Interviewer**: Coffee, okay. Don’t you have any other illness?

**Interviewee**: I don’t have.

**Interviewer**: Okay, so it is only the epilepsy.

**Interviewee**: It was but now

**Interviewer**: Are you fine now?

**Interviewee**: I am fine since 2012.

**Interviewer**: Are you taking the medication or did you stop taking it?

**Interviewee**: I stopped.

**Interviewer**: Did you stop? Aren’t you taking any medication now?

**Interviewee**: Yes

**Interviewer**: Who told you to stop?

**Interviewee**: My family. He said I will not give you medication when I told him to get it. He told me I will not give you and stop. Then I decided that to stop and pray if God cure me. And the medication also burn my heart, after that I didn’t take the medication.

**Interviewer**: Didn’t you take after that? Didn’t you seize since the two years?

**Interviewee**: Never

**Interviewer**: Were you taking medication at 2010?

**Interviewee**: Yes, I didn’t seize since I start taking from here. I had not been taking it continuously, I have no one to bring the medication when he is not in the nearby.

**Interviewer**: Wait, we will talk about him later. Did you say I have heart burn beside the epilepsy?

**Interviewee**: Yes, recently.

**Interviewer**: How do you feel?

**Interviewee**: I will be tired.

**Interviewer**: Tired?

**Interviewee**: Yes, I will sit down.

**Interviewer**: Is it tiredness?

**Interviewee**: I will be tired and it pulls me down like gastritis.

**Interviewer**: Like gastritis?

**Interviewee**: Yes, I have burning sensation.

**Interviewer**: Do you have difficulty of falling asleep, loss of appetite and stress?

**Interviewee**: I don’t have like that.

**Interviewer**: Don’t you have?

**Interviewee**: Yes

**Interviewer**: What about extreme happiness?

**Interviewee**: There is no extreme happiness.

**Interviewer**: So, do you have only epilepsy?

**Interviewee**: Yes

**Interviewer**: Okay. What do this epilepsy disease affected your life? For example, you told me as you were student, right?

**Interviewee**: Yes

**Interviewer**: Did it impose effect on your education?

**Interviewee**: At that time, I can’t attend my education and I don’t hear when there is noise in the class room.

**Interviewer**: Did the noise stress you?

**Interviewee**: Yes, I can’t hear noise like crying

**Interviewer**: Can’t you hear?

**Interviewee**: Yes

**Interviewer**: What about attending class?

**Interviewee**: This is it.

**Interviewer**: Did the noise stress when you went to school?

**Interviewee**: I didn’t like when the students shout.

**Interviewer**: Did you feel stress?

**Interviewee**: Yes

**Interviewer**: Did you seize at school?

**Interviewee**: At school, yes.

**Interviewer**: Did you seize?

**Interviewee**: Yes, while I was at elementary.

**Interviewer**: What did your friend say at that time?

**Interviewee**: They didn’t say anything; they took me to my home.

**Interviewer**: Did they take you to your home?

**Interviewee**: Yes

**Interviewer**: Was there discrimination by students at school since you seized?

**Interviewee**: No

**Interviewer**: Was there nothing? As some people think it is contagious.

**Interviewee**: Yes

**Interviewer**: Didn’t you encounter things like ignorance from some of your friends?

**Interviewee**: No

**Interviewer**: What about family?

**Interviewee**: Nothing

**Interviewer**: Didn’t you experience such type of things since you have epilepsy?

**Interviewee**: No

**Interviewer**: Is there nothing?

**Interviewee**: Yes

**Interviewer**: Not getting service that you should get, for example, it could be on wedding

**Interviewee**: There is nothing.

**Interviewer**: Okay. So, it didn’t have any impact on your education other than not going to school as the noise stress you.

**Interviewee**: Even though I went, I will take air and get back

**Interviewer**: Did you go out and return when you are stressed?

**Interviewee**: I will go back when I am fine.

**Interviewer**: Did it have effect on your grade?

**Interviewee**: No, it didn’t.

**Interviewer**: For example, you said as you become sick when you are thirteen years old

**Interviewee**: At third grade.

**Interviewer**: Which it started you?

**Interviewee**: Yes, I started with six medications. I didn’t know myself, and I am the one who pushes the mud.

**Interviewer**: You seized while you were at third grade?

**Interviewee**: It didn’t have feeling after that. There was nothing, but after three years I relapsed and started me while I was at sixth grade.

**Interviewer**: After that? But, didn’t you have anything in the middle?

**Interviewee**: There was nothing for three to four years.

**Interviewer**: Was there nothing?

**Interviewee**: Yes

**Interviewer**: What about after the sixth grade?

**Interviewee**: Just from sixth grade to tenth grade

**Interviewer**: Didn’t the illness have effect on your grade?

**Interviewee**: Lowering

**Interviewer**: Lowering, lack of attention to education while the teacher teaches; didn’t it bring about such type of things?

**Interviewee**: If I didn’t feel, there was bi such type of thing.

**Interviewer**: So, are you paying attention to your education?

**Interviewee**: Yes

**Interviewer**: What about at home work?

**Interviewee**: I work at home.

**Interviewer**: Do you work like your brothers and sisters?

**Interviewee**: I work a little bit, as I don’t have the strength

**Interviewer**: Will you be tired?

**Interviewee**: It is not being tired but they don’t also want me to work.

**Interviewer**: Don’t they ask you to work?

**Interviewee**: Yes, but I work less than them.

**Interviewer**: You told me as you have friends.

**Interviewee**: Yes

**Interviewer**: What about participating on social life, for example, it could be on mourning, going to church? Doesn’t your illness prevent you from that?

**Interviewee**: No

**Interviewer**: Okay, good. You had different type of symptoms and you told me as you are fine now.

**Interviewee**: Yes

**Interviewer**: Let’s talk about the past; did you have the symptoms that you don’t wish to have at that time?

**Interviewee**: Yes, I cried at that time since I always fear the medication. They told me as the medication will be taken life long and I just stopped taking it.

**Interviewer**: Among your illness symptoms, you told me as you had shivering, loss of consciousness and seize

**Interviewee**: Yes, I seized.

**Interviewer**: What was the symptom that you don’t like? Is it the seizure?

**Interviewee**: The whole disease.

**Interviewer**: The whole disease.

**Interviewee**: Yes

**Interviewer**: Okay. You told me as they took to *Butajira* for the treatment as soon as the disease started you

**Interviewee**: Yes

**Interviewer**: What else? Did you take any other treatment like traditional medication?

**Interviewee**: I didn’t.

**Interviewer**: Is it prayed for you?

**Interviewee**: Yes

**Interviewer**: Why did they take to *Butajira* first?

**Interviewee**: There is no treatment here.

**Interviewer**: Why didn’t they take you to traditional medicine or prayer?

**Interviewee**: I went to holy water first.

**Interviewer**: Did they take you there?

**Interviewee**: They bring holy water to my home and I had been using it, but it didn’t improve me much and then I went to *Butajira*.

**Interviewer**: Did you go there?

**Interviewee**: I went to psychiatric hospital and it was known as it is epilepsy.

**Interviewer**: But before that it was holy water that was tried.

**Interviewee**: Yes

**Interviewer**: Did you go there since nothing improved?

**Interviewee**: Yes, then I went to *Butajira*.

**Interviewer**: You went to *Butajira*, and when did you start the follow-up?

**Interviewee**: When it is said it is started here.

**Interviewer**: Did you come here when it said it is started?

**Interviewee**: Yes, it is better here.

**Interviewer**: For proximity?

**Interviewee**: Yes, for the proximity.

**Interviewer**: What about the hospitality and care, as you saw both?

**Interviewee**: There is no questioning there, there is nothing.

**Interviewer**: Where? Is that at *Butajira*?

**Interviewee**: Yes, you will go there, will be asked and you will just take the medication.

**Interviewer**: Do they ask you well here?

**Interviewee**: Yes

**Interviewer**: What do they ask you, as example?

**Interviewee**: As how is your health year after year?

**Interviewer**: Do they always ask you every time you come?

**Interviewee**: Yes, they asked me.

**Interviewer**: Then you will take your medication and go

**Interviewee**: Yes

**Interviewer**: Okay. Do they monitor your improvement? Do they ask you do you have improvement or something new?

**Interviewee**: Yes

**Interviewer**: Are you happy with what you got here?

**Interviewee**: Yes

**Interviewer**: Do you have anything you say that should be improved or added?

**Interviewee**: No

**Interviewer**: Why did you stop it?

**Interviewee**: I am healed and completely healthy.

**Interviewer**: By prayer?

**Interviewee**: There is prayer for the disease to don’t relapse.

**Interviewer**: Did you stop it since you are healed and you don’t need it now?

**Interviewee**: Yes

**Interviewer**: Was your father bored?

**Interviewee**: Most of the time, he was the one who brought the medication and pay for it.

**Interviewer**: Will you take the medication if it is given for free now?

**Interviewee**: No, I will not take it.

**Interviewer**: Why?

**Interviewee**: Believe is the paramount, so you believe I don’t need medication since I am saved.

**Interviewer**: So do you believe I don’t need medication?

**Interviewee**: Yes

**Interviewer**: Are there effects or harms you encountered because of the drug?

**Interviewee**: No

**Interviewer**: Earlier, you said my hurt burn it could be the medication

**Interviewee**: I stooped it before it hurt my heart.

**Interviewer**: Did they tell you about the medication when you took it from here?

**Interviewee**: Yes, they told me to take it on time properly.

**Interviewer**: What else would happen if you stop it?

**Interviewee**: Another, they told me that it is not good if the medication adapts. It is not good of the medication of any disease adapt.

**Interviewer**: Have you ever forgotten to swallow while taking the medication?

**Interviewee**: Never

**Interviewer**: Who reminds you?

**Interviewee**: Myself, I don’t forget.

**Interviewer**: Did your family help you on this?

**Interviewee**: Yes

**Interviewer**: Who helps you more?

**Interviewee**: All of them.

**Interviewer**: Does everyone help you?

**Interviewee**: Yes

**Interviewer**: Do they want to take the medication or not?

**Interviewee**: They wanted to take the medication.

**Interviewer**: Do they think to continue the medication it since help you?

**Interviewee**: Yes, they said like that.

**Interviewer**: Is that your father who doesn’t want it?

**Interviewee**: In the past, yes but now he says take it.

**Interviewer**: Why do you think they say take it?

**Interviewee**: He said you will not be healed as he is not the follower of the religion.

**Interviewer**: Are you protestant?

**Interviewee**: Yes, I am protestant and they are Orthodox.

**Interviewer**: So, do they say you have to take the medication?

**Interviewee**: Yes, they say prayer don’t heal.

**Interviewer**: Did they want you to take the medication because of that?

**Interviewee**: Yes

**Interviewer**: Okay. Did you remember what they asked you when you first went to *Butajira* health center?

**Interviewee**: He just looked at me and he told my father that it is epilepsy. He just touched my head like this.

**Interviewer**: Didn’t they ask you?

**Interviewee**: Nothing

**Interviewer**: Only by looking?

**Interviewee**: Yes

**Interviewer**: When you go to health center they may ask you about your personal life; would you be upset if you are asked about your personal life? Did they ask like that? Have you ever been asked like that?

**Interviewee**: Because of this disease?

**Interviewer**: Yes. Have you ever been asked that if you feel anxious and if you have suicidal ideation when you go to *Butajira* health center?

**Interviewee**: No

**Interviewer**: How would you feel if they asked you?

**Interviewee**: It doesn’t matter.

**Interviewer**: Why do you think they ask you? Do you think it is good if they asked you?

**Interviewee**: Yes

**Interviewer**: What does it benefit you?

**Interviewee**: To know what I have to do next.

**Interviewer**: Okay. People encounter a lot of things that prevent them to go to the health center and follow-up their treatment and because of that they will stop their treatment and will come back. Have you ever had difficulty to get your medication and discontinued it?

**Interviewee**: I don’t.

**Interviewer**: Do you always come?

**Interviewee**: I do.

**Interviewer**: Some people may not be able to come it could be due to lack of money or workload at home, and didn’t you be able to come?

**Interviewee**: No, I didn’t.

**Interviewer**: Didn’t you?

**Interviewee**: Yes

**Interviewer**: Okay. But you stopped it because your father didn’t have money to pay, right?

**Interviewee**: I want to stop it first and I stopped when he complain that.

**Interviewer**: Did you stop it?

**Interviewee**: Yes

**Interviewer**: Your family thinks your treatment is good, right?

**Interviewee**: Yes

**Interviewer**: Did your friends think like that?

**Interviewee**: Yes

**Interviewer**: Okay. What do you think should be done to improve the overall lives of people with epilepsy including you? When I said there social life, it means mourning, wedding and else. What do you think should be done to make their life better like anyone and for students to be well educated?

**Interviewee**: If people with epilepsy lives with people, if there family and the government support

**Interviewer**: What kind of support?

**Interviewee**: It means, not pressuring them to live with other people but it is good if the others people help them when they ask for something and if they are treated equally. It is good if they are encouraged not to feel inferior and it is good if the government support them.

**Interviewer**: Okay. What do you think the health center professionals should do?

**Interviewee**: If the health center gives them priority.

**Interviewer**: Is that for them to don’t wait in queue?

**Interviewee**: No, if care is given for them.

**Interviewer**: Okay. What should be done from the health institutions, hospital?

**Interviewee**: Politeness by itself.

**Interviewer**: If they give them love and care

**Interviewee**: Yes

**Interviewer**: What do you think should be done to make them as good as anyone else? What do you think the community should do?

**Interviewee**: If they are given love and if they are given what they want to let them they are equal to the others.

**Interviewer**: You told me if they don’t discriminate them, right?

**Interviewee**: Yes

**Interviewer**: Have you experienced that?

**Interviewee**: No but when the other said it is contagious

**Interviewer**: You heard that, but have you never experienced like that from your family?

**Interviewee**: I have never experienced such type of thing from my family.

**Interviewer**: Okay. What do you think should be improved at the health institutions to improve their lives? There re institutions that are working, for example, there are schools, so what is expected from them? You told me that if priority and care is given to them, so what about schools? There are those who get sick while they are student, right? You were sick while you were sixth grade; what do you think should be done to improve the lives of students?

**Interviewee**: If the teachers know as it is disease and supports them and teaching them as it is not contagious.

**Interviewer**: Are there teachers who think it is contagious?

**Interviewee**: There are.

**Interviewer**: What about the other institutions? Do you have anything else to say that I didn’t ask you?

**Interviewee**: No

**Interviewer**: Okay, I am done, thank you!
