# Supplementary material for: Experience and perceptions of mental ill-health in people with epilepsy in rural Ethiopia: A qualitative study
Source: PLoS One. 2024 Dec 13;19(12):e0310542. doi: 10.1371/journal.pone.0310542 (PMC11643256; doi:10.1371/journal.pone.0310542)
Supplement: S3 File — (ZIP) [file pone.0310542.s003.zip › data set/translation 011.docx]

**I:** Thank you so much for your willingness to do the interview. Let’s start with the problem that brought you to the health center. What was the problem?

**Responder:** Today or?

**I:** No. when you first got treated. How were you when the disease started?

**Responder:** I didn’t know at that time. It was my family who took me to get the treatment and I was a child.

**I:** How old were you?

**Responder:** Thirteen

**I:** That’s about ten years ago

**Responder:** Yes

**I:** Okay

**Responder:** It was only a month after that, that I become conscious. I was not aware.

**I:** You were unconscious

**Responder:** Yes, after two months I become conscious and was prescribed medicine in Addis Ababa but I didn’t know anything at that time.

**I:** Were you first got the treatment in Addis Ababa?

**Responder:** Yes

**I:** You told me that you didn’t know, but what did they say about how you were then?

**Responder:** First I fainted around the river. I got back from school and it was a sunny day. Then I went out to the river to fetch some water for the vegetables. Then I fainted there. After that they took me to the health care and they refer me to Addis Ababa. Then after getting in Addis Ababa, I become conscious after two months.

**I:** Were you unconscious till then?

**Responder:** Yes

**I:** You didn’t know where you go or where you were

**Responder:** Yes

**I:** You were unconscious

**Responder:** Yes

**I:** Did something hit you when you faint around the river; your teeth or your eyes something like that?

**Responder:** No, nothing

**I:** There’s nothing?

**Responder:** Yes

**I:** It’s just that you fainted

**Responder:** Yes

**I:** Okay, they took you to the health care first, when they found you like that.

**Responder:** Yes

**I:** But they didn’t take you to Tsebel or to the mosque or something like that

**Responder:** No, they didn’t take me there

**I:** Does it ever happen to you before? Have you ever fainted before that?

**Responder:** No, before when?

**I:** I mean before the first time

**Responder:** No

**I:** Was it your first time?

**Responder:** Yes

**I:** Okay what about after that?

**Responder:** After that it’s just medicine

**I:** You started the medicine

**Responder:** While I was in the medical follow up

**I:** Does it make you faint still?

**Responder:** Not that much. I used to faint while I was taking the medicine. Sometimes there’s headache and stress. I was not willing to take the medicine on time. Then I got better through time. I spent two years without fainting and I was taking the medicines. But it comes back again before a week or so. I have talked to the doctors and I told them there is no any symptom and I was not like I used to. And I asked them why. They told me that the medicine will make me faint until a year and they asked me why I quit taking it. Then I said okay and I counted the days and when I reach the second year I told them. And they said they would change the medicine so I said okay and I was taking it. Finally about last week, I went to the countryside and it got me faint there. But I was taking the medicine.

**I:** While you were taking it?

**Responder:** Yes

**I:** Was there any different thing that made you get stressed?

**Responder:** There’s nothing else. It was just the stress. Sometimes I get stressed and not even know it. There are times when there are a lot of thoughts but there was nothing at the time when it got me faint. I didn’t stop taking the medicine so I thought I was okay like before so I haven’t thought about anything. I haven’t talked to the doctors; I was just taking the medicine.

**I:** First let’s talk about the seizure you have. It makes you faint and what other symptoms do you have?

**Responder:** Nothing

**I:** You said that it makes you faint

**Responder:** Yes

**I:** What else happen when you had the seizure?

**Responder:** There is no other symptom. It makes me faint while talking to you but there is no any sign. I wouldn’t have been hurt if it had a sign.

**I:** You become aware when you wake up?

**Responder:** Yes. I think the disease differ from person to person. I heard this from a friend that there are people who get a sign when they are about to faint. But I know nothing.

**I:** Yours don’t show any sign?

**Responder:** It just makes me faint. It doesn’t show me any sign and it has done many harm because I didn’t know.

**I:** What damage did it do to you if you can tell me? You said it has done so much harm on you right?

**Responder:** yes

**I:** Is there anything that you can tell me about the damage it has done to you?

**Responder:** One reason is that I was living with my family and they were not willing that much. My father was not willing because I was sick and living with them. They bring the medicine from butajira and then one day he left me after saying that he doesn’t care and I can do whatever I want. I was getting sick a lot at that time.

**I:** You were not getting better. Is that why your father said that he doesn’t care or what do you think?

**Responder:** I don’t know if it’s because I’m not getting better or he’s just getting tired. The medicine doesn’t even cost that much. There was a time when you can buy it for 6 birr. But it’s getting expensive now, the transport also. I was living with him in the countryside so they used to give him the medicine that’s enough for 3 months. But then he said he doesn’t care and throws the card. And I didn’t know where I can buy it in butajira. Then I said okay and I come here and stayed with my uncle. Then I came to the hospital and beg them. They said they can’t give me if I was not referred to them. I begged them crying and then I stayed the night in the city. Then I started taking the medicine from here. And then I got married and I started living with my husband and I took money from him and buy the medicine and use it. My family was careless. After I got married I started getting better. But when I was with my family I was not, not even once. The disease doesn’t like working and getting angry. But my family, my father, my brother or anyone doesn’t understand. Both the literate and the illiterate blame me. So I get a lot of stress when I was with my family. I was doing fine for years but I got sick again.

**I:** Did you faint once since then?

**Responder:** Yes, once last week

**I:** When you faint, is there any foam coming out of your mouth?

**Responder:** Yes

**I:** What about shaking

**Responder:** They told me there’s foam but the shaking comes after I wake up

**I:** Did it make you shake?

**Responder:** Yes after I wake up

**I:** Okay, is there any other illness besides the epilepsy

**Responder:** No

**I:** You told me you have stress

**Responder:** Yes

**I:** What does the stress make you feel?

**Responder:** when I got depressed, I just know it. Most of the time, I spend the day at home fearing that it might make me faint. I hate working.

**I:** Do you get stressed when it’s a cloudy day?

**Responder:** Yes, I got stressed when it’s cloudy, when there’s so much to think about. Then I fear that it might make me faint so I don’t go out. At that time I take care of myself. There’s so much stress more than anything, more than the epilepsy even. I even make myself stressed

**I:** Does it make you feel anxious?

**Responder:** Yes

**I:** What do you feel when you say you are stressed? If you can describe it with an example

**Responder:** Sometimes I try to kill myself, when I get stressed. I think of so many things, but what can I do if God gives.

**I:** You told me that you get stressed and depressed when it’s cloudy

**Responder:** Yes

**I:** So you hate being with people during that time.

**Responder:** Yes, I hate it when people talk to me. I don’t like sitting and talking to people like this. It’s just, even if they talk with good intention I don’t respond to them well.

**I:** What about food

**Responder:** It’s just,

**I:** You don’t want to eat at that time

**Responder:** Yes

**I:** What about sleep

**Responder:** It couldn’t make me sleep

**I:** What about work? Doing the house chores

**Responder:** I don’t work

**I:** What about your children? How are you with taking care of them?

**Responder:** My elder son is with my mother. This one’s my second.

**I:** Taking care of them or is there anything like hating them?

**Responder:** No

**I:** I mean when you are depressed

**Responder:** No there is nothing like that.

**I:** What about with your husband

**Responder:** We don’t always have peace with my husband.

**I:** Why? Is it because of your illness or any other problem?

**Responder:** He also doesn’t understand me. When he wants to say something to me, it’s with an insult. When I felt anger inside, I don’t respond well and he just can’t stand it and I can’t stand it.

**I:** Is this when you are stressed and depressed or..?

**Responder:** No I get stressed sometimes. It’s not always

**I:** What do you mean by always?

**Responder:** I’ll not be in love with him even if I’m okay. It’s just that I get angry if he said something to me. I get anxious and no one understands that.

**I:** He doesn’t understands you

**Responder:** Yes

**I:** You get angry because he doesn’t understand you

**Responder:** Yes

**I:** do you feel like ”I am sick why can’t he understand that.”

**Responder:** Yes I get angry

**I:** The depression that you told me now, does it happen always or

**Responder:** Sometimes

**I:** It comes when what happens to you? You told me when it’s cloudy, for example.

**Responder:** Firstly, I don’t like when winter comes in. I hate it when it’s cloudy; I don’t even go out. Secondly, when I have a lot to think about.

**I:** The thought about life and your home right?

**Responder:** Yes, I get stressed when I think about it.

**I:** Do you think that the stress and the epilepsy are related?

**Responder:** Yes

**I:** How do you think they are related? If you don’t have the disease, do you think this shouldn’t be the case, or if you think that they relate, tell me how?

**Responder:** I just think they have relationship.

**I:** How?

**Responder:** Before I had this disease, I never had stress. It was only after this disease that I experience stress.

**I:** The thing that depresses you when it gets cloud, didn’t you have it in the past?

**Responder:** No

**I:** It was after this disease.

**Responder:** Yes it’s after this disease; I didn’t have it in the past

**I:** Do they consider it as a disease in your environment, I mean the stress. In your environment, the feeling of depression and stress, do you call it a disease or you don’t consider it as one.

**Responder:** Yes

**I:** What about you, do you think that it’s a disease in itself or what?

**Responder:** I don’t think so. I know I hurt myself.

**I:** Do you think that the thing that makes you faint is a disease?

**Responder:** Yes

**I:** What do you call it in your environment?

**Responder:** Epilepsy

**I:** You call it epilepsy

**Responder:** Yes

**I:** Okay, the thing that makes you feel stressed and depressed, do you think it’s another disease or?

**Responder:** I don’t think so. They are the same; I don’t think they have a difference.

**I:** Are you saying the epilepsy is making me this way or do you think there is another disease called depression?

**Responder:** I think they are the same

**I:** So you think the epilepsy is making you this way

**Responder:** Yes

**I:** Okay, you said that it has brought a lot of problems in your life. You said both the stress and the epilepsy have had a profound effect on your life. Let’s talk about each one by one. How does it affect your work, your education? Do you stop your education because of it?

**Responder:** I dropped out of school because of myself, because I didn’t get help from anyone in the house. My first reason is my family. My second reason is that I didn’t have any interest in education and I didn’t have any knowledge. I just went to school but I was not interested.

**I:** Is it after you started getting sick or is it before?

**Responder:** It’s after I get sick. I didn’t understand any of it so I stopped learning. I was not okay inside and I couldn’t agree with my family so I left school and came here

**I:** What about work? You told me that you don’t have a job but have you ever worked before?

**Responder:** I’ve worked a lot.

**I:** Does it affect your job?

**Responder:** As I told you before it used to make me faint while I was working. I have worked for many years. I have even worked here for five years as a servant. After that I got married. I used to get sick a lot at that time. I was using my medication and thanks to God I got fine after that.

**I:** At the time when you were having a seizure during your work time, were you working on your own business or were you employed?

**Responder:** I was working at someone else’s house.

**I:** You were working in someone else’s house

**Responder:** Yes

**I:** And what did the people say when you faint?

**Responder:** They didn’t say anything because they didn’t know the disease. They run away from me. No one approached me; everyone thought it’s transmittable so they run away from me. No one came near me and talked to me. I used to treat myself when I become conscious again.

**I:** But they didn’t do anything for you

**Responder:** They didn’t do anything for me

**I:** What about trying to fire you?

**Responder:** No I’ve never experienced that

**I:** Haven’t you?

**Responder:** yes

**I:** What about your friends? Did they lose their friendship with you after seeing you faint, thinking that it might get passed on to them?

**Responder:** No

**I:** And your family? Why were they like that?

**Responder:** How

**I:** You don’t have a good relationship with your family. You told me that you lost the relationship you had with your father because of this disease.

**Responder:** Yes

**I:** Why do you think that is?

**Responder:** I just don’t know anything

**I:** Do you have sisters?

**Responder:** I have.

**I:** Do you also have brothers?

**Responder:** I have but they don’t see me as equal as them

**I:** Why do you think they don’t see you as equal?

**Responder:** It’s just, you know.

**I:** Is it because of your illness or do they have other reason?

**Responder:** I don’t think there’s another reason. I guess my father is because of it. The rest, my brothers and sisters are younger than me and they know nothing. One of my sisters was not at home with us. But I know my father’s reason is because of that.

**I:** What different thing happened to him that makes him want to say like that. For example from his friends, his neighbors

**Responder:** I mean when I first got sick, he spent a lot of money. He even sold the cattle. If God doesn’t say “it’s your time”, then you don’t die so then I become myself again. Then he said “I sold all my cattle for you, I spent a lot of money for you and I don’t care about you from now on, you can go where ever you want.” And at that time when he said that to me my mother didn’t even say a word.

**I:** They stayed silent

**Responder:** Yes, my brother, he is elder than me, he’s elder than all of us. At least he was an educated man but he didn’t even say “she’s going to get hurt, don’t say that to her” there was no one who cares for me.

**I:** Isn’t there anyone who defended you against your father?

**Responder:** No one. No one talked to him about why he was doing this. I got hurt a lot while I was with my family. I got better after I left them. I was always busy with work, I got frustrated a lot. But after I left them, thanks to God, I am better than the past.

**I:** You now have peers here and in the countryside as well right? When you compare yourself with them do you think you’ll have a hard time working because of it?

**Responder:** I don’t think so

**I:** Do you visit them?

**Responder:** Yes

**I:** You do what you have to do?

**Responder:** Yes

**I:** So you don’t think your illness has effect.

**Responder:** I don’t think so

**I:** What about your marriage. When you were planning to get married, did your illness affect you? Does your husband knew before you get married?

**Responder:** He didn’t know

**I:** He didn’t know that you were sick?

**Responder:** yes

**I:** He found out after he got married to you

**Responder:** Yes

**I:** What did he say when he found out?

**Responder:** Then he told me that he had it and that he drank a traditional medicine.

**I:** Does your husband said that?

**Responder:** Yes, he told me that it was a simple thing. And he was the one that helps me get the treatment. Then I lived for three years, thanks to God.

**I:** How many years have you been with your husband?

**Responder:** Ten years

**I:** Ten years?

**Responder:** Yes

**I:** It’s been a long time. So he accepted you at first and he was treating you.

**Responder:** Yes

**I:** So your conflict is not because of your illness, it’s because of other reason

**Responder:** Yes, it’s because of other reasons, he never said anything about my disease. It’s just that he has no sympathy. He doesn’t think that I get sick if I get angry. If he doesn’t speak to me in good spirit, I get angry. If he speaks to me arrogantly, I get unconscious. There are times that I get unconscious while I am sitting. He speaks nonsense to me and then I get unconscious and for a while I couldn’t identify a person. I realize that I got so sick after I return back to myself

**I:** You told me you have different symptoms like getting unconscious, getting anxious, suicidal thoughts.

**Responder:** Yes

**I:** Above all this, which one is the most difficult for you, that you wish it to disappear?

**Responder:** The disease

**I:** No, you want the disease to go away but if you are told that you can get rid of only one of the symptoms, which one would you probably choose?

**Responder:** The stress

**I:** The stress disturbs you a lot

**Responder:** Yes

**I:** What about the seizure

**Responder:** It happens sometimes, it comes back after two years.

**I:** There’s also the seizure

**Responder:** Yes

**I:** From the seizure and the stress which one is very disturbing?

**Responder:** The stress

**I:** The stress is a lot for you.

**Responder:** Yes

**I:** It disturbs you a lot

**Responder:** Yes

**I:** You can’t work when you get stressed

**Responder:** I can’t. I can’t even open a Door

**I:** You hate it

**Responder:** Yes

**I:** Is that why you want to get rid of it

**Responder:** Yes

**I:** What about your social life? Don’t you like going to someone else’s home when you get stressed?

**Responder:** Yes, I won’t connect with anyone

**I:** So you’ll be happy if you get rid of it

**Responder:** Yes, very much

**I:** You told me you got the treatment

**Responder:** Yes

**I:** I mean for the seizure

**Responder:** Yes

**I:** What did they give you?

**Responder:** How

**I:** There

**Responder:** They gave me medicines

**I:** You first went to the hospital to get it treated right?

**Responder:** Yes

**I:** Then they gave you medicine

**Responder:** Yes

**I:** Are you taking the medicine till now?

**Responder:** Yes

**I:** What kind of medicine are you taking?

**Responder:** It’s small and white

**I:** Small?

**Responder:** Yes

**I:** Did you take it from the beginning or did they change it in between?

**Responder:** It’s not changed

**I:** It’s the same

**Responder:** Yes, the size was changed from small to large but the gram was the same

**I:** The gram was the same

**Responder:** Yes

**I:** When you say from the smallest to largest, is it the yellow one

**Responder:** No they didn’t give me a yellow one

**I:** It’s the white one

**Responder:** Yes white

**I:** The gram is increased

**Responder:** Yes

**I:** And then?

**Responder:** Then I took it and it got back again so now I’m taking the small one

**I:** Did they add the gram because you were getting sick a lot?

**Responder:** I don’t know how much the gram was when my father brought it.

**I:** It’s because you told me it was changed to the largest

**Responder:** But I remember them saying the gram is added. I heard them saying that they added the gram because the disease was getting worse. I was following up for some time here.

**I:** Okay

**Responder:** But I don’t know how much it was at first.

**I:** Many people go to different places when they get sick like this. You told me that your husband told you that he got better after taking the traditional medicine. And some people go to Tsebel and some to places where they get the traditional medicine. But you went to the hospital first, right?

**Responder:** Yes

**I:** Why do you think they took you to the hospital? Why didn’t they take you to get the traditional medicine?

**Responder:** I don’t know

**I:** After your father refused you didn’t use any other option. You went to the health center right?

**Responder:** Yes

**I:** Why did you choose the hospital over the traditional medicine?

**Responder:** I didn’t know anything at that time

**I:** Is this the only way you knew?

**Responder:** Yes

**I:** Is that why you came here first?

**Responder:** Yes

**I:** Many people take different options, what I wanted to say is that, why didn’t you take those options? Did you have any different reason?

**Responder:** I didn’t have any reason. I didn’t take Habesha medicine, but Tsebel.

**I:** You went to Tsebel

**Responder:** Yes I went to tsebel and baptized many times.

**I:** Have you stopped your medication or were you taking it

**Responder:** I stopped

**I:** You stopped

**Responder:** Yes

**I:** Okay, for how long have you been taking the Tsebel

**Responder:** I was taking tsebel in butajira for about four months.

**I:** In butajira?

**Responder:** Yes

**I:** Why did you stop your medication? Is it not allowed to take medicine while you are taking tsebel?

**Responder:** Yes it’s not allowed, you can’t take both

**I:** Did they say that to you or did you think that way?

**Responder:** It’s not allowed

**I:** Did they say it’s not allowed

**Responder:** Yes

**I:** Then you came back again because you haven’t seen any change

**Responder:** I haven’t seen any change so then I started taking the medicine again

**I:** What do you mean by I haven’t seen a change, were you having seizure there?

**Responder:** Yes, I used to faint while I was on medication

**I:** Were you having the stress too?

**Responder:** Yes there was.

**I:** Did you have the stress since you were thirteen?

**Responder:** No it was in between, it started recently.

**I:** Recently, like?

**Responder:** About three years ago

‘**I:** Had the tsebel no solution for it or for both?

**Responder:** I went to tsebel around the beginning

**I:** Did your family take you to tsebel?

**Responder:** Yes, my father took me. I didn’t have the stress then. It’s about eleven years since I got the disease. Then the stress came around three years ago. I didn’t go to tsebel or anywhere after that. I only take the medicine.

**I:** Have you told the doctors about the stress?

**Responder:** I haven’t told them. He didn’t even ask.

**I:** Here and butajira also? Your father used to bring it in butajira.

**Responder:** Yes, he was the one who went and brought it. I never went.

**I:** Yes

**Responder:** It’s after it was turned here.

**I:** They didn’t ask you about the stress and neither do you?

**Responder:** He doesn’t say anything to me

**I:** When you came here

**Responder:** When I come, he sometimes asks me if I have pain or symptoms. Then if I have; I’ll say yes and if I haven’t; I’ll say no. but he never talked to me about anything else, he just write and give me.

**I:** He just prescribes the medicine and you take it.

**Responder:** Yes

**I:** So you didn’t get any treatment for the stress

**Responder:** Yes

**I:** You told me that the medication you take didn’t make any difference to you

**Responder:** Yes

**I:** Why do you think that it doesn’t make any difference?

**Responder:** I am taking the medication regularly and on time. They told me not to drink alcohol and I don’t use such kind of things. I am taking the medicine regularly. I think the problem is not with the medicine, it’s just that the disease is getting worse. I don’t think the problem is the medicine.

**I:** You think the problem is not with the medicine, but the disease that’s getting worse.

**Responder:** Yes

**I:** Do you think you need to get better treatment to get better?

**Responder:** Yes

**I:** Where do you think there is

**Responder:** I don’t know that

**I:** You don’t know but you think you need to get better treatment

**Responder:** Yes

**I:** Okay, you are having a follow up in the health center

**Responder:** Yes

**I:** Are you following up in bui

**Responder:** Yes

**I:** You don’t remember the butajira. Your father used to go to bring you the medicine and you were taking it for a long time, right?

**Responder:** yes

**I:** you don’t go a lot, right?

**Responder:** sometimes I used to go

**I:** what did they say to you when you went there?

**Responder:** when I go, they tell me how to use the medicine

**I:** what about here, when you come for the follow up, is it the same as butajira?

**Responder:** Nothing to compare. Butajira is better

**I:** In what way is it better?

**Responder:** There, they will monitor what your problem is, but here; they just write and give you. I don’t even want to get the treatment here but butajira is a little far away.

**I:** They don’t ask you about your illness

**Responder:** They don’t ask anything, there was a time that I went to the manager and told him. I was sick and he just wrote the prescription and told me to go. What’s the point, if he didn’t ask me for my problem, if he doesn’t check whether the disease is getting worse or not? Anyone can write and give me. Like, sometimes he asks me if I have symptom and if there is, I say; I have and if there is not; I say I don’t and that’s it. But in butajira, every time I went, I used to go there within two months interval.

**I:** Do you come here from month to month?

**Responder:** I come here monthly.

**I:** Month by month

**Responder:** Yes, but there; whenever I went with my father when he was bringing the medicine, they do some check up on me. They used to talk to me for hours. But there’s nothing like that here.

**I:** when you come here, do they tell you about the medicine and how to use it?

**Responder:** I take one capsule a day

**I:** Did they tell you that?

**Responder:** They told me

**I:** Did they ever tell you about the side effects of the medicine

**Responder:** No they didn’t

**I:** And that you shouldn’t stop taking the drug

**Responder:** They told me

**I:** what did they say about what would happen, if you stopped?

**Responder:** They told me not to stop the medication unless they told me to because the disease might get worse. They said that it might decrease and that they’ll tell me to after they do some check up on me. But they told me that I shouldn’t stop unless they told me to.

**I:** Have you stopped while you went to tsebel

**Responder:** Yes

**I:** What about now, haven’t you ever stopped?

**Responder:** Yes

**I:** Is it while you were taking your medications that you have the seizure?

**Responder:** Yes

**I:** You said you don’t have that much change, is it also while you are taking your medication.

**Responder:** Yes

**I:** Haven’t you ever forgotten?

**Responder:** If I forget to take it one day I know I will have a seizure on the fifth day. If I stopped tonight, I don’t think I’ll have seizure today but I know I’ll have it after four days. There are times that I doubt because there are times that I could forget and take my medicine after I went out. If I stopped taking the medicine at that time, I don’t think I will have seizure then. It lasts up to four days.

**I:** But it will make you faint

**Responder:** It’s inevitable. I’ll wait knowing that I’ll have the seizure on the forth or the fifth day. But I wouldn’t have the seizure today if I stopped it now.

**I:** What about the feeling of stress, does it make a difference when you stop taking the medication and follow up?

**Responder:** It’s the same

**I:** It’s the same. Does it have no effect?

**Responder:** Yes

**I:** The other question is do you think the treatment helped you? Do you think the medication that you are taking helped you?

**Responder:** Yes if I didn’t take the medicine, I would have seizure daily

**I:** You will have the seizure daily if you didn’t take the medicine.

**Responder:** Yes

**I:** You said that the doctor didn’t ask you anything

**Responder:** Yes

**I:** But sometimes health professionals ask for your personal life as I am asking you right now, they ask questions like have you ever felt suicidal, or do you get anxious or they might ask you about your personal life, about your home, about your feeling. And do you think it’s good if they ask you like that?

**Responder:** Yes

**I:** What does it do to you? You said it’s good, right? So tell me how?

**Responder:** After I got asked, if it’s useful to me.

**I:** Do you think it’s useful for your heath?

**Responder:** Yes

**I:** Okay. You told me you have many problems. You don’t have a good relationship with your family. Sometimes you’ll get a seizure while you are working. You have stress which is making you lose interest in working and meeting people so you stay at home.

**Responder:** Yes

**I:** And in order to get rid of those problems, what do you think should be done in order to reduce them?

**Responder:** I think it will decrease if I calm down and find peace

**I:** If you calm yourself down?

**Responder:** Yes

**I:** What else can someone else do for you to make it reduce?

**Responder:** if they think of me and if they think about me with pure mind like me, if they give me love when I feel frustrated.

**I:** So if people understand when you are frustrated and if you don’t get mad. When you mean people, could it be your husband?

**Responder:** If there is someone who can understand you, if there is someone who can share your stress

**I:** There’s your husband, what about friends? Do you have any?

**Responder:** Yes

**I:** Who you can tell your secrets and who can understands you?

**Responder:** There’s no one that I can talk to that way

**I:** You just have friends

**Responder:** Yes

**I:** Do you think your stress will decrease if you have such kind of friend

**Responder:** I don’t think so

**I:** What do you think will make it reduce? What do you think should be done to make it go away?

**Responder:** I think it will go away if I find some peace

**I:** You mean peace with your husband?

**Responder:** Yes

**I:** Okay, what does your husband think about the treatment you are taking?

**Responder:** How?

**I:** What does he think about the medication you are taking? It could be your husband, or your family or your friends? Do they think it’s good and helpful?

**Responder:** Yes, he talks to me about it. He reminds me to take the medicine when I forgot taking

**I:** He reminds you?

**Responder:** Yes, he helps me when I get sick, but before that he didn’t help me. Sometimes when I get stressed he doesn’t help me but after I get sick he helps me a lot.

**I:** And does he think this treatment is good for you and encourages you to follow up or does he think it’s useless?

**Responder:** He says it’s good

**I:** He supports it and wants you to follow up

**Responder:** Yes

**I:** What about other people?

**Responder:** My family, my father; I told you earlier. And now I am just taking my medication.

**I:** Have you ever experience discrimination and stigma because of your illness or the stress?

**Responder:** No I haven’t

**I:** For example, it might be at a wedding venue, people might say ”we don’t want to sit with her” or it might be at your place of work.

**Responder:** I’ve got that once. They thought it’s transmittable. But in butajira, they told me that it’s not transmittable.

**I:** Yes

**Responder:** But a lot of people were running away from me thinking that I might pass on to them. And I couldn’t do anything. I even told them that it was not transmittable. I’ve tried so hard. I told them that it would’ve been transmitted to him since I am living with him. But there were some people who say “we don’t care, it might get transmitted”. But I know myself and I was told it’s not transmittable. But many people think that way.

**I:** But there are those who run away, thinking that you might pass on to them.

**Responder:** Yes there are some people who run away from me

**I:** And you try to tell them that it’s not transmittable

**Responder:** Yes

**I:** Okay we are finishing up. What do you think needs to be done to help those people with epilepsy, and to make their life better in all aspects like their social life, their work life, family life. What do you think needs to be done to make them live as everyone else who doesn’t have a disease or epilepsy, to improve their lives so that they can work as everyone does; and make the students learn as every other student?

**Responder:** For me to get better and go out with people

**I:** Yes, for you to get fine, and work, play and laugh like everyone else, what do you think people should do, like what do you think the society should do?

**Responder:** If the community can help us in their capacity

**I:** What do you want them to help you with?

**Responder:** For example in the time when I get the seizure or in time of need; if there is a person who I can talk to, who will tell me and guide me on what to do, if they understand me, I think they’ll share my worries a little.

**I:** Okay what should doctors do?

**Responder:** I say if the doctors ask me about my problems and if they can advise me and help me.

**I:** If they talk to you well

**Responder:** Yes, it’s really good for me, if they talk to me and if there is any alternative and if there is a solution for it.

**I:** What do you think healthcare facilities and hospitals should do?

**Responder:** What can the hospital do?

**I:** For example if there is financial problem giving the medicine for free could be one thing. What else can it do? Not just for those who suffer like you but for all.

**Responder:** As you said, there are times when I don’t have time to get the medicine and stop. They gave me for only a month when I was living in the city. They didn’t give me for more than a month. So there were times that I stopped but I pass through that. I took out a health insurance and I have talked to them so now I am getting it for free. And now thanks to God whenever I finish my medicines I went there and take it. I don’t have to worry about it now.

**I:** Because its free for you

**Responder:** I am happy

**I:** It would be helpful if it’s free for others too

**Responder:** Yes

**I:** You said you don’t have peace at home, right? What do you expect from your husband?

**Responder:** I expect peace from him too

**I:** What about your family?

**Responder:** I don’t keep in touch with my family. I don’t go there often

**I:** What do you like them to do?

**Responder:** I want them to give me peace and let me live in peace. My father said that I was not his child anymore because he regretted the money he spent for me because of my illness. I think he hates me because of that money. But you can get the money back.

**I:** So if it’s fixed, you’ll get your peace back and your stress will decrease.

**Responder:** Yes, you’ll go to your mother and your father unless you are dead and whenever I go to the village I’ll never return in peace. We fight a lot, with my father and my brothers, no one tries to think about my illness and understand me. They all blame me for everything.

**I:** What is making you stressed and what should be done to the seizure that you are having. As you told me earlier you are taking the medicine but it doesn’t make a difference, right?

**Responder:** Yes

**I:** is there anything that you think you can do to make a difference and to get better, to not get worried about whether you’re going to have a seizure or not. Is there anything that you heard someone saying “if you take this medicine or if you get this treatment, you’ll be fine?”

**Responder:** as I told you earlier they told me to take traditional medicine. They told me that I’ll never get better with this medicine.

**I:** Who said that to you?

**Responder:** My father said that, but he heard it from other people. And I was willing to go but he said I will not take you to anywhere so I didn’t go.

**I:** If you know the place now, do you think you’ll go?

**Responder:** If it doesn’t hurt, I’ll be glad.

**I:** Do you think it will make you feel better?

**Responder:** Yes, I saw a person who gets better. He was a student and he used to faint daily and he drinks it and he is fine after that.

**I:** So you want to find it

**Responder:** Yes

**I:** So have you made an attempt to try to find the place?

**Responder:** I don’t know the place

**I:** Have you asked?

**Responder:** Yes

**I:** Okay what about going to big hospital? The treatment you get here is not enough so have you ever thought about going to a bigger hospital?

**Responder:** Yes, I would be happy but there is a financial problem

**I:** Where do you think you can find such kind of treatment?

**Responder:** I think it’s in Butajira

**I:** What about higher than Butajira

**Responder:** Higher than Butajira, where I get the treatment now

**I:** Where, Addis Ababa?

**Responder:** Yes

**I:** Do you think you’ll get better if you go to Addis Ababa and get the treatment?

**Responder:** Yes

**I:** Do you think you didn’t get better because you are getting the treatment here?

**Responder:** Yes, if I was not asked whether I am sick or not, I may not know the benefits because I was given only the medication. They might add the dose or they might decrease it if it should be decreased but I think it’s because they don’t talk to me.

**I:** So you think it’s because they don’t talk to you well.

**Responder:** Yes

**I:** So you think you would’ve been better if they talked to you because they would’ve known if there is any illness besides the epilepsy like the stress, and you would’ve got the treatment for that too.

**Responder:** Yes

**I:** Okay I have finished, if there is anything that you want to add, if there is something that I didn’t ask you?

**Responder:** I have nothing to add. I just want them to talk to me well.

**I:** You would be happy if they speak to you

**Responder:** Yes

**I:** Okay thank you very much. We have finished.
