# Supplementary material for: Experience and perceptions of mental ill-health in people with epilepsy in rural Ethiopia: A qualitative study
Source: PLoS One. 2024 Dec 13;19(12):e0310542. doi: 10.1371/journal.pone.0310542 (PMC11643256; doi:10.1371/journal.pone.0310542)
Supplement: S3 File — (ZIP) [file pone.0310542.s003.zip › data set/translation 012.docx]

**Code Buipatruth0012a**

**Interviewer**: Let me start by asking some questions. Make your voice loud as much as possible and we will discuss the existing things. Your code is 0012. Let me ask you first some questions about yourself; how old are you?

**Interviewee**: I am thirty eight years old.

**Interviewer**: Your work?

**Interviewee**: I do security work.

**Interviewer**: Okay, education?

**Interviewee**: Education

**Interviewer**: Education

**Interviewee**: I completed education.

**Interviewer**: To what level?

**Interviewee**: I completed grade ten.

**Interviewer**: Up to ten grade?

**Interviewee**: Yes

**Interviewer**: Where do you live?

**Interviewee**: Here

**Interviewer**: Is it here, *Buee*?

**Interviewee**: Yes

**Interviewer**: What is your marital status?

**Interviewee**: I am married.

**Interviewer**: Are you married?

**Interviewee**: Yes

**Interviewer**: Children?

**Interviewee**: I have two children.

**Interviewer**: Okay, thank you. When I start my question, what brought you to the health center or hospital for the first time?

**Interviewee**: Thanks to God, I had epilepsy before and then started taking the medication. Now, I take the medication every month and use one pill per day. I am fine now. I take the medication every month. My brother also had such thing in recent time and then God brought us the medication, I am fine now. Two years ago, I want to try without taking the medication for one to two days but I became sick. After that, I take the medication properly and I will be given the medication from here. They give me thirty pills and I take one pill every night. It is very safe and I am comfortable.

**Interviewer**: How was it? What symptoms did you have when you first came here?

**Interviewee**: What do you mean?

**Interviewer**: What kind of symptoms?

**Interviewee**: Is that the symptom of the disease?

**Interviewer**: Yes

**Interviewee**: I didn’t know the symptom.

**Interviewer**: *Ehh*

**Interviewee**: I just don’t know when I fall; I will be conscious later.

**Interviewer**: Okay

**Interviewee**: I became sick when I am angry and don’t sleep. Now, thanks to God, I am very fine.

**Interviewer**: Okay. Did you have any other health problem beside the epilepsy?

**Interviewee**: No

**Interviewer**: Any other health problem?

**Interviewee**: I don’t have anything.

**Interviewer**: Other health problems related to mental health like depression

**Interviewee**: No, it was just the epilepsy.

**Interviewer**: *Eee*

**Interviewee**: I take medication for that.

**Interviewer**: *Eee*

**Interviewee**: I don’t have any other health problem.

**Interviewer**: Do you have anything that you say I fall when I do these things?

**Interviewee**: More, it was sleep.

**Interviewer**: Okay

**Interviewee**: It was sleep.

**Interviewer**: *Ehh*

**Interviewee**: I will be sick when I don’t fall asleep and do some works. Now, thanks to God, I am fine since I started taking the medication. I don’t miss the medication; I will take the medication after I eat my dinner.

**Interviewer**: Did you know about this disease before you were sick?

**Interviewee**: I didn’t know.

**Interviewer**: *Ehh*

**Interviewee**: I didn’t know anything.

**Interviewer**: What do you know about this disease after you became ill? What are the symptoms of the disease? Tell me, what did you know about the disease beside the symptoms you have? Do you know about epilepsy?

**Interviewee**: It is when it happens on others.

**Interviewer**: *Ehh*

**Interviewee**: Most of the time, when I saw it on some people.

**Interviewer**: *Ehh*

**Interviewee**: People will be distant.

**Interviewer**: *Ehh*

**Interviewee**: Las time a person seized at *Kela*

**Interviewer**: *Ehh*

**Interviewee**: There is teething and cutting tongue, as it also happened to me

**Interviewer**: *Eee*

**Interviewee**: When that person seized, I put pen on his mouth to don’t cut his tongue since there was time when I cut my tongue, but peoples thought that it will transmit to them ……5.42 but I don’t tolerate such type of things.

**Interviewer**: *Ehh*

**Interviewee**: I will support him when that person falls.

**Interviewer**: *Ehh*

**Interviewee**: I will support him to don’t fall on other things.

**Interviewer**: *Ehh*

**Interviewee**: Since it happened on me

**Interviewer**: What did this disease affect you?

**Interviewee**: The first thing that affected me was when I fall. I used to be a line worker on ninety seventy and I fall while I carried something and lost two teeth.

**Interviewer**: *Ehh*

**Interviewee**: I lost my front teeth. I don’t have any other thing, thanks to God.

**Interviewer**: Did it affect in relation to your work, education or social life?

**Interviewee**: It didn’t have effect.

**Interviewer**: For example, are there things like this in your life that you have not be able to do or that you have not been able to learn or that you want to do with the community because of this disease?

**Interviewee**: No. I completed my education while doing my work.

**Interviewer**: *Eee*

**Interviewee**: I don’t have any problem with my social life.

**Interviewer**: Did it not affect you?

**Interviewee**: There is nothing it affect me.

**Interviewer**: Okay. What do you think you don’t have when you compared yourself to others who done have epilepsy at this time?

**Interviewee**: I don’t have anything.

**Interviewer**: *Eee*

**Interviewee**: The discrimination hurts them and me to. I am using the medication and I am fine. I used to have a lot of friends and they were very worried about me.

**Interviewer**: *Ehh*

**Interviewee**: They tried a lot of herbal medication for me.

**Interviewer**: *Eee*

**Interviewee**: But that couldn’t be successful but I am fine after this medication came, thanks to God.

**Interviewer**: Are you fine?

**Interviewee**: I was very anxious about this disease.

**Interviewer**: *Ehh*

**Interviewee**: It what you said about mind

**Interviewer**: *Eee*

**Interviewee**: I am just fine.

**Interviewer**: *Ehh*

**Interviewee**: That means there is nothing to worry about at this time.

**Interviewer**: Which was in the past?

**Interviewee**: I had behavior of being angry in the past, as you said it before.

**Interviewer**: *Ehh*

**Interviewee**: I was embarrassed to see people leave me while I fall.

**Interviewer**: *Ehh*

**Interviewee**: There are some people who are talkative.

**Interviewer**: *Eee*

**Interviewee**: But I am fine now

**Interviewer**: *Eee*

**Interviewee**: Now I am relived from the anxiety.

**Interviewer**: Did you use anything to relieve from your anxiety?

**Interviewee**: That I used?

**Interviewer**: Did you use different methods to relieve from your anxiety?

**Interviewee**: I didn’t use anything.

**Interviewer**: That people can do when they are anxious

**Interviewee**: I didn’t use anything.

**Interviewer**: Going for treatment

**Interviewee**: I used to go for treatment.

**Interviewer**: Did you go to treatment for the treatment or anxiety?

**Interviewee**: *Eee*

**Interviewer**: Did you go to treatment for the anxiety or did you go to treatment for the epilepsy one?

**Interviewee**: At the time when I had seizure, I sometimes felt anxious.

**Interviewer**: *Ehh*

**Interviewee**: But after I started the medication, I started the medication at 2005 EC, since then I am fine since I have been taking the medication properly. The only solution is to use the medication properly.

**Interviewer**: *Ehh*

**Interviewee**: But I have no worries.

**Interviewer**: Do you use alcohol, *Khat*, or cigarette?

**Interviewee**: I don’t drink alcohol.

**Interviewer**: There are things like that.

**Interviewee**: There is nothing I will hide; I use some *Khat* due to my religion. I stopped drinking alcohol before.

**Interviewer**: *Ehh*

**Interviewee**: I didn’t use any other thing.

**Interviewer**: Did the addiction affect you?

**Interviewee**: No, there is nothing it affect me.

**Interviewer**: Okay. Why did you choose to come here? Where have you been following-up? Where did you follow-up your treatment?

**Interviewee**: Is it before?

**Interviewer**: You just started to follow-up for the epilepsy.

**Interviewee**: Here

**Interviewer**: In this hospital?

**Interviewee**: Yes, in this hospital. They came every three months …………11.26 and gave us something.

**Interviewer**: *Ehh*

**Interviewee**: I started the medication at that time. I didn’t miss the medication for once even since it was given at the hospital, you can ask them.

**Interviewer**: *Ehh*

**Interviewee**: I didn’t miss the medication in each month.

**Interviewer**: *Ehh*

**Interviewee**: I will came and bring it every time.

**Interviewer**: *Ehh*

**Interviewee**: It is for me, not for anyone.

**Interviewer**: How did you find the treatment?

**Interviewee**: It is good.

**Interviewer**: Did you come and just take the medication or did you meet the health professionals and discuss with them?

**Interviewee**: Sometimes they ask me how I am doing and did you encounter any problem. There other health professionals when *Dawit* is not here, they will ask me about everything and I will respond them.

**Interviewer**: What did you feel when they asked you?

**Interviewee**: I didn’t feel anything, I am happy.

**Interviewer**: Which you talk to them?

**Interviewee**: Yes

**Interviewer**: What do you think are the improvements you got from the treatment?

**Interviewee**: I got improvements. I think I got better improvements.

**Interviewer**: What kind of improvements?

**Interviewee**: I got improvements and I am very happy since I follow-up the treatment. My mind is also free and I am very happy at this time.

**Interviewer**: What do you think should be done to provide better treatment for people who are on treatment?

**Interviewee**: What do you mean?

**Interviewer**: What do you think the health centers or hospitals or the community should do to improve the treatment services for people with epilepsy?

**Interviewee**: I am happy if the community and hospital go to everywhere and give solution for this thing. We live at urban area but there are others who live in rural areas. We can come to the city and take the medication but advice should be given for these peoples.

**Interviewer**: Who should do this?

**Interviewee**: Patients together with doctors should do this. By the way, there are many patients and I think it is good if they go to rural areas and talk to them.

**Interviewer**: How much do you think it is beneficial? How would it benefit them?

**Interviewee**: They will understand it f I told them from my experience.

**Interviewer**: What should we expect from the community? How do you see the stigma and discrimination? Did you experience such type of things?

**Interviewee**: I am sorry, but what people do discriminate is that they will not touch a person when he seize.

**Interviewer**: *Eee*

**Interviewee**: After he is recovered from the seizure, there is strong feeling. His body will be hurt a lot and they should bring him to home or rest him somewhere but people will not be as close as they used to be.

**Interviewer**: In addition to that, for example, did you see isolation from workplace, school, and neighborhood or in the village since they have that disease? Have you ever seen anything like this that is associated with stigma and discrimination?

**Interviewee**: I didn’t see.

**Interviewer**: There are various social events, like *Edir* and *Ekub*; did you participate in such type of things?

**Interviewee**: I participate

**Interviewer**: Discrimination due to the disease

**Interviewee**: No

**Interviewer**: Are there no such type of things?

**Interviewee**: No

**Interviewer**: What do you think the community should do to help people with epilepsy? You have already told me about the health professionals.

**Interviewee**: Here

**Interviewer**: From the community

**Interviewee**: I would prefer to be able to teach people about this when they are in such difficult situation, along with the health workers.

**Interviewer**: Who? Is it to educate the community itself or is it the community provide education for others?

**Interviewee**: The community should just tell for others in the area.

**Interviewer**: *Eee*

**Interviewee**: The community expected what should be done when someone fall from you. You should do tell what they have to do and the community will help patients as much as possible if they know what do.

**Interviewer**: Okay. What you were asked when you came for the first time to this hospital for the follow-up?

**Interviewee**: When I first came, there were those who were doing in rural areas for six months by contract.

**Interviewer**: *Ehh*

**Interviewee**: There are our friends.

**Interviewer**: Yes

**Interviewee**: They worked here.

**Interviewer**: *Ehh*

**Interviewee**: The doctors came and bring us with our caregivers.

**Interviewer**: *Ehh*

**Interviewee**: They often asked the caregiver, for example, they asked my wife about me. They came every three months and asked.

**Interviewer**: *Ehh*

**Interviewee**: Then we would tell them about the situation and they also had form. They did come every three months.

**Interviewer**: When you came to this health center by yourself, it is not when they came to you; what kind of questions did they ask you when you came here for treatment?

**Interviewee**: When I came to take the medication, they asked me if there is any problem with the illness or if I am taking the medication. I take it every month, my brother has been also taking it every month since recently, and he is fine now. They also help us a lot.

**Interviewer**: *Ehh*

**Interviewee**: They told us to come back before three days of you run out of medication.

**Interviewer**: Did they say that?

**Interviewee**: They advise us.

**Interviewer**: How was their hospitality?

**Interviewee**: Their hospitality is good.

**Interviewer**: Do they ask you about your day to day life? For example, they often ask you are you taking the medication, do you seize and such type of questions.

**Interviewee**: They ask

**Interviewer**: Besides that, did they ask you questions about your life and things that may stress you?

**Interviewee**: Yes, they advise us.

**Interviewer**: What did they say?

**Interviewee**: Don’t worry; take your medication and things like this.

**Interviewer**: In addition to that, did they ask you questions in detail about things that worried you in besides your illness?

**Interviewee**: I don’t remember.

**Interviewer**: How do you feel if they ask you?

**Interviewee**: I will be happy.

**Interviewer**: What kind of improvement do you think that may happen to you if they ask like that?

**Interviewee**: Yes, a person.

**Interviewer**: From psychiatry?

**Interviewee**: If a person is given counseling, he will change.

**Interviewer**: Sometimes people are different, right? Some people don’t like it when you ask them about their personal life, some people will tell you when you ask them, and some people tell you even if you don’t ask them; there are different kinds of human behavior.

**Interviewee**: I would be happy if they ask me about my personal life.

**Interviewer**: Yes, discussions like family.

**Interviewee**: It is advice.

**Interviewer**: Asking question

**Interviewee**: It will not affect my life, it is just for me.

**Interviewer**: Do they do such type of things?

**Interviewee**: Sometimes they do like that.

**Interviewer**: What type of feeling it creates to you?

**Interviewee**: It is good.

**Interviewer**: *Ehh*

**Interviewee**: I will learn from it.

**Interviewer**: Are there any problems you face when you come here or that prevents you from coming here?

**Interviewee**: I don’t face any problem.

**Interviewer**: There could be different reasons like distance, being busy of work, being busy of family issue, and various things and are there things that prevent you?

**Interviewee**: If there is anything that prevents me

**Interviewer**: *Ehh*

**Interviewee**: There is nothing other than my life

**Interviewer**: *Ehh*

**Interviewee**: I will come and take the medication even if someone die.

**Interviewer**: *Eee*

**Interviewee**: There is nothing that prevents me.

**Interviewer**: What do your spouse, friends, or relatives around you feel about your current situation compared to the past?

**Interviewee**: What I am saying is that *Mohammed* *Nur* is in a good situation now.

**Interviewer**: How do they express your wellness?

**Interviewee**: They say he is fine after he started medication.

**Interviewer**: What else do they give comments regarding your work or family?

**Interviewee**: Yes, everyone is giving me a good comment so far.

**Interviewer**: What does the treatment looks like? Do they explain you about the medication? Do you know about the medication well or do you just do what they told you to do?

**Interviewee**: The doctor prescribed me medication. Before that, I used to take three pills of medication and it was reduced to one pill after two years.

**Interviewer**: *Ehh*

**Interviewee**: I will just take that one pill.

**Interviewer**: Do you take it?

**Interviewee**: Yes

**Interviewer**: In addition to the treatment, there is a feeling that you are embarrassed by the symptoms of the disease and the associated symptoms, as well as the embracement when you wake up; so, are there any steps you take to improve those things besides the treatment?

**Interviewee**: Me, now

**Interviewer**: Such as the life changes you have made

**Interviewee**: I have nothing to be ashamed of. This medication is very important for me; honestly, I have nothing to be ashamed of right now.

**Interviewer**: No, it is because you told me in the beginning that when you fall and wake up

**Interviewee**: It was in the past, but after I got this treatment

**Interviewer**: The treatment …..24.23

**Interviewee**: And it is also from God, there is nothing to be ashamed of. But after I started this medication, I am very fine.

**Interviewer**: What do you do to take care of yourself in addition to the medication?

**Interviewee**: I used my food and other things to take care of myself.

**Interviewer**: Are you taking better care of yourself?

**Interviewee**: I take care of myself and my family.

**Interviewer**: What do you think would be better for you?

**Interviewee**: What I think it benefits me is that being free from anything.

**Interviewer**: *Ehh*

**Interviewee**: It means the things that are beneficial.

**Interviewer**: *Ehh*

**Interviewee**: Now, for example, I told you that I chew *Khat* a little bit and my plan to withdraw it.

**Interviewer**: Okay. In some places, some people may exclude people with epilepsy, or they may not invite them by considering as ill and they may hurt their mind unintentionally; what do you think we should do to address these issues?

**Interviewee**: I never saw such type of things.

**Interviewer**: *Ehh*

**Interviewee**: I live with anyone.

**Interviewer**: What is the health problem that is worrying you at this time?

**Interviewee**: I don’t have anything to worry about.

**Interviewer**: *Ehh*

**Interviewee**: I am fine after I started the medication.

**Interviewer**: Okay. If you have anything that you want to say about the treatment which I didn’t ask you

**Interviewee**: There is nothing

**Interviewer**: Let me give you the chance

**Interviewee**: What I want to say is that this treatment is what Allah brought to me. I don’t have anything to say. And when I come here every month, they care for me and ask me about everything.

**Interviewer**: Okay, I finished. Thank you very much for taking your time for this interview.
