# Supplementary material for: Experience and perceptions of mental ill-health in people with epilepsy in rural Ethiopia: A qualitative study
Source: PLoS One. 2024 Dec 13;19(12):e0310542. doi: 10.1371/journal.pone.0310542 (PMC11643256; doi:10.1371/journal.pone.0310542)
Supplement: S3 File — (ZIP) [file pone.0310542.s003.zip › data set/translation 013.docx]

**Ruth round 2 013**

**Interviewer**: Hold it by your hand and make your voice loud. Thank you very much. Let’s start to ask some questions about you, how old are you?

**Interviewee**: Twenty six

**Interviewer**: Occupation?

**Interviewee**: Farmer

**Interviewer**: To what level did you learn?

**Interviewee**: Up to four

**Interviewer**: Where do you live?

**Interviewee**: *Chela*

**Interviewer**: Is Chela urban or rural?

**Interviewee**: Rural

**Interviewer**: Okay. Are you married?

**Interviewee**: I am not married.

**Interviewer**: Do you have children?

**Interviewee**: I don’t have.

**Interviewer**: The first question, how long have you been on this treatment?

**Interviewee**: More than ten years

**Interviewer**: Is it more than ten years?

**Interviewee**: Yes

**Interviewer**: How did you feel when you have this disease first?

**Interviewee**: I seized and there was lacrimation.

**Interviewer**: What else?

**Interviewee**: I had headache, nothing else.

**Interviewer**: What do you know about the disease in general? Do you know anything about its treatment?

**Interviewee**: I don’t understand you.

**Interviewer**: What are the symptoms of epilepsy?

**Interviewee**: I don’t know about that.

**Interviewer**: It can be from what you heard seen and from what you see from yourself in different occasions; what do you think are the symptoms?

**Interviewee**: You will be unconscious; I don’t even know myself when I fall and later someone will tell me as I was sick.

**Interviewer**: Do you have any other symptoms in addition to the epilepsy?

**Interviewee**: I don’t have.

**Interviewer**: For example, stress, depression and lack of interest in things

**Interviewee**: Sometimes there is feeling of depression, there is nothing else.

**Interviewer**: Do the epilepsy relate with these things? What is your experience?

**Interviewee**: Yes, it will come when I get angry.

**Interviewer**: Does it come when you are angry?

**Interviewee**: Yes, it comes. For example, I don’t have it now.

**Interviewer**: Did you stop taking the medication?

**Interviewee**: Yes, I stopped. We discussed with Dr. *Dawit* and I started from hundred milligrams.

**Interviewer**: Okay

**Interviewee**: Then the gram reduced, I used fifteen milligrams twice.

**Interviewer**: Okay

**Interviewee**: It means for two months and then I stooped it. It has been about three years since I stopped it.

**Interviewer**: Is it been three years since you stopped the medication?

**Interviewee**: Yes

**Interviewer**: Okay

**Interviewee**: I didn’t see anything till.

**Interviewer**: Didn’t you fall in the last three years?

**Interviewee**: Yes

**Interviewer**: Didn’t you feel anything?

**Interviewee**: Yes

**Interviewer**: Do you have follow-up even though you don’t take the medication?

**Interviewee**: It is not regularly but I will come when I am called. I don’t come regularly. Once *Dawit* told me you will be fine and told me to come back if I feel something. It have been two or three years and I didn’t feel anything, so I didn’t come for the follow-up.

**Interviewer**: Didn’t you follow-up?

**Interviewee**: Yes

**Interviewer**: Okay. Did he tell you something about the medication? How much did you take the medication before you stop taking it?

**Interviewee**: Seven years

**Interviewer**: Did you take it continuously for seven years?

**Interviewee**: Yes

**Interviewer**: Is it have been three years since you stop taking it?

**Interviewee**: Yes

**Interviewer**: Okay. Did you encounter anything because of this disease?

**Interviewee**: No

**Interviewer**: For example, having problem on work due to this disease or having problem on social life due to this disease

**Interviewee**: No

**Interviewer**: Do you something you troubled that you wish if it don’t exist at this time?

**Interviewee**: There is nothing.

**Interviewer**: Which you feel?

**Interviewee**: I don’t feel anything.

**Interviewer**: Have you been taking the medication from health professional at *Buee*?

**Interviewee**: Yes

**Interviewer**: What does your relationship with health professional at *Buee* looks like?

**Interviewee**: It is good.

**Interviewer**: Please explain it to me.

**Interviewee**: They welcome me when I come and I will not wait that much, I will go immediately. We will consult with *Dawit* and he told me to don’t discontinue and he also gave me two months medication. We did like that and I didn’t miss anything from them.

**Interviewer**: Did you proceed like that?

**Interviewee**: Yes

**Interviewer**: Sometimes there is discrimination of people with epilepsy from the community and family; what does your experience looks like?

**Interviewee**: There is nothing.

**Interviewer**: Sometimes people discriminate others because of different reasons, right?

**Interviewee**: Yes, they discriminate.

**Interviewer**: Is there such type of thing in this community?

**Interviewee**: No

**Interviewer**: That you experienced

**Interviewee**: I didn’t experience anything.

**Interviewer**: Okay, tell me about the treatment; how much the treatment was beneficial? How was the improvement from time to time?

**Interviewee**: It was good.

**Interviewer**: Now you are the level of discontinuing the medication?

**Interviewee**: Yes

**Interviewer**: Tell me the process briefly

**Interviewee**: It is good. I came and took the medication, I didn’t have something I miss and I also used it properly every time. I took it properly without missing for one day, thanks to God, I am fine now.

**Interviewer**: Did you have anything that you say it helped me more beside the medication which you did it for yourself?

**Interviewee**: Yes

**Interviewer**: For example

**Interviewee**: How was the medication?

**Interviewer**: In addition to the medication, what are the things you did which helped you more to take care of yourself?

**Interviewee**: I used to take of myself, for example, I didn’t listen to things which upset me. I will not be angry even if someone insult me, I will hurt myself I am angry and I may fall, but there is nothing else.

**Interviewer**: How much your family and people in the community are happy about your treatment?

**Interviewee**: They take care of me. They take care of me like themselves, not only my family but also my neighbors take care of me like their own children. They take care of me while I shepherd cattle on the filed like their children; they don’t have any problem.

**Interviewer**: How did you find the welcoming on the health center and hospital?

**Interviewee**: What do you mean?

**Interviewer**: How was the welcoming by health professionals starting from taking out the system card?

**Interviewee**: I will come and take out the card.

**Interviewer**: Was it time taking?

**Interviewee**: There is nothing, they will transfer my card and they will give me and I will leave. There is no problem with them.

**Interviewer**: Who bring you here for the first time?

**Interviewee**: It was my father. He brings me once and I came by myself the rest of time.

**Interviewer**: What were the questions they asked you when you came here for the first time?

**Interviewee**: How did you feel, did you fall, did you have lacrimation and how did you feel when you fall; I told them what I felt and they gave me the medication.

**Interviewer**: Did they ask you when you came in the middle or did they only gave you them medication?

**Interviewee**: They asked me as how often I seized. They asked me that as I seized every week or every two week and I told them how frequently I seized within a week or within two weeks. I told them my situation.

**Interviewer**: How many times did you come to hospital?

**Interviewee**: I don’t know.

**Interviewer**: How many years have you been since you started your follow-up here?

**Interviewee**: Seven years

**Interviewer**: Did you start here first?

**Interviewee**: I started first at *Butajira* and then I stooped since I was fine. It was not health professional who told me that but I said that I am fine. I stopped it for about seven years but it relapsed after that.

**Interviewer**: Have you ever forget to take medication?

**Interviewee**: No. Is it here?

**Interviewer**: Did you forget taking the medication while you have to take it?

**Interviewee**: I will take my medication when I travel and I didn’t miss for a day when I am at home.

**Interviewer**: Did the health professionals ask you about your personal life?

**Interviewee**: They didn’t ask me.

**Interviewer**: What do you feel if they ask you about your personal life?

**Interviewee**: I think it is good. I will explain to them if they ask me but they don’t ask.

**Interviewer**: Do you think it is important if they ask such type of questions?

**Interviewee**: Yes, it is beneficial for people, I think everything is important. I think it is good if they ask as how do I feel but they don’t ask.

**Interviewer**: Don’t they ask?

**Interviewee**: Yes

**Interviewer**: Where do you live? Do you have challenge to come here and follow-up at health center?

**Interviewee**: I don’t have any challenge.

**Interviewer**: From the perspective of time or distance

**Interviewee**: Except time and distance, I don’t have any other challenge.

**Interviewer**: Is it far?

**Interviewee**: No, it is not that much far.

**Interviewer**: What type of suggestion do your family gives about the treatment level you are at this time?

**Interviewee**: They already knew about it.

**Interviewer**: How did they find the improvement?

**Interviewee**: I also told them and when I asked my father to stop it, he just said did you consult the health professionals and then I told him as they said you will continue it if there is any problem.

**Interviewer**: Did you consult with them and stopped the medication?

**Interviewee**: With the doctor, yes.

**Interviewer**: What did you talk?

**Interviewee**: He just looked the dose of the medication that I had been taking and then he had been reducing the dose from time to time by asking me as what did I feel. Then the other doctor came and asked me and he decided as I had to stop it after taking the fifty gram dose. I also told that for *Dawit*.

**Interviewer**: What do you think should be done for people with epilepsy in order to improve their life?

**Interviewee**: They have to calm themselves and they have to take the medication properly. Now, thanks to God, I am fine. I think I am fine, thanks to God. I think we will be better if we used what the government brings to us; they also have to think like that. They have to take the medication properly like me. This is what I think and this is the important thing. They will be fine if they do what they are told.

**Interviewer**: What do you think the community should do in order to improve the life of people with epilepsy?

**Interviewee**: I think it is possible to live together by supporting each other and we will be fine too. If we look after each other, support each other and take the medication properly, I think it will be better.

**Interviewer**: What should the health profession do for the patient’s sake?

**Interviewee**: First, I think they should have to advise them in order to calm their mind. I am completely getting better now.

**Interviewer**: Do you think it will be better?

**Interviewee**: Yes

**Interviewer**: For example, what did you talk in relation to medication?

**Interviewee**: What they said about the medication is that, if I told them I will seize twice a week they will increase the dose of the medication. At that time, they told me to take it two pills during day time and one pill in the evening in order to control the disease. They increased the dose of the medication for those who fall. But if I told them the pain is decreasing, then the dose of medication will be reduced, fifty mgs will be reduced to forty and forty milligrams will be reduced to twenty five milligrams.

**Interviewer**: Have you ever missed the date of your appointment?

**Interviewee**: I never missed; I will come on my appointment day.

**Interviewer**: Okay. Is there anything you want to tell me about epilepsy disease which I didn’t ask you?

**Interviewee**: There is nothing, I was already asked.

**Interviewer**: Most of the time people become addicted to different substances to calm themselves when they are stressed; do you drink alcohol or chew *khat* or anything else or have you ever used such type of things?

**Interviewee**: I never used. I didn’t use anything, like *Khat*, since I started taking medication. Sometimes I use soft drinks but I don’t drink alcohol.

**Interviewer**: Okay, thank you very much for your time.

**Interviewee**: Thank you too.
