# Supplementary material for: Experience and perceptions of mental ill-health in people with epilepsy in rural Ethiopia: A qualitative study
Source: PLoS One. 2024 Dec 13;19(12):e0310542. doi: 10.1371/journal.pone.0310542 (PMC11643256; doi:10.1371/journal.pone.0310542)
Supplement: S3 File — (ZIP) [file pone.0310542.s003.zip › data set/Translation 018.docx]

 discontinued.

I: Ok, when did you discontinue?

R: it was last year.

I: last year? What was your last grade?

R: 6^th^ grade

I: ok, from the sixth grade. So what are you doing now?

R: I polish shoes.

I: ok, is that it?

R: yes,

I: ok do you live in the urban?

R: yes

I: do you live with your mother and father?

R: I sometimes goes to my mother.

I: aha, ok

R: I live with my father, my mother is alive, and my father is alive

I; do they live separately?

R: they are separated, so they live in their own.

I: ok they live separately, are you married?

R: I am not married.

I: ok, good. What was the main reason that you came to this health centre for the first time? What were the illnesses?

R: it is epilepsy.

I: what were the symptoms?

R: e… I fall down… I think that was the reason. I used to hang out with bad friends.

I: Aha ok

R; they were drunk men and we were not even the same in our religion. They were ….

I: yes

R: they were orthodox and I was protestant. They used to drink alcohol. They pushed and I fell down and I was broke. Because of this. When this becomes was very late….

I: what?

R: this epilepsy illness it started with feeling of fatigue……..

I: ok….

R: I started feel very tired.

I: then what happened?

R: then when it progress it becomes….. It has become worse

I: headache??

R: headache etc

I: what other symptoms does it have?

R: how?

I: it is about your illness you told me that you have fatigue, headache, what else do you have?

R: nothing

I: is there any signs of illness or any symptoms?

R: it was like that at the beginning.

I: at the beginning?

R: it was like that at the beginning

I: was it the headache that brought you to the health centre for the first time?

R: yes

I; was there fatigue?

R: felling sleepy

I: did you feel sleepy?

R: I don’t even like to wake up

I: to wake up?

R: even I am working as a shoe shining, I feel very tired and leave it to get back home

I; Ok, does that mean you become sleepy?

R: I get bored with something … get back to something

I: what?

R: it is because of the boredom that I feel very bored and then….

I: do you get bored of the work?

R: I become bored of the work and I go to ….

I: where?

R: I get so bored and I do not even like to drink coffee, I don’t have the appetite for food and I just simply sit

I: oh, this boredom?

R: sleeping on the day the time

I: what?

R: sleeping on the day time and sleeping in the night … just only that

I: Do you still have that problem?

R: not now

I: you used to have it but not now?

R: even if I had a good night sleep I just hate to wake up in the morning. I don’t used to wake up before. When I woke up…

I: what?

R: my face got swollen. Now the swelling on my face is better

I: Ok, it has decreased. Tell me about the boredom? Was it just boredom that you were feeling or was it something else?

R: boredom and to be with other people … means

I: what does that mean?

R: I did not like to be with other people. I am very happy when I am alone. Just to be alone

I: ok what else?

R: I prefer to be alone even in the church. Just to sit alone

I: why?

R: when I think about it I feel that people are discriminating me.

I: why would people discriminate you?

R: even when I was living with my family, I have to say alone. They bring me the medicine for me. I am upset because I am not able to bring it on my own. My room and my bed is on the other side and their room and the living room on one side. Why don’t I bring my own medicine? It is none of their business. I have asked him that. Patients can only be happy when they use their medicine by their own. I don’t have illness as it was before. I don’t think I have an illness now.

I: did you have seizure?

R: no I did not.

I: you did not. When was the last time you had seizure?

R: it was long time ago.

I: what does ‘my room is over there and their room’ is here mean? ‘I sit alone in front of them’ what kind of meaning does it have?

R: In the church it is not just that they prohibited me. It is me who likes to sit alone. They did not discriminate me there. I like to be alone. That is what my mind thinks.

I: ok tell me what your mind thinks?

R: my mind means?

I: what did you feel when you say that their room here and my room over there.

R: I mean it is like one house with different kinds of rooms.

I: are you upset that it was like that? About your room?

R: sometime when I am with my family tried to discriminate me. We were living in a rented house till the Kebele build for us. They said that I have to find another house in the neighbourhood. The rooms are small. The Kebele may give me my own house because I am sick and soon……. I am tired of their changing ideas… sometime stay with us … sometimes get out of the house. I just hate to live with them …. With my father.

I: so they say that to live your own life and your own house?

R: yes

I: why do they say that to you?

R: I think my step mother does not like me living with them.

I: so what do you feel when they say something like that?

R: it is not a problem, I have accepted it.

I; Ok, is there any other symptoms? Other than the one you told me, feeling fatigue, headache, and seizure. Is these the reasons that you come to the health centre? You said that you have boredom too. What else?

R: now I just don’t wake up in the morning. There is no other problem.

I: no problem now?

R: yes no problem.

I: any symptoms or feeling?

R: nothing

I: ok, did you say that you sustained an injury and broke your hand, then you started to feel tired.

R: after a while

I: after what?

R: when my hand was broke, it was fixed with the traditional healer. But after that there was something….

I: what?

R: he told me to drop it, to drop the thing…

I: what was that?

R: what was the thing? Are you the one who called Alemayehu?

I: Oh me… no when was that?

R: it was yesterday

I: yes I asked for you to come.

R: did you ask that I speak English.

I: no I did not say English. I said Amharic. I asked whether you speak good Amharic or Guragegna.

R: did he say that I speak English.

I: well I don’t remember. There is no problem as long as you speak Amharic. Ok so do you think there is relationship between the seizure and the other symptoms the headache?

R: they told me that it is genetic

I: who?

R: them

I: who are them?

R: Alemayehu

I: who is Alemayehu?

R: it is my father. He said that after I started to have the seizure he said that my grandfather had also the same seizure.

I: so?

R: but I don’t think it is genetic….. it is something that has occurred after a while.

I: so what do you think? Tell me your thoughts? What are the relationships between the symptoms? You have told me many symptoms

R: yes

I: isn’t it?

R: ok the seizure has sopped and the easy fatigability has also stopped

I: ok so do you think that there is a relationship between the two or are they different disease?

R: when I fall, I loss consciousness. I don’t know myself at the time. When I regain my consciousness, I feel very tired. That is how I know I had a seizure. When I woke up from my sleep if there is some saliva that is how I know.

I: ok that is the relationship?

R; and my face also shows. My face shows

I: how?

R: my face shows. I don’t like to go out looking like that. You will also lose your appetite. That is how it is.

I: ok. So does the symptoms have an impact … on your education or on your work?

R; yes sometimes it changed…………

I: what did it change?

R: because I fall (seizure), I discontinued my education last year. Now this year things has come up.

I; what has come up?

R: this year I was registered and I was grade eight student. But something happened

I: what happened?

R; It was not the Lord’s will.

I: so it is discontinued.

R: yes

I: so what was the main reason for discontinuation?

R: last year I fell and I discontinued.

I: were you sick?

R: yes, I was sick. And there is something

I: what happened?

R: And in the middle of the year I had seizure….they said that it is better to come back to school when you had a good health. I was only sick in the beginning. Otherwise I had a desire to learn.

I: ok, does that mean that you were not able to learn as you wanted?

R: yes

I: did the illness created any problems in your work?

R: I only work when I want and if I don’t want to work I don’t have too.

I: no problem?

R: nothing

I: I mean why don’t you work properly?
R: mine is ……something

I: what?

R: this is not a city. You just fulfil what is missing otherwise to live … it is hard.

I: what is missing?

R: everything is expensive. No one can live with his family all the time

I: what does Bue town is not a city?

R: there is no lots of people around here. When there is a lot of people you can make something

I: what?

R: even doing a shoe shining job, you can change your life. But in here you just get what is missing

I: only fulfilling what is missing.

R: yes

I: I understand. Did the illness bring any impact on your interpersonal relationship?

R: even if I am not with them I do something

I: why are you not with other people?

R: I don’t know

I: does loneliness make you happy?

R: yes

I: or do you think that people have negative perspective about yourself?

R: no that is not it.

I: or do you feel discriminated?

R; no they all love me, they love me

I: what about the things that you told me?

R: it was before. Now and before is different

I: how was it when you were sick or before?

R: because I used to be very sleep I don’t get something.

I: what?

R: I don’t go to have relation with them and they don’t get close to me.

I: they don’t get close to you?

R: I don’t have close relation with them too. In the first place I don’t enjoy it. No matter someone who wanted to be alone, it is enjoyable to sit alone.

I: enjoyable?

R: yes, now it is changed. There is nothing which cannot be changed

I: ok very good.

R: it is changed from before ……

I: is it?

R; yes

I; so what is changed?

R: I don’t have the seizure any more.

I: very good. Ok

R: I don’t feel very sleepy any more

I: very good. OK

R: I used to sleep till 10 or 11 am in the morning and they were the one ones who woke me up. And I did not even like that and it makes you feel like sleepy. After I went school I felt very dizzy. It feels like to sleep the whole day unless otherwise I got determined to wake up. It was like that.

I: ok, now all these problems have resolved?

R: Yes, I don’t have them now. Even there is time when I work. It is when you woke up in your own that you become happy.

I: you said that you were sleepy, had seizure and others. From all these problems which one of them were the most problematic?

R: the first one was to have relation with other people

I: the first one?

R: people are afraid of you. They discriminate you. It is something that I brought it on myself………….. It can come by its own.

I: yes, it is very hard ………..?

R: yes

I: when you were sick, what were the first things you did in order to get improvement?

R: what is it?

I: I mean when you were sick what kind of measurements did you take? What kind of treatments did you take?

R: I was told to go to Black lion hospital and I went there

I: what was the first thing you did? Did you go to hospital or the holy water or to a prayer or traditional places?

R: they say that to go to traditional places and I don’t something…….traditional places

I: something?

R: I hate it. In the first place I am a protestant in religion …….she even took me without my will.

I: why?

R: I don’t like holy water. I don’t like it at all……. I went to Dansha last year to get holy water

I: where did you first go?

R: how?

I: is it to the hospital or the health centre?

R: it was just changed otherwise there was no other health centre.

I: ok did you come here?

R: yes

I: ok when you come here?

R: it started around the time I come here

I: what?

R; wasn’t it two or three years since this health centre is built?

I: yes I think so

R: I went there that time.

I: you went to health centre and holy water at Dansha and ….. Isn’t it?

R: yes

I: which one helped the most?

R; it is the prayer.

I: the prayer?

R: yes

I: what did help you?

R: I was not able to stand there. I could not stand. I was trembling.

I: you trembled, then what happened

R: I was not able to stand in the hospital too.

I; ok, so I felt better. So how did the prayer help you to get better?

R: …..I fall down…..everything was as it was. They saw it and it talked. It talked how my illness was and what I did. It entered through my name. Then they prayed for me. Then they said let my name changed to Azarias. It talked all the things that my illness made me. Then we came here. And when I am at that religion …. I don’t know what was happening …..I was sick … they brought me to a witch house. I did not know myself. Then I started to be afraid, lost control and was acting like crazy. I went through many things. Even one time I don’t remember much but I only remember when I went to the forest. They told me that it is God who protected you. You would have been eaten by lion.

I; eaten by lion?

R: yes

I: so the prayer has helped you?

R: yes

I: Ok who helped you when you went to the health centre? What did they ask you?

R: what did they ask?

I: yes what did they ask you?

R: they are the ones who gave me the drugs

I: what did they ask before they gave you the drugs?

R: ….. Don’t know

I: didn’t they ask any questions when you come here? You don’t remember. Ok what did they say when you come for follow up?

R: it is when I come for follow up…

I; what did they say?

R: is it Dawit?

I yes, it is Dawit.

R: I don’t normally come. He is the one who brings the drugs for me.

I: who?

R: Alemayehu.

I: so you don’t usually come here. Why?

R: … It may be cruelty…I don’t know what it is …… it could be cruelty. I don’t know they are the ones.

I; do you want to come?

R: …. It should be yourself who should know about yourself. It should be yourself. No one can do it for you.

I: so why did they do that?

R: I don’t know.

I: ok, what kind of questions would you like to be asked when you come here?

R: like what kind of things will it make decreased? To decrease it from the previous one?....... I won’t be happy if they say it won’t be decreased.

I: the drugs?

R: they have decreased the drugs.

I: then what is it?

R: the things on me.

I: what is?

R: I mean the illness.

I: oh, you want them to say that to you.

R: Yes

I: what else do you want to know or what do you want them to ask you or what do you want them to tell you?

R: what?

I: I mean the health professionals?

R: stop that

I: to tell you that

R: yes to tell me that.

I: ok, what do you feel if they ask you about your private life or your relationships?

R; I am sure I am gone be fine…. I am sure this illness is gone away…. I will pray and I know that it will be taken away. I think it will be taken away.

I: so coming to here (health centre)?

R: just to take the drugs.

I: ok good. You don’t like to take the drugs?

R: yes

I: which part is the thing you don’t like?

R: you have to take it all the time. If you know the day that you get cured would have been good. I will wait for it with eagerness. The day that everything is cured.

I: But do you have any difficulties to take the drugs? Where do you put your drugs?

R: I don’t have a house now … so it is with them.

I: is it your father

R: yes it is with them

I: then do they give you?

R: yes, I take two or three tablets per day.

I: do you take drugs three times per day?

R: yes

I: do you take it on day time?

R: one tablet in the morning and one in the evening

I: ok, what do you know about the drug?

R: it is packed. It has a blue colour. I don’t know the name. There is no unpacked form.

I: Aha, did you say that you sometimes forget about the morning dose because you were asleep.

R: I take it when I sleep.

I: you don’t forget the evening dose too. It is just the day time one?

R: I don’t forget it.

I: what do you think is the benefit of taking the drug daily?

You don’t know? So why are you taking it?

R: yes, I am taking it…..

I; so what do you think is the benefit?

R: ……it is just they started it for me. You will never know what will happen…….

I: why?

R: if the illness is gone be cured, that would have been ok to take it without complaining…….then the illness is gone you will never know….. For those who are sick it would be better to take it every two days.

I: does that mean to decrease it?

R: yes to decrease it.

I: yes but what do you think is the benefit of taking the drug now?

R: to take it ……when you are taking it…. Is it possible that it could result in bleeding from the nose ……?

I; …. It could happen

R: when I was better and come to the hospital, there was something

I: what?

R: …… then after a long time it has come again. It happened when I was a kid and I was ok. Then it has come back again

I: what has come again?

R: the bleeding from the nose

I; the bleeding from the nose?

R: Yes

I: so does the bleeding from the nose come because the epilepsy has lasted for long time?

R; now the epilepsy is gone.

I; is it gone?

R: Yes

I: I don’t understand. What has brought the bleeding from the nose?

R: I don’t know. I am confused.

I: are you confused?

R: … Yeah…

I; so do you think there is a relation with the drug or the treatment?

R: no

I: no?

R: … when it comes…. It is connected

I: ok you prefer and would be happy to come to this hospital for follow up by your own? But you don’t usually come. Isn’t it?

R: yes

I: do you think you should come alone?

R: yes

I: your family are the reason for you not to come?

R: E…. they are not happy for me to come.

I; they are not happy?

R: Yes

I: otherwise you want to come?

R: yes I want to know for myself

I: also to tell them?

R: yes

I: that is right. So what do you feel if the health professionals ask you about your personal life?

Or do you think you should come by your own and tell them?

R: yes I think so.

I; you don’t get upset if they ask you?

R: yes

I: so what do your friends or family know about this illness treatment?

R: my friends don’t say anything.

I: how come?

R: in the first place…

I: what about your family?

R: there is something that they know. But I know that I will be cured…

I: ok what do they think?

R; what?

I: it is about your illness?

R: I think ……..

I: no I mean they take the drug for you. They tell you to take it. Isn’t it?

R: yes

I: so what do they think?

R: after the drug is changed the fatigability has improved. I don’t feel the fatigue anymore.

I: you don’t feel fatigued?

R: there is no epilepsy too. No excessive sleep.

I: no excessive sleep?

R: yes

I: Ok very good. What do you think should be done for you to continue your education or to work as you wanted?

What do you think the society should do?

R: if they can they should help like buying exercise books or pen…. If they can I would not be upset if they buy me cloth or shoes. The other thing…

I: the other thing?

R: nothing

I; there is nothing

R: yes

I: ok good. What do you think should be done so that you will have a good relation?

R: in order to have a good relation with other people, to know the reason for my illness, to know the reason for my loneliness? Why is it there?

I: you want to know?

R: yes

I; ok what do you think is the reason for your loneliness? What do you think is the cause of loneliness?

R: … for all the symptoms that I have my family should do something

I: what?

R: ….. Their love.

I: what else?

R: first of all not to isolate. It is not like before but to get close to me.

I: to have a close relation. What else do you think should be done?

R: … what do you mean when you say should be done?

I: I mean the health professionals, the hospital what can they do to improve your life or to improve all the problems that you mentioned?

R: It is not a lot…

I: yes what?

R: their income is not exactly known… and Dawit has other stuffs

I: what?

R: he serves the people alone. And if some other people could come and serve us…

I: to come here?

R: yes to come and serve is good

I: ok that is good.

R: yes

I: it means to have a lot of health professionals in the health centre?

R: additional.

I: other mental health professionals?

R: yes

I: ok good. What do you think the society should do to improve?

R: the society mean?

I: I mean the people in the town. What can they do to improve the life of people who have mental health problem?

R: discrimination is high in these people

I: ok

R: discrimination … the illness is also high. They should understand this. When this happens… it feels like leaving the drugs and go other places. When you hate it. No matter what, whether you have friends or not. No one knows what the patient has….. There is time for everything……this patient may pass away without any one knows what he has

I: that is right

R: no one knows what the patient has .the problem can be the source of the solution…

I: that is very important

R: yes

I: if there is anything that you want to add

R: no

I: ok we have finished our questions. Thank you.
