# Supplementary material for: Experience and perceptions of mental ill-health in people with epilepsy in rural Ethiopia: A qualitative study
Source: PLoS One. 2024 Dec 13;19(12):e0310542. doi: 10.1371/journal.pone.0310542 (PMC11643256; doi:10.1371/journal.pone.0310542)
Supplement: S3 File — (ZIP) [file pone.0310542.s003.zip › data set/Translation 019.docx]

**Translation interview 0019**

I:I am Dr R…..

R: I also accept

I: Ok , would you please tell me about yourself ? where were you born? your age ?

R: I was born around Olasa area, it is around BUe , I am around 50 years old, 51 or 52 I have evidence from my mother.

I: ok , are you married ? do you have children?

R: I am married. I have kids.

I: do you live with your wife?

R: yes I am living with my wife.

I: Do you live rural or urban?

R: rural

I: in the rural?

R; yes

I: ok what is your job?

R;my work farmer

I: ok, what about education?

R; I have learned up to grade 4

I;ok…..

I: ok, why did you come to the health centre for the first time?

R: I came to the health centre because I have epilepsy, I used to have treatment at Dukuman

I: what were the symptoms?

R: the symptoms were … I fall down, then I chew a lot, I mean I loss my consciousness. It started when I was a kid ..used to live with my mother and sisters…I think it started around 1986 EC. My older sister was sick and I cried a lot . I do not know whether I have the disease before that but after that I have it.. That is how it started. Then my sister died , I loved her so much, the way she died was also very sad , she died suddenly, I cried a lot . at the time I cried till I loss my consciousness. Then I g started to get sick that night , I lost my consciousness, I was bitting my self they told me this. Then progressively I started to fall and have epilepsy

I:aha, what other symptoms were there?

R: other , my body is no more present, my body has become weak, I feel like 80, 100 years old man. Like a very old man, my body is all dead, weak. Now I just cannot run, I cannot even keep my hand straight, it becomes floppy, has become very weak. I don’t know whether it is from the way I took the drugs or whether it is from the illness… for example I had a fight with my cousins and they have hit him on the head , my head was hurt very bad. I don’t know whether it is from the wound or my ear drum was damaged , I was very sick for 4 or 5 years. I had a treatment at Butajera….

Now I am waddling like a drunk guy. This has also become a problem. The main thing is my body has become weak. I cannot describe this illness. It has made me not to work. I just cannot stay still, I feel dizzy. I don’t know how it is related with the illness or other, I can not say this.

I:ok, do you have any other feeling or any health problem?

R: no

I: no problem?

R; yes

I: that is the only problem?

R: yes

I: your sleep appetite…

R: yes I do sleep, even though I don’t have much sleep now , I used to sleep during the night time. I had a good sleep before. Now days I have a problem to get back to sleep. if I wake up in the middle of the night. If I woke up at 6 (mid night) I usually got back to sleep after an hour or around 5 am like early in the morning. Otherwise I don’t sleep in between or if I wake up in the middle of the night.

I: your sleep does not come back. Does this problem lasted for long?

R: it is recent. It did not last long

I: what about you appetite?

R: I eat a decent food. I only eat delicious food. Starting from the beginning I only eat delicious food. She always make delicious food for me. But it is difficult to get good food in the rural areas. It is very tiresome. If I eat little food from the morning till the night, I get grumpy. Even if I am used to the modern food , I am gone eat whether I like it or not.

I: but do you have any other feeling?

R: I don’t have any other feeling. I get angry because it is not always enough. As you see the e children, they don’t obey your command as you want them too. As long as you don’t get the things you wanted there is always a feeling. In this area, if you want to do something that you really wanted or working by yourself with full health, you cannot get what you wanted. It is only when you are healthy that you can get what you wanted with confidence. If this is not there your feeling is gone get hurt. This is also a problem… that is what I wanted to say. I cannot follow as I wanted. I have a lot of influence.

I: so do you think that all these symptoms are related?

R: you mean?

I: the tiredness… you told me you get easily tired, there is also an epilepsy, you are angry

R: may be the angry feeling. From the health center, Butajera and Dukuman, I have learned that and they believe that I should not be angry, I have to relax, I need to take care of myself. If all that is related, it you the health professionals that knows. I know partially that getting angry can harm at anytime, you need to calm down but it is not as you wanted. As long as you don’t get what you wanted , there will be harm. You can not avoid to think, to get worries. During this time , everything is expensive, kids have a lot of needs. You have to buy shoes, cloths . for example children usually loss their pen everyday. That is also a problem. Every night when they will say they have lost their pen… that is also a big problem. It would have been good if you are able to full fill their needs but you can not. They have to use what they have and live their life. But you can not full fill everything and you have too feel angry. It is not we like to be angry.

I : it must be it?

R: yes it is. If everything was facilitated, I would have lived a free life. You have a lot options for yourself and for your family.

I : what are the options ?

R: there is no options . God only put the options. I cannot say there is one because I cannot do that with my income. And as you know human beings need….

I: what are the obstacles not to get this income?

R: because the place that I was born and raised give me headache even I worked hard. Even if you have full body , if all parts of your body is working, if someone is fully heathy , then God is with him. I swear to God, may God forgive me even daily labourer get 150 birr per day of they are fully healthy. If they are able to save , if they don’t drink, if they don’t move around, if they are not extravagant, they can change their life. If you are healthy, you are able to work whatever you like and change your life or mind with God’s willing. But not everyone is the same, some people are careless, some people work hard, the others live in pride, the other people live in moderation. How can you all do this? It is only if you are healthy, energy and mental health. If your mind is working properly, you can be changed. If I talk about myself , I plan to do some activities, but after a while, after doing something or a talk, I forget all about it. On the next day I think about doing this then the other, but after doing one thing I forget about the other. I wish I could even memorize (save ) this but I forget.

I; what are the other effects on your life? For example in your relationship with other people or social life

R: yes, as I said the social life is brought by you or you can bring other people‘s problem in your life. This is because I don’t violate the other people’s right and I don’t want anyone to violate my right. Therefore , people tend to push me , call me crazy. That is a big impact on my life, it hurts a lot and I could not bear it. This is one of the reasons that I fight with my cousins. They left me almost dead but God has saved me. It is after that I got married and have kids. I don’t judge even if I was dead but they almost killed me. This is also violation of the rights of others.

I: does the violation of rights comes because of your illness or not?

R: no, it is not.

I; No ? so what was the reason?

R: it is because they don’t care about the rights of others. People are like that.

I: aha, so there is no relation with the illness?

R: No, I don’t think so.

I: ok, what are the other impacts of this illness?

R: the other impact that I see is I am not able to work as I wanted. Because I don’t have energy and you need a lot energy to go where ever you want to go. But no energy, my power is not able to work

I:what about group work?

R: group work? I could not even do my own job let alone a group work. I am not even able to work and move by my own. I only does limited work then…

I: then what happens?

R: then my energy becomes dead

I: is this recent event?

R; it was before

I: is this recent event?

R: no , before I was married , before this illness was seen, I used to work a lot. I mean I was also a clever student, I even passed double grades till the grade 7. Then I stayed in Addis Ababa and continued night time school for a while.

I: why did you discontinue?

R: it was not convenient and I was also a kid. I used to live with my relatives and everything can not be comfortable. So I left and get back to my home where it is rural. To learn in the rural area I did not have the potential…

I; do you think that the society has impact on me because of your illness?

R; No, I don’t think my family has an influence/ impact on me. But there are other people in the society who tries to discriminate me because of my illness. I mean in our area.

I: what did they do? For example …

R: they will say like someone has epilepsy, don’t have a bright mind, or he is crazy. Both you and me there is nothing we can do. You can not be in other persons shoe and judge them. This is what I say regarding the illness.

I: do they invite you to participate in group activities?

R: yes, partially. There is nothing they do in public but they do it secretly.

I: secretly?

R; yes, in secret. People have told me that I was not invited because I have epilepsy. what people talk behind my back like in EDER..

I: EDER like funerals, wedding or etc?

R: yes especially around EDER

I: Around EDER?

R: yes around EDER, people say that I should not be invited because I may not attend properly or disturb etc..

I: you have told me that you feel weak and tired, have also epilepsy and you have difficulty of taking care of yourself. all of them are not easy. Which one has the greatest influence in your life \/

R: the main thing is the epilepsy. as I said before it has gotten worse after I got married.

I did it get worse?

R: Yes, the epilepsy started before I got married. It got worse after I got married, there is a lot of work. My body got weak ( fatigued).

I: did the fatigue comes ?

R: Yes , there is fatigue. I feel very tired.

I: does the epilepsy still there?

R; yes , my last seizure was 8 months back.

I: your last seizure.

R: if I especially work bending down, ploughing a corn field while bending makes me sick.

I : to be sick what?

R; I mean the seizure. That is when I have a seizure.

I : what else is also precipitating it?

R: it is just the corn field. I don’t even know myself, it was other people who told me. There was one guy who came running for me. He even had a lot of influence from the other people but he came for me ….as I told you before.

I: is this the guy whom you had a fight?

R: yes , he told him. Then he picked me up. At one time I was also weeding and weeding then I had a seizure. There is nothing I can do.

I : is there anything which you think is the precipitating factor or problem?

R: from the way I understood it… not taking care of yourself, to get anxious , not relaxing yourself. Regarding myself when I work too much, when there is too much tiredness it can be precipitated. That is my guess. But working hard is mandatory. Let alone when you are sick person , even if you are healthy if something bad happens to you, you will be hurt. That is my guess for myself. And when you have lost your appetite , when you don’t get the food that you want at the right moment, all these have an impact. Especially if I don’t eat at the right time, my whole body get weak becomes dead. Then I don’t eat properly because the time has already passed. That is what I belief for the precipitating cause. First, not eating food at the right time, getting anxious. Well we cannot decrease that it is a must. As I said before as long as if you don’t full filled your needs, there is always gone be worry. In order to decrease your worry, you have too get what you need. Everything should be at your hand. If you don’t have money, you can not full fill your needs. So the main reason are not eating at the right time, inconvenient situations, working too hard and too much worry.

I: OK good, so what have you done to improve the epilepsy

R: what can I do. What can you think should be done?

I: what was the initial response?

R: nothing. There is no solution that u can do and expect. Because it is a must. What you think as a solution could be examining yourself, trying hard not to get into anxiety even if you don’t get what you want. Full filling 50 % or 25%of your need means there is something that keeps you relaxed.

I: what I mean is when this illness happened what have you done to solve it?

R: oh, the solution is to go to a hospital. I did not know what to do at the time. You have to do something after you have relation with people. She told me to go to Dukuman. Then we went to Butajera

I: what did they ask you when you went there?

R: they have asked a lot but I don’t remember

I: was it long time ago?

R:it was long time ago. I don’t remember.

I: ok, how come you went to hospital? Did you go to holy water, or traditional medicine etc

R: yes , I always try holy water. But it is not convenient because I don’t wake up early in the morning. If I wake up early, I feel like I have been working very hard job, I will be tired. So I wake up after the sunrises, like an old man after the sun has fully rose, I will wake up. It is around 8: 00 am.

I: so about the treatment area for epilepsy, how did your mom knows about it?

R: I think she got the evidence from other people.

I: evidence ?

R: yes, I think she knows how it manifested. She knows it even from the old times. Even before I was married, one of my cousins had epilepsy. When I see it for the first time I was shocked. I have never seen such a thing. We were walking and playing with him, then he fell down, he chewed his tongue and drooled saliva. Then we went to his uncles in Shakiso and they said that he had the illness before. Then we got back to Bue. That is what I heard and seen about epilepsy. Then I started to have it. I was not aware what to do or how it manifested, I did not even know that I have it. I think she got the evidence from that, I had seizure two or three times before, as I told you before the first one was when my sister died. And then I had it when I was eating …. Then she took me to the hospital. Ther was also another person in our area

I:ok , good. How did you get the treatment ?

R: the treatment was good. I stayed without it for long time.

I: after the treatment?

R: yes, after the treatment I stayed free for long period of time . they even told me that I need an investigation

I: where was that?

R: it was DUKUman . I did not take the investigation. Then after a while I had an operation on my ears. Then it was approximately 8 months back in the month of May when I was ploughing the corn field I had a seizure, which was present as before

I: was it 8months back?

R: yes

I: where is your follow up now?

R: the follow up started in Butajera.

I; ok,

R: yes, now I got transferred to here and took the drugs for 2 months

I: when you come for follow up , what do they ask you?

R: do you have seizure? What are the changes from before?

I: ok they ask this?
R; yes

I: what else do they ask you?

R: there is nothing they ask.

I:ok, what do you ask them?

R: me, what is the solution? As I told you before I am not working properly. What is the solution for this?

R: what is their answer for this?

I: like decrease to work, eat food, yes that is what they say. But how can I decrease work, I don’t work extra work, it is just the farming, I don’t even harvest. Even I was supposed to take care of the cattle, I can not do that. when I was keeping the cattles in turns, I will sleep in the middle of it

I: aha, the fatigue

R: Yes, I feel fatigued. And I got bored. There is nothing you do, you just have to keep an eye on the oxen, the sheep, the goats. If we are not doing the harvest,.. that is why

I: how was the health professionals treating you?

R: oh , they were good. When I had a follow up in Butajera, there was a guy who worked at the card place and I thought he was sicker than me. But he was such honest and determined guy. I am ashamed of myself, I considered myself as healthy and him as a sick person. I should accept with patience of what God gave me (my potential).

I; I understand.

R: even now days he is angry with me. It’s amazing. Sometimes you have to swallow your anger. There is one guy and one female working there. You have to treat well with advice. When one of my children lost the tablets they did not treat us well. They may even have a respect for apes than us, they did not even respect us. I had this experience while I was taking from Butajera. I was observant of this.

I: is it while you were taking from there?

R: while I was using. Then he started to give me this drug and he even once brought the drug to my home and visit me. Whenever I come here he always give me education/ advice like decrease to work, take care of yourself, relax and decrease your anxiety.

I; Did they ask you about your feeling, or personal life?

R: yes, they ask how your living situation is and I will answer as I told you before.

I; they ask your living situation?

R; yes

I; so what do you feel about that?

R: yes they ask about that

I: they ask?

R: yes they do

I: so what do you feel when they ask you such kind of questions?

R; I am happy because I feel like they share my problem and I expect that they will bring a solution. I am also happy about this interview because people are following us from Addis Ababa. The chance of getting cure for our illness is high. If you make such a follow up that is one hope. This is one thing. I have waited here for long time. Even after I came here your phone was not working. I tried to call two or three times at least to get introduced to you. Because I was treated here many times but I did not get a cure. but your phone was not working. Then I thought about taking my drugs since I am left with two or three tablets. On the way, I went to the chart room. Then you called me again and I come back. This is also a good chance, that is what I wanted to say. When you ask me all this questions, I expect many things because I have a chance for cure. There is one guy( name) in our area who is cured from this illness, I don’t know how is it by prayer or with medical treatment. When I talk about myself, my children are working and I am afraid that it might be inherited to my children. There is one baby girl who is almost a year old and she was sick. Then she was taken to a traditional healer and got better. She took the traditional drug and become fine.

I: is she your child?

R: yes, that is my child. And one of my children , he is the second child he urinates while on a sleep. As I told you before, I am worried that their mind is not working properly. Even I am worried than them, because I have a suspicion that this illness is inherited to them and they are gone suffer. Currently she is doing fine but what if this illness might get hidden and will get worse later in her life. The boy is eight years old and he like sleep and he urinates at night and we are hoping he will get better. We tried but no improvement.

I: ok. We will talk later about this. What do you know about the drugs that is given to you?

R: yes , I always take one tablet at night time

I: one tablet?

R: yes, it was the same before and now

I:do you take in the morning?

R: not in the morning

I: do you ever forget to take the drug?

R: no, I sometimes forget. It is not that I am forgetful. I don’t know forgetting. When you are living in traditional society, there is my aunt, my sisters and my brothers. When there is wedding there is some alcohol and they told me that the drug contradicts with alcohol. But I am not dependent on alcohol. When people are living together, you have to use some alcohol. I discontinue and get worried. Whenever I am not taking alcohol I take my drugs but not with alcohol. This is a must

I: but did it ever cause any obstacles for your follow up or to come to this hospital?

R: yes it did but it is mandatory. even when I used to follow in Butajera I had to pay for the medication, for the treatment. It is mandatory isn’t it for my health? To improve myself or to get my family out of this. It is a must to keep my health.

I : ok good. What do you think should be done to improve the overall life of the affected people?

R: well if the government can… for me and other people like me… well thanks to God. But I see myself as disabled. If you are not able to work, you are like blind, or handicapped or deaf, then it means you are disabled. That is how I see it. If I am not able to work as I wanted or as long as I am disabled or not the same as other people, it is not convenient. For example difficult to travel as I wanted. There are also people who have got this illness and careless, who does not follow regularly or who drinks a lot. Even I am not the same as before, may God give more time… if I am out of this, I will be a person who struggles to change himself.

I: what do you think how the health professionals struggle?

R: I think the health professionals should cover the medication cost, help in getting things needed in the home. At least help in getting the important foods. There are many stuffs and now days the cost of life is very expensive. It is difficult. It is even very expensive to buy one jacket or a shoe…it is very expensive. You can’t run your life in debt. You have to fix this in order to live.

I: yes, it is very expensive

R : you cannot leave all this the government .some rich people should help.

I: what do you think the society should do?

R: the society is the same as I said before. If the society should help in any comfortable way. As I said before for those who are disabled, if the society has good attitude, compassion, serving, that is the most important thing that is expected from everybody. For example those charity worker their main job is giving charity. Doing good for anyone is good in both God’s eye and in other people. By bringing this quality someone life can be changed. The government means me and you.

I: yes the people

R; some people have money they deserve. They have said where they can take it or use it. It is good if they work in such kind of things

I: what about health facilities like health centre, hospitals?

R: well health facilities are by itself another compound and we understand many stuffs. Let me tell you what happened once, it was about the service, the medical service, I went to the chief. I went there to talk to him. And he was sitting with other woman like you. He did not give any attention to me. Even though I am just ordinary patient, he should have called me and said that he has no place or cannot help you. He is the main manager, there was no harm if he said ‘ how can I help you?’., ‘take your drug or something’. I don’t know whether he is still in the same position or not. But I have observed that. There is nothing I can do or I cannot prosecute him. First of all he is a government official, second I don’t have any power. I left there. It was about health insurance. The expiration date has reached and I told them let me bring the health insurance paper from my kebele. In the mean time I asked them to give me some drugs. They said that they could not do that. It is both for the treatment in the chart room and at the drug store. Then I left them and returned back after I renewed my health insurance card. It is mine now. For the question what the health facility do ... as I said before like a charity… if they have good attitude with good conscious value, they could help and treat us. Because when a patient comes here he comes with a lot of problems and with a expectation that the health professional will cure me … below the will of GOD. In that case if they help with their potential, I believe that it I good.

I: till you get health insurance card what did you do about the drug?

R: I don’t remember . it was long time ago.

I: is it long time ago?

R: yes, it has been over two years.

I: Ok, good, do you have anything to tell me. You have given me a lot of information. If you think that there is something left unsaid , please tell me.

R:there is nothing left. It is good. I have told you what I remember. It is good , it is always good to give attention to the patients. There might be some mistakes from the patient side but it is the responsibility of the institution. As long as you are in a professional job, this is mandatory. You cannot act and be part of those who are ignorant even if you are with ignorant people.

I: that is right

R: I think with all the knowledge you have, all your potential and faith you have, it is good to share that. It is good to have patience.

I: ok thank you very much!!!
