# Supplementary material for: Experience and perceptions of mental ill-health in people with epilepsy in rural Ethiopia: A qualitative study
Source: PLoS One. 2024 Dec 13;19(12):e0310542. doi: 10.1371/journal.pone.0310542 (PMC11643256; doi:10.1371/journal.pone.0310542)
Supplement: S3 File — (ZIP) [file pone.0310542.s003.zip › data set/translation 02.docx]

## 130615-002

**Interviewer**: Okay, thank you very much for being volunteer and take your time to come here. As I told you, we came from Addis Ababa University and our discussion will be about epilepsy disease and co-existing mental health disorders. What was your problem when you came to health center at the first time?

**Interviewee**: When I seize I came here for investigation. After that, I didn’t have improvement when I had been following-up here, due to that I was referred to *Butajira*. While I was following-up there, they referred me back to here and then I am following-up here.

**Interviewer**: When did you have the illness?

**Interviewee**: The illness, about eight years.

**Interviewer**: Is it?

**Interviewee**: Yes, it has been eight years but I didn’t have any improvement by the medication I take. Sometimes, I will have headache when I seize. I didn’t seize but I will have headache and my hand jerks a little bit and drop materials. I will seize when I am depressed and the medication doesn’t have improvement. The medication doesn’t have effect even though it was changed many times. But, now I have been given medication and I am fine.

**Interviewer**: Are you fine now?

**Interviewee**: Yes

**Interviewer**: Did you go to health institutions when the disease started you eight years ago or what?

**Interviewee**: I went to *Butajira* when I was sick.

**Interviewer**: No, did you try any other treatment option before you went to *Butajira*?

**Interviewee**: We didn’t try.

**Interviewer**: For example, it could be traditional healer, holy water and Protestants will go to prayer’ did your family try such type of options?

**Interviewee**: They didn’t try; they just took me to *Butajira* hospital.

**Interviewer**: Did you go to health institution?

**Interviewee**: Yes

**Interviewer**: Why do you think they choose that? Some people do that after they tried other options.

**Interviewee**: Yes. When I said that, when it begins me my cousin had graduation and I was carrying pot to kitchen with my cousin and they were shocked when I fall. And they just worried as what it is and all of them said took her to hospital and they will give her medication. They said she will get worse if she stays and I have been also getting worse. I was getting worse, they took me immediately to *Butajira* and from *Butajira* I was sent back to here.

**Interviewer**: Why did they send you to here?

**Interviewee**: They said it is better if we referred you than you come here.

**Interviewer**: To don’t go there?

**Interviewee**: Yes, they said we will prescribe you medication to their rather than you come to here.

**Interviewer**: But you went back to *Butajira* since you didn’t have any improvement here?

**Interviewee**: Yes, they took me there when I didn’t have any improvement. It was good there (the follow up) and was happy. I even thought it may not be epilepsy. I mean my hand bleeds a lot without any reason and I have headache, I will have burning sensation on my head and I will vomit like a baby. At this time when my hands bleed, I will feel free and I don’t seize. I will drop things and I will seize sometimes when I am depressed.

**Interviewer**: What stress you most?

**Interviewee**: Yes, I will be simply depressed. When I will be depressed it will goes to my head, it doesn’t like depression. I was told the disease didn’t like depression and then I will try to make my mind free but I can’t, at that time I will seize and I will be hurt a lot. Last time my medication changed and I am fine after taking it. I am a little bit better now.

**Interviewer**: When you say I don’t have improvement and I am very sick, is it the epilepsy or the burning sensation?

**Interviewee**: When I am sick it means I am gone seize. It shows me a sigh when it is going to seize me. When I am going to seize it show me signs, I will depressed and then lost interest for everything. After that, I will know myself and sit down or I will be suddenly sick.

**Interviewer**: So when you say I don’t have improvement, what did you see and go to *Butajira*?

**Interviewee**: How?

**Interviewer**: You told me as you didn’t have any improvement, then you went to *Butajira* and came back here, right?

**Interviewee**: Yes

**Interviewer**: How did you see it, is it since you seize? How did you measure your improvement?

**Interviewee**: As I told you about my improvement, I will be relieved when my hands bleed. I feel my head burning and I will be relieved when my hands bleed. It is not he medication but I will be relieved when my hand bleeds.

**Interviewer**: Do you feel better?

**Interviewee**: Yes, I feel better, it means rather than the medication

**Interviewer**: Rather than the medication?

**Interviewee**: When my hand bleeds, I feel better.

**Interviewer**: Okay, I understand what you are saying. My question is, you are still saying you are sick, right?

**Interviewee**: Yes

**Interviewer**: What do you say is your illness? Is there still the seizure frequently?

**Interviewee**: In the past, I seize every month but when my family worried, I came and asked *Dawit* and he had be changing medication for me.

**Interviewer**: Is it since you seize every month?

**Interviewee**: Yes

**Interviewer**: Was it once a month?

**Interviewee**: Yes, once every month. He was confused and said I have to send to other place and then my mother came and asked him. I had been taking two pills at the morning and three pills at the night but these medications didn’t improve me.

**Interviewer**: Is it similar medication with different dose?

**Interviewee**: It made it itself three times, it was twice before that I took. I took two pills in the morning and two pills in the evening but now I am taking two pills in the morning and three pills in the evening.

**Interviewer**: Do you take the same type of medication in the morning and evening?

**Interviewee**: No, I take big and small size medications. The one I take at evening is small and the one I take in the morning is small.

**Interviewer**: Is it different? Are there two types?

**Interviewee**: Yes

**Interviewer**: Do you remember the color?

**Interviewee**: It is white.

**Interviewer**: Are both white?

**Interviewee**: Yes both are white, the one is small and the other is big.

**Interviewer**: Is the white sell in plastic? Do you count and take it?

**Interviewee**: Yes

**Interviewer**: What about the other one?

**Interviewee**: The other one is also like that.

**Interviewer**: Is it given to you in plastic? Do you count and take it?

**Interviewee**: Yes

**Interviewer**: Did you start taking the big one recently or earlier?

**Interviewee**: It is recently, I will not be two months since it is changed.

**Interviewer**: Do you take that in the morning?

**Interviewee**: I take two pills from the big one in the morning and three pills from the small one in the evening. I saw a little improvement after taking that.

**Interviewer**: Did you see improvement?

**Interviewee**: Yes

**Interviewer**: When you said I saw improvement, for example, what is it?

**Interviewee**: I don’t have seizure and I don’t drop things since I started this medication. I have seen a little improvement by this medication.

**Interviewer**: Okay, I understand you. I just want to know your improvement when you said so. Is it just that the seizure reduced?

**Interviewee**: Yes, it is reduced.

**Interviewer**: What were the symptoms of your illness? You told me seizure and burning sensation at your head

**Interviewee**: Yes

**Interviewer**: You told me your hand bleed and drop things. What other symptoms did you have?

**Interviewee**: From the past illness?

**Interviewer**: Yes

**Interviewee**: I have gastritis and I can’t take meal, and then I will be very sick.

**Interviewer**: What else? What about depression?

**Interviewee**: Depression, yes. Sometimes I can’t also be able to take rest even though I tried relaxing myself and then I will be depressed and become sick when unexpected things come to my mind.

**Interviewer**: Do you think the depression related to your epilepsy?

**Interviewee**: I don’t think so, I mean the thing I told you it will show me symptom, I will be depressed, lost interest for everything and don’t want to talk when I am going to be sick.

**Interviewer**: You told me that. Do you have any other depression?

**Interviewee**: Yes, I have depression.

**Interviewer**: Do you have?

**Interviewee**: Yes

**Interviewer**: Does the depression last for long time?

**Interviewee**: I may be depressed the whole day.

**Interviewer**: Have you ever been depressed for fifteen days?

**Interviewee**: No, I was depressed for three or two days, but it never last longer. The depression comes occasionally without realizing it.

**Interviewer**: It means, for example, how many times a month it happens?

**Interviewee**: About five times.

**Interviewer**: You will be fine one week and depressed the other week

**Interviewee**: Yes

**Interviewer**: When some people encountered depression they may have intention to commit suicide, right? Have you ever felt like that?

**Interviewee**: I don’t think like that, it means when I lost something and that thing depressed me, I will feel better when I cry.

**Interviewer**: Does it make you cry?

**Interviewee**: Yes, it is like that but it I don’t think to commit suicide. It never came to my mind, I just cried and it just be disappeared.

**Interviewer**: Okay. Do you have difficulty of falling asleep when you are depressed?

**Interviewee**: Yes, there is occasionally. Sometimes I can’t fall asleep.

**Interviewer**: Is there a loss of appetite?

**Interviewee**: Yes

**Interviewer**: Okay. What about feeling of crying?

**Interviewee**: Yes, there is.

**Interviewer**: Do you have like that?

**Interviewee**: Yes

**Interviewer**: These are the symptoms of depression. There are such types of symptoms, such as insomnia, loss of appetite, and feeling of crying, and there is epilepsy on the other hand, it means the seizure. Which one is difficult for you?

**Interviewee**: What do you mean?

**Interviewer**: Which one is difficult? Is the depression or the epilepsy difficult?

**Interviewee**: The epilepsy is severe. The epilepsy is severe than the depression for me.

**Interviewer**: Why do you think it is severe?

**Interviewee**: I mean, when I think about it in my life, what I was doing before is a mistake. I wonder how I ever saw such type of thing and something like this happen in my life and I also felt to that level.

**Interviewer**: Okay. This disease has different effects on your life and is there anything you have experienced?

**Interviewee**: What do you meant?

**Interviewer**: For example, if something happens to you in your education because of your illness since of you have this disease

**Interviewee**: Yes, there is.

**Interviewer**: What, tell me?

**Interviewee**: That means I don’t learn much because of this disease because sometimes when it makes me depressed while studying at school, and at that time it hurts me too.

**Interviewer**: So, did it affect your education?

**Interviewee**: Yes

**Interviewer**: What about on your social life? Did it influence on your social life with your friends and influence you to go to church?

**Interviewee**: It didn’t.

**Interviewer**: Do your friend know you have this disease?

**Interviewee**: Yes

**Interviewer**: Do your family know?

**Interviewee**: They don’t know.

**Interviewer**: Is there discrimination and stigma because of the disease?

**Interviewee**: No

**Interviewer**: Is there no such type of thing? Don’t you experience anything?

**Interviewee**: Yes

**Interviewer**: Do you think that people may discriminate me because of my disease and is there a thing of not joining other people?

**Interviewee**: I think like that. My friends rumor that she have the disease. And again I try not to go too far because I fear I may be sick.

**Interviewer**: Since you have the fear?

**Interviewee**: Yes, so I don’t go.

**Interviewer**: That means you don’t go too far.

**Interviewee**: Yes

**Interviewer**: What about working at home?

**Interviewee**: I didn’t work at home; they didn’t let me to do work.

**Interviewer**: Will you be tired?

**Interviewee**: I will not be tired but they thought I will be sick.

**Interviewer**: Did you say they will not give me?

**Interviewee**: Yes, my family lives at rural area and I live here, I tried to go there and when I went there I will not work since they think I will be sick while working.

**Interviewer**: What do you feel when they don’t let you work?

**Interviewer**: I will feel since how long I will stay without working id I don’t do at this time. As I may go somewhere else to work so I struggle and work.

**Interviewer**: Does that means you work for yourself?

**Interviewee**: No, when I go there.

**Interviewer**: Do you say I will work?

**Interviewee**: For example, my mother used to bring me food and I will be very upset since when she bring food at the time when I can prepare.

**Interviewer**: So, didn’t you try to say don’t bring food for me I can prepare?

**Interviewee**: I tried.

**Interviewer**: Don’t they agree?

**Interviewee**: They don’t agree; they will be happy if I don’t work.

**Interviewer**: Okay, now there are your peers, right?

**Interviewee**: Yes

**Interviewer**: Do you think there is problem when you compare yourself with them; it could be on work, social life or education? Do you think you are different from other people?

**Interviewee**: I feel when my friend works as they want and I work with fear. I think I could work like them.

**Interviewer**: Okay. Are you different on your social life?

**Interviewee**: No

**Interviewer**: Do you go to mourning like your other friends? Do you do what all of your peers do?

**Interviewee**: Yes

**Interviewer**: Are you not the same at work?

**Interviewee**: Yes

**Interviewer**: You think you are not similar at work. What about on school?

**Interviewee**: We will learn.

**Interviewer**: Do you learn?

**Interviewee**: Yes

**Interviewer**: What about on education? You told me it started eight years ago, right?

**Interviewee**: Yes

**Interviewer**: Are the result of your education eight years ago different from your education result eight years later?

**Interviewee**: Very different.

**Interviewer**: Did it affect you?

**Interviewee**: Yes, I told you before, because I hate education when I am sick. I don’t know why I say I don’t want to go to school. I will be stressed when I entered into the class. I didn’t hear when they talk and teach. I used to have good results but I am losing now since I will be absent many times.

**Interviewer**: Will you be absent because of that?

**Interviewee**: Yes

**Interviewer**: You tell me as there are different reasons such as the epilepsy and there is also depression; if you are said to lose one of these, what do you want to lose?

**Interviewee**: The epilepsy

**Interviewer**: Do you think you can handle that one?

**Interviewee**: Yes

**Interviewer**: Or do you think the other will be gone if you don’t have the epilepsy?

**Interviewee**: The depression?

**Interviewer**: Yes

**Interviewee**: If that one disappears, the depression will also disappear as it is related with it. They are two different types; sometimes people will be depressed that is real. But if the epilepsy is cured, I don’t have the problem with the depression.

**Interviewer**: So, do you wish the epilepsy to be cured?

**Interviewee**: Yes

**Interviewer**: Okay. The other thing is that as you told me as this disease influenced you a lot and you told me that the first measure you took was going to health center immediately and got treatment.

**Interviewee**: Yes

**Interviewer**: How did you find the treatment?

**Interviewee**: When I went to *Butajira*?

**Interviewer**: Yes

**Interviewee**: The doctors asked my symptoms and they told them as I seized. My family didn’t understand the disease, they thought I seized because of depression or something else. When they explained as I fall to them, they said it is epilepsy and immediately gave me medication. Beyond that, my families and neighbors told him that it is epilepsy and you should bring her there and they will give her medication.

**Interviewer**: Did they say that?

**Interviewee**: Yes

**Interviewer**: Did the neighbors guess?

**Interviewee**: Yes, as I told you before it was my cousin graduation when I seize and at that time many people saw that and they said it is epilepsy and took her to hospital immediately. Then he took me and I was examined. I was given medication immediately and I wasn’t improved even though I followed-up the treatment. But after that, I became fine but it relapsed in the middle. Then, I decided for myself that i don’t have epilepsy. I thought if it is epilepsy why not it is improved by the medication. After a while, my hand became red and I just touched it. I went to Alert hospital, Addis Ababa last year due to that. Because of that, I also stopped my education. I was told by the doctors that I had nothing. I will have headache when my hand bleeds and it is confirmed that I have epilepsy when I seized.

**Interviewer**: As what it is?

**Interviewee**: It is confirmed that it is epilepsy.

**Interviewer**: What it is called in your community? Is it called epilepsy?

**Interviewee**: “*Azurit*”

**Interviewer**: Is it called “*Azurit*”?

**Interviewee**: Yes

**Interviewer**: Okay. Is depression considered as a disease in your community? Do you consider the depression as a disease?

**Interviewee**: When I am depressed?

**Interviewer**: Yes. You told me as you are depressed, anxious and want to cry, right?

**Interviewee**: Yes

**Interviewer**: Do you consider that as a disease?

**Interviewee**: No, when I think about things it makes me cry. What makes me cry is this disease. If I don’t have this disease, I will go to Addis Ababa and be with someone. I will think about that and it makes me to cry.

**Interviewer**: Can’t you be with someone because of the disease?

**Interviewee**: I can, but I don’t want to because I need rest. Now, for example, I think I may be unconscious if I go somewhere else. Since I get up in the morning and eat food to take my medication, in the middle of it, the other people who don’t know about me may not help me, and I will be upset when I think about that. I will say I can be as I wish if I don’t have this disease and that make me feel bad.

**Interviewer**: You told me that the depression and epilepsy influenced your life a lot.

**Interviewee**: Yes

**Interviewer**: What about the depression on your education and work?

**Interviewee**: Sometimes I wondered why I get depressed when I am.

**Interviewer**: Did you be absent from school when you are depressed?

**Interviewee**: I didn’t be absent. There is nothing but I will be depressed. Now the epilepsy is known and I will be depressed sometimes. When I am sick, it means I will be suffer from a disease other than the epilepsy.

**Interviewer**: Do you have gastritis or what?

**Interviewee**: Yes, I will have gastritis and other illness when I am depressed.

**Interviewer**: Is that when you are depressed?

**Interviewee**: Yes

**Interviewer**: So, what is affecting your life is the epilepsy rather than the depression?

**Interviewee**: Yes

**Interviewer**: Okay, let’s talk about the treatment you took. We talked a lot of things in the beginning, right?

**Interviewee**: Yes

**Interviewer**: Did they tell you well about the medication when you come to this health center? How it is when you come here?

**Interviewee**: The doctor could not be able to follow-up me well but he advised me several times to don’t be stressed before. But I don’t know, I try to be happy when I am with my friends but I can’t; I didn’t do that intentionally. Then he tells my family that she is just worrying about simple things. Now, when I come to take my medication as he doesn’t respond well, so I will be afraid and say I have nothing. I will tell him when it became severe. They changed my medication when I am very sick. I told him I am sick and my mother also told him to change the medication and we will go to other place if he don’t change it, then he changed the medication. Then he told her as he didn’t know about my stress, and she told me what he told her. The medication has been changed for me and I am taking the medication.

**Interviewer**: Didn’t he prescribe you medication for the depression?

**Interviewee**: He didn’t prescribe.

**Interviewer**: Did he give you the two for the epilepsy?

**Interviewee**: Yes

**Interviewer**: Okay. Have you ever forgotten to take medication?

**Interviewee**: I will not forget.

**Interviewer**: Do you always take it?

**Interviewee**: Yes

**Interviewer**: The medication doesn’t show you improvement, but you never forget to take it

**Interviewee**: It was in the past. I forget to take it before sometimes, but I don’t forget at this time. There is no problem at night, I will stay from one o’clock to four o’clock and will take it and at the morning, I will wake up at one o’clock and take it.

**Interviewer**: Okay. Tell me what you know about the medication?

**Interviewee**: How?

**Interviewer**: What do you know about the medication? What did they tell you?

**Interviewee**: They didn’t tell me anything.

**Interviewer**: Did they tell you how to take it?

**Interviewee**: Yes, they just told me how to take it, nothing more.

**Interviewer**: No. Didn’t they advise you things to do and don’t while taking medication?

**Interviewee**: They advise is just too don’t be stressed, to don’t do anything stressful and to don’t overwork.

**Interviewer**: Is that what they said?

**Interviewee**: Yes

**Interviewer**: Okay. What about as you don’t have to discontinue taking the medication?

**Interviewee**: They advise me to don’t discontinue taking the medication.

**Interviewer**: Do they advise you?

**Interviewee**: Yes

**Interviewer**: Do they tell you that alcohol should not be taken while taking medication?

**Interviewee**: They don’t tell me.

**Interviewer**: Didn’t they tell you since you don’t take it?

**Interviewee**: I don’t take.

**Interviewer**: Is that the reason they don’t tell you?

**Interviewee**: *Dawit* told me at the beginning.

**Interviewer**: Did he tell you?

**Interviewee**: Yes, he told me at the beginning.

**Interviewer**: Okay, in general, how did you find the treatment? Do you think it is good to take the treatment given here or do you think why it does not make any difference to you?

**Interviewee**: I think it is good because if I didn’t take this medication I may not be alive. It helped me even though I become sick once a month or a year; I have seen a lot of improvement. If I didn’t change the medication I will be sick. But I think it is good if I go somewhere else. It is good for me to take the medication.

**Interviewer**: What do your family think about the treatment?

**Interviewee**: They think the treatment is good.

**Interviewer**: D they encourage you to take it?

**Interviewee**: Yes. They are worried about me and they support me, but they didn’t discourage me. They will also come here.

**Interviewer**: Why do you live here being separated from your family?

**Interviewee**: I mean, I have been at compassion since my childhood and I have been learning here as it is not possible at rural area.

**Interviewer**: Is it for you to learn in the urban?

**Interviewee**: Yes, the compassion refused for us to learn at rural area and they said as it is possible to learn at urban.

**Interviewer**: What is compassion?

**Interviewee**: Is there no compassion at Addis Ababa?

**Interviewer**: I don’t know.

**Interviewee**: They aid cloth, bag and book.

**Interviewer**: Can’t that be while being with your family? And it is possible if you are, maybe is that why you came here?

**Interviewee**: They told me as it was impossible if it is in rural area since they have been registering when I was a child. Then I just start to live here.

**Interviewer**: Okay, good. Do the health professionals ask you about your personal life when you go to health institutions? They may ask you not only about your illness but also about your personal life, and how would you feel if they asked you about that question such as sleeping too much and suicidal ideation? And how would you feel if they asked you that question?

**Interviewee**: Personal life?

**Interviewer**: Yes, if they asked you when you go for treatment.

**Interviewee**: I will be happy.

**Interviewer**: Okay. What do you think is the importance of asking about personal life?

**Interviewee**: I think it is good to be asked because I would be happy to explain them about my personal life.

**Interviewer**: What would be the benefit for you if you explained that for him?

**Interviewee**: They will encourage me, they tell me what to do when I tell them, and they will tell me what to take.

**Interviewer**: Okay. What about if they ask you about your feeling, for example, there are questions about depression and thought of self-harm?

**Interviewee**: If they asked me about that, I don’t know.

**Interviewer**: Do you think you would be offended if you were asked?

**Interviewee**: I will not be.

**Interviewer**: Do you think it will be beneficial?

**Interviewee**: Yes, I think it will be beneficial.

**Interviewer**: Why do you think it is beneficial?

**Interviewee**: I get advice from then when I am depressed. I think they let me know what I don’t know. I will not do anything when I am depressed. I think some people go into something else when they are depressed, and I think I will get some advice from them.

**Interviewer**: Okay. The other thing is that, some people do not go to a health center when they are sick, so why do you think they will go to other place, like traditional healers, when they are sick?

**Interviewee**: I don’t know that.

**Interviewer**: When they encounter disease like you. You directly came to the health institutions, right? Other people don’t come to the health institutions, they go somewhere else.

**Interviewee**: Yes

**Interviewer**: Why do you think they are going somewhere else?

**Interviewee**: As I told you before, my follow-up was at *Butajira*. They need good doctors and nurses.

**Interviewer**: Yes

**Interviewee**: For example, I would not be beneficial if I follow-up here. As I told you before, I was sick and I was going to somewhere else by the thought if there are other doctors found who are better than these doctors.

**Interviewer**: I will go if there are better doctors than these

**Interviewee**: Yes

**Interviewer**: By what criteria? For example, what do you think you will find elsewhere that you didn’t find here?

**Interviewee**: That means, even though most of them are equal, some of them have knowledge gap and simply prescribe. But at the same time, there are some who know and examined well and prescribe medication.

**Interviewer**: What do you think? Do you think you have been diagnosed well, or do you think you should be examined again?

**Interviewee**: I think I should go somewhere else and be examined.

**Interviewer**: Do you think you disease not been diagnosed?

**Interviewee**: Yes

**Interviewer**: Do you think like that since you don’t have that much improvement, or don’t you rely by the doctors here?

**Interviewee**: Yes, if there are better than these.

**Interviewer**: Don’t they ask you much or don’t they examine you well?

**Interviewee**: Yes, they just prescribe medication.

**Interviewer**: But, don’t they ask you well?

**Interviewee**: Yes, if you go to somewhere else, there are those who ask and examine well. Beyond that, medication should not be prescribed without examining; I think like that.

**Interviewer**: Okay, good. The other thing is that there are many people who have epilepsy, right?

**Interviewee**: Yes

**Interviewer**: And these people, for example, there are some people who can’t work, some people who don’t participate on social life like *Edir*, mourning and wedding. There are those who don’t go to weddings, those who don’t wear their clothes, and what do you think should be done to make these people life better than anyone else? For example, you have this disease and your other friend has this disease and what should be done to make your life better than the life of your friends?

**Interviewee**: Did you say what should be given?

**Interviewer**: Yes

**Interviewee**: For example, taking them to health institutions by collaborating. If nothing can be done at health institution, they can be taken to somewhere else.

**Interviewer**: When you say collaborating, is that the community or who?

**Interviewee**: The communities, for example, collaborate and take them

**Interviewer**: Taking them to treatment?

**Interviewee**: For treatment. Those who have support for others who don’t have

**Interviewer**: Okay. What about those who are taking medication, are they fine? For example are there people who are as healthy as you? What else should be done to improve their lives more? When I said they are fine, I mean their seizure is reduced. You told me they have to be taken to the health institutions to reduce their seizure, right? What should be done to make them work and to participate them in social life like their friends?

**Interviewee**: As I told you, I don’t think they will not be better if they get treatment. It will be better, if I take me as an example, if I go somewhere else and get treatment, I think I can work. If those are treated like that

**Interviewer**: Do you mean if they get better treatment?

**Interviewee**: If they get treatment and make themselves free.

**Interviewer**: Okay. Now you are telling me if the communities do this, right?

**Interviewee**: Yes

**Interviewer**: What do you think the government should do?

**Interviewee**: I think if the government does what it can.

**Interviewer**: For example, when I said to improve their live it is not only money aid but there are different things.

**Interviewee**: If the government wants a sick person not to die, it should do what have to be done. Or I think supporting; it may not be money, as you said it before, if they share their knowledge. I think that will help them.

**Interviewer**: What type of knowledge should they share? For example, what type of knowledge did they share you?

**Interviewee**: As I told you before, I have depression, right?

**Interviewer**: Yes

**Interviewee**: I don’t know about depression, I don’t know what worries me. If the government gives me better advice, I would not be depressed.

**Interviewer**: Do you mean the health professionals? They are the health professionals who will give advice, right?

**Interviewee**: Yes

**Interviewer**: What do you say that should be done at health center?

**Interviewee**: Advice

**Interviewer**: No, it is not. There is advice that is given at the health center. Do you have something that you say it would be good for our lives if the health did that?

**Interviewee**: I told you before, if the advise us.

**Interviewer**: you told me if they advise us, I mean if you have anything else to say. Regarding the medication, do you always get medication?

**Interviewee**: Yes

**Interviewer**: Do you always get it?

**Interviewee**: I do.

**Interviewer**: Okay, what else? The other thing is, do you always come to health center by your appointment?

**Interviewee**: Yes

**Interviewer**: Always?

**Interviewee**: When I run out of medication, I come in a month.

**Interviewer**: Have you ever been absent or missed one or two day?

**Interviewee**: I have never been absent.

**Interviewer**: Don’t you?

**Interviewee**: Yes

**Interviewer**: But some people will be absent, what do you think could be their reason? There are people who tell their reason at their appointment.

**Interviewee**: I think they may forget it because of their illness.

**Interviewer**: Forget?

**Interviewee**: Yes

**Interviewer**: If there is another problem that could forbid from coming to the health center.

**Interviewee**: They may not like it.

**Interviewer**: Taking the medication?

**Interviewee**: Yes, I am like that but I will come every month, I don’t discontinue.

**Interviewer**: But sometimes, do you want to discontinue it? Don’t you like it?

**Interviewee**: Yes, I don’t like, but I will take it. Even food is not taken like that and I am taking like food and I hate taking it. You may want to commit suicide and you may forget and don’t come due to the disease.

**Interviewer**: Why do you hate it? Is it since you took it for long time?

**Interviewee**: Yes

**Interviewer**: That is why you hate it; you told me as you take it as food, right?

**Interviewee**: Yes, I have used it for eight years.

**Interviewer**: Do you hate if for a long time or do you have another reason?

**Interviewee**: It has been eight years. I am always stressed when I was thinking about taking the medication.

**Interviewer**: Did taking it for long time and when will I stop stress you?

**Interviewee**: Yes, when I will stop this medication and when I will be said it is enough from hospital worried me.

**Interviewer**: Another question I forgot is; have you ever seize at school?

**Interviewee**: I seize last year once at school.

**Interviewer**: Did you seize?

**Interviewee**: Yes

**Interviewer**: What did the students say?

**Interviewee**: By chance I was sick while we were going out from school. My friends were there when I seized. They knew and helped me. There were some peoples who didn’t know about me and I sat down. Then a teacher came and hug me, she saw me and called my family. Then my mother came.

**Interviewer**: Did you seize in front of the students?

**Interviewee**: The students laughed and one of them who didn’t know shocked. The students laughed and left me. At that time, I hated myself since I thought I would never be like that if I don’t have this.

**Interviewer**: Did you experience anything after that from students?

**Interviewee**: No

**Interviewer**: Nothing, things like insult

**Interviewee**: Nothing

**Interviewer**: Nothing?

**Interviewee**: Yes

**Interviewer**: Okay. Have you ever seize at bad place? Have you ever been hurt?

**Interviewee**: Yes, my nose a little bit

**Interviewer**: Is it lacerated?

**Interviewee**: It was lacerated. It was lacerated. I was going to church with my cousins and I didn’t know when I fall and it hurts me. Then when my father saw me, he said is this Marta and at that time I was unconscious and sat down while my face covered with cloth. Then, my father said this is my daughter and he cried. That is when I was so hurt. My face was lacerated and I came to hospital and they treated me with alcohol and it became fine. I thanked God because I didn’t think it would be fine, this is the only sign left. I just remembered that time when I saw this.

**Interviewer**: Have your family ever been discriminated because of you?

**Interviewee**: No

**Interviewer**: Have your family ever been upset because of you? Did they day we spend a lot of money because of you?

**Interviewee**: They didn’t say that because they want me to be healthy. They just said stay with us because they don’t spend money too.

**Interviewer**: There is aid, right?

**Interviewee**: They don’t spend money because it is covered by health insurance.

**Interviewer**: No, before that. The health insurance came recently, right?

**Interviewee**: Before that, they were sad since I was sick and even before they were like that.

**Interviewer**: Do you live here alone?

**Interviewee**: Now my brother lives here. He is grade nine students and I live with him.

**Interviewer**: Is there a worry of she may seize at night time?

**Interviewee**: Yes, there is. They will be worried if I go out. They protect my mind and they don’t want me troubled.

**Interviewer**: Okay. I finished my questions, if you have something you want to say or if you have something to add

**Interviewee**: No

**Interviewer**: Okay, thank you!
