# Supplementary material for: Experience and perceptions of mental ill-health in people with epilepsy in rural Ethiopia: A qualitative study
Source: PLoS One. 2024 Dec 13;19(12):e0310542. doi: 10.1371/journal.pone.0310542 (PMC11643256; doi:10.1371/journal.pone.0310542)
Supplement: S3 File — (ZIP) [file pone.0310542.s003.zip › data set/Translation 020 AM.docx]

ID- AM 0020

Place: Bue Hospital

Interviewer – Ruth

Time 43:05 minutes

I: My name is Dr R. Now I will ask you about epilepsy and the comorbid illness. The interview will take approximately one hour. I would like to thank you for agreeing to be interviewed.

R:I am willing

I : Please tell me about yourself.

R: what does it mean when you say tell me?

I: it means tell me about your life, what is your name?

R: AM

I: your age

R: I don’t remember what my age is. It could be recorded in the chart

I: what is your job? What is your level of education?

R: I am not educated. I did not get education even when I was a kid. Isnt it this because my health was …… I have no education. I spent the day at home. I cannot do anything.

I: are you a house wife?

R:Yes

I:No I mean…

R:Yes

I: I think you have kids?

R:yes

I:you are a house wife and you take care of your kids ?

R: Yes

I: where do you live rural or urban?

R: in the urban

I: in the urban?

R: Yes

I: ok. So what was the reason that brings you to the health centre for the first time?

R: this is the case. I used to take the drug from Butajera

I: why is that?

R: for epilepsy

I: what was the signs of epilepsy?

R: if I get ill, I don’t know anything but people know. It started after I got married.

I: Oh…it started

R: Yes

I: when was that?

R: it started 18 years back from now on.

I: what was the signs when it started for the first time?

R: It starts from my heart. Then I don’t know how it make me. After I fell down I don’t know anything. Then I get back to myself. It get me to somewhere and get me back to myself.

I: what does it mean “to get me somewhere and get back to myself”?

R: It is just an anxiety on my heart. An anxiety on my heart. Even now I sometimes get sick even though I am taking the drug. I get sick. I feel anxious in my heart

I: what does anxiety mean? Tell me about that.

R: what do I know? God knows it for me. I don’t know anything. God knows. Difficult to express. It is just…. It starts from my heart. Sometimes I get better. After 2 weeks it starts again. I take drug both in the morning and evening. I take 120 tablets per month.

I: one month?

R: I took it monthly, it won’t be discontinued

I: we will talk about that later. First you lose consciousness?

R: yes

I: oh... don’t know where you fall?

R: I don’t know anything

I: What does people say about your symptoms? When you get ill what do they tell you about the symptoms?

R: people say that it is epilepsy. That is why I started the treatment. People asked me how did I get it. I was also asked how it has made me in here. I told them simply. My anxiety from my heart did not get better even if I am taking the drugs

I: would you please tell me very well what does “I feel anxious at my heart mean

R: I don’t know anything. I feel anxious at my heart. I don’t know anything. It is after it passed I know people. It is like an animal… anxiety on my heart is the only anxiety I feel. ( she showed difficulty to express her feeling). But I never discontinued the drugs both at day and night

I : what other feeling do you have?

R: I don’t have any other things. Just the anxiety. I don’t have any other problem.

I: when you say anxiety, is there any other associated symptoms?

R: I don’t have any other feeling. Just the anxiety on my heart. The anxiety on my heart I don’t know what it is.

I: therefore, does the anxiety comes together with the epilepsy… every 15 days or do you feel anxious alone? How many times do you feel anxious? Please tell me about that?

R: sometimes 15 days or it may even last this much… it may starts every three days. This anxiety may starts every 3 days or sometimes every 7 days. When I tell him the guy about this anxiety, he told me that I have to go to other doctors for investigations, I told me that I don’t have any place to go. It is just an anxiety which needs medication. This anxiety on my heart. Only God knows. When he he keep on telling me that I should go. Then I started to tell him that I am ok. I feel peace I said to him. When he asked me to whether I have seizure or not, I just tell him that everything is good ( at peace). God only knows for me.

I: therefore, do you think that the anxiety that you feel and the epilepsy has relationship or are they different?

R: I don’t have any thoughts

I: no thoughts?

R: I don’t have any thoughts of worry. No thoughts. Just the anxiety… the anxiety on my heart. Only God could know

I; so does the epilepsy make you to lose consciousness?

R: Yes

I: Ok, does other people say you have other symptoms?

R: yes

I: do you feel anxious? and people say you have epilepsy?

R: yes

I: do these two have any relation?

R: I don’t know anything.

I: do you think that you have only anxiety?

R: first I feel anxious and something…. Then people know what happened next

I:please make this feeling clear for me

R ( husband) : first she cries, then she feels anxious , then she talks, talks and talks. Then she has drooling of saliva and her body… the drug has helped a lot.

I: ok lets hear what she says , first you feel anxious then you feel fear?

R: yes

I: do you cry?

R: yes

I: what other?

R: I see something. From the place where I sit I see something.

I; something else?

R: yes, I see something else. It changes my house. I just see something else.

I :ok ?

R: even I feel like I am seeing my children as other things

I;Ok?

R: after I had seizure. I don’t know where was I. “where did I go and back again? “ I asked my children if they are around me. It is something like a jungle that my heart shows me.

I: yes: does this mean that you see objects which are not real?

R: Yes

I: is there any other symptoms that you feel?

R: No, I don’t feel any other additional thing.

I: Is there any other health problem?

R: I don’t have any other problem. I just hate it. There is banging on my head. It is like a side way banging on my head.

I: what does banging on the head mean? please tell me.

R: yes it bangs my head. I feel anxious. It divides my head and there is banging

I: how many hours does it stay?

R: after a while it will go away. Sometimes it stays longer. The drug was good especially the packed one. After the packed one was available it was good. I used to take two kinds of tablets and they helped a lot.

R husband: she got sick because the packed drug was not available

R : it is available

R husband: didn’t you say that it was not available.

R: it is available

I: Ok you told me that you feel anxious and you have epilepsy> Is that right?

R: yes

I : what is the impact that this symptoms have n your life.

R: the things that brought it on me? I don’t know/

I: when I say ‘impact’ what effect does it have on your life ? For example on your work or on your life. That is what I mean

R: effect? I don’t know

I: impact mean effect

R what does impact mean?

R husband: it means a challenge

I: yes, yes, impact means effect on your life . for example did it make you not to work. That what I mean

R: yes very much. It has made me not to do my work at home

I: tell me about that

R: I used to leave the house work

I: OK

R: if I get ill , I don’t do any of the house work.

I; what kind of work ? for example

R: like baking injera

I: oh, house chores

R: yes

I: you are not able to work if you have seizure ?

R; yes I don’t do any house work because of my sickness.

I:OK

R: even I will drop the things that I hold with my hand

I : o. what about your social life? Like your relationship with your neighbour, with your husband and with your children?

R:I don’t have any problem. I am at peace

I: no problem?

R: the only problem is the sickness. No other problem.

I: does this problem bring any impact/ effect? Any challenge on your social life example ‘eder’ that is what I mean.

R: yes I have

I: do you have “eder”?

R:yes

I : do you attend weddings, funerals?

R: yes, I do

I: every activities?

R: yes

I: do you go to funeral places?

R: Yes

I: Do you go?

R: Yes, Yes

I: how is your relationship with your husband? With your family/ with your neighbours? your social life?

R: I don’t understand this ‘social life ‘

I: I mean your participation

R: you mean ‘ I spent the day with discussion’. My life is good. My husband take care of me. My husband is also worried about me

I: so do you think that the illness has caused a challenge in your life? In your social life which means to attend funerals to be part of ‘Eder’ to drink coffee with your neighbours? Did the illness has stopped you to do this activities? That is what I mean

R husband: if she goes to the neighbours for coffee she will get sick. She is also thinks that she will get sick if she go out for coffee. I also know this problem, the neighbours know it , therefore …

I : so this means she is unable to drink coffee with the neighbours as she wanted?

R: Yes

I: do you understand what I was asking?

R: yes

I: social life means when you are grieving people come to you, you also go when other people are grieving. But because of your illness you have difficulties to do this activities/? That is what it mean?

R husband: she was sick previously. So they said not to come again not to go out of her house. They (the neighbours) said they won’t be upset if she does not come. They said that please do not bring her to funerals or Eder what if something happens to her. All the women knows her situation and they come , and they don’t want her to come. It is good not to be in a bad place

I: yes, so it means it has stopped her to go to the neighbours isn’t it ?

R: Yes

I: is there any other things that the illness has created?

R: there is no other problem… because I am sick even if I was at home I don’t go to Eder places, I don’t go to weddings , I don’t go places where people talk and discussed. It does not allow it

I What will happen if you go?

R: I will have a relapse I will feel anxious in my heart

I: is there any other place where you feel anxious?

R: there place where people are grieving and crying, it always make me feel something. I don’t always go there

I: how is your family involved in your day to day activities? Like going to the market, house chores?

R: I stay at home. This is other people country. My family are in the country side. The thing that you said about the market. I usually go to the market and do my business quickly and go back to home before I get sick in my heart.

I: so do your family help you to do these activities? How much do you your husband and children help each other?

R: my children and my family live in the rural area. I don’t do any work. He is the only one who knows my anxiety. He is the only one.

I: how does he help you by worrying?

R: he knows about my anxiety and he is .. everything for the house.

I: what does “he is everything “ mean

R: he was the one who helped me before

I: your house mean

R: the thing I do at home he is the one. I don’t have any work. House wife mean to work on the things that is given to you. Otherwise I don’t have any work.

I: ok what other kind of work do you?

R: If I am not sick I work.

I: what do you do?

R: I only work at home nothing else

I: so what kind of work do you do at home?

R: if there is something to bake I do that every three or four days. But if I get sick I leave it.

I: so do you do the baking

R : yes

I: what about your children?

R: my children do what they can

I: what do you do for them?

R: I don’t do anything>

I : there is many stuffs to do at home for kids, isn’t it?

R: there is only baking injera, what other I can do?

I: so you are saying that the illness has influenced me , when the illness start there is an impact on your work , on your social life. You were not able to attend weddings or go to a market, it has also an impact on your house chores.

R: Yes

I: isn’t it?

R: yes

I: what have you done to cure this illness?

R: it is the drugs

I: is there any place you went before the drugs?

R: yes we went together to a physician at kebelle called Goro. There is place called Hawasa we also went there. I have went to all places where there is holy water

I: what else?

R: there is nothing else

I: did you go to holy water for the anxiety or for the epilepsy/

R: I was taking the drugs at home. If the drugs are delayed , the epilepsy…, I went to the holy water place for the anxiety because I thought it is something else

I: did you go to holy water because you thought the anxiety is another disease?

R: yes

I : why did you choose the holy water?

R: because even if I am taking the drugs the anxiety is on my head day and night. If I don’t stop the drugs something is not gone relapse. They say to get to a holy water rather than the hospital, so I went to Hawasa , to Goro, to Addis Ababa Shunkuru holy water. When I was in Hawasa I spent the night with anxiety. I went to Gabriel holy water with my brother. When I was at the holy water, there was nothing found in me. The monk told me to sit there. The other people were exorcised from the evil spirit. The monk told me to sit there and blessed me with something .. .you know. The he said there is a spell on the devil and that is why it is not talking ( getting exorcised). It is a spell on the devil. Even if she is going to a holy water place, it won’t talk. It is a spell on the devil. they have also put a magic on her while she was in her father’s house. Even when I am going to holy water it the spell, magic made it difficult to get it exorcised. It is because of the spell that her anxiety started. Before the epilepsy started it is the spell. Her spirit has talked.

I; what happened next?

R: what can I do. I just kept saying that may God reveal everything. Then my brother said may St Gabriel reveal everything what can we do. She also has epilepsy. the man said that she also has an evil eye there is a magic and spell put on her. What can I do, my God reveal to her said my brother. She just needs to take these drugs and one day it might be all revealed. It is the anxiety that comes first.

I; wat kind of changes did you get after the holy water

R there is nothing when I was getting baptized. It does not make me talk, it did not reveal anything, then I changed my religion. They said that I will be fine if I change my religion. After two years the anxiety was still not cured. So I said I wont stay like this, then I started to go a church and I follow there. With the drugs my god knows it all. I have no skills. All the wisdom is with God

I ; When did you go to Butajera, the health centre for the first time

R:Dukuman

I: who helped you at the Dukuman ? how did you get it ?

R: they were very good

I :the health professional who helped you

R: when I started the drug I was not married

I: when was that?

R: after I started the drug and after I had the illness that I get married. After a year I used to take the drug every month, there was no the drug which was sealed, I took the other drugs.

I: what kind of questions were they asking you?

R: do you mean when I come to take the drug?

I: yes, when you come for follow up or to take the drugs what do they ask you?

R: yes, he asked how many times do I have epilepsy. when I told him that I have anxiety in my heart, he told me that I should go to other places, I will refer you to other places.

I: who said that?

R: Daniel

I who is Daniel?

R; a doctor called Daniel

I; Aha a doctor.

R: it is from him that I take the drug. This card is taken to him every month and he will give me… something

I: why did he say that you are going to other place?

R: I just told this to you. I didn’t tell him that I still have the anxiety. I asked that is there any other drug or something different for it. He said that the only drug that is available is this one. if I still have the illness when I am taking the drug, I will refer you to other places. When he said that I don’t want to go anywhere, so I told him that am ok, just give me the drug.

I: so do you come here every month?

R:yes

I: what kind of drugs are you given?

R; it is 120 drugs

I: one hundred twenty?

R: yes

I: do you always follow the drug instruction?

R: yes every morning and at night

I:every morning and at night ?

R:yes

I: did you ever forget?

R; yes I have never forgotten about it. Even I am going away from home I will take it with my bag

I: won’t it be lost?

R:no it won’t get lost

I: why do you follow it strictly?

R: because I am hoping that I will be fine.

I: ok

R: I am hoping that my anxiety will get better. What can I say

I: what changes have you seen with the drugs?

R: the drug is very good. There is something…

I: what has the drug helped you? I mean how did the drug help you?

R: there is some something changed. I used to be very anxious before. Now days it is sometimes… what will happen if the drug is not here. Both of them the anxiety in my heart would not disappear.

I : what do you mean both?

R: the illness

I: so how did you find the treatment at the health center?

R; going over there?

I: over there or here?

R: when I go there they told me it is available at BUe. Then I started from here

I: how did you find it?

R: I told them and it is on my card . they give me from the card

I: so what does the health professional asks you?

R: who is he?

I: the doctor over here. You told me that when you come for follow up they ask you about your health? What more questions he asks?

R: there is nothing he asks. He asks How do you feel now? Is there any epilepsy now? How are you? Are you feeling better?

I: what about your personal life?

R: what does personal life mean?

I: e… it means about your lifestyle. What do you feel when asks you about you your personal life?

R: I don’t feel anything , I don’t feel anything, I don’t feel anything.

I: what about your feelings?

R: what I feel is this illness only nothing else

I: about your feeling mean about the anxiety, fear, getting upset. What do you feel if he asks you this? Did he ever ask about this?

R: oh he has never asked about this. How is the illness? Are you feeling better? Did you ever have any seizure ?. how are you this last month? You will be referred to other places. I will send you to other places? This is what he said. I thought that they will give drugs only, they wont say anything else. I kept saying I am fine.

I: so you thought that you will also get follow up drugs?

R: yes

I: is there any barrier for not having a regular follow up? Do you come every month?

R: yes I thought about it two or three days prior

I: before your follow up?

R: yes

I: aha

R: I am happy even if they tell me to take the drug four time if the anxiety will get better. My God. I take three tablets every day. At night three tablets. In the morning four tablets

I: are you taking that?

R: yes every day

I; what makes you to follow the treatment every month, by keeping your appointment strictly?

R: my illness, the anxiety

I: is there any barrier not to take the drugs or to have a regular follow up ?

R: there is nothing. There is no other drugs

I: what do your family thinks about your illness?

R: about me?

I: yes

R: even before I get married I went to the holy water. The anxiety.. the evil spirit talked it was long time ago.. before I get married

I: yes , yes

R: I used to only to holy water with my mother

I: ok

R: this a place called Goro holy water, I used to go there. There is nothing found in me when I was baptized with holy water. I was very anxious. It is 18 years back before get married. I started the epilepsy drug this long time ago

I : so what do your family say about the medical treatment?

R: this is good . a good drug. They ask me whether I am having a regular follow up.

I: ok good, in general what can be done to improve the quality of life of people with epilepsy?

R: what can be done ? I don’t have anything that I thought about. I only beg God , I only pray to almighty. That is the only skill that I have.

I:it is not just only you but the health facility, the health professionals what shall they do to improve your health condition ? that is what I mean.

R: I don’t have anything to say or think. I just only pray to god to cure me. That is the only thing I am thinking

I: nothing?

R: what can I think?

I: so what can the society do so that you all will have a better life ?

R; what does the society mean?

I: society mean the people who lives around you

R: what can they think? Actually they said that to go to a holy water, the holy water will cure your anxiety. That is why I went to the holy water.

I; does this mean they were helping you a lot?

R: yes

I: the other issue is sometimes people have the wrong impression. So do the society have done bad or influenced you in the negative way?

R: there is no bad thing happened to me

I : what can they do to improve the illness ?

R: what can they think? They only say May God help her. They have no other skills.

I: there is nothing they can do?

R: yes

I: there are people with epilepsy who don’t come for follow up regularly. So what can be done so people with epilepsy can have a regular follow up and treatment like you do?

R: they have to think it by heart. Like me they have to know it by heart. I only think about my self. I think hard and when I am left with two or three days of drugs, I come to the health center. They also give me the drugs and said that I have to come early… two days earlier before I run out of the drugs. I said ok and take the drugs day and night

I: you told me that the society has helped me, they have told you to pray and to go to holy water. To the contrary was there any stigma, whether they have thought that the illness was contagious or was there any bad saying?

R: I have nothing like that

I: no problem?

R: Yes

I: no experience?

R: I have nothing like that in a place where I live

R husband: it is not contagious. I have seen it. They say it is contagious but it is a lie.

R: is it contagious?

R husband: not contagious

R: if it was contagious

I: by thinking like that do ever people have stigmatized you or influenced you?

R: they have said that if I am sick and if the kids are not around me, they have told me to be with them

I: who? The neighbours ? I understand

R: yes

I: so there is nothing that happened to upset you?

R: yes there is nothing

I: is there any additional thing that you want to add?

R: I have nothing to add. It is just only the things that I told you

I: may be there is something that I did not ask?

R: no

I: everything is enough

R: yes that is enough

I: ok Thank you very much. Thank you for taking your time and talk to me.

R; I also thank you.
