# Supplementary material for: Experience and perceptions of mental ill-health in people with epilepsy in rural Ethiopia: A qualitative study
Source: PLoS One. 2024 Dec 13;19(12):e0310542. doi: 10.1371/journal.pone.0310542 (PMC11643256; doi:10.1371/journal.pone.0310542)
Supplement: S3 File — (ZIP) [file pone.0310542.s003.zip › data set/Translation 022.docx]

Translation interview 22

I: my name is …. I will ask some questions. Thank you for your willingness…..

R: I also thank you

I; what is your name?

R; …

I: what is your age?

R: 25 years

I: what about your work?

R: I have no work.

I: what about your level of education?

R: up to grade 7

I: whom are you living with?

R: I live with my father and mother which means with my family

I: very good. Where do you live? Is it rural or urban?

R; at the rural, Sefera Gore

I: are you married

R: not married

I: what brought to you the health center for the first time?

R: ………because I have mental problem and I was sick.

I: what does mental problem mean?

R: mental problem mean epilepsy

I: what were the symptoms?

R: it is just I see something in front of me and then I fell. I get very sick

I: when did it start?

R: when I was a baby.

I: at what age?

R; at least I was 4 years old

I: what does “ see something “ mean? What do you see?

R: I feel like someone is speaking to my face, I feel like something is coming on me. I see a lot of stuff…

I: so you see something?

R: yes

I: do other people see it ?

R; I don’t know … I don’t think they can see it.

I: how long does it last?

R; five minutes sometimes it may last an hour. I feel very sick. Occasionally it gets worse and I feel very sick. If I have a fight with someone and if I am angry, I get sick, I feel it…..

I: anger mean?

R: when I have a fight with my father, If I am with him and if he does something to me or insult me when we fight, I feel upset and get sick.

I; ok so whenever you have a fight you get sick, you loss consciousness, what are the other symptoms?

R: after I had a fight with father, I feel like I am fighting again and again…

I: does the emotion last longer ? is that what you mean?

R: yes

I; what else?

R: I have nothing

I: what other illness do you have?

R: I have nothing

I: there is no illness?

R: nothing

I; so does the anger I mean the irritability lasts longer after I had a seizure?

R; it only for a few minutes. It does not last longer

I; do you have any other health problems?

R: I have no other illness

I: no health problem? Is it just the epilepsy?

R: I have epilepsy and I have pain on my hands and legs.

I: what do you feel on your hand and leg?

R :I have something on my hand

I: Is your hand problem comes with the epilepsy or is it a separate illness?

R: my hand problem comes after the epilepsy

I: what about your leg?

R:it was together with my hand

I: ok so do you think that all these problems are the symptoms of one illness or not? All the problems that you mentioned like I feel dizzy, I feel anxious do they have relationship?

R: yes they come after I had seizures…

I: do you think that they have connections?

R: yes

I: is it after that you see something?

R: yes

I: isn’t it before the seizure?

R: before the seizure I see something, then I fell have seizure. Then after a while I get better

I: do you have any other emotional problems?

R: no

I: when did you start follow up for these symptoms?

R; it was long time ago. At least five or ….ten years back

I: where did start the medical follow up?

R; I started it here in kella

I; what did they ask you when you come for medical treatment?

R: they have asked me all what you have asked me

I; what else did they ask you?

R: they did not ask anything else. They just gave me medicine and told me to use that… like this and…like that

I; what is the frequency of your follow up?

R: I used to come every month

I; you used to come every month and then you discontinued it?

R; when I was getting sick while taking the medicine then I discontinued it

I: which one made you to discontinue; does the medicine get you sick or is it because you were getting sick even if you are taking the medicine

R; when I was sick, I thought I was not cured and I discontinue it. I thought I would be cured immediately. I did not know that it will last longer

I: so you discontinued it in between?

R; yes

I: why?

R: I thought taking the medicine once would be enough and I would be cured quickly. When I was taking the medicine, I thought that I was not cured and I discontinued it. I thought I would be cured.

I: what happened when you discontinue the medicine?

R: I got sick again.

I: did it come again?

R: yes

I: then what did you do?

R: I did not do anything

I: are you having a follow up now?

R: yes I came to have the follow up again

I; you are not taking the medicine now?

R:yes

I: how long has it been since you stopped to take the medicine?

R: it is long time ago… ten years back.

I; are you saying that it has been ten years since I had follow up? How long did you have follow up after your first visit?

R: I had follow up for four up to six months.

I: did you do anything from other places? Do you still have seizure now ? how many seizures do you have per day after you stopped the medicine/

R; occasionally I have frequent seizures

I: you are not still taking the medicine

R; I did not say that I will not the medicine. i came here today to take it

I: still today?

R: yes, I used to take it before but not now

I: you did not take the medicine every day?

R: yes I discontinued it.

I: for nine years?

R: yes

I : do you have seizure?
R: yes I have seizures

I: so why didn’t you take the medicine?

R: I did not come here before. I came here today after I talked with a health professional who lives around me

I: how many seizures do you have per month?

R: I have seizure twice per month

I: so you did not come to the health center while you are having two seizures per month?

R: yes, I did not come

I:why not?

R: I thought the medicine was not improving the illness. I did not know before this. Now I know. That is why I came today after I asked her … the health professional in our neighborhood. She told me that there is no problem and I can take the medicine and get improvement.

I: do you have any other reason for not taking the drug?

R: no nothing

I: so you were not on follow up?

R: yes

I: well you should not have waited all these years. When we finish this interview you will re-start treatment and follow up.

I: do the health professionals ask you about your personal life, your social life, your education?

R: yes

I: for example do they ask you how you feel? How you are?

R: no one asked me this, asked about my sickness only

I; they don’t say that

R: yes

I: so what did they ask you?

R: when you have some other illness, they just give you medicine after their physical examination. Otherwise they don’t ask that

I; what do you feel if they ask you about your private life?

R: I will tell them what I feel……… I feel like something and something. It would be better if I do this and that

I; is it good if they ask you?

R: if they ask……… but they did not ask

I: ok, you have told me that the symptoms of the epilepsy are that you see something and you fell. Because of the epilepsy was there any problem or impact on your life?

R: no

I: no? did you understand what I mean? Because of the epilepsy was there any problem or impact on your life?

R: yes. I feel another problem.

I: what do you feel?

R: there is excessive sweat on my body and I also limp.

I: you limp. Do you ever go to health facility?

R: no, I did not go.

I: why did you ask them to take you to the health facility?

R: yes, I did but it is only my father and he does not have any education and he does not know.

I; what do your family think about your treatment and the medicine?

R: my mother is busy, she is the only one in the house . she is a house wife… it is not convenient for her. It is stressful for her to take me. My father does not think that I need treatment.

I: didn’t they say to take you to the holy water or traditional medicine?

R: no, they don’t

I: so what do they think about your illness?

R: previously they thought about to take me to the holy water place … then my grandmother helped me a lot. Because she raised me she took me to many holy water places and she helped me. That is it.

I: what about your father?

R: he did not say anything

I: what did they do so that your illness get improved or get cured?

R; now they say that to go to health facility. That is why I came today and they also said that they will take me to the health center.

I: now you can do it by your own.

R: yes

I: do you help with the house work?

R: yes, I herd the cattle.

I: what else?

R: when they harvest corn I work with them and I do some other farm activities. That is it.

I: you help?

R: yes

I: why did you discontinue your education?

R: I discontinued it when I get sick. I feel anxious when I learn. Whenever there is an exam, I started to have seizure. That is the reason I discontinued.

I: do you have friends?

R: yes

I: what do you do with your friends?

R: I go out with them. We drink coffee and talk with each other

I: so it means that you have a lot of friends and the epilepsy did not have any influence on your social life?

R: it did not influence me. They call me if I am available and we hang out together.

I: ok very good. Do you attend wedding or funerals all the time?

R: yes

I: was there anything that you did not do because of your illness?

R: nothing

I: you do everything as other ordinary person?

R: yes I do.

I: do you attend? What about the work?

R: yes, I do the work which I can. I make fence and I fetch woods.

I: it is good that you are doing what you can. Do you have girlfriend?

R: no, I don’t have

I: do you want to get married? Have you ever thought about having girlfriend?

R; yes

I : you are thinking of getting married? what do your parents say?

R: yes, they have told me that they will build me a house, give me a land. My uncles also said that they will give me land.

I: your family cares a lot about you. Does your neighborhood and the society also care about you?

R: yes, they do.

I: ok good. Did you ever encountered anything from the society saying bad or discriminate you?

R: yes, it happens sometimes. When there is some substantial work with my friends at the kebele or when there is some aid for me, they don’t give me. They take it for themselves. They have taken some salaries from me, the Kebele authorities.

I: so they took it after they promised to give it?

R: yes, initially the authorities said that I could work with my friends and get substantiated. They also register me. Then they said that I cannot work and expelled me

I: so what did you feel when they do that?

R: I was upset, very upset but what can you do…

I : that is very hard. Was there any other bad thing happened to you? have you ever been not invited to a wedding?

R: no

I: from all the problems that you have told me which one did you want to get it better?

R: I wish I could work with my friend. I wish there is some work created for me and do that.

I: from all the symptoms which one is the most bothersome?

R: that is it. it is only the seizure.

I; can you move around by your own?

R: Yes

I: Can you do may activities by your own?

R: yes

I: so you are telling me that for the last nine years you did not take the medicine or had a follow up because the medicine did not improve your illness after you took it for a while? Is that the main reason?

So what do you think should be done so that you have a better health, a better quality of life?

R: if I ever get cured from my illness.

I: what does cure mean?

R: if the seizure stopped. If I get help to stop my seizure I would be happy.

I: who shall help you? What do you think should help you? the health center, the health professional or the community?

R; it should be the health professional

I; please tell me again the reason you delayed the treatment. You did not have the follow up because you were not getting any improvement?

R: yes, I was not cured. I took it once and there was no improvement and I did not know about helth issues. Because I did not know anything about health I did not go.

I: did the health extension worker come? Didn’t she come to your house, to your village every month? Dint she come and gave health education?

R: there is nothing like that. The first health extension used to come but now she is changed.

I; whenshe comes doesn’t she give health education about epilepsy and tells to get treatment

R: yes she did

I: then if she did that how come you did not get the treatment

R: yes I asked her even before she came to our house.

I: what did you ask her?

R: I am sick like this and that. I asked her what shall I do?

I: ok what did she say?

R: she told me to go to the health center. Then I came here. The new changed health extension worker also asked me that I have sickness like that and I said yes. She also asked why did discontinue the medicine and I told her that I did not know about the medicine. Then she told me that there is no problem with my discontinuation of the medicine, she registered me and told me that I will be called ad restart the medication. It is with her help that I came here today.

I: do you remember what kind of drug was it prescribed to you? Do you know it? Was it taken in the morning or in the evening or both?

R: it was a kind of red.

I: red kind? Did they tell you the purpose of the medicine?

R: they told me that the seizure would improve

I: ok, did they advise you how to take and how much should be taken?

R; no they did not

I: they did not, ok. So do you think that the health professionals should proper and full treatment?

R: yes

I; ok what should the society do so that you will have a better health, better quality of life and so that you can work equally as your friends?

R: the society should find me a job when my friends are doing some jobs. I would be happy if I work, give me a job.

I: is that a job opportunity equally as your friends?

R: yes, even if I was not able to work what they do, it will be good if I work other jobs.

I: what else?

R: nothing else.

I: what about other people with epilepsy what do you think should be done for them so that they can have a better quality of life?

R; they should be helped like me like this one.

I: what kind of help?

R: for example to give aid. For those who does not know anything like me should be helped. I also think that for those who can work should be helped to work.

I; do you think that people with epilepsy need special kind of aid or help?

R: yes

I ; what kind of help?

R: something like to help them in getting their medicine monthly.

I: provision of monthly medicine. What else?

R; there is nothing

I: what about the discontinuation?

R: health education should be given to stop discontinuation of medicine.

I: who should give the health education?

R: the health professional should give advices. For those who are taking the medicine they should give advice on how to take the medicine, they should advice not to discontinue.

I what about the health center?

R: the health center should help. It should follow patients and ask them that they are taking the medicine at least once in 2 weeks or a month. Even if they encounter patients on the road they should ask them about the medicine

I: so do you think that a good follow up will improve the disease?

R: yes

I: Ok, your family said that they will build you a house and will support you. What do they say about the medical treatment?

R: they don’t know anything. Because they are not educated they don’t know anything.

I; what about your brothers?

R: my brothers are younger than me, they don’t know anything.

I: was there anything that I did not ask you?

R: nothing

I: did you tell me everything?

R: yes

I: thank you very much for the interview
