# Supplementary material for: Experience and perceptions of mental ill-health in people with epilepsy in rural Ethiopia: A qualitative study
Source: PLoS One. 2024 Dec 13;19(12):e0310542. doi: 10.1371/journal.pone.0310542 (PMC11643256; doi:10.1371/journal.pone.0310542)
Supplement: S3 File — (ZIP) [file pone.0310542.s003.zip › data set/translation 03.docx]

**Code Buirithoo1**

**Interviewer**: Okay, thank you very much for being volunteer and come here. Your code is 01. Let’s first ask you some questions about ---------- 0.34. Hold it like this and make your voice louder to hear you clearly.

**Interviewee**: Okay

**Interviewer**: Make it close to your mouth and you will answer me by making your voice louder. How old are you?

**Interviewee**: I am about thirty years old.

**Interviewer**: Okay, what about work? Do you have work?

**Interviewee**: I don’t have any work and I am dependent to people after I became sick. The town only

**Interviewer**: Where did you live?

**Interviewee**: Around Mosque.

**Interviewer**: At *Buee* town?

**Interviewee**: Yes

**Interviewer**: Are you married?

**Interviewee**: Yes. I have two children.

**Interviewer**: Do you have two children?

**Interviewee**: I have two children.

**Interviewer**: Okay, thank you. Let me ask you some questions about your treatment. What was the problem that brought you to the health institution at the first time?

**Interviewee**: At the first time, I was told that my lung had problem and I started the treatment. First, I was taken to *Butajira* and they gave me glucose and they prescribed me medication. I don’t know whether it is the medication or human hand (a spell on me), my hand can’t move when I started the medication. Then I was just admitted. I was healthy until I gave birth of two children and I was married. Then my husband left me and I started living with my mother. Since then, thanks to God, I live by the help of you, by the help of people

**Interviewer**: What did you feel when you first came here for treatment? What was the symptom on your body?

**Interviewee**: Something just moves on my abdomen. It moves from side to side on my abdomen and it moves to my head. I was confused and didn’t go to holy water, I just started medication here. When I started that medication it tied my hand and leg and they also move me to toilet. Then, I was diagnosed with pulmonary TB after I completed that medication. After I completed the medication I went to holy water. Then, my leg started moving after I went to holy water. After I started walking, I became sick of epilepsy disease and then I started medication. They referred me to *Buatjira*, they told me to go to where I had been treated first. They sent me to here and I started the medication. It has been a while since I started the medication here.

**Interviewer**: Tell me in detail about the epilepsy, what were your symptoms? Did you have symptoms before your seize?

**Interviewee**: Yes

**Interviewer**: Explain it to me.

**Interviewee**: When it starts to seize me, something in my hand vibrates. Something vibrated in my hand when I am going to seize. It is just like that.

**Interviewer**: After what you told me, were there other symptoms that you manifest or other type of disease symptoms?

**Interviewee**: This is the symptom, it is only that. It is my left arm which is very painful. It is painful over here and it is like a foreign body.

**Interviewer**: Is your hand become like that before or after the epilepsy?

**Interviewee**: It is before.

**Interviewer**: Is it happened before?

**Interviewee**: It tied me when I started the TB medication. After that, I didn’t go to holy water and didn’t say anything. They gave me the medication and they told me as it is not the medication which did that. I don’t know whether it is when they secured the glucose or not. It just tied my hand. I completed the medication after six months. I went to holy water after I completed the medication. After I went to the holy water, I began walking. They took me there by chain and started standing up. This epilepsy happened to me after I started waking.

**Interviewer**: Why did they bring you there by chain?

**Interviewee**: It means by chair, *Kareza*.

**Interviewer**: By Kareza, did they carry you?

**Interviewee**: Yes, *Kareza*. They carried me by Kareza and took me there. I came back by my foot after a while.

**Interviewer**: Did you know about epilepsy before?

**Interviewee**: I don’t know anything.

**Interviewer**: What do you think are the symptoms of epilepsy?

**Interviewee**: It just throws some people to the ground. Some of them twist their necks, some will be anxious when they are going to seize. Some of them hands will be shake, my hand also shake when I am going to seize. I will just seize after my hand shake.

**Interviewer**: Do you have any other illnesses other than the epilepsy?

**Interviewee**: I don’t have any other illness.

**Interviewer**: For example, other co-morbidities related to mental health illness.

**Interviewee**: I don’t have anything else; I don’t have any illness. It just ache my head and I have dizziness. I just have nothing else I know.

**Interviewer**: For example, what about things like anxiety and depression?

**Interviewee**: Sometimes I feel anxious.

**Interviewer**: Tell me about that.

**Interviewee**: Sometimes I wonder what kind of life it is? And I feel hopeless and I will be anxious.

**Interviewer**: How often this happens? Is there a lot of stress?

**Interviewee**: Hope

**Interviewer**: What do you do when you are depressed?

**Interviewee**: Sometimes I give up ( feel hopless), sometimes I say only what God say will happen. I said let God do what he want.

**Interviewer**: Are there signs that you see when you are depressed? It means the things you do when you are stressed or did you seize when you are depressed?

**Interviewee**: I will not seize.

**Interviewer**: *Eee*

**Interviewee**: I will not seize

**Interviewer**: Okay, what about the other things, it could be things related to mental illness or it could be substance uses, such as drinking or chewing *khat* or it could be using different type of things.

**Interviewee**: I don’t drink alcohol.

**Interviewer**: *Ehh*

**Interviewee**: I don’t drink alcohol after I started medication. I don’t want to drink and I don’t even want to smell alcohol. I just want to save my life.

**Interviewer**: Okay. Moat of your symptoms that you told me are depression, dizziness and seizure; you told me that.

**Interviewee**: Yes

**Interviewer**: What kind of impact do these things have on your life?

**Interviewee**: Nothing. What will you say, what God brought is what God will solve it one day. God have its own time; it is not what we say. It can’t be solved by worrying, God have its own time and one day I hope God will save me.

**Interviewer**: Do you hope?

**Interviewee**: Yes

**Interviewer**: Did this illness affect your life, job and your relationship with your family?

**Interviewee**: What do you mean?

**Interviewer**: For example

**Interviewee**: *Eee*

**Interviewer**: The thing you say I would have done this if I don’t have this illness,

**Interviewee**: I say

**Interviewer**: What do you say that are missing?

**Interviewee**: I say, I used to work like the others. I thought that I used to work like the others if my hand is well, but it is what God have done to me, what I can do.

**Interviewer**: For example, did it affect or impose problem your work or relationships? Or is there things that you say it is because of my illness I became like this?

**Interviewee**: I will say that when people disregard me.

**Interviewer**: *Ehh*

**Interviewee**: Sometimes people disregard you.

**Interviewer**: *Ehh*

**Interviewee**: I say why they did this to me. I said I was like them I used to work like them. I was like them, how can they disregard me.

**Interviewer**: *Ehh*

**Interviewee**: I may say that why a person is disrespected.

**Interviewer**: *Ehh*

**Interviewee**: But, I will say let them do what they want. I will say anything happen when God say and will forgive them.

**Interviewer**: What do you feel when you compare yourself with other people who do not have epilepsy or who do not have depression? Are there things that you say I can’t do this since I am different from other people?

**Interviewee**: I will say.

**Interviewer**: Please tell me your feeling without fear. Please share me the things that you thought while being at your home.

**Interviewee**: It will always be what God say. I can’t do anything that God has commended me to do. If God allows I can do my work like anyone else and I can live in my marriage, and if God don’t allow nothing can be done.

**Interviewer**: Do you think the problem with you marriage related to your illness?

**Interviewee**: Yes, my marriage broken because of my illness.

**Interviewer**: Which illness? Is it the first one or the epilepsy?

**Interviewee**: The first one.

**Interviewer**: By the pulmonary TB?

**Interviewee**: Yes

**Interviewer**: *Ehh*

**Interviewee**: My husband left me since I was sick like that. I just sat down and I gave up when I can’t move. And it was like that. I was like that in the first place and I become like this after I become sick.

**Interviewer**: What are the symptoms that you are most concerned and wish it would be easier? Which symptom you are most worried about?

**Interviewee**: It is just that one, the epilepsy.

**Interviewer**: How often do you seize? Doesn’t it have improvement after the medication?

**Interviewee**: It has

**Interviewer**: *Eee*

**Interviewee**: It has improvement after the medication. I will be depressed when there is no medication and I will seize frequently.

**Interviewer**: What do you feel now?

**Interviewee**: I forgot the disease.

**Interviewer**: Which of the symptoms are you more concerned at this time?

**Interviewee**: There is nothing, I am fine now.

**Interviewer**: Are you fine?

**Interviewee**: Yes

**Interviewer**: There are symptoms that you told me, right?

**Interviewee**: Yes

**Interviewer**: You told me seizure and dizziness.

**Interviewee**: Yes

**Interviewer**: You told me that you will be tired and depressed. Which one do you like if we treat it that you think your life will be easier?

**Interviewee**: You are the one who know about that, I don’t know anything.

**Interviewer**: No, which of the symptoms do you say that hurts you a lot?

**Interviewee**: Sometimes I feel depressed and sometimes I feel better. Sometimes it just wants to seize me and sometimes I will be fine. So, if you have solution for that.

**Interviewer**: Which one does it mean? Is it the stress?

**Interviewee**: Yes

**Interviewer**: What did you do to reduce your stress?

**Interviewee**: I can’t stand and do anything. I will buy and take soft drink.

**Interviewer**: What else?

**Interviewee**: That is it.

**Interviewer**: Tell me if there is any stigma and discrimination from the community, people who live with you, it could be from family or neighbors because of the disease

**Interviewee**: There is no

**Interviewer**: Tell me?

**Interviewee**: There is no. I just ignore then when people try to talk to me.

**Interviewer**: *Ehh*

**Interviewee**: My life is more important than anything. I will ignore and leave when anyone talks to me.

**Interviewer**: But, do they say something?

**Interviewee**: Even if they say something, I will ignore them.

**Interviewer**: What did they do? What type of talk or action do they do?

**Interviewee**: They just say it is your disease that makes you sick, but I will ignore them and say God know what will happen.

**Interviewer**: Do you participate in social life, such as *Ekub* and *Edir*?

**Interviewee**: I don’t participate. I can’t participate.

**Interviewer**: Going when you are called at *Mahiber*

**Interviewee**: Sometimes I will go when they call me.

**Interviewer**: *Ehh*

**Interviewee**: Some disrespect you and don’t care about you.

**Interviewer**: Are there things like disrespecting?

**Interviewee**: Yes

**Interviewer**: Are there?

**Interviewee**: Yes

**Interviewer**: Tell me about it, what did they do?

**Interviewee**: Just some people will disregard you and some insult you. Due to that, I say why I come here.

**Interviewer**: *Ehh*

**Interviewee**: And the others will invite you properly and served you well.

**Interviewer**: *Ehh*

**Interviewee**: There are some people who will serve you well. I will not be upset. I don’t touch any person that is my behavior.

**Interviewer**: But, sometimes in the community

**Interviewee**: Yes, I live a little bit far from the village.

**Interviewer**: *Eee*

**Interviewee**: They liked me most because of my behavior. I don’t touch any person and I don’t insult anyone. It is just like that.

**Interviewer**: Okay. You told me how much the disease imposed you impact. What did you do to be cured from this disease?

**Interviewee**: That is like what I told you. I told you that I used this medication and then I went to holy water. After the holy water, they told me as it is epilepsy ……….19.5ዐ. Then I started the medication. While I was going to start the medication at *Butajira*, they referred me to here. Then I started taking the medication.

**Interviewer**: Okay. How did you choose to be treated here? You are being treated at this hospital, right?

**Interviewee**: Yes

**Interviewer**: How was it, tell about that?

**Interviewee**: It is just here since I live here.

**Interviewer**: Did you choose it since it is nearby?

**Interviewee**: Yes

**Interviewer**: *Ehh*

**Interviewee**: Yes

**Interviewer**: How was the treatment?

**Interviewee**: The treatment is good.

**Interviewer**: What does the epilepsy treatment looks like?

**Interviewee**: The treatment of epilepsy, is it the medication?

**Interviewer**: How is the medication they give to you?

**Interviewee**: It is good. Sometimes it has burning sensation and it is its side effect. I don’t like when I have that feeling.

**Interviewer**: What type of side effect?

**Interviewee**: I mean I don’t like when I don’t take meal adequately. It hurts my stomach. Due to that, sometimes I will take soft meals and it after that I will take medication.

**Interviewer**: *Ehh*

**Interviewee**: Otherwise, I don’t take medication in empty stomach.

**Interviewer**: *Ehh*

**Interviewee**: So the medication is good, it relieved me. It relieved me and I like the medication so much.

**Interviewer**: I will come, wait me.

**Interviewee**: Okay

**Interviewer**: Okay, I will call you back.

**Interviewee**: Okay

**Interviewer**: We don’t finish, okay?

**Interviewee**: Okay. Shall I go or what?

**Interviewer**: Wait me, okay. I am sorry.

**Interviewee**: No problem.

**Interviewer**: How did you find the improvement after the treatment?

**Interviewee**: I used to take the medication once a day.

**Interviewer**: *Eee*

**Interviewee**: I used to take the medication once a day.

**Interviewer**: Okay

**Interviewee**: Once when I told them I am not improved they increased the dosage of the medication, they make it day and night.

**Interviewer**: Did it improve after that?

**Interviewee**: Yes

**Interviewer**: Okay, what else?

**Interviewee**: It is improved now.

**Interviewer**: What else did you do to improve your life? Are there things you do to reduce the depression at your home with your family?

**Interviewee**: What can I do with my family?

**Interviewer**: To relieve your depression.

**Interviewee**: I will take a shower.

**Interviewer**: *Ehh*

**Interviewee**: That is it.

**Interviewer**: Do you feel better when you do that?

**Interviewee**: Yes. I will move by my energy I have. I don’t like sitting.

**Interviewer**: *Ehh*

**Interviewee**: I will get up and wash myself with my one hand. I will wash my self and feel better. When I told them the medication I took at night didn’t improve me they increased the dose of the medication, they make it day and night. They don’t let me to wait when I come, they will serve me immediately.

**Interviewer**: Which solution benefited you a lot? Which one did you benefit most from the care and treatment you received from this hospital?

**Interviewee**: It is the medication.

**Interviewer**: The medication?

**Interviewee**: Yes, it is the medication, with the help of God, which help me. I couldn’t live without it.

**Interviewer**: How was their approach when you came here for treatment?

**Interviewee**: It was very good.

**Interviewer**: *Eee*

**Interviewee**: It was good.

**Interviewer**: Tell me a little bit about that.

**Interviewee**: They are good. They are good for me, they don’t upset me.

**Interviewer**: *Ehh*

**Interviewee**: They serve me immediately. They bring medication for me as I can’t move. They give me priority and treat me.

**Interviewer**: What kind of questions did they ask you when you first came here? Did they ask you well about your illness?

**Interviewee**: Yes

**Interviewer**: What did they ask you?

**Interviewee**: They asked me, what do you feel? What do you like? Do you like music? They asked me like that.

**Interviewer**: Okay

**Interviewee**: I told them before as I don’t like music, it is just for fun.

**Interviewer**: In addition to your illness there are a lot of things that worry you in your daily life, right?

**Interviewee**: Yes

**Interviewer**: Did they ask about that?

**Interviewee**: Yes, they ask me.

**Interviewer**: What did you feel when they asked you about that?

**Interviewee**: I didn’t feel anything. I don’t feel anything. Now I have nothing to worry about, I just want to be cured, and I told them as I want to be with my children when they asked me. They asked me if I have urged to urinate and defecate when I seize.

**Interviewer**: *Ehh*

**Interviewee**: I don’t have that feeling. I told them as I become fine immediately after I seized.

**Interviewer**: How many times did you come to this health center?

**Interviewee**: It has been too long.

**Interviewer**: How often do you follow-up?

**Interviewee**: Every month.

**Interviewer**: Do you come every month?

**Interviewee**: Yes

**Interviewer**: Did they tell you about the type medications prescribed for you? What do you know?

**Interviewee**: About the prescribed medications?

**Interviewer**: As what type of medications prescribed for you

**Interviewee**: I don’t know. When they tell me to take which one to take in the morning and which one in the evening, I will take what they tell me to do, but I don’t know anything about the medications.

**Interviewer**: About the feelings that you may feel when you take the medications, some medication may be difficult.

**Interviewee**: Yes

**Interviewer**: Did they explain these things to you?

**Interviewee**: Yes

**Interviewer**: What did they say?

**Interviewee**: They told me to take fluid. Take fluid, the medications are risky. They warn me take adequate fluid and they will give me the medication after that. I will be sick when I take the medications if I am not comfortable at home.

**Interviewer**: *Ehh*

**Interviewee**: I have to take much fluid I can before I take the medication.

**Interviewer**: *Ehh*

**Interviewee**: I will take the medication after that.

**Interviewer**: Do they tell you that?

**Interviewee**: Yes

**Interviewer**: Have you ever forget to take medication?

**Interviewee**: I will not forget, it is my life

**Interviewer**: Didn’t you forget even for once?

**Interviewee**: No, I didn’t.

**Interviewer**: Didn’t you? Why do you think we should take medication regularly without forgetting?

**Interviewee**: It is to preserve life.

**Interviewer**: What will happen if the medication is discontinued?

**Interviewee**: The disease will relapse. The disease will be relapsed, but if you take the medication on time, you will get rid of the disease. That is it.

**Interviewer**: Okay. Do the health professionals or doctors ask you about your personal life?

**Interviewee**: What do you mean?

**Interviewer**: About your personal life, living situation, and familiar status

**Interviewee**: Yes, they ask me a lot of things.

**Interviewer**: What kind of questions do they ask you?

**Interviewee**: They ask me a lot of things. They knew about my personal life and family. I will tell them as they know my standard of living.

**Interviewer**: Now, for example, what do you feel when they ask you? Some people say why they don’t just give me the medication and why they ask me about my family and my life; what do you feel when they ask you such type of question? Don’t you want them to ask you, or are you happy to be asked?

**Interviewee**: I am happy.

**Interviewer**: Why, tell me about that?

**Interviewee**: I am happy; it is just to know who I am.

**Interviewer**: *Ehh*

**Interviewee**: It is just to know who I am, as I am weak and don’t have anyone one who helps me.

**Interviewer**: *Ehh*

**Interviewee**: They asked me to know that but it is not for anything else.

**Interviewer**: There are some people who want to share their worries, and sometimes there are people who don’t want to share to anyone, right? Which one do you like? Do you like to discuss or not?

**Interviewee**: I like to discuss. It is good to discuss. It is better to share your thought than being worried.

**Interviewer**: Are there problems that you encounter to come to this health center?

**Interviewee**: I don’t encounter. No, I don’t encounter anything.

**Interviewer**: It could be lack of time, not finding someone who comes with you or transport problem that you worry about when you come here that you say if these things improved I can come here easily.

**Interviewee**: As it is in the nearby, I will come with my children, but I can’t come by foot.

**Interviewer**: *Ehh*

**Interviewee**: Sometimes they bring me Bajaj.

**Interviewer**: *Ehh*

**Interviewee**: They will understand and help me, and sometimes I will pay and come if someone gives me money.

**Interviewer**: *Ehh*

**Interviewee**: That is it, I am happy.

**Interviewer**: What type of suggestions did your family, friend and neighbors gives about your treatment?

**Interviewee**: What do you mean?

**Interviewer**: Did they say the treatment helps you or not? What did they say?

**Interviewee**: They don’t know about that; it is me which knows.

**Interviewer**: *Ehh*

**Interviewee**: It is me who know all about my treatment, my illness and my improvement; nobody knows about that.

**Interviewer**: *Ehh*

**Interviewee**: It is me who knows as I am fine or not.

**Interviewer**: Are there no one who say that you have improvement or not?

**Interviewee**: They don’t know if they so too. I am the one who is happy.

**Interviewer**: *Ehh*

**Interviewee**: I will say this medication improved me like this and will be happy.

**Interviewer**: What about family? People who live with you know when you are stressed or sick, right?

**Interviewee**: Yes

**Interviewer**: How do they feel about your treatment?

**Interviewee**: They say it is good. The medication is good and it makes you better. They say you were stressed before you start the medication and they are happy about it.

**Interviewer**: *Ehh*

**Interviewee**: But it is me who knows everything about me.

**Interviewer**: In general, do you anything that you think is good for people with epilepsy or anything you want to say if we can help them in this way?

**Interviewee**: Yes

**Interviewer**: What is it?

**Interviewee**: Yes, it is good if everyone like that gets support and if they get chance like what we get. It would be nice if they had such type of opportunity.

**Interviewer**: *Ehh*

**Interviewee**: There is no such type if thing. We don’t pay for the treatment, it is free. We are being given medication and we are saving our lives.

**Interviewer**: *Ehh*

**Interviewee**: I think it is good for everyone who has the disease to get that kind of opportunity.

**Interviewer**: What else should the community do?

**Interviewee**: The community, what can I say

**Interviewer**: For example, you told me that some people will disregard and some people will treat well, there are different things. What can the community do to improve the lives of people with epilepsy and make them better mentally?

**Interviewee**: The community is supportive and they aid. There is no community which is more supportive than this community. The people of this town are very nice. They care too much for people. I can’t tell you, they are very nice for other people.

**Interviewer**: What should the health professionals do?

**Interviewee**: The health professionals renew the health insurance and gave it to me. They let us to be aided for free and they are very cooperative and helped us a lot.

**Interviewer**: *Ehh*, *Ehh*. What should the hospitals or health centers do?

**Interviewee**: The hospitals treat us well according to the instruction without hesitation. They are treating us in an orderly manner by giving priority for sick one.

**Interviewer**: *Ehh*

**Interviewee**: They treat us properly and they don’t disregard. They don’t make mistake and they are very supportive. They are very helpful and may God help them.

**Interviewer**: *Ehh*

**Interviewee**: The people of this town are always very nice and very kind to others. They are very helpful, and above this may God help them.

**Interviewer**: Okay, thank you very much! In addition to the epilepsy, there is being depressed by thinking about different things, right? What do you think could be done to treat and improve that?

**Interviewee**: Which one?

**Interviewer**: The stress. Beyond the epilepsy there is also stress, right?

**Interviewee**: It is thought that stress me. You will be stressed when you have a lot of thoughts. If someone shares you an idea it means it has helped you.

**Interviewer**: So, what is the solution?

**Interviewee**: The solution

**Interviewer**: Did you say sharing an idea with people? When you say someone gives an idea

**Interviewee**: That means, it will relieves you of that stress when you talk about it with other people.

**Interviewer**: *Ehh*. Is there anything you want to add and say that I didn’t ask you?

**Interviewee**: No, there is nothing. Besides that, may God bless the health professionals; they are my mother and brothers. They care for me; they are very respectful of me. They treat me well and they are worried since I can’t move when I come here. I will take it every month, and beyond that may God bless them.

**Interviewer**: Okay, thank you very much! I took your time.
