# Supplementary material for: Experience and perceptions of mental ill-health in people with epilepsy in rural Ethiopia: A qualitative study
Source: PLoS One. 2024 Dec 13;19(12):e0310542. doi: 10.1371/journal.pone.0310542 (PMC11643256; doi:10.1371/journal.pone.0310542)
Supplement: S3 File — (ZIP) [file pone.0310542.s003.zip › data set/translation 05.docx]

**Interviewer**: Okay, thank you very much. Hold it like this and make your voice louder to communicate clearly.

**Interviewee**: Okay

**Interviewer**: Make your voice louder so we can get your voice well.

**Interviewee**: Okay

**Interviewer**: Thank you very much again for taking time and coming to us. Hold it like this. First of all, I would like to start with a question, how old are you?

**Interviewee**: I am about fifty years old now.

**Interviewer**: Fifty?

**Interviewee**: Yes

**Interviewer**: Do you work?

**Interviewee**: I had been working in the past.

**Interviewer**: Okay

**Interviewee**: I had been working in the past. It had been eighteen years since I fall into fire. I was burnt at the age of sixteen. I fall into the fire when I run out of pills. I fall into the fire while holding my child like this when he was fifteen days old, then God saved him. I am fine now. I became like that due to the fire burn. It had been sixteen years since my hand amputated.

**Interviewer**: In the past?

**Interviewee**: Yes

**Interviewer**: Haven’t you ever worked after that?

**Interviewee**: I had been working before. I stitch up, bake *Enjera* at others home, I will do what I can at others home and raise my child. I didn’t have any problem before this happened in the last eighteen years.

**Interviewer**: Yes. Did you stop the work after that?

**Interviewee**: Yes, after I fall into the fire.

**Interviewer**: What about education?

**Interviewee**: After that, I have been afraid of getting near to fire.

**Interviewer**: What about education?

**Interviewee**: I am illiterate, I didn’t learn.

**Interviewer**: Where do you live?

**Interviewee**: I am at here *Buee*.

**Interviewer**: What about your marital status? Are you married?

**Interviewee**: I was married at rural village.

**Interviewer**: *Ehh*

**Interviewee**: I was married at rural village, and then I became sick when I found and pick a sick child. When another person child came here to call me for coffee and fall, I picked him up and hugged him. I hugged him and then I become ill but the child was fine.

**Interviewer**: Are you married now?

**Interviewee**: Thanks for God, now I am married and have children. I live with my children at this time.

**Interviewer**: How many children do you have?

**Interviewee**: Me?

**Interviewer**: Yes

**Interviewee**: I have five children.

**Interviewer**: Five?

**Interviewee**: Yes, I have five children other than those who died. This little boy who is sixteen years old is just with me now, the other one went to school.

**Interviewer**: Let me start my question with, why did you come to the health facilities first? What type of symptoms did you have?

**Interviewee**: I fall by this side. I fall after the week after I picked up the child at the market.

**Interviewer**: At the market, okay.

**Interviewee**: They picked me up when I fall at the market and my child was not with me. I hugged the child and came to Addis Ababa for examination. Then they examined me and at that time I seized every day, but thanks to God I am fine now.

**Interviewer**: Are you fine?

**Interviewee**: I am fine. I am fine after I got this pill. I am just fine. Sometimes I seized and this is my child who is with me. I have dizziness when I walk on the road. Thanks to God I am fine since I started my treatment. I take this pill every night. I have been given two types of pills and now I have it. If you still see it, I have the pills since I am afraid to don’t fall when going out on the road. Thanks to God I didn’t miss anything. But I can’t work and get married; unless I look support from my family, I don’t have anything. Anyways, thanks to God.

**Interviewer**: Do you have any other health problems other than the epilepsy?

**Interviewee**: It is just that. I had been sick before and I came here and take medication.

**Interviewer**: What kind is it?

**Interviewee**: I will not come here; I will go to holy water. I will drink holy water and it will protect me well. I will bring this and take holy water. I will only come to you, Addis Ababa to take pills.

**Interviewer**: What do you think about the symptom of epilepsy disease before? Do you know about it or tell me about the disease?

**Interviewee**: I don’t know anything. I don’t know who I am when I fall. I don’t what will happen to me when I fall. It is since I don’t know what will happen my children will be with me.

**Interviewer**: Okay, do you have any other symptoms, for example, symptoms related to other mental illness or something other than the seizure?

**Interviewee**: I don’t have.

**Interviewer**: Do you have stress and like?

**Interviewee**: Yes, I don’t like to sit indoor. I will seize immediately when I feel stress.

**Interviewer**: Will you seize?

**Interviewee**: Yes

**Interviewer**: Are you stressed?

**Interviewee**: Yes, I am stressed.

**Interviewer**: Tell me about the stress?

**Interviewee**: I am stressed about what will happen to my children. I am stressed about what will happen to my children since I have never worked and don’t have income. I can’t get anything from my hand other than what I got from my family.

**Interviewer**: Otherwise you don’t have something like pain that is stressful when you wake up every day, feeling of tiredness or

**Interviewee**: I will sleep. I will sleep at that time, but I will not do anything.

**Interviewer**: What impacts do these symptoms have on your life, work and the lives of your children?

**Interviewee**: What can I do? I don’t know anything.

**Interviewer**: It means, what type of problem did it cause? Do you have anything you say I missed since this thing happens to you?

**Interviewee**: I didn’t have this thing if I didn’t pick and hug the child. I was a type of person whom I can work and don’t have anything if I didn’t pick that child when he fall at my door and I didn’t notice at the girl was at my hand. They fight with the other person by saying whey you didn’t pick her and she is only sick. I didn’t even say I am sick and then they took me for examination on the next day.

**Interviewer**: What things happened to your life since this disease after that?

**Interviewee**: I have nothing after that.

**Interviewer**: I mean did you stop your work? What happened to your marriage and children?

**Interviewee**: I have no problem at that time.

**Interviewer**: No. What kind of problem did the illness cause to you after it happens?

**Interviewee**: Nothing

**Interviewer**: Did it stop you from doing your work?

**Interviewee**: It is after I fall into the fire.

**Interviewer**: After that

**Interviewee**: Yes, I don’t know this thing. This child cursed yesterday but he wasn’t sick before. I just hugged him when he fall, but the child become well. I became ill after a week and there was a woman at the time I fall and she covered me with close. I don’t know, I don’t know anything since I fall. I don’t know anything when I become sick. I didn’t even go to *Butajira* for medication; I just came to Addis Ababa. After I gave birth to my child I went to Addis Ababa with my three months child. I just went to Addis Ababa when they told me there is medication there. And now, thanks to God, I am buying and taking the medication. The medication didn’t come for fifteen days when I gave birth to this child. I was sixteen years old when I deliver this child. At that time, I fall into the fire when I walk from outside to home and they fire burnt my body. At that time, people from the neighborhood came took me and my child out from the fire. My child cured, thanks to God. My hand amputated at Addis Ababa and this one of my hand is functional. Now, thanks to God, I am paying for the medication and I am taking the medication. If there is nothing I can eat, I will take it by empty stomach, but it hurts. I have dizziness but I don’t fall at this time. When I don’t eat, I will be sick. Thanks to God, I have two children.

**Interviewer**: Which symptoms of the disease you say that I shouldn’t have in order to feel better?

**Interviewee**: Symptom?

**Interviewer**: Yes

**Interviewee**: If I don’t have it

**Interviewer**: Symptoms that makes you feel better if not present

**Interviewee**: It is good if God give me a little thing but I am fine after I drunk the holy water. I am also fine now and it has been three years since I start drinking the holy water. I am fine now. I used to take it three times a day but now I take at night time.

**Interviewer**: The medication?

**Interviewee**: I take the medication. In the past I took it in the morning, afternoon and evening. But now after I start taking holy water I am fine thanks to God. I can’t even meet other peoples but know I am fine thanks to God.

**Interviewer**: Are there discrimination and stigma from the society since you have epilepsy?

**Interviewee**: Yes

**Interviewer**: Tell me more about it

**Interviewee**: Yes. They say what will happen to you. It is said you will die.

**Interviewer**: Tell about it; what will be said in the neighborhood and family?

**Interviewee**: It is like that in the neighborhood. I run away when they said that in the neighborhood.

**Interviewer**: What do they say?

**Interviewee**: I will tell you.

**Interviewer**: What do they say?

**Interviewee**: They say you are handicapped. It annoys you when they say that. You have to run away from them. If I don’t run away, I will meet that person. I am not lucky but thanks to God, but I am fine.

**Interviewer**: Is there discrimination and stigma from the family?

**Interviewee**: Some of your family will do that. There are some who care about what you eat, but there those who wish your death. But I don’t care; I already got it so you can do anything. What can I do, it is a thing thing that God gave me.

**Interviewer**: When you are stressed because of the disease?

**Interviewee**: I will be angry when they said that.

**Interviewer**: Do you fall at that time?

**Interviewee**: Yes, there is at that time. Yes, it may hurt you at that time. Nothing will happen to me, thanks to God. Thanks to God for giving me the pill from you.

**Interviewer**: I have seen the effects of your fire burn accident and about the discrimination, and you told me about the things at the time when this disease begins you. What did you do to get rid of this disease?

**Interviewee**: To be cured?

**Interviewer**: Yes

**Interviewee**: I want to be cured by the medication and holy water. Thanks to God, I want to be cured and be equal to everyone.

**Interviewer**: What did you do to be cured?

**Interviewee**: What can I do other than this pill and holy water; I can’t do anything. I can’t do anything.

**Interviewer**: How was the treatment? Does it have improvement?

**Interviewee**: If I don’t eat, the pill will not be comfortable and it will burn me. Other than that, if I get food and drink, I don’t have any problem. If you don’t get this what can you do, if God give me this? But if you want to be cured, for example, I am praying to God saying how you are going to save me. I am praying for God to save me, but I don’t know at this time. You pill are helping me, it makes me feel healthy.

**Interviewer**: What did you do to don’t be disturbed when your family and neighborhood discriminate you?

**Interviewee**: I will run away from neighborhoods and I don’t want to go in to that home. Why do I upset, so I want to leave that home? I want to be with my children alone.

**Interviewer**: Where was the treatment?

**Interviewee**: There

**Interviewer**: Where?

**Interviewee**: Amanuel, Addis Ababa

**Interviewer**: Have you ever got treatment here?

**Interviewee**: I haven’t treated here.

**Interviewer**: You never went?

**Interviewee**: I came here once when it don’t came to my home.

**Interviewer**: Okay. Where? Is it in this hospital?

**Interviewee**: Yes, I got it here. I took it but my disease relapsed and seizes me every day.

**Interviewer**: The medication you received here.

**Interviewee**: Yes, I throw that medication and they bring me from there. That is the only medication I use, that is where I get the benefit.

**Interviewer**: Is it there?

**Interviewee**: Yes

**Interviewer**: Don’t you ever been treated and talk to doctors here.

**Interviewee**: No, there is no one here. I was scared of that after I took the pill from here and became sick. I also sent to other place and the medication come for me from there.

**Interviewer**: You said treatment like that not given here

**Interviewee**: Yes, there is no such type of treatment here.

**Interviewer**: Did they ask you questions about your day to day life when you go there?

**Interviewee**: Yes, they asked. I was sick at that time and my sister was with me. I went with my sister and she took the medication since I was sick and unconscious.

**Interviewer**: What do you think is the most effective solution to your problem?

**Interviewee**: That helps me

**Interviewer**: The solution that you say helps me now

**Interviewee**: The pill

**Interviewer**: The pill. Did they reserve the medication here?

**Interviewee**: It has been three years since I go to the holy water but it was just pill that came for me before.

**Interviewer**: Don’t you go there and meet them? Is the pill just bought there and sent to you to here?

**Interviewee**: Yes, it will be sent to me.

**Interviewer**: Are you not going?

**Interviewee**: I will not go there; I just went two to three times to that town. I was going there to that town, but after that I will send the paper and they will send it back.

**Interviewer**: The medication?

**Interviewee**: Yes, the medication. The medication is good for me, it keeps me safe.

**Interviewer**: What did the doctor here ask you when you discontinue the medication and come here?

**Interviewee**: My pill was there and I asked them to give me the pill if they have here, and then they said let’s see her.

**Interviewer**: Were there questions you were asked here?

**Interviewee**: I got worse. I took what was given to me but I throw it, I didn’t take it, I didn’t like it. Then, I didn’t take it here.

**Interviewer**: Is it because you are ordered?

**Interviewee**: I don’t get this kind of medication here. I can’t find that come from there.

**Interviewer**: Do you know anything about the medication? What do they tell you about the medications?

**Interviewee**: Yes, I took it at night and day time. I took it day and night time before, three times a day. I have taken it three times a day but after I went to holy water, thanks to God, I just took at night time only.

**Interviewer**: Did they explain you about the medication?

**Interviewee**: Yes

**Interviewer**: About its side effects

**Interviewee**: Yes

**Interviewer**: What did they tell you?

**Interviewee**: This will take at night and this one at day. Let me show you this, I bring the medication, as you said to speak while holding this

**Interviewer**: Okay, don’t that have side effect?

**Interviewee**: I will bring this everywhere to don’t be lost. This is the one I take, that means this one, there is also another. I will take two from this one.

**Interviewer**: Okay, is it a good medication?

**Interviewee**: Yes, I have been taking this one. I used to take this in the morning and at the night. But, since I went to church, Arsema Mariam, thanks to God I take only at night time.

**Interviewer**: Have you ever forgotten to take medication?

**Interviewee**: *Eee*

**Interviewer**: Have you ever forgotten?

**Interviewee**: I forget sometimes.

**Interviewer**: Okay

**Interviewee**: I forgot when I wake up. I also forget it when I come and it was on the road

**Interviewer**: That you swallowed?

**Interviewee**: Yes, it means that I swallowed it on the road. I swallowed it since I thought I will stay there.

**Interviewer**: Why do you forget?

**Interviewee**: *Eee*

**Interviewer**: Why do you forget?

**Interviewee**: It is my mind. I lost my attention when I talk now too. I forget when I lost my attention.

**Interviewer**: But, there is no other problem

**Interviewee**: Yes

**Interviewer**: Why do you think medication needs to be taken regularly? Why do you think it shouldn’t be discontinued?

**Interviewee**: To don’t die, to don’t fall into water or, it is to don’t fall anywhere, right?

**Interviewer**: Yes

**Interviewee**: Like it is said, it will not be forgotten in that way. I think I may fall in to fire or anywhere.

**Interviewer**: Did the physician ask you about your personal life when you came here?

**Interviewee**: This one?

**Interviewer**: Yes

**Interviewee**: If those who are here say, it didn’t come from there. I told them to give me but it became worse.

**Interviewer**: But they didn’t ask you about your personal life?

**Interviewee**: They didn’t ask.

**Interviewer**: Didn’t they ask?

**Interviewee**: Yes

**Interviewer**: What do you feel if you are asked?

**Interviewee**: Me?

**Interviewer**: Yes

**Interviewee**: Is it not good for you to be asked to don’t do this?

**Interviewer**: Yes

**Interviewee**: *Eee*

**Interviewer**: If they asked you about your personal life and about your family

**Interviewee**: No, I have nothing. Thanks to God, I don’t have any problem, I can go to my family, and I can go to anywhere and bring my meal. I have nothing else.

**Interviewer**: Now you live here in the city, right?

**Interviewee**: Yes

**Interviewer**: Is it difficult for you to follow-up here rather than going to Addis Ababa?

**Interviewee**: I think I will be sick if I don’t get this kind. I am not sick like before and I am worried if I become sick like that otherwise there is no other problem if I follow-up here.

**Interviewer**: What do you think should be done to get better treatment and medication for you to be healthy?

**Interviewee**: I don’t know anything.

**Interviewer**: For example, what this hospital should do in order it to be better for you?

**Interviewee**: I don’t know anything. The one that came from Addis Ababa was not here, that is why I become sick. I am sick because of that.

**Interviewer**: So, you can have follow-up here?

**Interviewee**: Yes, I can have follow-up here.

**Interviewer**: How are they?

**Interviewee**: If they say pay money, I don’t have money to pay since I don’t have anything. Even now, they boy paid ten birr and bring this too. Now, he brought this but I wake up in the morning and forget to bring it. I bought this because they said that you can’t get in without this.

**Interviewer**: What do your children and neighbors say about your improvement?

**Interviewee**: My children take care of me. My children look after me and I will not miss it too. Thanks to God my children are taking care of me.

**Interviewer**: Are they seeing your improvement?

**Interviewee**: Yes

**Interviewer**: Do they say you have improvement and support you?

**Interviewee**: Yes

**Interviewer**: What does it like?

**Interviewee**: Yes, thanks to God. They said *Etetiye* don’t go to church without this.

**Interviewer**: Yes. Both?

**Interviewee**: Yes, they tell me to don’t do that. My children say why don’t you take it in the morning? I will take the afternoon at them but in the past when I went to church. But know I will drink holy water and then will take my medication at night.

**Interviewer**: Okay. What advice would you give to people with epilepsy to improve their lives?

**Interviewee**: Me?

**Interviewer**: There are people like you, right?

**Interviewee**: Yes, there are.

**Interviewer**: What do you think should be done to improve their life?

**Interviewee**: Me?

**Interviewer**: Yes

**Interviewee**: I don’t know anything, but let God gives the right thing to you. I am not the one who can say but it is you who can say that.

**Interviewer**: For example, some people say that if the health care providers treat us well, some people say if the government support us in this way, so if you have something to say

**Interviewee**: The government provides support; I bring this from there since the government provides support, isn’t it? I don’t have anything, I am poor that is why I bring this paper, isn’t it? I will send that paper and they will send it to me, isn’t it?

**Interviewer**: Yes

**Interviewee**: And if God gives, it is good to help things that could be supported. It is common to help those who don’t have anything.

**Interviewer**: What do you think the community should do? You told me as there is discrimination and stigma.

**Interviewee**: Yes

**Interviewer**: What should we expect from the community to improve this?

**Interviewee**: There is nothing to expect from the community. I told you as I run away from the community.

**Interviewer**: Yes

**Interviewee**: I am staying away from them to don’t fight and be upset. I am saying no that is why I staying away from them.

**Interviewer**: Yes

**Interviewee**: What is the problem if the families say let me do this for him. It don’t have problem, right?

**Interviewer**: Yes

**Interviewee**: If the health one supports, but I am sick. It is good if the health one say what should I do my brother and my sister, right?

**Interviewer**: Yes

**Interviewee**: Who gave him this?

**Interviewer**: What about the health professionals?

**Interviewee**: If they say we should give this, right?

**Interviewer**: Yes

**Interviewee**: Who gave this to him?

**Interviewer**: What about the health professionals?

**Interviewee**: It is also the time

**Interviewer**: The time, yes.

**Interviewee**: It is the time, and they get worse too.

**Interviewer**: The health professionals?

**Interviewee**: God bless all of them, I don’t know anything. Those people who are far away who are helping us now and I ask the government if the government aid what it got.

**Interviewer**: If there is anything you want to say that I should asked; let me give you the chance

**Interviewee**: It was good if I asked. Nothing will prevent me to ask my family if I am healthy and have something

**Interviewer**: No, what I said you is that I am finishing my questions and if you have anything you want to tell about your health, treatment and your relationship with your neighbors and family which I don’t asked you

**Interviewee**: Let keep them as they are, they do what they do, they are similar. Thanks to God, you are the one who will protect every time. I told you I have to stay away. They should have say this person is sick and what should we have do for him. They didn’t so this.

**Interviewer**: Okay, thank you very much. I have finished my questions. I know I take your time, thank you.
