# Supplementary material for: Experience and perceptions of mental ill-health in people with epilepsy in rural Ethiopia: A qualitative study
Source: PLoS One. 2024 Dec 13;19(12):e0310542. doi: 10.1371/journal.pone.0310542 (PMC11643256; doi:10.1371/journal.pone.0310542)
Supplement: S3 File — (ZIP) [file pone.0310542.s003.zip › data set/translation 06.docx]

**2010 Dr. Ruth**

**Interviewer**: Thank you very much for your willingness. We will take some time with me; let me ask you some questions first about yourself. Okay, how old are you?

**Interviewee**: Forty one

**Interviewer**: Forty one?

**Interviewee**: Yes

**Interviewer**: Okay, occupation

**Interviewee**: I was farmer but I don’t work now since i can’t. I fear since I will fall and I also afraid to travel and I also don’t like noise. Sometimes I also felt bad when I am in problem. I spent most of my time in good situation. When I become sick of this disease, I went to *Butajira* for medication and then I was referred from *Butajira* to *Tiya* and I was referred from *Tiya* to here. I am following-up the medication here.

**Interviewer**: Did you learn?

**Interviewee**: I learnt up to grade two.

**Interviewer**: Okay. Where do you live?

**Interviewee**: Around *Suten* at *Mitema.*

**Interviewer**: Are you married?

**Interviewee**: Yes

**Interviewer**: Do you have children?

**Interviewee**: I have children but they are not here.

**Interviewer**: How many children do you have?

**Interviewee**: Six

**Interviewer**: Tell me about your illness when it begins you. What type of symptoms did you see when you first went to health institutions?

**Interviewee**: At that time my father died and we went to collect wood to the forest. When we went to the forest, I fall there which I didn’t seen before. Then after a while it is known as it is this disease, I didn’t know anything about it. It was like a dream, I will be conscious when I wake up. I don’t know that I may fall while I am travelling and I can’t avoid it. And then I started taking the medication and I am taking it still. I will take two pills in the morning and in the evening.

**Interviewer**: As you told me, you fall for the first time after your father died; are there symptoms you show after that? What are the symptoms you have beyond the seizure?

**Interviewee**: I felt when I can’t do work, I can’t talk. My sisters are at Addis Ababa and they support me something during holiday. I also have land in the village but I fall many times when I tried to work.

**Interviewer**: Do you have any other symptoms beyond the seizure?

**Interviewee**: I felt when I fall to the ground when the surface hits me. It means, if I fall tonight I will know it tomorrow. I don’t know the feeling too at that time. I don’t know anything. I also encountered accident when I went to Addis Ababa to my sister and work.

**Interviewer**: Is it after you started the medication?

**Interviewee**: While I was taking the medication. I was taking one pills at that time but I am fine now since the dose of the medication increased. But I don’t like noise and anger. It is just like this.

**Interviewer**: What do you thinks are the symptoms of epilepsy? What do you know about this disease, it could be what you heard?

**Interviewee**: I don’t know anything. I just fall; there are also times that I seizure three times at night while I sleep. Then it cut my tongue and lip and the wound will hurt me. But, there is nothing that I know.

**Interviewer**: Do you have any other health problem, for example depression?

**Interviewee**: Yes, the depression is due to work. I used to work hard on farming and trade too before. I felt bad when that interrupted and see other peoples. When I feel like that, I will go to home and sleep. My wife and family are home and she is one who will take care of me when I am sick. I will be conscious in the morning.

**Interviewer**: Did you fall?

**Interviewee**: Yes

**Interviewer**: Other things, for example, do you have things like not talking to others and being alone when you are depressed?

**Interviewee**: Now, it is noisy too when I go to home, it is when I heard noise like mourning at the neighbor as it is rural area not urban. If it was in the urban you can close your door and sit. There are people who left when they have conflict. I will feel and be sick when there are such type of things.

**Interviewer**: Did you have things like suicidal ideation?

**Interviewee**: Suicide?

**Interviewer**: Suicidal ideation or harming oneself

**Interviewee**: No

**Interviewer**: Such type of things

**Interviewee**: I don’t have anything other than working to grow and help my sister.

**Interviewer**: Okay. As you told me, your symptoms are the seizure and becoming sick when you are depressed, right. How these things impacted your relationship with people because of this disease? Tell me in detail about this disease,

**Interviewee**: Yes, it is just this one. There are no other things.

**Interviewer**: You started to say something earlier.

**Interviewee**: My friends and other people don’t approach me, I also felt due to that. I felt bad since everybody including my children doesn’t approach me. I feel bad when my cousin and everybody are far from me and I will be sick. My father and mother are died, I don’t have anywhere to go and I will be more hurt at other place. I felt such type of things in my mind.

**Interviewer**: Be strong. How much the treatment helps you? Is the medication you are taking not helping you?

**Interviewee**: It is very helpful. It will be worse if I don’t take it.

**Interviewer**: Do you see improvements by the medication? Do you have things that you say these things are not changed?

**Interviewee**: No

**Interviewer**: Do you fall after you take the medication? Did you fall in this week?

**Interviewee**: If I take one or two months medication, I will be fine till two months. But I will fall when I am at the top of the mountain and I run out of the medication. I will fall when I don’t come in a month.

**Interviewer**: When you run out of the medication and didn’t take it?

**Interviewee**: Yes, I run out of the medication but the medication is helping me a lot. I will be hurt if there is no medication.

**Interviewer**: What did you do when you are depressed beside the medication?

**Interviewee**: Nothing. Everybody said why you work, you have to simply rest and live with things that you have like anyone else. They told me you will not fall; you have your wife and children. There is nothing, I just live simply.

**Interviewer**: Didn’t you do anything?

**Interviewee**: Yes

**Interviewer**: Are there discrimination of people with epilepsy in your community?

**Interviewee**: Yes. I became Christ when I was sick of this disease and they said you have to be similar as it is rural. There is discrimination in the community and I also left the area.

**Interviewer**: Tell me more

**Interviewee**: Okay

**Interviewer**: About the past, yes.

**Interviewee**: I left from there, she was alone and my children were kid and they can’t work. My children grow up in the town with my sister and my brother started living in the village. He also created pressure on sharing land and I went back again. But, I became Christ because of that.

**Interviewer**: What do you mean by Christ, is it Orthodox?

**Interviewee**: No

**Interviewer**: Protestant?

**Interviewee**: Yes. So, they have such type of pressure but it is good for me if I am there. You can’t do any work if there is discrimination.

**Interviewer**: Are you discriminated because of your religion or your illness?

**Interviewee**: Religion

**Interviewer**: Because of your religion?

**Interviewee**: Yes

**Interviewer**: Was there discrimination because of the disease?

**Interviewee**: Yes

**Interviewer**: Tell me about that. Have you ever been isolated from *Edir* or from other things since you have the disease?

**Interviewee**: Yes, they isolated me from the *Edir*.

**Interviewer**: Is it because of the disease?

**Interviewee**: Yes because of the disease, it is rule. It was told they don’t concern about religion. I felt bad and my mother became old too. At that time, I took her to Addis Abeba to my sister but she came died. Because of these things I felt bad.

**Interviewer**: Did the people isolate you from the *Edir* because of your illness or your religion?

**Interviewee**: It is about the religion, they said he can’t be in our *Edir* if he changed his religion. When they decided that I was at health center and I bring the case to the court. When I saw the things they did to me, I changed my religion. But there was no anything.

**Interviewer**: Did they discriminate you because of your disease?

**Interviewee**: *Eee*

**Interviewer**: Did they ever discriminate since you have the illness?

**Interviewee**: Yes

**Interviewer**: Is that due to the disease?

**Interviewee**: They discriminated me for long time.

**Interviewer**: Why did they discriminate you?

**Interviewee**: Nobody didn’t even pick me up when I fall since they thought it is communicable. They did many things on me.

**Interviewer**: What did you do when they did that to you? What did you do at that time to don’t feel bad when they did that to you?

**Interviewee**: I just did my work, farming and trade. They said person who went to his home will be sick when I tried to recruit daily laborer.

**Interviewer**: Where do you follow-up the treatment now?

**Interviewee**: Here

**Interviewer**: How is it? What does their treatment provision looks like?

**Interviewee**: It is very good. I have card and they gave me the medication immediately when I give them the card. They will give me two months medication to don’t come repeatedly.

**Interviewer**: Do they give you two months medication?

**Interviewee**: I take it from Dr. *Dawit*.

**Interviewer**: Did they ask you about your personal and family life when you came here?

**Interviewee**: Yes

**Interviewer**: How did they ask you?

**Interviewee**: *Dawit* ask me how I am doing and if there are new things, and they ask me as how is the medication. In the past, the medication was one and he made it three types of medication and he also gave me two months medication as I can’t afford the transportation payment. I just take it in the morning and evening.

**Interviewer**: What type of questions did they ask you when you came here first before they gave you the medication?

**Interviewee**: They asked me as from where it was written. I had been taking from *Tiya*, our district was *Tiya* and there was a doctor named *Reta* there. *Buee* is near to me and when I asked them for medication they told me to bring referral paper from there. Then they wrote me referral paper from here and at that time they accepted me. But first I took the medication from *Butajira* *Girar* house. Then from Girar house I went to Tiya district and when I beg them they wrote me referral paper to here. Then I will just come her and take it.

**Interviewer**: Do you always go to the doctor or do you just take your medication from the pharmacy when you come here? Do you send other to take your medication?

**Interviewee**: I will come myself. I will come and go to the doctor. Maybe if he goes to training, they will ask me my name at the office and they bring my card form registry, then they will give me immediately. They will give me immediately and I will just go home and I will come back when it is over.

**Interviewer**: Do you sometimes tell the health professionals about your worried or do you just talk about your illness when you come here?

**Interviewee**: They tell me not to go to the place of mourning, around river, don’t climb on tree; they advise me like that. They also tell to me about the medication.

**Interviewer**: Do they tell you about the medication?

**Interviewee**: Yes

**Interviewer**: Don’t they ask you about your life and thing that you encounter?

**Interviewee**: Yes, as I told you, I am depressed sometimes. I feel depression when I lose something. I will answer them that I am fine and it is my mind.

**Interviewer**: What do you mean by my mind?

**Interviewee**: *Eee*

**Interviewer**: What do you mean by I am fine and it is my mind?

**Interviewee**: It means that it doesn’t hurt me anywhere else, it is only here. They asked me as how many times I fall per day, and then I will answer them that once or twice a day and I will show them my tongue as an example.

**Interviewer**: What do you know about the medications prescribed for you?

**Interviewee**: *Eee*

**Interviewer**: Do you know about the medications that are prescribed for you?

**Interviewee**: I take it as they told me, I will take one pill if they said so and I will take two pills if they said two pills. I just take it as they ordered. In the past, it was one pill in the morning and one pill in the evening and when I told them I am sick, they just increase the dose and changed the medication too. They also told me to take of myself.

**Interviewer**: Do they explain about the medication side effects?

**Interviewee**: Yes. They told me to don’t go to noisy place, don’t drink alcohol and to make myself free. They also told me to take the medication properly on time.

**Interviewer**: Do you have challenges to come here? What do you think are the problems to come here, it could be time or distance?

**Interviewee**: As I told you, I come here when the appointment day approach. They even said come before the appointment day and before you run out of the medication. Unless there is mourning in the village, I will come by transport and bring it on time.

**Interviewer**: Don’t you have difficulties to come here?

**Interviewee**: Yes

**Interviewer**: What did your family, wife and children say about your treatment?

**Interviewee**: They just said don’t stop taking medication. They care about me and they worried about me. She also said don’t wake up early in the morning and take rest. My children are at Addis Ababa and Southern region. There are also children who live here but they are worried about me.

**Interviewer**: What did they say about the improvement you have after the treatment?

**Interviewee**: It is good. They said you are better than the past and you have to take the medication properly. They also said don’t be stressed. As I told you, I used to work since my childhood to support my family. They said don’t worry your children grown up and you have sisters in the town who can help you. I am younger than my sister, the other are older. My mother gave birth for ten children and they cried when I was sick. At that time, I will be stressed but I can’t do anything.

**Interviewer**: Please calm down. Are you taking the medication now?

**Interviewee**: Yes

**Interviewer**: The other question is that people use *khat* and alcohol when they are stressed, do you use such type of things?

**Interviewee**: I used to drink alcohol when I go to the town and I also drink local alcohol at my home. We drink water from spring and I take medication by it that is infectious to but I don’t use any other thing.

**Interviewer**: *Khat*?

**Interviewee**: I never chew *Khat*.

**Interviewer**: How much do you drink alcohol? Do you drink till you become drunk or do you drink at your home or is it when you go to other places?

**Interviewee**: It is when I go to my family and when there is wedding to be similar like anyone else. I drink one or two cup and then I will go to my home. Taking such type of things is bad not only for me but also it is bad for anyone. I also saw elder people drink alcohol when I came to the town and it doesn’t trouble them but I will feel bad when I even smell alcohol. My body dont be comfortable about alcohol.

**Interviewer**: So, what do you think should be done to improve the overall life of people with epilepsy?

**Interviewee**: They have to take care of themselves, they should take medication, and they have to work as they can if they have family who can support them. There is *Eniset* in our area and they can also cultivate cabbage. It is difficult to go to farming area and difficult to travel long distance. I will use car as you don’t know where you are going to fall. So, they have to take care of themselves, you can’t do any other thing as it is not preventable.

**Interviewer**: As individual you have to do the things you told me now. What should the community do to improve the life of these people? What do you think should the community do for people with epilepsy to have better life?

**Interviewee**: There is nothing other than this; you can’t do any other thing in rural area. It is just take caring oneself and improving thought. It is to live up to God wish. There is nothing that you can do. There is medication in this town but there was no such type of thing before. Now there is medication and they can be cured if they take it properly. The main thing is to take caring oneself.

**Interviewer**: What should the government and health institutions do?

**Interviewee**: The health institutions should advice and educate. They have to go and advise them in every *Got*. There are heath extension workers in every place. They said you have to go and get education every two weeks if you have such type of disease. They can also provide the education there; they should provide advice. It is not like the past, there were no physicians at that time. At this time, thanks to God, there is treatment and it is free. The people should go themselves and get the treatment. If they provide education, the people will understand it but they don’t have to be said there is herbal medication and they are being misinformed about this disease. So, the health extension workers should have to provide education for everybody; that is what I think.

**Interviewer**: I asked some individuals questions and if you have something you say I don’t want to miss this thing about the illness. Let me give you the chance if you have something you want to say.

**Interviewee**: What?

**Interviewer**: If you have something to say, I finished my questions.

**Interviewee**: It is just taking care myself to live up to God wish. I have to take care of myself for my children and wife to don’t be worried. That is my choice, there is no other choice and there is no other choice. Where can I go? There is work if I go, many individual go there to rural area. I will be sick if I go there and nobody approach sick people. Even your neighbors will approach you; they don’t want you since you are sick. So, this is my only choice.

**Interviewer**: Thank you very much!

**Interviewee**: Okay

**Interviewer**: I am done. Sorry if I took your time.

**Interviewee**: No problem.

**Interviewer**: Okay.
