# Supplementary material for: Experience and perceptions of mental ill-health in people with epilepsy in rural Ethiopia: A qualitative study
Source: PLoS One. 2024 Dec 13;19(12):e0310542. doi: 10.1371/journal.pone.0310542 (PMC11643256; doi:10.1371/journal.pone.0310542)
Supplement: S3 File — (ZIP) [file pone.0310542.s003.zip › data set/translation 07.docx]

**I:** it’s only you and me. It will be erased after it’s recorded on the tape. We don’t mention your name. You can tell us what you feel. There’s no right or wrong answer for the treatment. We are going to discuss things that are related to you in a way that we can help you. We are health professionals. We are here to discuss things together. Let’s start from you, how old are you?

**Responder:** my age, 20 years

**I:** okay which grade are you?

**Responder:** I am in grade 11

**I:** 11, do you work?

**Responder:** I don’t work that much

**I:** where do you live?

**Responder:** me? Baragote. Baragote 3

**I:** where does it mean? Is it in kela?

**Responder:** Bui, it’s outside kela.

**I:** okay, are you married? Any children?

**Responder:** no I am not married.

**I:** okay my first question is, how did you feel when you first went to the clinic or the health center

**Responder:** when I was sick, I knew nothing at that time. But I was so worried; I didn’t get in touch with anyone. I had a hard time going out and I couldn’t trust myself. And I stress myself thinking that it’ll make me faint. I lost hope in my education and I dropped out of school. I used to get stressed a lot. But now I can go with people freely. I am much better now. The medicine has helped me a lot. I was told to take it after meal and sometimes if I take it a little late, it’s very hard for me. I felt like I was carrying a huge rock and I was worried a lot. But now I am very fine.

**I:** what were the symptoms you first saw? How did it start? When did it start?

**Responder:** I was very young. I was very young. At that time my mother was the one who know I was very young. When I was a kid, I used to come here to take the medication. I mean on the border. I used to take it from butajira until I was eleven years old. If I miss it today; if I miss to take it one day It will make me faint. You have to take it day to day. But this one, which came to Addis Ababa, it won’t make you faint if you miss it today and take it the next day. But you will get a little headache and nothing more. When you feel stressed or when you get stressed, if I take the medicine at that time, I feel nothing; the stress will go away. It’s just like the headache medicine.

**I:** okay so what do you know about epilepsy. What are the symptoms?

**Responder:** the symptoms are first I used to get a headache. You will feel headache then your body’s temperature will rise. At that time when your temperature rises you have to sit or you have to sleep. I feel such kind of things. If my body gets very hot I will sit down quickly because I know it will make me faint. If there is anyone nearby I will call them then they will bring me water. These are the symptoms.

**I:** these are the things. Okay, do you have any other health problem besides the symptom of epilepsy? Is there any other health problem that you think is a little bothering?

**Responder:** no there’s nothing. There’s nothing else. It’s just that which is bothering me.

**I:** that’s bothering you. How long has it been since you started taking the medicine?

**Responder:** the medicine from Addis Ababa or?

**I:** that you took since you were a child.

**Responder:** yes, since I was a child. I started taking since I was a child.

**I:** have you ever stop taking the medicine?

**Responder:** I quit. I used to quit that’s why it stayed for long time. If I didn’t quit it I would be fine. I started to walk freely after I get the treatment in Addis Ababa. The medicine from Addis Ababa helped me a lot. Now that they refer me to kela from butajira, we are paying a lot but hey refer me to kela.

**I:** okay are you getting the treatment from kela now.

**Responder:** yes in kela.

**I:** how is the treatment? Does it help you?

**Responder:** it really helped me. It helped me because it was in kela. I was so worried to even come to Addis Ababa. I was so worried thinking that there might not be the medicine. I was so worried. But I go to kela on my own and they give me. The medicine is really helpful to me.

**I:** is there any problem that you experience due to this disease? It might be on your education or on your relationship with your family. And can you say I lost these things because of this disease?

**Responder:** no there’s nothing.

**I:** for example: I could’ve been there but I couldn’t because of this illness, I could’ve done that but couldn’t because I am sick. Is there such kind of think that you can mention, I mean the impact of exposure to this disease?

**Responder:** actually it’s been a long time since started learning. But when I was in grade one to eight. I would’ve joined university by now. I would’ve been third or fourth year. But I was never ashamed and I never felt less from my friends when I was learning. I would’ve joined university by now, I would’ve gotten a job by now, but I was not educated the same level as my friends and the reason is that.

**I:** you consider this as a reason

**Responder:** yes

**I:** what about your relationship with your families and friends other than your education.

**Responder:** no there’s nothing like that.

**I:** is there any discrimination or stigma in your area or in you because of this disease?

**Responder:** they used to do that before and there was a girl who works in health center. A girl who works at the health center, you know her, her name is Tenagne, she speaks to them. And after she speaks to them they kept quiet. But before that, they were worried when I was with them. They think I was going to get sick. They will leave if I go to them. I was very ashamed and i used to get angry then. But now it’s okay.

**I:** when they do this in the past, were there thing that you used to do in order to control your anger and frustration?

**Responder:** I used to go home and sleep. When I was angry, I used to in and sleep. I will tell my family that I am sick. I keep things on my own, I won’t tell other people most of the time. Even if someone insults me I will not tell anyone. I will keep it on my own and let it pass. When I get stressed, no one understands me most of the time, I mean people that are outside our home. My mother is the one who understands me and with my father we’re not that much. We don’t have much to work these days so I don’t get stressed that much. I used to get stressed a lot before but now I am fine.

**I:** is there any symptom that you want to go away?

**Responder:** symptom. There is no symptom after Addis Ababa. It doesn’t show any sign. I told you that it used to make me stressed but now there’s nothing that makes me stressed. I even forgot what I thought would make me sick if I was angry. I am just taking the medicine. I even forgot that I have the disease. The medicine is making me forget it and I am learning happily.

**I:** okay, great. Sometimes when people are stressed or when something goes wrong, they get themselves in different kind of addictions in order to avoid the stress. And is there any kind of things that you use like alcohol, cigarette or khat?

**Responder:** no. I don’t even like coffee since I was little. I don’t even drink coffee I really hate it. I have friends who are using it after they failed in grade 10. They carry khat with them always. But I want to teach them but they think the other way. You have to do something to make them understand. But I can’t do anything whether they understand or not.

**I:** okay let’s talk about the things you get in the health center. What was it like? How do they greet you when you go to kela? Do you just go to the pharmacy or do you meet with the health professionals?

**Responder:** yes they ask me just like you are doing. Then they'll give me. They’ll write and give me

**I:** you told me you had it since you were a kid. at a time when you remember, when you went to the health professionals what kind of questions did they ask you? About your feelings for example: you are adult, there might be some feelings that you feel. And also it might be about the relationship with your friends or your families, you might have things to worry about: your education might stress you, your future might stress you, some problems at home might stress you, there are many things that might get you stressed and have you ever discussed about this issues with the health professionals?

**Responder:** They have never asked me like this in the health center. They asked me about myself. I mean just about the illness. There are a lot of times that i wanted to tell them about my feelings if they asked me about it but they ask me only about my illness and they write for me and go. But i wanted to tell them about me and let it out. but at that time they do not ask me. They just ask me about my wellbeing. they ask," how was it, how are you feeling" and so on. Then they tell me to wait outside and give me the medicine and they'll go. But i would be happy if i told them about me at that time. But it might be because of the people that are waiting for them, i don't know.

**I:** do you go there often? If they ask, what do you think the solution would be? In our country there are some people that don’t like to be asked. And there are some who wanted to be asked so that he can tell. And there are some who tell everything without being asked just to let it out. How do you think it's important to discuss about your personal life?

**Responder:** If they didn't ask and if they mention it a little, i will tell them. I think that I should talk about it to let it out but I don’t have to be asked. People ask you and want you to tell them about your life but i would be happy if i tell him and he understands me. But I wouldn’t be happy if he listens and kept quiet after i told him. I would be glad if he agrees after i told him about my life.

**I:** okay, sometimes you just want to find someone to talk to, and then you go home and worry about what to do and spent the night trying to find a solution. What are the things that you did to get rid of your anxiety? if you have any from your past experience.

**Responder:** I do that many times. If i get stressed over my education I'll tell my sister. She’s the one who understands me at home. She’s older than me. She understands me a lot. She went to kela to work and i have no one to talk to so i get stressed a lot. And my friends don't understand me most of the time so i keep quiet. I'll pass it on my own quietly.

**I:** you said that it would be better if they ask you such things in health center and what do you advise to improve this service for another time. We all work to improve these services.

**Responder:** I would be happy if other people, who are like me, don’t suffer like I did. I want to tell my story and discuss about it and i would be glad if they can help them too. There’s a guy who's sitting there. His name is melaku, he's black. We learned together a lot of times. But I got shocked when he got sick. i think of myself. I took him to his house many times. i let him sleep at my place because i go to his many times. But I think of myself whenever I see him. I get worried wondering how someone would be in trouble if they were in my shoes. But I would be happy if they don't do that. I will ask them and I would be happy if there's a solution to it.

**I:** so you are saying it would be better if the health professionals to discuss and to talk to such people about themselves

**Responder:** yes, there will be a solution

**I:** okay, do you know about the medicines that are prescribed for you? Do you talk about their side effects?

**Responder:** yes, you can never use medication other that what's prescribed for you. I was told to take after meal; if i use it before meal it will hurt me. It’s both useful and harmful. I take it because it benefits me. i was told to take after meal so i take it after i eat food. If I don’t take it after meal I will get sick and I'll lose myself. The medicine will harm me. I think it will harm me.

**I:** do they tell you about the medicines? What kind of medicines you are taking. There are some side effects of the medicine. Some of it hurts your stomach and some people don’t know about it and they'll say "my stomach ached" after they take the pill. Such kind of things that are related to the medicine like it shouldn't be stopped and so on.

**Responder:** yes they told me. They told me not to stop. At first they told me that if I don’t take the medicine, if I don’t take it regularly, I will get sick. They said it hurts so you should take it regularly. But they didn't say anything more.

**I:** how much do you ask and talk to the health professionals about what you want to know? for example: you want to know many things. You are a student so you are at age where you can read and understand. If you have any questions regarding your illness, do you communicate openly with the health professionals to ask them questions like "why does this happen"?

**Responder:** When i ask? i have a great desire to ask many questions but you might not get a person who you can ask. You don't feel good to ask them there in kela. They work very fast and it won't be good if it is done quickly. But if I want to ask there's a guy whose name is Abera. i like him. He answers me most of the time. He talks softly, he tells me while entertaining me. But i don't have the courage to ask the others. They won't be there so I don’t ask them. There’s a doctor whose name is Abera, i ask only him.

**I:** okay, have you ever forgotten to take your medicine.

**Responder:** I’ve never forgotten. If I have to go somewhere far, I'll take it with me.

**I:** why do you think the medicine shouldn't be stopped?

**Responder:** Because it will hurt me. At least if i am hurt my family will too. It’s not just me I have to think about my family too. If I quit it will be just a waste of time for time; if I quit it will just be a waste of time for the doctors. They get worried to do the treatment and to give me the medicine but it shouldn't be just about me, I have to think about all of them. I will never stop it. I have never left without the medicine, I'll take it everywhere. I always take it at night so I have to be sure in case i spend the night outside. So I take it everywhere

**I:** okay, is there a problem with coming to kela or the health center, when you come for a regular checkup

**Responder:** no, there's no problem.

**I:** regarding time, education or distance and so on.

**Responder:** it's not that far. In school they might call us for tutorial but its known which one I give priority to at that time. This is life and that's life too but my life comes first. I want to give priority to my education, I doubt sometimes but my treatment should come first.

**I:** what does your family say about the treatment that you are getting at this level?

**Responder:** my family, i don't usually agree with my father. I have ear problem and he doesn’t understand when they told me. He thinks of himself. Most of the time people who have ear problem don’t understand. They are happy for me, everyone in the neighborhood was very happy, they were happy and even for myself

**I:** what do you think should be improved with this treatment? What do you think about the things that should be done in order to improve the lives of people especially for people with epilepsy? For example: if you have something to say about the things that an individual should improve so that he will live longer.

**Responder:** he knows how to improve his life but if he is using his medicine, if he is using it regularly then nothing else is needed. I mean if he takes care of himself. If he is taking care of himself, he should use the medicine. He needs to have support from his family. He shouldn't be angry. If his neighbors understand him, he doesn't need to be rich, he just have to take care of himself. It shouldn’t be like that i mean there's a guy who has grown up rich but is suffering. He’s in our neighborhood but they took him to Addis Ababa and he hasn't come yet. They say we're taking the treatment there so they didn't bring him here still. We talk on the phone but I choose him. He started with me. But I am fine and I'm walking by my own, I am learning with my friends. But if they take their medicine well, then that's good.

**I:** okay as a community, what do you think the society should do in order to improve the lives of people with epilepsy? What should the society help with?

**Responder:** the society, I think they'll listen if they can assemble them and tell them about the treatment. in addition to that, I think something different would come up if the doctors gather all the people and talk to them at the health center; I think they would all agree.

**I:** what should they talk to them? What is the society's problem or what can the role of the society do for the people?

**Responder:** the doctors may know about the patient but the community doesn't know anything about the illness. They think it's just normal pain, they don’t know its serious illness: I mean the community. But they can understand gradually. If they are told and if they are given advice, I think it would be different. I would be happy if they are told about the disease.

**I:** okay, what should health centers do to improve the lives of the people who are ill? What should health centers and health professionals do?

**Responder:** it would be great if a branch is opened just for this disease. I mean something different. If that’s done, there are people who are in pain but doesn't tell, who are suffering and there are some people who are using the medicine only and there are some who cannot even go to bring their medicine but if everything is opened in health care it would've been great. it would also be near for us.

**I:** can opening something of its own benefit the patient?

**Responder:** it has a great benefit. He might not understand the patient just by going to bring him from far if he has many people around him. a person with that disease can go to his grandfather or try to go elsewhere. And everyone might understand. People understand each other by seeing one another and maybe they can at least protect themselves.

**I:** okay, i have limited questions. There is no related stress or any other disease or symptoms so my questions are short. But is there anything that you want to tell me that i didn't mention? If we can improve such things because you said you go through different things, you might go through it. There are many different types of problems and if the treatment you are giving us is centered on this, is there something you would like to add to this?

**Responder:** I don’t think i have something different to say to this but I am happy on the way that you are treating us. but since you are only in kela, outside, I came from kela. But I don't know Bui that much. I came to Bui just once. We came here to take matric exam and thats how I come to the hospital. I came here by asking people but I would be happy if it's in kela. I don't know Bui, it's not just me people may not know it too. It would be great if it's in kela. There are many people around our area who have this illness; it would be nice if it is in kela even if it's on the hill.

**I:** we are doing our research in sodo woreda and include other woredas too like kela, bui, tiya and so on. So today it is the conveyance that came here, but there is another one there too. I've finished my questions. If you want to say something; if there's something that I didn't ask you, let me give you a chance.

**Responder:** there's nothing else

**I:** okay.
