# Supplementary material for: Experience and perceptions of mental ill-health in people with epilepsy in rural Ethiopia: A qualitative study
Source: PLoS One. 2024 Dec 13;19(12):e0310542. doi: 10.1371/journal.pone.0310542 (PMC11643256; doi:10.1371/journal.pone.0310542)
Supplement: S3 File — (ZIP) [file pone.0310542.s003.zip › data set/translation 08.docx]

**Ruth round 2 012**

Interviewer: Thank you very much for taking your time and come here. Let’s start by asking you some questions. There is no need to mention your name and you code is eleven. How old are you?

Interviewee: I am twenty three years old.

Interviewer: Twenty three. Do you have work?

Interviewee: Yes

Interviewer: What do you work?

Interviewee: Farming

Interviewer: Farming?

Interviewee: Yes

Interviewer: To what grade did you learn?

Interviewee: Up to ten

Interviewer: Where do you live?

Interviewee: *Gogete* *Sosit*

Interviewer: Is it around *Kela*?

Interviewee: No, it is around *Sibisti*.

Interviewer: Okay. Are you married?

Interviewee: No, I am not married.

Interviewer: Okay, do you have children?

Interviewee: I don’t have.

Interviewer: Okay, my first question is that when the illness did begins you?

Interviewee: It is around October and February.

Interviewer: Last year?

Interviewee: No, it has been a while.

Interviewer: How many years?

Interviewee: At least twelve years.

Interviewer: What did you feel when this illness begins you first?

Interviewee: I don’t know anything when I was sick. I was with my friends and don’t remember when I fall. I became conscious after I went to my home. I found myself at home after they took me.

Interviewer: What other symptoms did you show after that?

Interviewee: When I am going to seize, it will tie my mouth and after a while I will fall.

Interviewer: Do you have any other health problem other than the epilepsy?

Interviewee: No

Interviewer: What do you know about epilepsy disease symptoms?

Interviewee: I don’t know anything.

Interviewer: *Eee*

Interviewee: I don’t know.

Interviewer: There are symptoms that you have and if you have anything to say that this disease have these types of symptoms that you know and heard, it could be what you heard from others or what you see from others or from yourselves.

Interviewee: I heard as this disease leads to loss of memory after a while. It has nature of loss of memory but I don’t encounter that yet.

Interviewer: Okay. Do you have symptoms of depression, hating to be with other peoples and else?

Interviewee: Yes

Interviewer: Tell me about that.

Interviewee: I will be sad.

Interviewer: Okay

Interviewee: I will be sad but I don’t have depression.

Interviewer: What is depression? How do you feel?

Interviewee: It is like sleep. I just have that.

Interviewer: Do you fall asleep?

Interviewee: Yes

Interviewer: What else? Do you feel tired when you wake up in the morning?

Interviewee: Yes

Interviewer: What else?

Interviewee: I wake up at three o’clock in the morning. My family also told me to don’t wake up early. They don’t want me to wake up in the morning. It is just like that.

Interviewer: But, do you want to wake up early in the morning?

Interviewee: No

Interviewer: *Ehh*

Interviewee: I will not be depressed when I work.

Interviewer: *Ehh*

Interviewee: I will be depressed when I went to movie house and then I will go back to home.

Interviewer: What do you do when you are depressed?

Interviewee: I don’t do anything.

Interviewer: *Ehh*

Interviewee: I will go to work.

Interviewer: What about when you go to there?

Interviewee: I will not be sick.

Interviewer: No, the depression?

Interviewee: The depression will go away?

Interviewer: Will it go away?

Interviewee: Yes

Interviewer: For example, sometimes when people are depressed they don’t want to join others or they may not like to do work; is yours like that?

Interviewee: No, I like to play with other individuals much.

Interviewer: *Ehh*

Interviewee: I like to join other people.

Interviewer: *Ehh*

Interviewee: I will be depressed when I am alone.

Interviewer: Okay. Does this illness have impact on your life?

Interviewee: Yes, once I wake up in the morning and I fall. It was around august which I fall in front of our home. I fall on the stone and I lost my ear. I feel pain on my ear now. It is just that. I can’t hear well, I hear a little bit.

Interviewer: Is it your left ear?

Interviewee: Yes

Interviewer: Okay, what else did it impact on your life? For example, did it change your thought, or do you have things like giving up?

Interviewee: No. in the past I cut my tongue when I fall.

Interviewer: *Eee*

Interviewee: But I don’t at this time.

Interviewer: The thing you encountered due to this disease, for example, you told me about physical things.

Interviewee: Yes

Interviewer: You told me as you cut your tongue, do you have things that you think lost that you may achieve, it could be on education?

Interviewee: No, there is nothing. I stopped my education by my own reason.

Interviewer: Is not related to your illness?

Interviewee: It is not.

Interviewer: Did the illness have impact on your work?

Interviewee: There is nothing.

Interviewer: Is there nothing?

Interviewee: There is nothing.

Interviewer: Okay. Where do you follow-up the treatment?

Interviewee: It is on the health center.

Interviewer: Which health center?

Interviewee: Yes

Interviewer: Where?

Interviewee: *Kela*

Interviewer: How was the treatment at *Kela*?

Interviewee: It was good.

Interviewer: What did they tell you about the treatment?

Interviewee: There was medication that they will give me. I have brother at *Butajira* hospital and he is the one who will send me the medication from there.

Interviewer: Did you stop the follow-up at *Kela*?

Interviewee: Yes

Interviewer: Why?

Interviewee: When I fall repeatedly last time.

Interviewer: *Ehh*

Interviewee: Then when I told him that he told me to try this one. Now, I tried that and I am at good situation.

Interviewer: When did you fall repeatedly?

Interviewee: It has been a while.

Interviewer: How long?

Interviewee: It has been a year.

Interviewer: When did you fall for the last time?

Interviewee: For the last time, October.

Interviewer: When did you fall recently?

Interviewee: October

Interviewer: This October?

Interviewee: Yes

Interviewer: While you were taking the medication?

Interviewee: Yes

Interviewer: Why do you think that happened?

Interviewee: I didn’t take the medication at that time.

Interviewer: Didn’t you take the medication?

Interviewee: I went to holy water. I stayed a week for a week.

Interviewer: Did you stay a week?

Interviewee: Yes. I didn’t have anything there but I fall repeatedly when I went back to home. I fall on the first and second day and then I started taking it.

Interviewer: The medication?

Interviewee: Yes

Interviewer: Have you ever fall after that?

Interviewee: No, I don’t.

Interviewer: Don’t you?

Interviewee: Yes

Interviewer: What did the health professionals tell you about the medication at the health center?

Interviewee: They don’t tell me that much about the medication.

Interviewer: Why?

Interviewee: He is the one who bring it.

Interviewer: Your father?

Interviewee: Yes

Interviewer: Why don’t you go?

Interviewee: No. he will say I will go and bring it.

Interviewer: Don’t you want to go to health center and tell the doctors what you feel?

Interviewee: I don’t want.

Interviewer: Why?

Interviewee: No

Interviewer: Explain it to me; it is only you and me that are here. You can consider me like your sister and tell me what you feel. We come here to make you get better things. Did you see gap at the health center?

Interviewee: No, there is nothing. There is no gap, just that medication not comfortable for me.

Interviewer: Which they gave you?

Interviewee: Yes

Interviewer: Why don’t you tell them that the medication wasn’t comfortable for you? Why didn’t you ask them to explain about the medication behavior or why didn’t you ask them to change the medication? Didn’t you have close relationship?

Interviewee: Yes, I don’t have close relationship. And when *Abera* called me said tried this one, I said okay and took it.

Interviewer: Were the medication that he gave you and they gave you different?

Interviewee: Yes

Interviewer: Did you get improvement then?

Interviewee: Yes

Interviewer: Consulting at health center, for example, it is not only about medication but they may teach you how to take care of yourself and they may teach you different things; don’t you want to get such type of services?

Interviewee: No, I want. My brother also advises me, the advice is good. Since he advises me , I don’t have intention to go there.

Interviewer: To go to the health center?

Interviewee: Yes

Interviewer: What did you relationship with the health professionals looks like when you went to the health center before?

Interviewee: It was good but we didn’t meet every time, they just bring me the medication. I think it was once that we met.

Interviewer: What did you talk at the time you met? What did they ask you?

Interviewee: They asked me as what did you feel when you are sick, and then as what did upset me. He asked me if I had anger and I told him I didn’t. They also asked me if I worked a lot and told them I don’t.

Interviewer: Did they ask you this?

Interviewee: Yes

Interviewer: What did you feel when they asked you that?

Interviewee: It was good.

Interviewer: *Eee*

Interviewee: It was good advice.

Interviewer: Was it good advice?

Interviewee: Yes

Interviewer: The, you stopped going there?

Interviewee: Yes

Interviewer: What did make you to don’t go there to get more advice?

Interviewee: There is nothing, I just don’t think going there.

Interviewer: What is your health status now?

Interviewee: It is good.

Interviewer: When did you fall? Did you have any symptoms? Do you have symptoms on you body?

Interviewee: No

Interviewer: Feeling angry

Interviewee: No, there is no such type of thing.

Interviewer: Okay. Sometimes people may be addicted when they are depressed due to different reasons, it could be drinking alcohol, chewing *khat,* or smoking; which one do you use? Do you chew *khat*?

Interviewee: No, I don’t.

Interviewer: Things like this

Interviewee: I don’t.

Interviewer: Okay. How do you see the treatment service at the health center? Are there things that should be improved?

Interviewee: Improvement?

Interviewer: Are there things that should be improved at the health center?

Interviewee: No, there is nothing.

Interviewer: Okay. Who did help you when your first went to the health center?

Interviewee: It was my father.

Interviewer: Did you go with your father?

Interviewee: Yes

Interviewer: What did the health professionals at the health center asked you?

Interviewee: It was about the medication.

Interviewer: Okay. What did they tell you?

Interviewee: They told me to take it after meal.

Interviewer: What else?

Interviewee: Other, to don’t be upset, to don’t work much and to don’t worry much, they told me such type of things.

Interviewer: Did you ever forget to take the medication?

Interviewee: I didn’t.

Interviewer: Did you ever miss medication?

Interviewee: Yes

Interviewer: Why did you stop taking the medication last time/

Interviewee: I went to holy water.

Interviewer: Wasn’t it possible to take both?

Interviewee: It want possible.

Interviewer: Who told you as both can’t be taken together?

Interviewee: It was me who decided that.

Interviewer: Was it you?

Interviewee: Yes

Interviewer: Why do you think the medication should not be discontinued? Why do you think it is said that the medication should not be discontinued?

Interviewee: It is said that the medication should not be discontinued since the disease will relapse.

Interviewer: Okay, what else? How much improvement did the medication you take bring to you?

Interviewee: It is good.

Interviewer: Tell me about the improvement that the medication brings to you by comparing before and after you start taking it.

Interviewee: In the past, I fall every month. I fall every Friday or Saturday, but I don’t fall like that now or I will fall if I miss the medication.

Interviewer: What about now?

Interviewee: Now it is good. I am at good situation if I don’t discontinue.

Interviewer: What do you feel if the health professionals ask you about your personal and familiar life when you go to health center?

Interviewee: I feel happiness.

Interviewer: *Eee*

Interviewee: I feel good things.

Interviewer: What do you think is the importance of asking such type of question?

Interviewee: What?

Interviewer: Most of the time you discuss about your personal and familiar life, right?

Interviewee: Yes

Interviewer: What do you feel if they ask you about your personal life?

Interviewee: I feel good thing.

Interviewer: For example, some people will say why when they are asked about their personal life and others peoples say I feel relieve when if they ask me and told them about my worry and some people also will tell them even though they are not asked; so, from which one are you?

Interviewee: I am happy about my family.

Interviewer: If you talk to the health professionals every time like your friends about what you feel.

Interviewee: Yes. Most of the time I am not depressed but I will be depressed when I don’t do work. But I am happy about my family.

Interviewer: Are you happy?

Interviewee: Yes

Interviewer: That is the main thing. What do you think should be done for people with epilepsy for them to have better life? What should they do for themselves?

Interviewee: They have to follow-up the medication and I advise them to don’t use alcohol, chew *khat* and smoke cigarette. In addition, they have to make themselves happy.

Interviewer: What should the community do? Are there discrimination and stigma due to this disease? Are there discrimination, it could be from the family or from friends or from the community since you have the disease? There are such types of things in the community, right?

Interviewee: Yes

Interviewer: Did you experience such type of things? Tell about your experience?

Interviewee: I didn’t experience such type of things.

Interviewer: *Ehh*

Interviewee: My friends like me much.

Interviewer: *Ehh*

Interviewee: I just say there should be no discrimination.

Interviewer: Is there discrimination by the community?

Interviewee: Some people say this disease is communicable and don’t touch me.

Interviewer: What else?

Interviewee: They say don’t be close to me and some people also say don’t be near to him he has the disease. There are people who say that but I advise for such type of things to don’t exist.

Interviewer: Did you encounter such type of person?

Interviewee: I didn’t.

Interviewer: Okay. What do you think should the health professionals do?

Interviewee: They should go every month and provide advice. They have to gather people and advise them.

Interviewer: Whom?

Interviewee: Those people who are sick.

Interviewer: Sick people?

Interviewee: Yes

Interviewer: Okay. What should they advise them?

Interviewee: They have to ask them about their health, as they are taking the medication and they have to ask if there are new things after a month.

Interviewer: What is the importance of asking such type of questions for the patients?

Interviewee: New things may come for the patients. They may be completely cured if they properly take medication. ………22.26, it just that.

Interviewer: Do you have anything else you want to say which I don’t ask you?

Interviewee: No, there is no.

Interviewer: The thing that you tell me about your current health status.

Interviewee: I am at good health status.

Interviewer: Are you?

Interviewee: Yes

Interviewer: Okay, thank you for giving your time and discuss with me.

Interviewee: Thank you.
