# Supplementary material for: Experience and perceptions of mental ill-health in people with epilepsy in rural Ethiopia: A qualitative study
Source: PLoS One. 2024 Dec 13;19(12):e0310542. doi: 10.1371/journal.pone.0310542 (PMC11643256; doi:10.1371/journal.pone.0310542)
Supplement: S3 File — (ZIP) [file pone.0310542.s003.zip › data set/translation 09.docx]

**P009**

**Interviewer**: Okay, thank you very much for your willingness. Your code is nine. Okay, what was the problem that brought to the health center for the first time?

**Interviewee**: For the first time?

**Interviewer**: Yes

**Interviewee**: When I seize?

**Interviewer**: Increase your voice.

**Interviewee**: I seized a lot of time,

**Interviewer**: Did you seize?

**Interviewee**: Yes, when I seized I didn’t have anyone on my side. Someone child was sick like me and he was at *Butajira*. His child was being treated and there was occasion when there was no medication due to that I knew him at *Butajira* and he said I will bring him to *Butajira*. That man took me to *Butajira* and I started the medication there. After that, I didn’t seize miraculously.

**Interviewer**: Didn’t you seize?

**Interviewee**: Yes, I don’t seize since, in the first place, the physician told me about the medication and I took it properly. I was not here, I live in rural area. I didn’t use alcohol since I had to use it according to the information they gave us. There was a lot of children there and it is not that much helpful for them since they drink alcohol and they don’t care much about their health. But it helps me and I haven’t seen anything after that.

**Interviewer**: Is it not useful?

**Interviewee**: It is useful for me.

**Interviewer**: Okay, what is it?

**Interviewee**: Since I took it properly, I don’t use alcohol drinks.

**Interviewer**: Don’t you use?

**Interviewee**: Yes. The health professionals advise me, especially when I come to take the medication. So, I use it properly.

**Interviewer**: You told me as you seize and they took you to hospital at that time, right?

**Interviewee**: Yes

**Interviewer**: What other ill ness did you have?

**Interviewee**: At that time?

**Interviewer**: Yes

**Interviewee**: I didn’t know anything; I didn’t know myself for more than an hour.

**Interviewer**: Did you lose consciousness?

**Interviewee**: I simply lose consciousness and I fall at anyplace car, fire or water. I don’t know myself when I seize and I will wake up after one to two hours. Thanks to God I have good improvement now.

**Interviewer**: Were there other symptoms in addition to that?

**Interviewee**: Something like

**Interviewer**: Foaming?

**Interviewee**: Yes

**Interviewer**: What else do you this illness?

**Interviewee**: *Azurit*

**Interviewer**: *Azurit*?

**Interviewee**: Yes, it is *Azurit*.

**Interviewer**: Okay, besides the epilepsy, you just said seizure and foaming are signs of epilepsy

**Interviewee**: Yes

**Interviewer**: What else? Does it have other symptoms?

**Interviewee**: Shaking

**Interviewer**: Shaking?

**Interviewee**: Shaking

**Interviewer**: Did you have like that?

**Interviewee**: Yes

**Interviewer**: Shaking?

**Interviewee**: Yes

**Interviewer**: Okay

**Interviewee**: At that time, my body shakes and I didn’t know it. It just shakes me a lot.

**Interviewer**: Did it shake you?

**Interviewee**: Yes

**Interviewer**: What did they do to you when you were like that?

**Interviewee**: They took me to shadowy place. It wasn’t like this at that time, people were very afraid to pick you up.

**Interviewer**: Why are they afraid?

**Interviewee**: They think it is contagious disease. People have such type of fear since they are human being. So, there will be such type of fear and I also fear like them. I thought it will be transmitted to family and I was terrified.

**Interviewer**: Don’t you want them to touch you?

**Interviewee**: Yes, I didn’t want to. I lost consciousness simply at that time and then I thought it would be something that would be transmitted and hurt them.

**Interviewer**: Yes. At what age did it start you?

**Interviewee**: I was kid.

**Interviewer**: How old were you?

**Interviewee**: I was very kid.

**Interviewer**: Do you remember how old were you?

**Interviewee**: I don’t remember; it has been a while.

**Interviewer**: Yes, it has been a while.

**Interviewee**: It has been a while since I started using the medication.

**Interviewer**: Yes. What do your family told you when you started it?

**Interviewee**: I know at the time when it started me a little bit. I used to work at another person house. I was servant and I seized while preparing coffee, I just never seen that before.

**Interviewer**: Let’s start from where we stop. Did you say I was preparing coffee? Okay

**Interviewee**: Yes, then I found the two. It was very painful when it hit me, and then it turned me around and threw me away. I wasn’t sick before that and then I became sick.

**Interviewer**: Did it start you after that?

**Interviewee**: Yes

**Interviewer**: Did your employer take you for treatment or someone else?

**Interviewee**: No, someone else, my family can’t afford it. It was my grandmother and she could not see. It was the other guy since he took his child for treatment.

**Interviewer**: Is he a neighbor?

**Interviewee**: No, we didn’t meet. 0619…………… the man took me since he know as I am sick and his child also sick like me, but we don’t know each other.

**Interviewer**: Did he hear as you seize and take you?

**Interviewee**: Yes, it is like that. Then he directly took me to *Butajira* and examined me and I started medication.

**Interviewer**: Did you start follow-up by yourself?

**Interviewee**: Yes

**Interviewer**: Okay, good. Don’t you remember how old you were?

**Interviewee**: At that time, at least

**Interviewer**: How many years will it be? For example, how many years will it be?

**Interviewee**: It could be around twelve years.

**Interviewer**: Could you be twelve years old?

**Interviewee**: Yes

**Interviewer**: After how long did you start the treatment?

**Interviewee**: Treatment, I waited for at least a year.

**Interviewer**: Had you been not treated for a year?

**Interviewee**: Yes, I had not been treated.

**Interviewer**: Did you stay since you didn’t know as it had treatment?

**Interviewee**: There is no family who will remember me. That person was like a messenger of God and that is why I could be diagnosed. It is not possible to get treatment; *Butajira* was a big deal for me.

**Interviewer**: Yes

**Interviewee**: So, I don’t know, it is hard for me.

**Interviewer**: Yes

**Interviewee**: That is it.

**Interviewer**: Okay, what else? Do you have any other mental illness besides the epilepsy?

**Interviewee**: I don’t have.

**Interviewer**: What other health problem?

**Interviewee**: There is nothing.

**Interviewer**: Is there nothing? For example, it could be drinking alcohol or addiction

**Interviewee**: There is no such type of thing.

**Interviewer**: Didn’t they say you depression at the hospital?

**Interviewee**: They didn’t say.

**Interviewer**: Did you take medication for the epilepsy only?

**Interviewee**: I don’t take other medication. Sometimes I will come here and treated when become I am sick of other illness.

**Interviewer**: What do you mean by other illness?

**Interviewee**: It means, like common cold.

**Interviewer**: Like typhoid?

**Interviewee**: It was only typhus; I don’t have any other illness.

**Interviewer**: Don’t you have any other medication that you take continuously, like depression?

**Interviewee**: There is no.

**Interviewer**: Is it only the epilepsy?

**Interviewee**: Yes

**Interviewer**: Okay, so do you only have epilepsy?

**Interviewee**: Yes

**Interviewer**: Okay, let’s talk about the epilepsy disease.

**Interviewee**: Okay

**Interviewer**: You told me that in your community epilepsy is called *Azurit*, right? Does it have impact on your life since you are epilepsy patient?

**Interviewee**: Yes, I told you that it was at the beginning. I was worried not for me but for the others. I was worried that it might be contagious.

**Interviewer**: Did the epilepsy have impact on your work?

**Interviewee**: There is no such type of thing.

**Interviewer**: Have you ever been said we don’t recruit you?

**Interviewee**: No

**Interviewer**: You told me as you are daily laborer, so was there time that you had been said you can’t work that?

**Interviewee**: There is no such type of thing.

**Interviewer**: What about on your education? Didn’t you ever stop your education?

**Interviewee**: Education, the problem is the family capacity. My grandmother also was supported by me as there is no one. My mother is also psychiatric patient and I am the one who buy medication for both of us.

**Interviewer**: What kind of mental health problem do she had?

**Interviewee**: She also takes the monthly medication.

**Interviewer**: Is it the epilepsy one?

**Interviewee**: No

**Interviewer**: Another?

**Interviewee**: Yes. She used to lives alone but now she lives with us. Thanks to God, she take medication every month like us.

**Interviewer**: Isn’t it epilepsy?

**Interviewee**: Yes, there are people who beg on the road, right?

**Interviewer**: Yes

**Interviewee**: She was like that, but thanks to God she is fine now. They are also following her, but I don’t know as what it is. She is taking medication every day like me.

**Interviewer**: Do she take medication?

**Interviewee**: Yes, it is because of that I stopped my education.

**Interviewer**: Okay, did it have impact on your social life? Social life means, things like discrimination and stigma since you have this disease

**Interviewee**: There is nothing.

**Interviewer**: For example, not meeting friends

**Interviewee**: I understand you, there is no such type of thing.

**Interviewer**: Is there no such type of thing?

**Interviewee**: I didn’t experience such type of thing.

**Interviewer**: At mourning, wedding

**Interviewee**: There is nothing.

**Interviewer**: Did they know as you have the disease? Did people and your friends know as you have the disease?

**Interviewee**: There is nothing I am ashamed of. For example there are many type of disease and I don’t say I have this disease.

**Interviewer**: Don’t you say that?

**Interviewee**: Yes. It is about using what is good for me but I don’t feel whether someone knows about my disease or not because everyone lives their life and take of their health. It is meaningless if not implemented even though the health professionals educate.

**Interviewer**: Yes, I asked this because it is when people know they will ignore you.

**Interviewee**: There is no such type of thing.

**Interviewer**: What about family? Did you participate when there is *Mahiber*?

**Interviewee**: Yes

**Interviewer**: When there is mourning, wedding

**Interviewee**: I participate on wedding to.

**Interviewer**: Do you participate at all?

**Interviewee**: Yes

**Interviewer**: Okay. Are you fine now? Don’t you seize while taking the medication?

**Interviewee**: Yes, thanks to God.

**Interviewer**: What about at the time you had been seizing?

**Interviewee**: Once the medication dose was 100 mg and then the health professionals reduced about thirty mg for trial. In the middle, they were very happy and they told me to come back and consult us if I had seizure. Then they told me to come even though I have the medication and take the regular one. In the middle, when I felt sick, I came up and consulted them,

**Interviewer**: How did you feel?

**Interviewee**: Your body may have vibration.

**Interviewer**: Yes. Is it before the seizure came?

**Interviewee**: No, it is not. It was while I took it first it was reducing and they told me as it is reduced. I was scared first when I thought about it, that medication was completely good. Then I felt sick and I got that back.

**Interviewer**: Did you feel vibration?

**Interviewee**: Yes

**Interviewer**: Did you go back when you were sick?

**Interviewee**: Yes, I came here and took the first medication.

**Interviewer**: Okay. You told me as you have a variety of symptoms; which of these symptoms has affected you the most?

**Interviewee**: It can’t be said that this doesn’t cause that much problem.

**Interviewer**: Are all problematic?

**Interviewee**: Yes

**Interviewer**: But, what you say that it is very difficult for me

**Interviewee**: The epilepsy

**Interviewer**: The epilepsy?

**Interviewee**: Yes, you don’t know anything because you can’t be able to decide to take a break, sit down and do something.

**Interviewer**: So, do you wish to get rid of it when you were sick first?

**Interviewee**: That is faith.

**Interviewer**: Have you ever been hurt when you fall?

**Interviewee**: Yes

**Interviewer**: Have you ever fall in risky place?

**Interviewee**: Yes

**Interviewer**: For example

**Interviewee**: It is a lot. The place we live is cliff, it is like round house. Generally, it is cliff and I work there which scare you. First, when burn fire you will be hurt if there is no one. The fire burnt a little bit since I work at preparing charcoal and I am not injured that much since there were people.

**Interviewer**: I don’t understand you, tell me more

**Interviewee**: I was preparing charcoal.

**Interviewer**: While you were preparing charcoal?

**Interviewee**: Yes, I work at rural area.

**Interviewer**: I understand you. Is that when you became sick while preparing the charcoal?

**Interviewee**: Yes

**Interviewer**: Did you fall?

**Interviewee**: Yes, I fall. I wasn’t injured much since there were individuals.

**Interviewer**: Would everything burn if there is no one?

**Interviewee**: Yes, to certain extent. It is not also good when you are very tired. It is not good for this disease when there is excessive fatigue. So, that is what creates much trouble to me.

**Interviewer**: Is it the seizure?

**Interviewee**: Yes

**Interviewer**: Do you feel like that when you are tired now?

**Interviewee**: No, I don’t feel like that since the work doesn’t make you tired that much.

**Interviewer**: Is it not that much exhausting?

**Interviewee**: Yes

**Interviewer**: What else? Have you ever fallen and injured your body?

**Interviewee**: No

**Interviewer**: I mean in the past.

**Interviewee**: No

**Interviewer**: Were you not injured?

**Interviewee**: No

**Interviewer**: Okay. My other question is that you told me as you got treatment and you told me that you got improvement by the treatment

**Interviewee**: Yes

**Interviewer**: Did you go for other treatment other than this modern treatment?

**Interviewee**: No

**Interviewer**: People try different things when they are sick, such as holy water.

**Interviewee**: No

**Interviewer**: Other like holy water

**Interviewee**: No

**Interviewer**: Didn’t you try anything?

**Interviewee**: Indeed, I drink holy water; I will go to church and drink holy water. But there is nothing else.

**Interviewer**: Do you stop taking the medication when you go to holy water?

**Interviewee**: I don’t stop.

**Interviewer**: Do you take it together?

**Interviewee**: Yes

**Interviewer**: Do you think there is a problem with taking more than one?

**Interviewee**: I don’t think so.

**Interviewer**: Some people stop taking it when they go to holy water.

**Interviewee**: I will go and I drink holy water. I get up in the morning and I drink holy water but I don’t stop taking medication. I also have no desire to stop taking the medication.

**Interviewer**: Is it since you have improvement?

**Interviewee**: Yes

**Interviewer**: So, don’t you want to stop taking it?

**Interviewee**: Yes

**Interviewer**: Do you think taking the medication improved my life?

**Interviewee**: Yes

**Interviewer**: Can you tell me how it improved your life? You told me some of it, if you have anything to add.

**Interviewee**: Yes, I am free from the seizure at this time. I don’t have anything to worry about; I can do what I want. I don’t think I will be in danger.

**Interviewer**: Don’t you worry?

**Interviewee**: Yes, I don’t worry as it helps me completely.

**Interviewer**: Okay, you told me as you didn’t take anything other than the medication

**Interviewee**: Yes

**Interviewer**: Okay. Some people often find it difficulty when they are sick; have you experienced things like that?

**Interviewee**: Yes

**Interviewer**: Like wearing their cloth, cooking their food, eating food; some people find it difficult to do such type of things.

**Interviewee**: Yes

**Interviewer**: Did you experience like that?

**Interviewee**: No

**Interviewer**: When you were ill?

**Interviewee**: Before that?

**Interviewer**: Yes

**Interviewee**: How to dress?

**Interviewer**: Yes

**Interviewee**: No

**Interviewer**: Eating food

**Interviewee**: It is when I am sick otherwise I don’t have such type of thing.

**Interviewer**: Did you become normal when you wake up?

**Interviewee**: I didn’t experience anything. I don’t have any problem.

**Interviewer**: You were treated at *Butajira* first, right?

**Interviewee**: First

**Interviewer**: How did you find it there? Who is there?

**Interviewee**: There?

**Interviewer**: How were the doctors?

**Interviewee**: They cared and welcomed us a lot.

**Interviewer**: A lot?

**Interviewee**: In addition, they will write me a referral when there is no medication. They will not give me the medication if they don’t write referral to me.

**Interviewer**: Did they take adequate time to talk to you about the problem and the medication when you go there?

**Interviewee**: Yes, they ask me both at here and there. They will ask me on a monthly basis when I come here. If I go there in two months, they ask me about the two months and if I go there in a month, they ask me about that.

**Interviewer**: Do you go to the pharmacy or do you go to the doctor? Do you go to the doctor first and then to the pharmacy?

**Interviewee**: I go to the doctor first and then he asks me questions about the improvement and then he prescribes me medication.

**Interviewer**: Yes. Do you take the medication then?

**Interviewee**: Yes

**Interviewer**: Okay. Was the health professional treated you was polite?

**Interviewee**: Yes

**Interviewer**: Did you ask what you want?

**Interviewee**: Yes

**Interviewer**: Was there something you were upset when you went there?

**Interviewee**: There is nothing.

**Interviewer**: Sometimes they rush up, there is such type of thing and people don’t want to go to them as they think they don’t treat well.

**Interviewee**: There is no such type of thing.

**Interviewer**: Did you experience such type of thing?

**Interviewee**: We are human and we make mistakes sometimes.

**Interviewer**: Yes

**Interviewee**: Once referral paper was written for me and no one could accept me, it could me my weakness.

**Interviewer**: Didn’t you see them the paper?

**Interviewee**: I showed them but they said we don’t know and at that time I felt upset. The doctor prescribed me the medication to the pharmacy. The chief doctor was not required at that time, I just knew at that time that I was the beneficiary and he also knew as I was there. I had also the paper and then they said we don’t give you and I was upset. Sometimes when I am really in trouble, I buy the medication from outside.

**Interviewer**: Did you send for medication from the outside?

**Interviewee**: Yes, I bought it from the outside at *Butajira*. I buy from outside when I come there. When he said I will not give you, I will buy from outside. When the medication is not available at *Butajira*, I will buy from outside that is my option. It has additional payment and it doesn’t go with my capacity. You can’t do anything; it is a matter of life. Then he said I am sorry I didn’t see him sincerely. No one didn’t show me because I didn’t know as who can help me.

**Interviewer**: Yes

**Interviewee**: At the end I went to the head and told him about that and I also showed him the paper. Then I went back to the doctor and he said why you went to him, with him did you go and who showed you, I felt a lot at that time because no one should be asked like that. That was my first experience, then he gave me the medication but he was not happy. I was hurt because he was not happy. Care has to be given not only for this disease but for any type of accident. For example, our mother is tired and it is necessary to help her more than ourselves. I told him that as I would not worry him if the disease not acute. At the end he just gave me the medication and I brought it. I have never had anything other than that. To tell you the truth, sometimes I came here at Saturday without knowing that and they gave me at least two days medication. Last time, I run out of medication and didn’t take it. I got him at *Kela* when I went to buy the medication but there was no medication there and he called to *Butajira*. Then God bless them, they called me here and they also knew as I used medication. I also came in the evening on Saturday. It was four o’clock in the evening and then I went on Sunday and they gave me two days medication and I came the next day and took the medication. They are very supportive of me. I have never encountered anything other than this.

**Interviewer**: Okay. Did you forget and stop taking it for a long time?

**Interviewee**: No, I don’t forget for one day. For example, today is Friday and I may take it Friday and forget to take it Saturday.

**Interviewer**: No, while you have the medication?

**Interviewee**: No, I don’t discontinue.

**Interviewer**: At what time do you take it? Is it in the morning or evening?

**Interviewee**: In the evening.

**Interviewer**: In the evening?

**Interviewee**: Yes, it is in the evening.

**Interviewer**: Okay. What did they tell you about the medication you are taking?

**Interviewee**: No, it is in the evening.

**Interviewer**: Did they say take it in the evening?

**Interviewee**: Yes

**Interviewer**: What else did they tell you?

**Interviewee**: To don’t drink alcohol.

**Interviewer**: What about the medication?

**Interviewee**: Take it once.

**Interviewer**: Did they say take it once?

**Interviewee**: Yes

**Interviewer**: If you have been told about the medication like take it before or after meal

**Interviewee**: It doesn’t bother me much since I know how to take it.

**Interviewer**: What did they tell you at that time?

**Interviewee**: It is before meal for the first time.

**Interviewer**: Did they tell you to take it before meal?

**Interviewee**: Yes

**Interviewer**: Did you take the same type of medication since the beginning or was the medication changed?

**Interviewee**: Yes, it is the same type of medication.

**Interviewer**: Is it?

**Interviewee**: Yes

**Interviewer**: Is it hundred milligrams?

**Interviewee**: Yes

**Interviewer**: Wasn’t the medication changed?

**Interviewee**: I told you as it had been changed once.

**Interviewer**: Was it the gram that changed?

**Interviewee**: Yes, the gram.

**Interviewer**: No, I want to say the color of the medication. What is the color of the medication of that you are taking?

**Interviewee**: The white.

**Interviewer**: The white?

**Interviewee**: Yes

**Interviewer**: Have you been taking the white till?

**Interviewee**: Yes

**Interviewer**: Okay. Did it have improvement?

**Interviewee**: Yes

**Interviewer**: Did it have good improvement?

**Interviewee**: Yes

**Interviewer**: Have you never fall since you start taking the medication?

**Interviewee**: There is nothing.

**Interviewer**: Nothing?

**Interviewee**: Yes

**Interviewer**: Don’t you have the depression?

**Interviewee**: Nothing?

**Interviewer**: So, are you happy about the medication?

**Interviewee**: Yes

**Interviewer**: Okay. The other thing is that the health professionals may ask you about your personal life when you go to bring the medication.

**Interviewee**: They will ask me.

**Interviewer**: What did you feel when they asked you?

**Interviewee**: I didn’t feel anything. They ask me about my work, symptoms and about the medication. I am taking it properly and don’t be worried since the medication helped me. I don’t so anything that troubles them.

**Interviewer**: No, if you have other illness they may ask you question like if you have suicidal ideation

**Interviewee**: No, they didn’t ask me.

**Interviewer**: The other thing is, did they ask you if you have you feeling of depression? Did they ask you such type of personal life?

**Interviewee**: They didn’t ask me anything.

**Interviewer**: Don’t you feel if they ask you such type of questions?

**Interviewee**: I don’t feel anything.

**Interviewer**: Why?

**Interviewee**: Because the medication helps me. The medication is helpful and it doesn’t have any side effects.

**Interviewer**: What if it is harmful?

**Interviewee**: For the medication to don’t be harmful, I will take it properly. I don’t have anything that upset me, I am happy. They also support me and there is nothing that harms me.

**Interviewer**: Okay. You told me as someone took you to *Butajira* health center.

**Interviewee**: Yes

**Interviewer**: Didn’t you have a gap for a month before that person came?

**Interviewee**: Where? There?

**Interviewer**: Yes

**Interviewee**: I didn’t go.

**Interviewer**: Did you get treatment?

**Interviewee**: Yes, it is like that.

**Interviewer**: Is it more than a month?

**Interviewee**: Yes, it was more than a month.

**Interviewer**: Did you stay more than a month

**Interviewee**: Yes

**Interviewer**: Didn’t you go since you hadn’t the information?

**Interviewee**: They didn’t prescribe the medication.

**Interviewer**: Didn’t they prescribe you?

**Interviewee**: I can’t at that time.

**Interviewer**: You may not know, but what about family?

**Interviewee**: I told you that my grandmother also can’t see.

**Interviewer**: I mean other family member.

**Interviewee**: They don’t remember such type of thing. They only care about their life but they don’t worry about me.

**Interviewer**: So, is it by you?

**Interviewee**: Yes, it is like that.

**Interviewer**: What about your family support at this time?

**Interviewee**: Family doesn’t support me in the past and at this time too.

**Interviewer**: No. for example, you have wife and children.

**Interviewee**: Yes

**Interviewer**: Do they support you?

**Interviewee**: Yes, they support me.

**Interviewer**: What type of support, for example, regarding your illness?

**Interviewee**: First, to don’t forget to take my medication and they remember me to take it on time.

**Interviewer**: Okay

**Interviewee**: To don’t forget taking it.

**Interviewer**: Your family; your wife and children.

**Interviewee**: Yes, my children remind me to don’t forget it. They bring me water since I take medication. They support me what they can. They will put water to my bed room and my wife also cares me much.

**Interviewer**: What else do they do for your illness to don’t relapse?

**Interviewee**: All of them don’t want me to drink alcohol as they know it will hurt me and I don’t also have interest to drink alcohol.

**Interviewer**: What about chewing *Khat*?

**Interviewee**: There is nothing.

**Interviewer**: Is there nothing?

**Interviewee**: Yes

**Interviewer**: So, are your families supporting you now?

**Interviewee**: Yes

**Interviewer**: Okay. Did you marry before you were ill or was it before that?

**Interviewee**: After I became ill.

**Interviewer**: After you became ill?

**Interviewee**: Yes

**Interviewer**: Did you encounter problem on getting married because of the epilepsy? Some people say that it the disease that kept me from getting married.

**Interviewee**: I didn’t have that kind of problem, she knew about it from the beginning.

**Interviewer**: Did she know?

**Interviewee**: She knew from the beginning. She knew as I use medication since we live in the same place.

**Interviewer**: So, what about the fear that it will transmit to me?

**Interviewee**: It is me that fear, they don’t fear about that since they don’t care if it is transmitted or not. But I used to be worried about what will happen to my family, my son, and my neighbor, but know I am sure I am not going to seize.

**Interviewer**: Did you worry that it will transmit to my children?

**Interviewee**: I was scared every time when they rolled over my body. I will not tell them since I don’t want my children to worry. Children don’t forget the thing they hear once, so I had that fear before.

**Interviewer**: Did you reduce your contact with your children so that it would not be transmitted to them?

**Interviewee**: Yes, I was scared but I couldn’t do anything about it. What can you do, children are the gift of God. It is God who gave me them, you can’t do anything. I worried that if it is transmitted to my children and if they will be harmed as well.

**Interviewer**: So to don’t be like that?

**Interviewee**: But now

**Interviewer**: Don’t you worry about that now?

**Interviewee**: I don’t worry.

**Interviewer**: Didn’t you play with your children so that it would not pass it to them?

**Interviewee**: Yes

**Interviewer**: Did you isolate yourself?

**Interviewee**: No, I am just worried. It is more terrifying if I transmitted to my children and then told them.

**Interviewer**: Didn’t tell that to your children?

**Interviewee**: There are incidental diseases like common cold headache; I just thought it was like that which will transmit to them when they approached me. I just thought like that, I didn’t understand it.

**Interviewer**: So, did you restrict your contact with them?

**Interviewee**: Yes

**Interviewer**: Is that in the past?

**Interviewee**: Yes, I was very scared, especially when my children rolled over me.

**Interviewer**: What do your family think about the treatment?

**Interviewee**: She just wants me to follow-up my treatment properly.

**Interviewer**: Do they think it will help you?

**Interviewee**: They think so much.

**Interviewer**: Do they support you like you told me before?

**Interviewee**: Yes

**Interviewer**: What kind of additional treatment do you think that you may need to have more improvement?

**Interviewee**: It is good if there is another treatment besides this medication. But I think there is nothing like that.

**Interviewer**: Is it enough? Do you think that this medication make me equal to anyone else who don’t have this disease?

**Interviewee**: It is enough for me. It is really enough for me, I have no any problem.

**Interviewer**: Like your friends? Like what everybody wants to do?

**Interviewee**: It with any people because I can go to where my friends are going as I have nothing to worry about.

**Interviewer**: So, don’t you think I need any better treatment?

**Interviewee**: I don’t think so.

**Interviewer**: Is this enough?

**Interviewee**: Yes

**Interviewer**: Okay, let’s finish this and what do you think should be done to improve the lives of not only you but also the lives of people with epilepsy?

**Interviewee**: The first thing we need to do is stop taking alcohol while taking medication because it is useless if you drink alcohol with medication. Just like you said, there were my friends who drank alcohol while taking medication. They drank alcohol, but it doesn’t help them. It is the medication that helps us.

**Interviewer**: When you say it is useless will they seize?

**Interviewee**: It will seize them because the medication can’t work if they drink alcohol. They are not taking it properly. If they had taken the medication properly and it doesn’t have any improvement, they can consult health professional. So when the two are taken together, I think it will be more stressful.

**Interviewer**: So should the community be advised?

**Interviewee**: Yes

**Interviewer**: Who should advise them?

**Interviewee**: Health professionals.

**Interviewer**: They didn’t hear when the health professionals advise them, right? So, who should advise them to make their lives better?

**Interviewee**: I advise the boy who was there. As I told you before, the medication will runs out and I will bring hundred milligrams from him and take it.

**Interviewer**: Is it because he doesn’t take it?

**Interviewee**: No, when I run out of medication.

**Interviewer**: Okay

**Interviewee**: For example, he may bring in the week I brought it.

**Interviewer**: Yes, I understand you.

**Interviewee**: So, I will use two pills from him and the gram is also the same as the one he brought it.

**Interviewer**: Is the medication similar?

**Interviewee**: Yes, the medication is similar so I will use it. He doesn’t use it by his will. Sometimes he takes two pills, I think it is to check whether it is beneficial or not.

**Interviewer**: Without physician order?

**Interviewee**: Yes, but that medication doesn’t helps him. So, the problem is taking alcohol with medication. The problem is not using the medication properly, but they could consult health professional. They should say it don’t help me if it doesn’t help them. For example, I will consult health professionals when the does reduced as it is not helpful for me. The main thing to prdo with such treatment is to do what the health professionals advised.

**Interviewer**: What do the health professionals do?

**Interviewee**: It also helps the health professional because when he explains the problem the physician may increase or decrease the medication dose. So what the patients have to do it explaining the problem for the health professionals.

**Interviewer**: Okay, generally there are a lot of problems with our life; there could be many problems in our social life, our work and education. People will not fulfill everything in their life, right?

**Interviewee**: Yes

**Interviewer**: People with epilepsy will have more problems than the others and you told me to as they should take the medication properly and they don’t have to take alcohol first. What else can health professionals do in addition to what they are doing now to improve the lives of people with epilepsy?

**Interviewee**: The health professionals should inform about life style. Everybody also knows how much harm this disease has and as they can’t work for living. They have to follow-up properly and they have to inform for health professionals

**Interviewer**: Who should search and tell?

**Interviewee**: It is the community. For example, the community knows about the existing problem. The family also knows whether I am using it properly or not. No one can know whether a person take the medication or not. So, it is up to the health professionals and the patient who should understand the problem.

**Interviewer**: Since they saw the improvement?

**Interviewee**: Yes, first the patient should see the improvement and explain the problem to the health professional. Secondly, it is the responsibility of the caregivers to respond appropriately when the health professionals asks them. So, the health professionals should study such type of things.

**Interviewer**: What should the community do?

**Interviewee**: Whenever possible, it is important if the family, the husband and the community support when a person is troubled but a lot of people don’t do that because, for example, nobody knows as I have the disease. I mean, a lot of people don’t know as I am like this. Let me tell you, for example if I go to Addis Ababa no one will know me. There are many children who don’t know the main road and I felt a lot when they fall. I feel so bad about them; it is not about my life. At that time I knew what is good and what is bad for me. For example, I will be beneficiary when I take the medication, but I am sure as they don’t take the medication. At that time, they may give them water but no one will advise them to take their medication properly. Only the health professionals are worried about this but I don’t think the communities have responsibility too.

**Interviewer**: So, should they have to educate the community?

**Interviewee**: They should, everything have to be with the community and the health professionals. It is beneficial but not harmful.

**Interviewer**: What should the health institutions do?

**Interviewee**: They have to study and they have to ask how it is done. They should ask them in order for them to take it properly. They have to monitor them as that person may not take it intentionally. There may be a lot of people who take the medication but don’t benefit from it. For example, the dose may not be balanced with the disease which will not be important. There are a lot of people who say I will not go back there since they don’t know about it. I also know some people, who don’t go, but that should not have to be; they have to go to health professionals and understand it well.

**Interviewer**: Do they have to explain it well?

**Interviewee**: Yes, they have to.

**Interviewer**: Do they have to explain about the disease well?

**Interviewee**: Yes, they have to.

**Interviewer**: So, do they have to explain it well there?

**Interviewee**: They have to.

**Interviewer**: Do you have anything to add?

**Interviewee**: No

**Interviewer**: If you don’t have, I am done. Okay, thank you
